# An atlas of associations between 14 micronutrients and 22 cancer outcomes: Mendelian randomization analyses

**DOI:** 10.1186/s12916-023-03018-y

**Published:** 2023-08-21

**Authors:** Jong Yeob Kim, Minku Song, Min Seo Kim, Pradeep Natarajan, Ron Do, Woojae Myung, Hong-Hee Won

**Affiliations:** 1 Samsung Advanced Institute for Health Sciences & Technology (SAIHST) Sungkyunkwan University, Samsung Medical Center, 81 Irwon-Ro, Gangnam-Gu, Seoul, 06351 Republic of Korea; 2 Yonsei University College of Medicine, Seoul, Republic of Korea; 3 Medical and Population Genetics and Cardiovascular Disease Initiative Broad Institute of Harvard and MIT, Cambridge, MA USA; 4 Cardiovascular Research Center Massachusetts General Hospital, Boston, MA USA; 5 Department of Medicine, Harvard Medical School, Boston, MA USA; 6 The Charles Bronfman Institute for Personalized Medicine, Icahn School of Medicine at Mount Sinai, New York, NY USA; 7 Department of Genetics and Genomic Sciences, Icahn School of Medicine at Mount Sinai, New York, NY USA; 8 Department of Neuropsychiatry, Seoul National University Bundang Hospital, Seongnam, Republic of Korea; 9 Samsung Genome Institute, Samsung Medical Center, Sungkyunkwan University School of Medicine, Seoul, Republic of Korea

**Keywords:** Micronutrient, Mineral, Vitamin, Cancer, Mendelian randomization

## Abstract

**Background:**

Micronutrients, namely vitamins and minerals, are associated with cancer outcomes; however, their reported effects have been inconsistent across studies. We aimed to identify the causally estimated effects of micronutrients on cancer by applying the Mendelian randomization (MR) method, using single-nucleotide polymorphisms associated with micronutrient levels as instrumental variables.

**Methods:**

We obtained instrumental variables of 14 genetically predicted micronutrient levels and applied two-sample MR to estimate their causal effects on 22 cancer outcomes from a meta-analysis of the UK Biobank (UKB) and FinnGen cohorts (overall cancer and 21 site-specific cancers, including breast, colorectal, lung, and prostate cancer), in addition to six major cancer outcomes and 20 cancer subset outcomes from cancer consortia. We used sensitivity MR methods, including weighted median, MR-Egger, and MR-PRESSO, to assess potential horizontal pleiotropy or heterogeneity. Genome-wide association summary statistical data of European descent were used for both exposure and outcome data, including up to 940,633 participants of European descent with 133,384 cancer cases.

**Results:**

In total, 672 MR tests (14 micronutrients × 48 cancer outcomes) were performed. The following two associations met Bonferroni significance by the number of associations (*P* < 0.00016) in the UKB plus FinnGen cohorts: increased risk of breast cancer with magnesium levels (odds ratio [OR] = 1.281 per 1 standard deviation [SD] higher magnesium level, 95% confidence interval [CI] = 1.151 to 1.426, *P* < 0.0001) and increased risk of colorectal cancer with vitamin B12 level (OR = 1.22 per 1 SD higher vitamin B12 level, 95% CI = 1.107 to 1.345, *P* < 0.0001). These two associations remained significant in the analysis of the cancer consortia. No significant heterogeneity or horizontal pleiotropy was observed. Micronutrient levels were not associated with overall cancer risk.

**Conclusions:**

Our results may aid clinicians in deciding whether to regulate the intake of certain micronutrients, particularly in high-risk groups without nutritional deficiencies, and may help in the design of future clinical trials.

**Supplementary Information:**

The online version contains supplementary material available at 10.1186/s12916-023-03018-y.

## Background

Numerous studies have addressed the effects of micronutrients, particularly vitamins and minerals, on various health outcomes, including cancer. Observational studies have frequently reported the benefits of micronutrient supplementation on cancer risks [1–7], whereas randomized controlled trials (RCTs) have often reported a null effect [8–10]. However, observational studies are inherently prone to confounding and reverse causation, and RCTs are expensive and often insufficiently powered for cancer outcomes, requiring long-term follow-up and large cohort sizes. A systematic review conducted in 2013 reported a paucity of fair- or good-quality studies assessing the associations between micronutrient supplementation and cancer and concluded that there was a lack of evidence to support micronutrient supplementation for cancer prevention [9]. The Mendelian randomization (MR) approach attempts to overcome these limitations by using genetic variants as instrumental variables (IVs) to assess the potential causal association between risk factors and disease [11].

In the past decade, many health benefits of micronutrients reported in observational studies have been shown to have null causal associations in MR studies. For example, although vitamin D is a promising micronutrient with statistically significant beneficial effects on various malignant, cardiovascular, metabolic, and other diseases [12], over 60 MR studies published in the previous decade found no effect of genetically predicted vitamin D concentrations on most health outcomes [13]. Numerous MR studies reporting associations between various micronutrients and the risk of various cancers [14–17] revealed that only a few exposure-outcome pairs were potentially genuine associations. Furthermore, MR methods are not consistent across studies, making it difficult to compare the robustness of associations. According to a recent systematic review, most MR studies assessing cancer outcomes did not adequately perform sensitivity analyses assessing the pleiotropy of MR associations, such as MR-PRESSO, resulting in potentially biased estimates [18], and many previous MR studies reporting micronutrient-cancer associations [15–17, 19–22] chose IVs under linkage disequilibrium (LD) thresholds less strict than the conventionally used threshold of *r*^2^ < 0.001, resulting in potentially biased estimates. To overcome these limitations and clarify the presence and robustness of causal associations, we performed exposure-wide and outcome-wide MR analyses of 14 micronutrients and 48 cancer outcomes.

## Methods

### Study design

This study was conducted in accordance with STROBE-MR (Strengthening the Reporting of Observational Studies in Epidemiology using Mendelian Randomization) guideline (Additional files 1 and 2) [23]. We conducted a two-sample MR study using single-nucleotide polymorphisms (SNPs) associated with various micronutrient levels as IVs to assess the causal association of 14 micronutrients with 48 cancer outcomes (overall cancer or 21 site-specific cancers) [11]. We used publicly available summary statistics data from the largest available genome-wide association studies (GWASs) for various micronutrients and GWASs from the UK Biobank (UKB) study, FinnGen study, and various cancer consortia [24–28] for cancer outcomes. All the GWAS cohorts were of European descent. There was no overlap between the exposure and outcome cohorts. MR relies on the following three assumptions: first, genetic predictors of the exposure of interest are strongly associated with exposure; second, genetic predictors of exposure are not correlated with exposure-outcome association confounders; and third, genetic predictors affect the outcome only by affecting the exposure of interest [29]. All analyses were based on publicly available non-individual-level data; therefore, no ethical approval from an ethics committee was required.

### Genetic associations with micronutrients

Genetic associations with micronutrients were obtained from the largest available GWAS in the European population, identifying SNPs associated with each micronutrient at a genome-wide significance threshold (*P* < 5 × 10^−8^). We systematically searched for GWASs in PubMed and screened the references of relevant articles (details in Additional File 1). When two or more independent GWASs were available for exposure, the GWAS with the highest number of participants was selected. GWASs for seven essential minerals (serum calcium, copper, iron, magnesium, phosphorus, toenails, blood selenium, and zinc) and seven vitamins (25-OH vitamin D, vitamins A1 [retinol], B6, B9 [folic acid], B12, C, and E) were identified (Additional File 2: Table S1) [30–41]. For IVs representing iron status, we selected three SNPs known to show concordant effects on serum iron, ferritin, transferrin, and transferrin saturation and consistent effects on overall iron status [30, 42]. For IVs representing vitamin C status, a GWAS study [41] reported 11 SNPs, and we selected 10 SNPs after excluding one SNP (rs174547) reported to have pleiotropic effects on the *FADS1* gene, which is associated with a large number of glycerophospholipids or sphingolipids. Two GWASs were available for selenium levels [31, 32], and we selected the one with the largest number of participants [31] for our analysis. All GWASs measured serum micronutrient levels, except for selenium, which was measured in either the toenails or blood. Potassium, sodium, and vitamins B1, B2, and K were excluded because of a lack of GWAS reporting SNPs of genome-wide significance [38, 43].

### Genetic associations with cancer

We identified 22 cancer outcomes for which GWAS summary statistical data were available for both the UKB (Lee Lab GWAS [44] or Neale Lab [45] GWAS if Lee Lab GWAS was not available) and FinnGen studies, R9 release [46]. The resulting cancer outcomes were overall cancer and 21 major site-specific cancers: bladder, brain, breast, cervical, colorectal, Hodgkin’s lymphoma, kidney, leukemia (lymphoid or myeloid), liver, lung, melanoma, multiple myeloma, esophageal, oral and pharyngeal, ovarian, pancreatic, prostate, stomach, testisticular, thyroid, and uterine cancers. For each cancer outcome, we meta-analyzed summary statistics from the UKB and FinnGen cohorts using METAL software [47]. The UKB and FinnGen studies are ongoing cohorts containing data from approximately 500 thousand and 390 thousand participants, respectively, of European descent. The combined outcome summary statistics included up to 940,633 participants and 124,092 cases of overall cancer, ranging from 992 to 27,554 site-specific cancer outcomes.

To replicate and strengthen our findings in other databases, we identified six cancer outcomes from the updated data provided by various cancer consortia, performed additional MR analyses using identical exposure datasets, and compared their findings with those of MR analyses using the UKB and FinnGen cohorts. The six additional cancer outcomes were as follows: breast cancer, derived from the Breast Cancer Association Consortium (BCAC) [24]; colorectal cancer, from the meta-analysis of studies including the Genetics and Epidemiology of Colorectal Cancer Consortium (GECCO) [25]; lung cancer, from the Transdisciplinary Research of Cancer in Lung of the International Lung Cancer Consortium (TRICL-ILCCO) [26]; invasive and non-invasive ovarian cancer, from the Ovarian Cancer Association Consortium (OCAC) [27]; and prostate cancer, from the Prostate Cancer Association Group to Investigate Cancer-Associated Alterations in the Genome (PRACTICAL) consortium [28]. The number of cancer cases ranged from 3103 to 133,384, and an additional 20 cancer subsets were available from the consortia for breast, lung, and ovarian cancers.

### Selection of genetic instruments

Of the SNPs obtained from the GWASs of micronutrient levels, we further selected only SNPs with a minor allele frequency > 0.01, which were not in LD (*r*^2^ < 0.001 and clumping window within 10,000 base pairs based on European 1000 Genomes Project reference panel) for MR analyses. We replaced SNPs that were not available in the outcome summary statistics or that were palindromic with non-inferable allele frequencies (minor allele frequency > 0.42) with SNPs in LD (*r*^2^ > 0.8). We calculated MR estimates per 1 standard deviation (SD) difference at the micronutrient or log-transformed micronutrient level. We estimated *F*-statistics representing the strength of the association between IVs and exposure [48] and estimated the variance of each exposure explained by the IVs (details provided in Supplementary Methods) [49]. Details of the eligible IVs and micronutrient GWAS cohorts are reported in Supplementary Tables S1-2. For each association, we assessed the minimum detectable odds ratio (OR) assuming a statistical power of 80%. The statistical power assuming ORs of 1.1, 1.3, and 1.6 was also calculated, in conformance with previous MR studies [50–52].

### Statistical analysis

We performed MR analysis for each exposure-outcome pair, resulting in multiple tests for a total of 672 associations (14 exposures × 22 UKB plus FinnGen cancer outcomes, 6 main cancer outcomes, and 20 cancer subset outcomes from cancer consortia). The random-effects inverse-variance weighted (IVW) method was used to obtain a summary of the associations. The IVW method assumes that all genetic variants are valid; thus, it is prone to bias when a large portion of IVs are subject to horizontal pleiotropy [53]. Statistical significance was set at* P* < 0.05, and the Bonferroni correction was applied (*P* < 0.0036 based on alpha = 0.05/14 available exposures and *P* < 0.00016 based on alpha = 0.05/14 × 22 exposure-outcome pairs) for multiple testing. To test for evidence of horizontal pleiotropy, we performed sensitivity MR analyses under varying assumptions, including the weighted median, MR-Egger, and MR-PRESSO methods. The weighted-median method assumes that the majority of IVs are valid and is considered robust when the percentage of horizontal pleiotropic IVs is < 50% [54]. The MR-Egger method is reliable when more than 50% of the IVs are subject to horizontal pleiotropy [55]. We further inspected horizontal pleiotropy using the Egger regression intercept and outlier correction by MR-PRESSO methods [56] and inspected visual asymmetry in funnel plots [57]. We assessed heterogeneity using Cochran’s *Q*-test and *I*^2^ and visually inspected heterogeneity using scatter plots [57]. We examined whether the IVs were causally associated with major risk factors for cancer, such as body mass index (BMI), weight circumference, physical activity, and smoking, using corresponding GWAS summary data [58–61]. All statistical analyses used R version 4.0.5 (R Foundation) and its package “*TwoSampleMR*” and “*MRPRESSO*.”

## Results

**Fig. 1:**
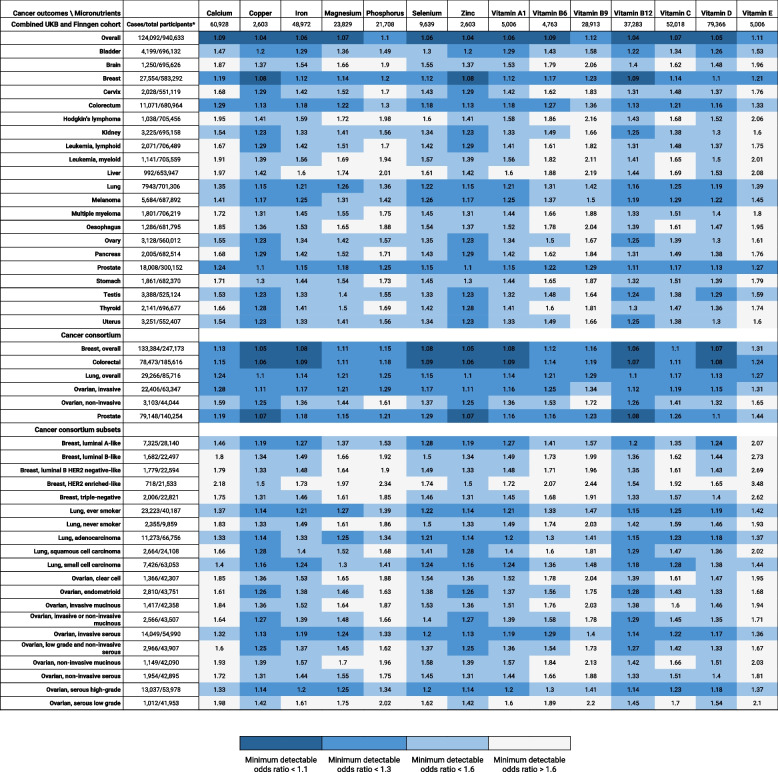
Minimum detectable odds ratios assuming statistical power of 80% for the 672 micronutrient-cancer associations. * The number of cancer cases and total participants are listed for cohorts reporting cancer outcomes, and the number of total participants is listed for cohorts reporting micronutrient levels

For the 14 exposures, there were one to nine available IVs, and the *F* statistics ranging from 11.3 to 544, which were well above the recommended value of 10 (Additional file 2: Tables S1 and S2). The variance in exposure explained by the IVs ranged from 0.6 to 4.6%. The number of cases and total participants in each cohort and the minimum detectable ORs, assuming 80% power for the 672 associations, are shown in Fig. 1.

### Analyses of UKB plus FinnGen cancer outcomes

**Fig. 2:**
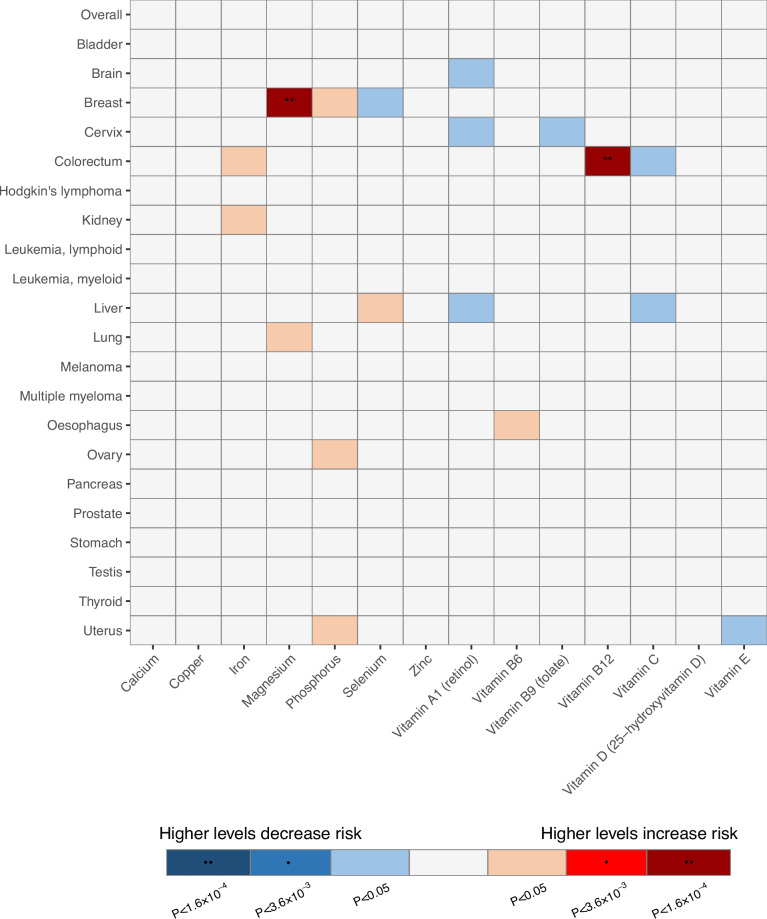
Results of Mendelian randomization associations of micronutrients with cancer outcomes from combined UK Biobank and FinnGen cohort. * Bonferroni-corrected significance threshold by the number of exposures. ** Bonferroni-corrected significance threshold by the number of exposures-outcome pairs

**Fig. 3:**
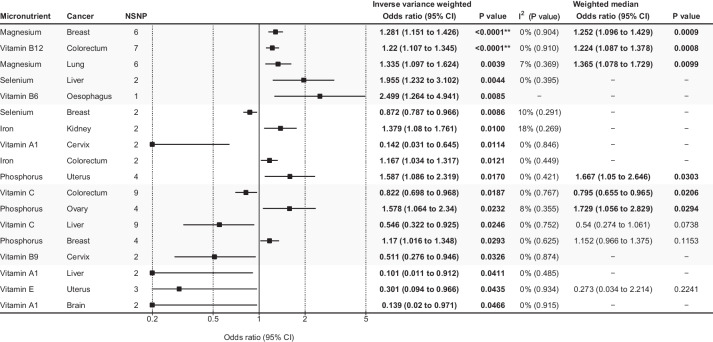
Statistically significant associations between micronutrient levels and cancer outcomes in UK Biobank and FinnGen meta-analysis. Associations are listed in ascending order of *p*-values in the inverse variance-weighted model. The forest plot represents the pooled odds ratio under the inverse variance-weighted model. For the association supported by 1 instrumental variable (vitamin B6-esophagus cancer association), the Wald ratio was reported instead of the pooled inverse variance weighted odds ratio. A weighted median analysis was not available for the associations supported by two or fewer instrumental variables. Details of all the statistical results are shown in Supplementary table 3. Abbreviations: CI, confidence interval; NSNP, number of single-nucleotide polymorphisms. ** The association showed Bonferroni-corrected significance by the number of exposure-outcome pairs (P < 0.00016)

Of the 308 associations included in the UKB and FinnGen meta-analyses (14 exposures × 22 outcomes), 18 were statistically significant in the IVW analysis (Figs. 2 and 3, Additional file 2: Table S3, and Additional file 1: Figs. S1-31), of which two associations showed Bonferroni-corrected significance based on the number of exposure-outcome pairs (*P* < 0.00016). There was an increased risk of breast cancer with magnesium levels (OR = 1.281 per 1-SD higher level, 95% CI = 1.151 to 1.426, *P* < 0.0001) and an increased risk of colorectal cancer with vitamin B12 levels (OR = 1.22 per 1 SD higher level, 95% CI = 1.107 to 1.365, *P* < 0.0001). These two associations had six and seven IVs, respectively, and their heterogeneity was low (*I*^2^ = 0% and Q statistics *P* > 0.1 for both associations). No evidence of pleiotropy was found using the MR-Egger intercept test or the MR-PRESSO test, and no outlier SNPs were detected (Additional file 2: Table S3). The two associations were statistically significant in the weighted median analysis, indicating that the association was significant even if some (< 50%) of the IVs were horizontally pleiotropic. Visual examination of the funnel and scatter plots revealed no obvious asymmetry or heterogeneity. For both micronutrient-cancer associations, all individual SNPs showed concordance of effect estimates (Additional file 1: Figs. S15-16).

The other 16 statistically significant associations were iron, magnesium, phosphorus, selenium, and vitamins A1, B6, B9, C, and E associated with various cancer outcomes, of which eight had decreased risks and eight had increased risks of cancer outcomes with higher micronutrient levels (Figs. 2 and 3). However, these associations did not show Bonferroni-corrected significance according to the number of exposures (*P* < 0.0036). Of the 308 tested associations, 15, 100, and 235 had > 80% power to detect ORs of 1.1, 1.3, and 1.6, respectively (Fig. 1; Additional file 2: Table S3). The power of the tested associations differed by cancer outcome, and the number of exposures that had > 80% power to detect an OR of 1.3 were 14 (all exposures) for overall cancer, breast cancer, and prostate cancer; 11 for colorectal cancer; and 9 for lung cancer (Fig. 1, Additional file 2: Table S3). Considering the overall cancer risk, no micronutrient levels showed a benefit or risk, although the minimum detectable ORs assuming 80% power were < 1.2 for all 14 associations. Some IVs were strongly associated with risk factors of cancer (*P* > 1 × 10^−5^). One IV for calcium (rs780094) and another for copper (rs2769264) were associated with BMI (Additional File 2: Table S4). However, calcium and copper levels were not significantly associated with any cancer outcome in the UKB plus FinnGen analysis or major cancer outcomes in the cancer consortia analysis.

### Analyses of consortia cancer outcomes

**Fig. 4:**
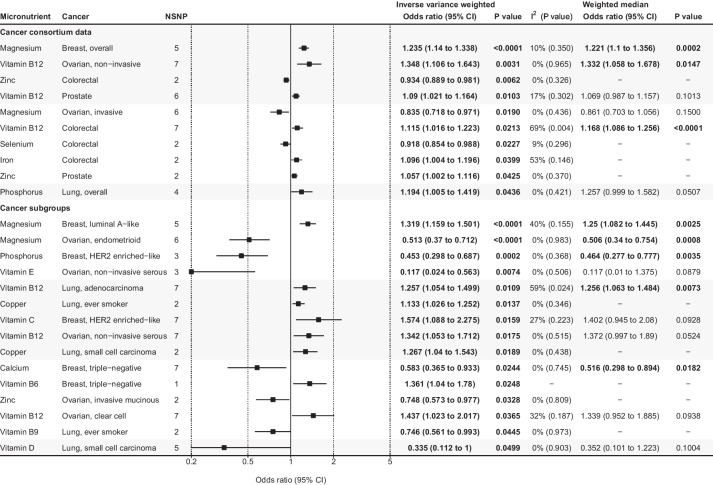
Statistically significant associations between micronutrient levels and cancer outcomes in cancer consortia studies. Associations are listed in ascending order of p-values in the inverse variance-weighted model. The forest plot represents the pooled odds ratio under the inverse variance-weighted model. For the association supported by 1 instrumental variable (vitamin B6-triple negative breast cancer association), the Wald ratio was used instead of the pooled inverse variance weighted odds ratio. A weighted median analysis was not available for the associations supported by two or fewer instrumental variables. Details of all the statistical results are shown in Supplementary table 3. Abbreviations: CI, confidence interval; HER2, human epidermal growth factor receptor 2; NSNP, number of single-nucleotide polymorphisms.

Of the 84 major cancer associations (14 exposures × 6 cancer outcomes), 10 were statistically significant (Fig. 4, Additional file 2: Table S3, and Additional file 1: Figs. S32-41). Two out of these associations, increased risk of breast cancer with magnesium levels (OR = 1.235 per 1 SD higher level, 95% CI = 1.14 to 1.338, *P* < 0.0001) and increased risk of colorectal cancer with vitamin B12 levels (OR = 1.115 per 1 SD higher level, 95% CI = 1.016 to 1.223, *P* = 0.0213), were statistically significant with similar effect sizes in both the cancer consortia and UKB plus FinnGen analyses (Figs. 3 and 4). Both associations were found to be significant using a weighted median analysis. Evidence of horizontal pleiotropy was identified for the vitamin B12-colorectal cancer association (MR-PRESSO P for horizontal pleiotropy = 0.014); however, the association retained its significance (OR = 1.171, 95% CI = 1.1 to 1.246, *P* = 0.004) after excluding the outlier SNP. The number of exposures that had > 80% power to detect an OR of 1.3 was 14 (all exposures) for colorectal and lung cancers, 13 for breast and prostate cancers, 12 for invasive ovarian cancer, and 3 for non-invasive ovarian cancer (Fig. 1, Additional file 2: Table S3).

Moreover, 20 cancer subset outcomes, corresponding to 280 analyses (14 exposures × 20 outcomes), were available (Fig. 4, Additional file 2: Table S3, and Additional file 1: Figs. S42-55). There were five breast cancer outcomes (luminal A-like, luminal B-like, luminal B human epidermal growth factor receptor 2 [HER2] negative-like, HER2 enriched-like, and triple-negative), five lung cancer outcomes (ever-smokers only, never-smokers only, adenocarcinoma, squamous cell carcinoma, and small cell carcinoma), and ten ovarian cancer outcomes, including serous, mucinous, clear cell, and endometrial subtypes. Magnesium was associated with a higher risk of luminal A-like breast cancer (OR = 1.319, 95% CI = 1.159 to 1.501, *P* < 0.0001) but not with the other four breast cancer subtypes, although the minimum OR assuming power of 80% was large for these subtypes (ranging from 1.37 to 1.97). Other associations showing concordance in the overall cancer and subset cancer analyses were magnesium levels associated with a lower risk of invasive ovarian cancer and endometrioid ovarian cancer, and vitamin B12 levels associated with a higher risk of noninvasive, noninvasive serous, and clear cell ovarian cancers (Additional file 2).

## Discussion

In this large-scale MR analysis of 672 micronutrient-cancer associations, we provided the most comprehensive and updated atlas of associations between micronutrients and cancer. We discovered that two associations, the association of magnesium with the risk of breast cancer and the association of vitamin B12 with the risk of colorectal cancer, were robust in terms of statistical significance, sensitivity, and replicability in different cohorts. Cancer subset analysis revealed that magnesium was associated with the luminal A-like breast cancer subtype. No specific micronutrients were beneficial in preventing cancer overall, which is consistent with the findings of previous RCTs [9].

Genetically predicted 1 SD higher levels of magnesium were associated with 1.281 higher odds of breast cancer in the UKB and FinnGen meta-analyses, and 1.235 and 1.319 higher odds of overall breast cancer and luminal A-like breast cancer, respectively, in the BCAC analysis. These findings are consistent with the results of a previous MR study [15] that used an earlier version of the BCAC summary statistics [62], which reported that a 1 SD higher genetically predicted magnesium level was robustly associated with 1.17 higher odds of breast cancer and 1.2 higher odds of estrogen receptor-positive breast cancer, respectively. Although we could not find RCTs or observational studies assessing magnesium levels and breast cancer outcomes, the consistent results and similarity in the effect sizes in different datasets support the validity of our findings. Evidence from in vitro and animal studies has suggested plausible pathways wherein high magnesium concentrations can promote tumor growth and metastasis [63–65].

Genetically predicted 1 SD higher levels of vitamin B12 were associated with 1.22 increased odds of colorectal cancer in the UKB and FinnGen meta-analysis and 1.115 increased odds of colorectal cancer in the analysis using cancer consortium data. This result is consistent with that of a previous MR study that used colorectal cancer GWAS meta-analysis of GECCO and other consortium data [16], which reported 1.12 higher odds per 1 SD increase of genetically predicted vitamin B12 level of colorectal cancer (specifically 1.1 higher odds of colon cancer and 1.21 higher odds of rectal cancer). Previous RCTs reported conflicting results [66, 67]. A long-term follow-up study of RCT of 2524 Caucasian participants compared 2–3 years daily supplementation of folic acid (0.4 mg)/vitamin B12 (0.5 mg) and placebo and reported a higher risk of colorectal cancer in the treatment group (hazard ratio = 1.77, 95% CI = 1.08 to 2.90) [66]. The median age of the participants was 74 years, and the median follow-up period was 78 months, resulting in 68 colorectal cancer cases. Another four-arm RCT assessed 6837 Caucasian patients with ischemic heart disease, with a median age of 63 years. Patients were administered folic acid (0.8 mg), vitamin B12 (0.4 mg), or vitamin B6 (40 mg) [67] for a median of 39 months and were followed for an additional 38 months, resulting in 95 colorectal cancer cases. In this study, the incidence of colorectal cancer did not differ according to the supplements received. One limitation of these trials was their low power. The latter trial [67] reported 629 cases of any cancer and the power to detect any cancer incidence difference between the two groups was 61% [67]. It can be expected that the power to detect differences in colorectal cancer incidence was much lower, given that there were only 95 colorectal cancer cases. Additionally, both trials assigned both vitamin B12 and folic acid to treatment arms, and the participants either had elevated homocysteine level [66] or ischemic heart disease [67], making it hard to generalize the results to the effect of vitamin B12 on the general population. Another key limitation of these RCTs was that the follow-up duration was insufficient to detect an effect on the incidence of colorectal cancer (77–78 months), given that the progression of colorectal adenoma to colorectal cancer may take 10–15 years [68]. A long-term study over 10 years may be beneficial for identifying the true association between vitamin B12 supplementation and colorectal cancer.

For breast, colorectal, lung, ovarian, and prostate cancers, the associations between micronutrient levels were assessed in both the combined UKB and FinnGen cohorts, as well as the cancer consortia cohort. However, there were considerable inconsistencies between the two results. For example, in the UKB plus FinnGen cohort, vitamin C was found to be associated with a decreased risk of colorectal cancer (OR = 0.822, 95% CI = 0.698 to 0.968, *P* = 0.0187) (Fig. 3), which aligns with the results of a previous MR study [69]. However, this association was not replicated in the cancer consortia analysis in our study. Similarly, vitamin B12 was associated with an increased risk of non-invasive ovarian cancer in the cancer consortia dataset analyses (OR = 1.348, 95% CI = 1.106 to 1.643, *P* = 0.0031) and was statistically significant after Bonferroni’s correction for the number of exposures (*P* < 0.0036) (Fig. 4). This was consistent with a previous MR study [22], but the result was not replicated in the UKB plus FinnGen meta-analysis dataset in our study. Only one association, that of iron and colorectal cancer, was statistically significant in both analyses. Nevertheless, this association showed a borderline p-value (0.01 < *P* < 0.05) in both results and a sensitivity analysis was not feasible because it was supported by only two SNPs. This discrepancy indicates that the seemingly robust associations reported in previous MRs need to be carefully assessed to confirm their true causality.

Associations detected on MR may raise awareness regarding the potential harm of micronutrient supplementation, which should be considered when conducting RCTs. For example, the present study and another MR [15] identified high serum magnesium levels as a risk factor for breast cancer; clinicians conducting RCTs with magnesium supplementation should be cautious about breast cancer as a potentially negative outcome and carefully consider whether to include individuals with a high risk of developing breast cancer. Additionally, results from robustly conducted MR may serve as secondary evidence in clinical decision-making before sufficiently powered RCTs are fully conducted. Results from our MR and previous MR studies [15, 16] may imply that excessive intake of magnesium or vitamin B12 (via diet or supplements) can potentially be harmful, especially for individuals without nutritional deficiency and who have a high risk of developing breast cancer or colorectal cancer.

The strengths of our study include its extensive scope, examining over 600 potential causal associations in a consistent manner and analyzing cancer outcome data from the UKB, FinnGen, and various cancer consortia. Additionally, the MR design is inherently less likely to be biased compared to those in classical observational studies, and our MR analysis also reflected the effect of lifelong exposure to micronutrients, thereby assessing long-term risks that may not be moderated by relatively short-term interventions [70].

Nevertheless, this study has several limitations. First, the number of SNPs was small, ranging from 1 to 9, and some exposures had less than four genome-wide significant SNPs; thus, tests for potential pleiotropy could not be performed. Second, although we used the largest available micronutrient and cancer GWASs available to our knowledge to report the most powered associations, not all 308 associations using the UKB plus FinnGen data were sufficiently powered, partly because of our stringent IV selection method. Null associations with low power should be interpreted cautiously to avoid false negative results. Third, because full summary statistics data were not available for most exposures, bidirectional MR analysis was not possible, and some exposure GWASs of micronutrients were adjusted for cancer risk factors (i.e., mediators) and cancer status, potentially leading to collider bias [71]. For example, the GWASs of vitamins A1 and E were adjusted for BMI, cholesterol level, and cancer status (Additional file 2: Table S1). Fourth, we could not assess micronutrients, such as vitamin K, with no appropriate GWAS for MR analyses that may affect cancer outcomes [72]. Fifth, participants in the UKB and FinnGen are likely to represent well-nourished populations without nutritional deficiency, and the observed associations may differ in populations with nutritional deficiency [8], which were not assessed here. Associations may also vary according to sex [9], and sex-stratified MR analyses were not available because of the absence of GWAS results for exposure. Sixth, we meta-analyzed the UKB and FinnGen cohorts; however, the ancestry of the UKB and FinnGen cohorts may have been slightly different, and participants in the FinnGen cohort had fewer close genetic relatives than those in the UKB cohort [73], potentially leading to heterogeneity in the association effect between the cohorts. Additionally, we restricted the study sample to individuals of European ancestry to minimize the population stratification bias. This, in turn, prevents our findings from generalizing to other ancestries. Finally, two-sample MR assumes that the relationship between exposure and outcome is linear; thus, we might not have detected true nonlinear relationships between micronutrients and cancer.

## Conclusions

We performed extensive exposure- and outcome-wide MR analyses to determine the associations between 14 major micronutrients and 22 cancer outcomes. While our study did not show a causal association between micronutrients and overall cancer outcomes, we identified two robust micronutrient-cancer associations, which merit further investigation in clinical trials. Our results may aid clinicians in deciding whether to regulate the intake of certain micronutrients, particularly in high-risk groups without nutritional deficiencies and may help in designing future clinical trials.

### Supplementary Information

**Additional file 1:** **Supplementary Checklist**. Strengthening the reporting of observational epidemiological studies using the Mendelian randomization (STROBE-MR) Checklist. Exposure GWAS search strategy. Modified PRISMA flow chart. Supplementary Methods. **Supplementary Figure 1**. Genetic association of calcium with cancer outcomes. **Supplementary Figure 2.** Genetic association of copper with cancer outcomes. **Supplementary Figure 3.** Genetic association of iron with cancer outcomes. **Supplementary Figure 4.** Genetic association of magnesium with cancer outcomes. **Supplementary Figure 5.** Genetic association of phosphorus with cancer outcomes. **Supplementary Figure 6.** Genetic association of selenium with cancer outcomes. **Supplementary Figure 7.** Genetic association of zinc with cancer outcomes. **Supplementary Figure 8.** Genetic association of vitamin A1 (retinol) with cancer outcomes. **Supplementary Figure 9.** Genetic association of vitamin B6 with cancer outcomes. **Supplementary Figure 10.** Genetic association of vitamin B9 (folate) with cancer outcomes. **Supplementary Figure 11.** Genetic association of vitamin B12 with cancer outcomes. **Supplementary Figure 12.** Genetic association of vitamin C with cancer outcomes. **Supplementary Figure 13.** Genetic association of vitamin D (25-hydroxyvitamin D) with cancer outcomes. **Supplementary Figure 14.** Genetic association of vitamin E with cancer outcomes. **Supplementary Figure 15.** Genetic association of magnesium with breast cancer. **Supplementary Figure 16.** Genetic association of vitamin B12 with colorectal cancer. **Supplementary Figure 17.** Genetic association of magnesium with lung cancer. **Supplementary Figure 18.** Genetic association of selenium with liver cancer. **Supplementary Figure 19.** Genetic association of selenium with breast cancer. **Supplementary Figure 20.** Genetic association of iron with kidney cancer. **Supplementary Figure 21.** Genetic association of vitamin A1 (retinol) with cervical cancer. **Supplementary Figure 22.** Genetic association of iron with colorectal cancer. **Supplementary Figure 23.** Genetic association of phosphorus with uterine cancer. **Supplementary Figure 24.** Genetic association of vitamin C with colorectal cancer. **Supplementary Figure 25.** Genetic association of phosphorus with ovarian cancer. **Supplementary Figure 26.** Genetic association of vitamin C with liver cancer. **Supplementary Figure 27.** Genetic association of phosphorus with breast cancer. **Supplementary Figure 28.** Genetic association of vitamin B9 (folate) with cervical cancer. **Supplementary Figure 29.** Genetic association of vitamin A1 (retinol) with liver cancer. **Supplementary Figure 30.** Genetic association of vitamin E with uterine cancer. **Supplementary Figure 31.** Genetic association of vitamin A1 (retinol) with brain cancer. **Supplementary Figure 32.** Genetic association of magnesium with breast cancer, overall. **Supplementary Figure 33.** Genetic association of vitamin B12 with ovarian cancer, non-invasive. **Supplementary Figure 34.** Genetic association of zinc with colorectal cancer. **Supplementary Figure 35.** Genetic association of vitamin B12 with prostate cancer. **Supplementary Figure 36.** Genetic association of magnesium with ovarian cancer, invasive. **Supplementary Figure 37.** Genetic association of vitamin B12 with colorectal cancer. **Supplementary Figure 38.** Genetic association of selenium with colorectal cancer. **Supplementary Figure 39.** Genetic association of iron with colorectal cancer. **Supplementary Figure 40.** Genetic association of zinc with prostate cancer. **Supplementary Figure 41.** Genetic association of phosphorus with lung cancer, overall cancer type. **Supplementary Figure 42.** Genetic association of magnesium with breast cancer, luminal A-like. **Supplementary Figure 43.** Genetic association of magnesium with ovarian cancer, endometrioid. **Supplementary Figure 44.** Genetic association of phosphorus with breast cancer, HER2 enriched-like. **Supplementary Figure 45.** Genetic association of vitamin E with ovarian cancer, non-invasive serous. **Supplementary Figure 46.** Genetic association of vitamin B12 with lung cancer, adenocarcinoma. **Supplementary Figure 47.** Genetic association of copper with lung cancer, ever smoker. **Supplementary Figure 48.** Genetic association of vitamin C with breast cancer, HER2 enriched-like. **Supplementary Figure 49.** Genetic association of vitamin B12 with ovarian cancer, non-invasive serous. **Supplementary Figure 50.** Genetic association of copper with lung cancer, small cell carcinoma. **Supplementary Figure 51.** Genetic association of calcium with breast cancer, triple-negative. **Supplementary Figure 52.** Genetic association of zinc with ovarian cancer, invasive mucinous. **Supplementary Figure 53.** Genetic association of vitamin B12 with ovarian cancer, clear cell. **Supplementary Figure 54.** Genetic association of vitamin B9 (folate) with lung cancer, ever smoker. **Supplementary Figure 55.** Genetic association of vitamin D (25-hydroxyvitamin D) with lung cancer, small cell carcinoma. 

**Additional file 2:** **Table S1. **Characteristics of genome-wide association studies of exposures.** Table S2. **Details of instrumental variables used in mendelian randomization analyses. **Table S3. **Details of all mendelian randomization analyses associations. **Table S4. **Effects of instrumental variables on major risk factors of cancer (potential confounders).

## Data Availability

All data used in this study are publicly available, and the source of the data are described in the main text.

## Abbreviations

BCAC: Breast Cancer Association Consortium
CI: Confidence interval
GECCO: Genetics and Epidemiology of Colorectal Cancer Consortium
GWAS: Genome-wide association study
IV: Instrumental variable
IVW: Inverse-variance weighted
LD: Linkage disequilibrium
MR: Mendelian randomization
OR: Odds ratio
OCAC: Ovarian Cancer Association Consortium
PRACTICAL: Prostate Cancer Association Group to Investigate Cancer-Associated Alterations in the Genome
RCT: Randomized controlled trial
SD: Standard deviation
SNP: Single-nucleotide polymorphism
STROBE-MR: Strengthening the Reporting of Observational Studies in Epidemiology using Mendelian Randomization
TRICL-ILCCO: Transdisciplinary Research of Cancer in Lung of the International Lung Cancer Consortium
UKB: UK Biobank

## References

[1] Veronese N, Demurtas J, Pesolillo G, Celotto S, Barnini T, Calusi G (2020). Magnesium and health outcomes: an umbrella review of systematic reviews and meta-analyses of observational and intervention studies. Eur J Nutr.

[2] Chen Z, Huang Y, Cao D, Qiu S, Chen B, Li J (2021). Vitamin C intake and cancers: an umbrella review. Front Nutr.

[3] Liu D, Meng X, Tian Q, Cao W, Fan X, Wu L (2022). Vitamin D and multiple health outcomes: an umbrella review of observational studies, randomized controlled trials, and Mendelian randomization studies. Adv Nutr.

[4] Bo Y, Zhu Y, Tao Y, Li X, Zhai D, Bu Y (2020). Association between folate and health outcomes: an umbrella review of meta-analyses. Front Public Health.

[5] Huang Y, Cao D, Chen Z, Chen B, Li J, Wang R (2023). Iron intake and multiple health outcomes: umbrella review. Crit Rev Food Sci Nutr..

[6] Li J, Cao D, Huang Y, Chen B, Chen Z, Wang R (2022). Zinc intakes and health outcomes: an umbrella review. Front Nutr.

[7] Mocellin S, Briarava M, Pilati P (2017). Vitamin B6 and cancer risk: a field synopsis and meta-analysis. J Natl Cancer Inst.

[8] Rautiainen S, Manson JE, Lichtenstein AH, Sesso HD (2016). Dietary supplements and disease prevention - a global overview. Nat Rev Endocrinol.

[9] Fortmann SP, Burda BU, Senger CA, Lin JS, Whitlock EP (2013). Vitamin and mineral supplements in the primary prevention of cardiovascular disease and cancer: an updated systematic evidence review for the U.S. Preventive Services Task Force. Ann Intern Med..

[10] Manson JE, Cook NR, Lee IM, Christen W, Bassuk SS, Mora S (2019). Vitamin D supplements and prevention of cancer and cardiovascular disease. N Engl J Med.

[11] Smith GD, Ebrahim S (2003). 'Mendelian randomization': can genetic epidemiology contribute to understanding environmental determinants of disease?. Int J Epidemiol.

[12] Theodoratou E, Tzoulaki I, Zgaga L, Ioannidis JP (2014). Vitamin D and multiple health outcomes: umbrella review of systematic reviews and meta-analyses of observational studies and randomised trials. BMJ.

[13] Bouillon R, Manousaki D, Rosen C, Trajanoska K, Rivadeneira F, Richards JB (2022). The health effects of vitamin D supplementation: evidence from human studies. Nat Rev Endocrinol.

[14] Fu Y, Xu F, Jiang L, Miao Z, Liang X, Yang J (2021). Circulating vitamin C concentration and risk of cancers: a Mendelian randomization study. BMC Med.

[15] Papadimitriou N, Dimou N, Gill D, Tzoulaki I, Murphy N, Riboli E (2021). Genetically predicted circulating concentrations of micronutrients and risk of breast cancer: a Mendelian randomization study. Int J Cancer.

[16] Tsilidis KK, Papadimitriou N, Dimou N, Gill D, Lewis SJ, Martin RM (2021). Genetically predicted circulating concentrations of micronutrients and risk of colorectal cancer among individuals of European descent: a Mendelian randomization study. Am J Clin Nutr.

[17] Yuan S, Mason AM, Carter P, Vithayathil M, Kar S, Burgess S (2022). Selenium and cancer risk: Wide-angled Mendelian randomization analysis. Int J Cancer.

[18] Markozannes G, Kanellopoulou A, Dimopoulou O, Kosmidis D, Zhang X, Wang L (2022). Systematic review of Mendelian randomization studies on risk of cancer. BMC Med.

[19] Chen H, Du Z, Zhang Y, Li M, Gao R, Qin L (2022). The association between vitamin C and cancer: a two-sample Mendelian randomization study. Front Genet.

[20] Zhao H, Zhu J, Tse LA, Kinra S, Li Y (2022). Genetically predicted circulating levels of antioxidants and risk of breast and ovarian cancer. Cancer Prev Res (Phila).

[21] Yuan S, Carter P, Vithayathil M, Kar S, Giovannucci E, Mason AM, et al. Iron status and cancer risk in UK Biobank: a two-sample Mendelian randomization study. Nutrients. 2020;12(2):526. 10.3390/nu12020526 PMC7071358 32092884

[22] Guo Y, Lu Y, Jin H (2020). Appraising the role of circulating concentrations of micro-nutrients in epithelial ovarian cancer risk: a Mendelian randomization analysis. Sci Rep.

[23] Skrivankova VW, Richmond RC, Woolf BAR, Yarmolinsky J, Davies NM, Swanson SA (2021). Strengthening the Reporting of Observational Studies in Epidemiology Using Mendelian Randomization: the STROBE-MR statement. JAMA.

[24] Zhang H, Ahearn TU, Lecarpentier J, Barnes D, Beesley J, Qi G (2020). Genome-wide association study identifies 32 novel breast cancer susceptibility loci from overall and subtype-specific analyses. Nat Genet.

[25] Huyghe JR, Bien SA, Harrison TA, Kang HM, Chen S, Schmit SL (2019). Discovery of common and rare genetic risk variants for colorectal cancer. Nat Genet.

[26] McKay JD, Hung RJ, Han Y, Zong X, Carreras-Torres R, Christiani DC (2017). Large-scale association analysis identifies new lung cancer susceptibility loci and heterogeneity in genetic susceptibility across histological subtypes. Nat Genet.

[27] Phelan CM, Kuchenbaecker KB, Tyrer JP, Kar SP, Lawrenson K, Winham SJ (2017). Identification of 12 new susceptibility loci for different histotypes of epithelial ovarian cancer. Nat Genet.

[28] Schumacher FR, Al Olama AA, Berndt SI, Benlloch S, Ahmed M, Saunders EJ (2018). Association analyses of more than 140,000 men identify 63 new prostate cancer susceptibility loci. Nat Genet.

[29] Lawlor DA, Harbord RM, Sterne JA, Timpson N, Davey SG (2008). Mendelian randomization: using genes as instruments for making causal inferences in epidemiology. Stat Med.

[30] Benyamin B, Esko T, Ried JS, Radhakrishnan A, Vermeulen SH, Traglia M (2014). Novel loci affecting iron homeostasis and their effects in individuals at risk for hemochromatosis. Nat Commun.

[31] Cornelis MC, Fornage M, Foy M, Xun P, Gladyshev VN, Morris S (2015). Genome-wide association study of selenium concentrations. Hum Mol Genet.

[32] Evans DM, Zhu G, Dy V, Heath AC, Madden PA, Kemp JP (2013). Genome-wide association study identifies loci affecting blood copper, selenium and zinc. Hum Mol Genet.

[33] Grarup N, Sulem P, Sandholt CH, Thorleifsson G, Ahluwalia TS, Steinthorsdottir V (2013). Genetic architecture of vitamin B12 and folate levels uncovered applying deeply sequenced large datasets. PLoS Genet.

[34] Hazra A, Kraft P, Lazarus R, Chen C, Chanock SJ, Jacques P (2009). Genome-wide significant predictors of metabolites in the one-carbon metabolism pathway. Hum Mol Genet.

[35] Jiang X, O'Reilly PF, Aschard H, Hsu YH, Richards JB, Dupuis J (2018). Genome-wide association study in 79,366 European-ancestry individuals informs the genetic architecture of 25-hydroxyvitamin D levels. Nat Commun.

[36] Kestenbaum B, Glazer NL, Köttgen A, Felix JF, Hwang SJ, Liu Y (2010). Common genetic variants associate with serum phosphorus concentration. J Am Soc Nephrol.

[37] Major JM, Yu K, Wheeler W, Zhang H, Cornelis MC, Wright ME (2011). Genome-wide association study identifies common variants associated with circulating vitamin E levels. Hum Mol Genet.

[38] Meyer TE, Verwoert GC, Hwang SJ, Glazer NL, Smith AV, van Rooij FJ (2010). Genome-wide association studies of serum magnesium, potassium, and sodium concentrations identify six Loci influencing serum magnesium levels. PLoS Genet.

[39] Mondul AM, Yu K, Wheeler W, Zhang H, Weinstein SJ, Major JM (2011). Genome-wide association study of circulating retinol levels. Hum Mol Genet.

[40] O'Seaghdha CM, Wu H, Yang Q, Kapur K, Guessous I, Zuber AM (2013). Meta-analysis of genome-wide association studies identifies six new Loci for serum calcium concentrations. PLoS Genet.

[41] Zheng JS, Luan J, Sofianopoulou E, Imamura F, Stewart ID, Day FR (2021). Plasma Vitamin C and Type 2 Diabetes: Genome-Wide Association Study and Mendelian Randomization Analysis in European Populations. Diabetes Care.

[42] Gill D, Benyamin B, Moore LSP, Monori G, Zhou A, Koskeridis F (2019). Associations of genetically determined iron status across the phenome: a mendelian randomization study. PLoS Med.

[43] Dashti HS, Shea MK, Smith CE, Tanaka T, Hruby A, Richardson K (2014). Meta-analysis of genome-wide association studies for circulating phylloquinone concentrations. Am J Clin Nutr.

[44] Lee Lab. https://www.leelabsg.org/resources. Accessed 1 May 2022. .

[45] GWAS results. http://www.nealelab.is/uk-biobank. Accessed 1 May 2022. .

[46] FinnGen Research Project. https://finngen.gitbook.io/documentation/data-download. Accessed 8 June 2023. .

[47] Willer CJ, Li Y, Abecasis GR (2010). METAL: fast and efficient meta-analysis of genomewide association scans. Bioinformatics.

[48] Bowden J, Del Greco MF, Minelli C, Davey Smith G, Sheehan NA, Thompson JR (2016). Assessing the suitability of summary data for two-sample Mendelian randomization analyses using MR-Egger regression: the role of the I2 statistic. Int J Epidemiol.

[49] Shim H, Chasman DI, Smith JD, Mora S, Ridker PM, Nickerson DA (2015). A multivariate genome-wide association analysis of 10 LDL subfractions, and their response to statin treatment, in 1868 Caucasians. PLoS ONE.

[50] Tang B, Yuan S, Xiong Y, He Q, Larsson SC (2020). Major depressive disorder and cardiometabolic diseases: a bidirectional Mendelian randomisation study. Diabetologia.

[51] Yuan S, Larsson SC (2020). An atlas on risk factors for type 2 diabetes: a wide-angled Mendelian randomisation study. Diabetologia.

[52] Brion MJ, Shakhbazov K, Visscher PM (2013). Calculating statistical power in Mendelian randomization studies. Int J Epidemiol.

[53] Hartwig FP, Davey Smith G, Bowden J (2017). Robust inference in summary data Mendelian randomization via the zero modal pleiotropy assumption. Int J Epidemiol.

[54] Bowden J, Davey Smith G, Haycock PC, Burgess S (2016). Consistent estimation in Mendelian randomization with some invalid instruments using a weighted median estimator. Genet Epidemiol.

[55] Bowden J, Davey Smith G, Burgess S (2015). Mendelian randomization with invalid instruments: effect estimation and bias detection through Egger regression. Int J Epidemiol.

[56] Verbanck M, Chen CY, Neale B, Do R (2018). Detection of widespread horizontal pleiotropy in causal relationships inferred from Mendelian randomization between complex traits and diseases. Nat Genet.

[57] Burgess S, Bowden J, Fall T, Ingelsson E, Thompson SG (2017). Sensitivity analyses for robust causal inference from Mendelian randomization analyses with multiple genetic variants. Epidemiology.

[58] Doherty A, Smith-Byrne K, Ferreira T, Holmes MV, Holmes C, Pulit SL (2018). GWAS identifies 14 loci for device-measured physical activity and sleep duration. Nat Commun.

[59] Liu M, Jiang Y, Wedow R, Li Y, Brazel DM, Chen F (2019). Association studies of up to 1.2 million individuals yield new insights into the genetic etiology of tobacco and alcohol use. Nat Genet..

[60] Shungin D, Winkler TW, Croteau-Chonka DC, Ferreira T, Locke AE, Magi R (2015). New genetic loci link adipose and insulin biology to body fat distribution. Nature.

[61] Yengo L, Sidorenko J, Kemper KE, Zheng Z, Wood AR, Weedon MN (2018). Meta-analysis of genome-wide association studies for height and body mass index in approximately 700000 individuals of European ancestry. Hum Mol Genet.

[62] Michailidou K, Lindström S, Dennis J, Beesley J, Hui S, Kar S (2017). Association analysis identifies 65 new breast cancer risk loci. Nature.

[63] Castiglioni S, Maier JA (2011). Magnesium and cancer: a dangerous liason. Magnes Res.

[64] Lymburner S, McLeod S, Purtzki M, Roskelley C, Xu Z (2013). Zinc inhibits magnesium-dependent migration of human breast cancer MDA-MB-231 cells on fibronectin. J Nutr Biochem.

[65] Mendes PMV, Bezerra DLC, Dos Santos LR, de Oliveira SR, de Sousa Melo SR, Morais JBS (2018). Magnesium in breast cancer: what is its influence on the progression of this disease?. Biol Trace Elem Res.

[66] Oliai Araghi S, Kiefte-de Jong JC, van Dijk SC, Swart KMA, van Laarhoven HW, van Schoor NM (2019). Folic acid and vitamin B12 supplementation and the risk of cancer: long-term follow-up of the B vitamins for the prevention of osteoporotic fractures (B-PROOF) trial. Cancer Epidemiol Biomarkers Prev.

[67] Ebbing M, Bonaa KH, Nygard O, Arnesen E, Ueland PM, Nordrehaug JE (2009). Cancer incidence and mortality after treatment with folic acid and vitamin B12. JAMA.

[68] Papaioannou D, Cooper KL, Carroll C, Hind D, Squires H, Tappenden P (2011). Antioxidants in the chemoprevention of colorectal cancer and colorectal adenomas in the general population: a systematic review and meta-analysis. Colorectal Dis.

[69] Larsson SC, Mason AM, Vithayathil M, Carter P, Kar S, Zheng JS (2022). Circulating vitamin C and digestive system cancers: Mendelian randomization study. Clin Nutr.

[70] Walker VM, Davey Smith G, Davies NM, Martin RM (2017). Mendelian randomization: a novel approach for the prediction of adverse drug events and drug repurposing opportunities. Int J Epidemiol.

[71] Burgess S, Davey Smith G, Davies NM, Dudbridge F, Gill D, Glymour MM (2019). Guidelines for performing Mendelian randomization investigations. Wellcome Open Res.

[72] Nimptsch K, Rohrmann S, Kaaks R, Linseisen J (2010). Dietary vitamin K intake in relation to cancer incidence and mortality: results from the Heidelberg cohort of the European Prospective Investigation into Cancer and Nutrition (EPIC-Heidelberg). Am J Clin Nutr.

[73] Kurki MI, Karjalainen J, Palta P, Sipilä TP, Kristiansson K, Donner KM (2023). FinnGen provides genetic insights from a well-phenotyped isolated population. Nature.

