# Supplementary material for: An atlas of associations between 14 micronutrients and 22 cancer outcomes: Mendelian randomization analyses
Source: BMC Med. 2023 Aug 21;21:316. doi: 10.1186/s12916-023-03018-y (PMC10441703; doi:10.1186/s12916-023-03018-y)
Supplement: Supplementary file 1 — Additional file 1: Supplementary Checklist. Strengthening the reporting of observational epidemiological studies using the Mendelian randomization (STROBE-MR) Checklist. Exposure GWAS search strategy. Modified PRISMA flow chart. Supplementary Methods. Supplementary Figure 1. Genetic association of calcium with cancer outcomes. Supplementary Figure 2. Genetic association of copper with cancer outcomes. Supplementary Figure 3. Genetic association of iron with cancer outcomes. Supplementary Figure 4. Genetic association of magnesium with cancer outcomes. Supplementary Figure 5. Genetic association of phosphorus with cancer outcomes. Supplementary Figure 6. Genetic association of selenium with cancer outcomes. Supplementary Figure 7. Genetic association of zinc with cancer outcomes. Supplementary Figure 8. Genetic association of vitamin A1 (retinol) with cancer outcomes. Supplementary Figure 9. Genetic association of vitamin B6 with cancer outcomes. Supplementary Figure 10. Genetic association of vitamin B9 (folate) with cancer outcomes. Supplementary Figure 11. Genetic association of vitamin B12 with cancer outcomes. Supplementary Figure 12. Genetic association of vitamin C with cancer outcomes. Supplementary Figure 13. Genetic association of vitamin D (25-hydroxyvitamin D) with cancer outcomes. Supplementary Figure 14. Genetic association of vitamin E with cancer outcomes. Supplementary Figure 15. Genetic association of magnesium with breast cancer. Supplementary Figure 16. Genetic association of vitamin B12 with colorectal cancer. Supplementary Figure 17. Genetic association of magnesium with lung cancer. Supplementary Figure 18. Genetic association of selenium with liver cancer. Supplementary Figure 19. Genetic association of selenium with breast cancer. Supplementary Figure 20. Genetic association of iron with kidney cancer. Supplementary Figure 21. Genetic association of vitamin A1 (retinol) with cervical cancer. Supplementary Figure 22. Genetic association of iro [file 12916_2023_3018_MOESM1_ESM.docx]

**Supplementary material**

**An atlas of association between 14 micronutrients and 22 cancer outcomes: Mendelian randomization analyses**

Contents

[Supplementary Checklist. Strengthening the Reporting of Observational Studies in Epidemiology using Mendelian Randomization (STROBE-MR) checklist 4](#_Toc139684091)

[Exposure GWAS search strategy 9](#_Toc139684092)

[Modified PRISMA flow chart 9](#_Toc139684093)

[Supplementary Methods 10](#_Toc139684094)

[Supplementary Figure 1. Genetic association of calcium with cancer outcomes 11](#_Toc139684095)

[Supplementary Figure 2. Genetic association of copper with cancer outcomes 12](#_Toc139684096)

[Supplementary Figure 3. Genetic association of iron with cancer outcomes 13](#_Toc139684097)

[Supplementary Figure 4. Genetic association of magnesium with cancer outcomes 14](#_Toc139684098)

[Supplementary Figure 5. Genetic association of phosphorus with cancer outcomes 15](#_Toc139684099)

[Supplementary Figure 6. Genetic association of selenium with cancer outcomes 16](#_Toc139684100)

[Supplementary Figure 7. Genetic association of zinc with cancer outcomes 17](#_Toc139684101)

[Supplementary Figure 8. Genetic association of vitamin A1 (retinol) with cancer outcomes 18](#_Toc139684102)

[Supplementary Figure 9. Genetic association of vitamin B6 with cancer outcomes 19](#_Toc139684103)

[Supplementary Figure 10. Genetic association of vitamin B9 (folate) with cancer outcomes 20](#_Toc139684104)

[Supplementary Figure 11. Genetic association of vitamin B12 with cancer outcomes 21](#_Toc139684105)

[Supplementary Figure 12. Genetic association of vitamin C with cancer outcomes 22](#_Toc139684106)

[Supplementary Figure 13. Genetic association of vitamin D (25-hydroxyvitamin D) with cancer outcomes 23](#_Toc139684107)

[Supplementary Figure 14. Genetic association of vitamin E with cancer outcomes 24](#_Toc139684108)

[Supplementary Figure 15. Genetic association of magnesium with breast cancer 25](#_Toc139684109)

[Supplementary Figure 16. Genetic association of vitamin B12 with colorectal cancer 27](#_Toc139684110)

[Supplementary Figure 17. Genetic association of magnesium with lung cancer 29](#_Toc139684111)

[Supplementary Figure 18. Genetic association of selenium with liver cancer 31](#_Toc139684112)

[Supplementary Figure 19. Genetic association of selenium with breast cancer 33](#_Toc139684113)

[Supplementary Figure 20. Genetic association of iron with kidney cancer 35](#_Toc139684114)

[Supplementary Figure 21. Genetic association of vitamin A1 (retinol) with cervical cancer 37](#_Toc139684115)

[Supplementary Figure 22. Genetic association of iron with colorectal cancer 39](#_Toc139684116)

[Supplementary Figure 23. Genetic association of phosphorus with uterine cancer 41](#_Toc139684117)

[Supplementary Figure 24. Genetic association of vitamin C with colorectal cancer 43](#_Toc139684118)

[Supplementary Figure 25. Genetic association of phosphorus with ovarian cancer 45](#_Toc139684119)

[Supplementary Figure 26. Genetic association of vitamin C with liver cancer 47](#_Toc139684120)

[Supplementary Figure 27. Genetic association of phosphorus with breast cancer 49](#_Toc139684121)

[Supplementary Figure 28. Genetic association of vitamin B9 (folate) with cervical cancer 51](#_Toc139684122)

[Supplementary Figure 29. Genetic association of vitamin A1 (retinol) with liver cancer 53](#_Toc139684123)

[Supplementary Figure 30. Genetic association of vitamin E with uterine cancer 55](#_Toc139684124)

[Supplementary Figure 31. Genetic association of vitamin A1 (retinol) with brain cancer 57](#_Toc139684125)

[Supplementary Figure 32. Genetic association of magnesium with breast cancer, overall 59](#_Toc139684126)

[Supplementary Figure 33. Genetic association of vitamin B12 with ovarian cancer, non-invasive 61](#_Toc139684127)

[Supplementary Figure 34. Genetic association of zinc with colorectal cancer 63](#_Toc139684128)

[Supplementary Figure 35. Genetic association of vitamin B12 with prostate cancer 65](#_Toc139684129)

[Supplementary Figure 36. Genetic association of magnesium with ovarian cancer, invasive 67](#_Toc139684130)

[Supplementary Figure 37. Genetic association of vitamin B12 with colorectal cancer 69](#_Toc139684131)

[Supplementary Figure 38. Genetic association of selenium with colorectal cancer 71](#_Toc139684132)

[Supplementary Figure 39. Genetic association of iron with colorectal cancer 73](#_Toc139684133)

[Supplementary Figure 40. Genetic association of zinc with prostate cancer 75](#_Toc139684134)

[Supplementary Figure 41. Genetic association of phosphorus with lung cancer, overall cancer type 77](#_Toc139684135)

[Supplementary Figure 42. Genetic association of magnesium with breast cancer, luminal A-like 79](#_Toc139684136)

[Supplementary Figure 43. Genetic association of magnesium with ovarian cancer, endometrioid 81](#_Toc139684137)

[Supplementary Figure 44. Genetic association of phosphorus with breast cancer, HER2 enriched-like 83](#_Toc139684138)

[Supplementary Figure 45. Genetic association of vitamin E with ovarian cancer, non-invasive serous 85](#_Toc139684139)

[Supplementary Figure 46. Genetic association of vitamin B12 with lung cancer, adenocarcinoma 87](#_Toc139684140)

[Supplementary Figure 47. Genetic association of copper with lung cancer, ever smoker 89](#_Toc139684141)

[Supplementary Figure 48. Genetic association of vitamin C with breast cancer, HER2 enriched-like 91](#_Toc139684142)

[Supplementary Figure 49. Genetic association of vitamin B12 with ovarian cancer, non-invasive serous 93](#_Toc139684143)

[Supplementary Figure 50. Genetic association of copper with lung cancer, small cell carcinoma 95](#_Toc139684144)

[Supplementary Figure 51. Genetic association of calcium with breast cancer, triple-negative 97](#_Toc139684145)

[Supplementary Figure 52. Genetic association of zinc with ovarian cancer, invasive mucinous 99](#_Toc139684146)

[Supplementary Figure 53. Genetic association of vitamin B12 with ovarian cancer, clear cell 101](#_Toc139684147)

[Supplementary Figure 54. Genetic association of vitamin B9 (folate) with lung cancer, ever smoker 103](#_Toc139684148)

[Supplementary Figure 55. Genetic association of vitamin D (25-hydroxyvitamin D) with lung cancer, small cell carcinoma 105](#_Toc139684149)

# Supplementary Checklist. Strengthening the Reporting of Observational Studies in Epidemiology using Mendelian Randomization (STROBE-MR) checklist

| Item | Complete/location |
| --- | --- |
| 1. Title and Abstract  Indicate Mendelian randomization (MR) as the study’s design in the title and/or the abstract if that is a main purpose of the study | Title and abstract |
| Introduction |  |
| 2.Background  Explain the scientific background and rationale for the reported study. What is the exposure? Is a potential causal relationship between exposure and outcome plausible? Justify why MR is a helpful method to address the study question | Introduction, Paragraphs 1–2 |
| 3.Objectives  State specific objectives clearly, including pre-specified causal hypotheses (if any). State that MR is a method that, under specific assumptions, intends to estimate causal effects | Introduction, Paragraphs 1–2 |
| 4. Study design and data sources  Present key elements of the study design early in the article. Consider including a table listing sources of data for all phases of the study. For each data source contributing to the analysis, describe the following: | Introduction, Paragraph 1–2  a-d) Information about the GWAS data is described in Methods paragraphs 1–4 (sections: *Study design, Genetic associations with micronutrients, and Genetic associations with cancer*) and listed in Supplementary Table 1. Further information is provided in each of the original GWAS publications, and their reference listed in the manuscript. Selection of genetic variants is described in Methods paragraph 5 (section *Selection of genetic instruments*), and the list of variants is provided in Supplementary Table 2.  e) not relevant |
| a) Setting: Describe the study design and the underlying population, if possible. Describe the setting, locations, and relevant dates, including periods of recruitment, exposure, follow-up, and data collection, when available. |  |
| b) Participants: Give the eligibility criteria, and the sources and methods of selection of participants. Report the sample size, and whether any power or sample size calculations were carried out prior to the main analysis |  |
| c) Describe measurement, quality control and selection of genetic variants |  |
| d) For each exposure, outcome, and other relevant variables, describe methods of assessment and diagnostic criteria for diseases |  |
| e) Provide details of ethics committee approval and participant informed consent, if relevant |  |
| 5. Assumptions  Explicitly state the three core IV assumptions for the main analysis (relevance, independence and exclusion restriction) as well assumptions for any additional or sensitivity analysis | Methods paragraph 1 (Section *Study Design*) |
| 6. **Statistical methods main analysis** Describe statistical methods and statistics use | a, b, c, e) Methods paragraph 6 (section *Statistical analysis*)  d) not applicable |
| a) Describe how quantitative variables were handled in the analyses (i.e., scale, units, model) |  |
| b) Describe how genetic variants were handled in the analyses and, if applicable, how their weights were selected |  |
| c) Describe the MR estimator (e.g. two-stage least squares, Wald ratio) and related statistics. Detail the included covariates and, in case of two-sample MR, whether the same covariate set was used for adjustment in the two samples |  |
| d) Explain how missing data were addressed |  |
| e) If applicable, indicate how multiple testing was addressed |  |
| 7. Assessment of assumptions  Describe any methods or prior knowledge used to assess the assumptions or justify their validity | Methods paragraph 6 (section *Statistical analysis*) |
| 8.Sensitivity analyses  Describe any sensitivity analyses or additional analyses performed (e.g. comparison of effect estimates from different approaches, independent replication, bias analytic techniques, validation of instruments, simulations) | Methods paragraph 6 (section *Statistical analysis*) |
| 9. **Software and pre-registration** a) Name statistical software and package(s), including version and settings used | a) Methods paragraph 6 (section *Statistical analysis*) b) Not applicable |
| b) State whether the study protocol and details were pre-registered (as well as when and where) |  |
| Results |  |
| 10. **Descriptive data** a) Report the numbers of individuals at each stage of included studies and reasons for exclusion. Consider use of a flow diagram | a) Supplementary Table S1  b) Supplementary Table S2  c) Not applicable  d) Methods paragraph 1 (Section *Study Design*) |
| b) Report summary statistics for phenotypic exposure(s), outcome(s), and other relevant variables (e.g. means, SDs, proportions) |  |
| c) If the data sources include meta-analyses of previous studies, provide the assessments of heterogeneity across these studies |  |
| d) For two-sample MR:  i. Provide justification of the similarity of the genetic variant-exposure associations between the exposure and outcome samples ii. Provide information on the number of individuals who overlap between the exposure and outcome studies |  |
| 11. **Main results** a) Report the associations between genetic variant and exposure, and between genetic variant and outcome, preferably on an interpretable scale | a) Supplementary Table 2  b) Results paragraph 2-5, Table 1-2, Supplementary Table S3  c) Not applicable  d) Figure 1, Supplementary Figure 1-51 |
| b) Report MR estimates of the relationship between exposure and outcome, and the measures of uncertainty from the MR analysis, on an interpretable scale, such as odds ratio or relative risk per SD difference |  |
| c) If relevant, consider translating estimates of relative risk into absolute risk for a meaningful time period |  |
| d) Consider plots to visualize results (e.g. forest plot, scatterplot of associations between genetic variants and outcome versus between genetic variants and exposure) |  |
| 12. **Assessment of assumptions** a) Report the assessment of the validity of the assumptions | a, b) Results paragraph 1-5, Table 1-2 |
| b) Report any additional statistics (e.g., assessments of heterogeneity across genetic variants, such as I2, Q statistic or E-value) |  |
| 13. **Sensitivity and additional analyses** a) Report any sensitivity analyses to assess the robustness of the main results to violations of the assumptions | a, b) Results paragraph 1-5, Table 1-2  c, d) not applicable  e) Supplementary Figure 1-51 |
| b) Report results from other sensitivity analyses or additional analyses |  |
| c) Report any assessment of direction of causal relationship (e.g., bidirectional MR) |  |
| d) When relevant, report and compare with estimates from non-MR analyses |  |
| e) Consider additional plots to visualize results (e.g., leave-one-out analyses) |  |
| Discussion |  |
| 14. Key results  Summarize key results with reference to study objectives | Discussion paragraph 1 |
| 15. **Limitations** Discuss limitations of the study, taking into account the validity of the IV assumptions, other sources of potential bias, and imprecision. Discuss both direction and magnitude of any potential bias and any efforts to address them | Discussion paragraph 7 |
| 16. **Interpretations** a) Meaning: Give a cautious overall interpretation of results in the context of their limitations and in comparison with other studies | a, b, c) Discussion paragraph 1–6 |
| b) Mechanism: Discuss underlying biological mechanisms that could drive a potential causal relationship between the investigated exposure and the outcome, and whether the gene-environment equivalence assumption is reasonable. Use causal language carefully, clarifying that IV estimates may provide causal effects only under certain assumptions |  |
| c) Clinical relevance: Discuss whether the results have clinical or public policy relevance, and to what extent they inform effect sizes of possible interventions |  |
| 17. Generalizability  Discuss the generalizability of the study results (a) to other populations, (b) across other exposure periods/timings, and (c) across other levels of exposure | a, b, c) Discussion paragraph 1–6 |
| 18.  Funding  Describe sources of funding and the role of funders in the present study and, if applicable, sources of funding for the databases and original study or studies on which the present study is based | Funding |
| 19. Data and data sharing  Provide the data used to perform all analyses or report where and how the data can be accessed, and reference these sources in the article. Provide the statistical code needed to reproduce the results in the article, or report whether the code is publicly accessible and if so, where | Data availability statement |
| 20. Conflicts of Interest  All authors should declare all potential conflicts of interest | Competing interest statement |

Skrivankova VW, Richmond RC, Woolf BAR, et al. Strengthening the reporting of observational studies in epidemiology using mendelian randomisation (STROBE-MR): explanation and elaboration. Bmj. 2021;375:n2233

# Exposure GWAS search strategy

Micronutrients were defined as vitamins and minerals needed by the body in very small amounts.
WHO. Health topics: Micronutrients. <https://www.who.int/health-topics/micronutrients#tab=tab_1>

We utilized the examples of micronutrients provided by NIH for constructing search terms.
NIH. Vitamins and minerals. <https://www.nccih.nih.gov/health/vitamins-and-minerals>

Search term for PubMed: (vitamin*[tiab] OR micronutrient*[tiab] OR "Vitamins"[Mesh] OR "Micronutrients"[Mesh] OR thiamin*[ti] OR riboflavin*[ti] OR niacin*[ti] OR pantothenic[ti] OR pyridoxine*[ti] OR pyridoxal*[ti] OR cobalamin*[ti] OR biotin*[ti] OR folate*[ti] OR folic[ti] OR calcium*[ti] OR phosphorus*[ti] OR potassium*[ti] OR sodium*[ti] OR chloride*[ti] OR magnesium*[ti] OR iron*[ti] OR zinc*[ti] OR iodine*[ti] OR sulfur*[ti] OR cobalt*[ti] OR copper*[ti] OR fluoride*[ti] OR manganese*[ti] OR selenium*[ti])

Search date: July 01, 2022

Hits: 1041

# Modified PRISMA flow chart

**Identification of studies via databases and registers**

Records removed *before screening*:

Duplicate records removed (n = 0)

Records marked as ineligible by automation tools (n = 0)

Records removed for other reasons (n = 0)

Records identified from:

PubMed (n = 1041)

**Identification**

Records screened

(n = 1041)

Records excluded

(n = 959)

Reports sought for retrieval

(n = 82)

Reports not retrieved

(n = 0)

**Screening**

Reports excluded: 71

Not genome-wide study of micronutrient (n = 50)

Irrelevant study (n=18)

Genome-wide significance not reached (n = 2)

Larger GWAS available (n = 1)

Reports assessed for eligibility

(n = 82)

Studies included in review

(n = 12)

Micronutrients reported

(n = 14)

Reference screening of relevant articles

(n = 1)

**Included**

*From:*  Page MJ, McKenzie JE, Bossuyt PM, Boutron I, Hoffmann TC, Mulrow CD, et al. The PRISMA 2020 statement: an updated guideline for reporting systematic reviews. BMJ 2021;372:n71. doi: 10.1136/bmj.n71. For more information, visit: <http://www.prisma-statement.org/>

# Supplementary Methods

Instrumental strength for the SNP–exposure association was calculated as the average of SNP-specific F-statistics. SNP-specific F-statistics were approximated by the square of the beta divided by the variance for the SNP–exposure association, as follows:

$$\boldsymbol{F=}\frac{\boldsymbol{\beta}\boldsymbol{2}}{\boldsymbol{se}\left( \boldsymbol{\beta} \right)^{\boldsymbol{2}}}$$

where β is the effect of SNP and se(β)^2^ is the variance of β.^1^

Variance explained by the SNP–exposure association was calculated as the sum of variances explained by each SNP, which were each approximated as follows:

$$\frac{\boldsymbol{2}\boldsymbol{\beta^{2}MAF(1-MAF)}}{\boldsymbol{2}\boldsymbol{\beta}^{\boldsymbol{2}}\boldsymbol{MAF}\left( \boldsymbol{1-MAF} \right)\boldsymbol{+(se}\left( \boldsymbol{\beta} \right)^{\boldsymbol{2}}\boldsymbol{)2}\boldsymbol{NMAF(1-MAF)}}$$

where β is the effect of the SNP, MAF the allele frequency, se(β)^2^ the variance of β, and N being the number of participants the SNP is based on.^2^

**References for Supplementary Methods**

1. Bowden J, Del Greco MF, Minelli C, Davey Smith G, Sheehan NA, Thompson JR. Assessing the suitability of summary data for two-sample Mendelian randomization analyses using MR-Egger regression: the role of the I2 statistic. *Int J Epidemiol* 2016;45:1961-74.

2. Shim H, Chasman DI, Smith JD, et al. A multivariate genome-wide association analysis of 10 LDL subfractions, and their response to statin treatment, in 1868 Caucasians. *PLoS One* 2015;10:e0120758.

# Supplementary Figure 1. Genetic association of calcium with cancer outcomes


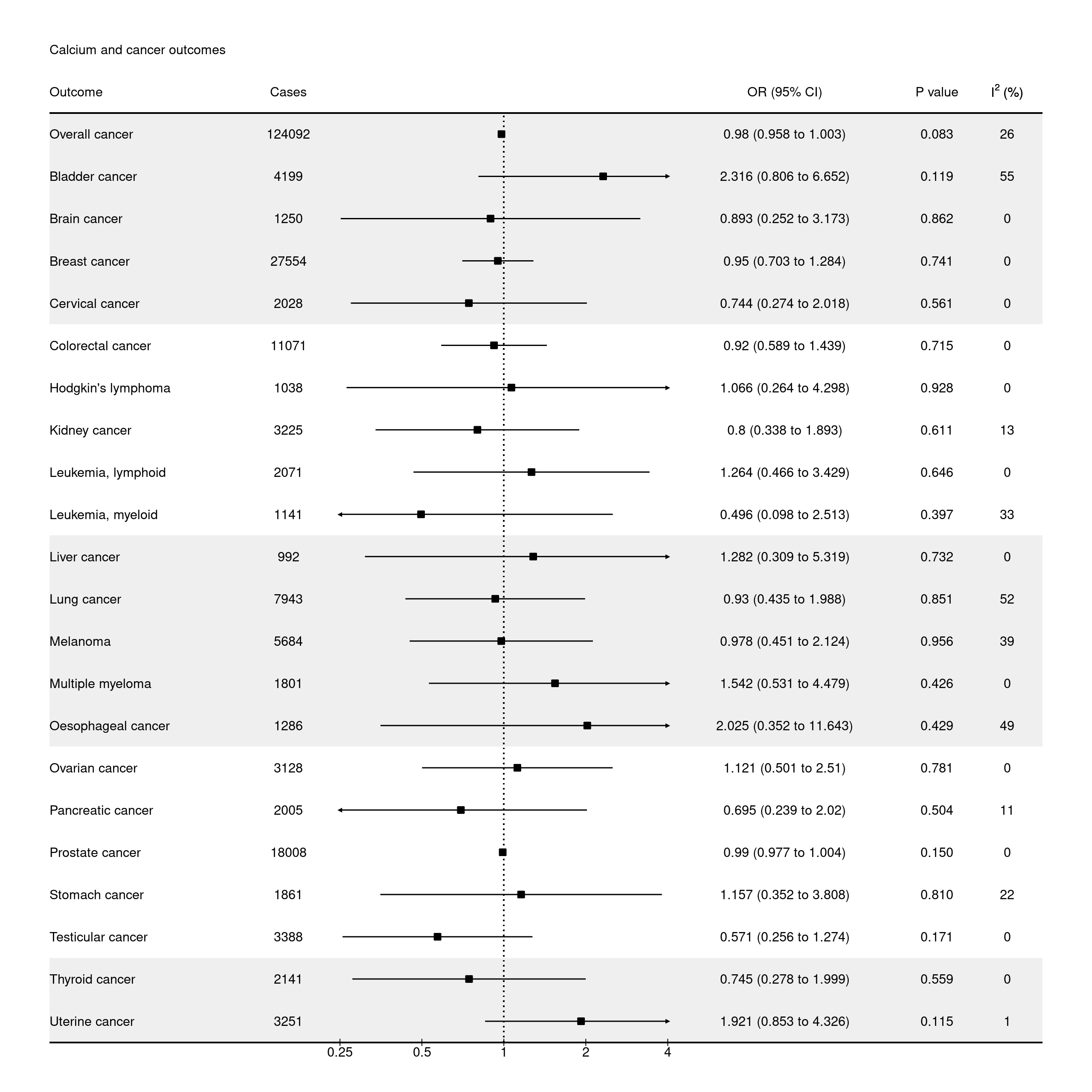


# Supplementary Figure 2. Genetic association of copper with cancer outcomes


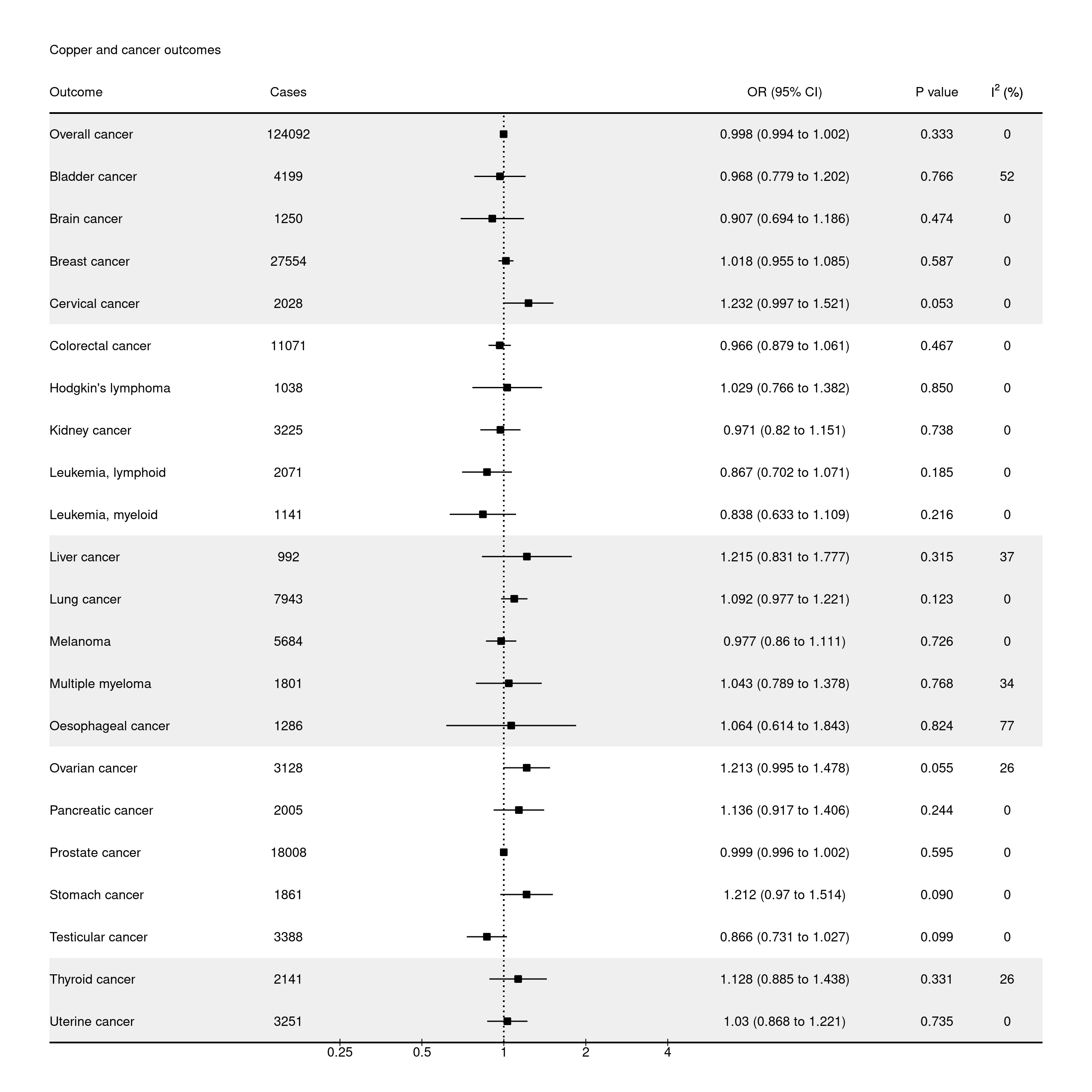


# Supplementary Figure 3. Genetic association of iron with cancer outcomes


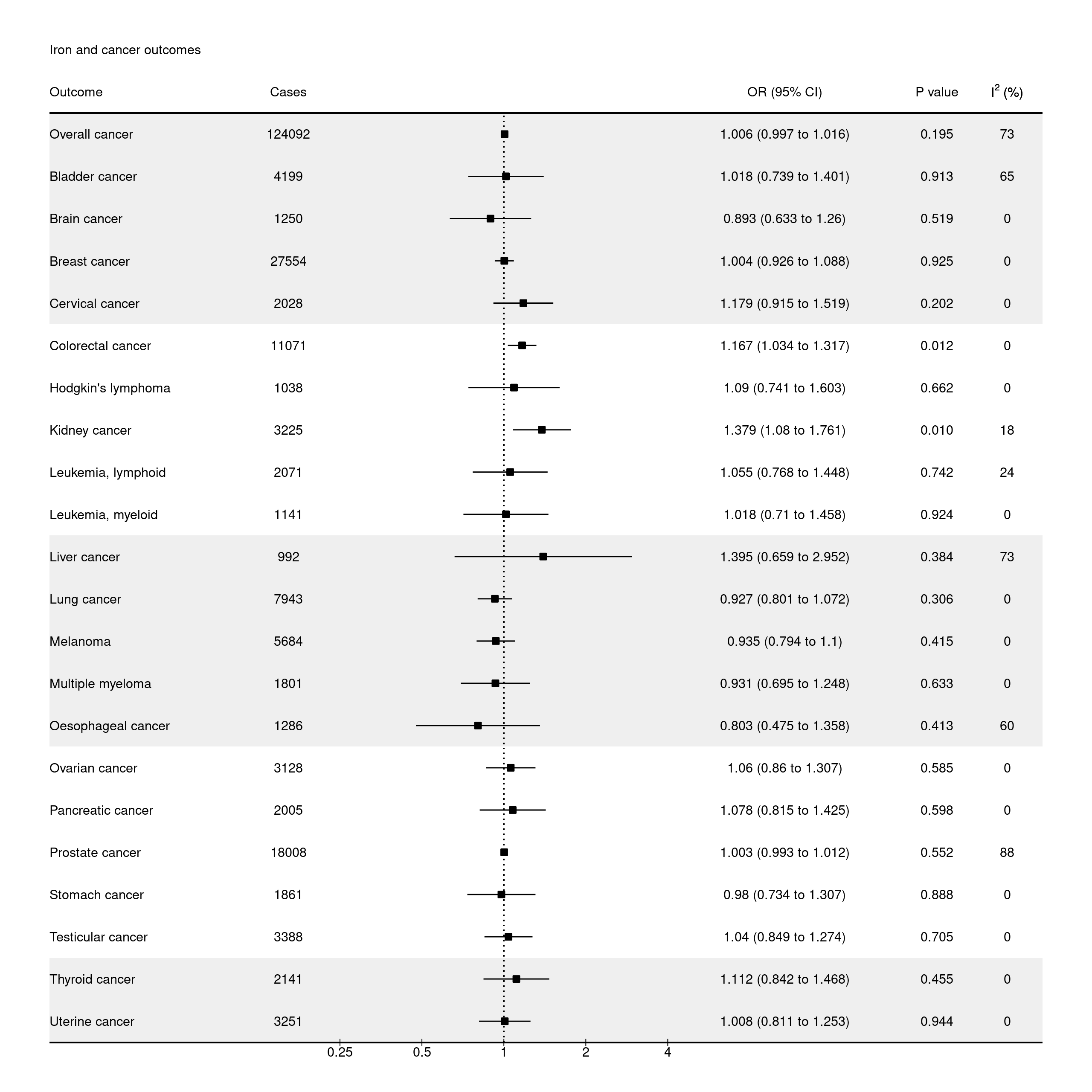


# Supplementary Figure 4. Genetic association of magnesium with cancer outcomes


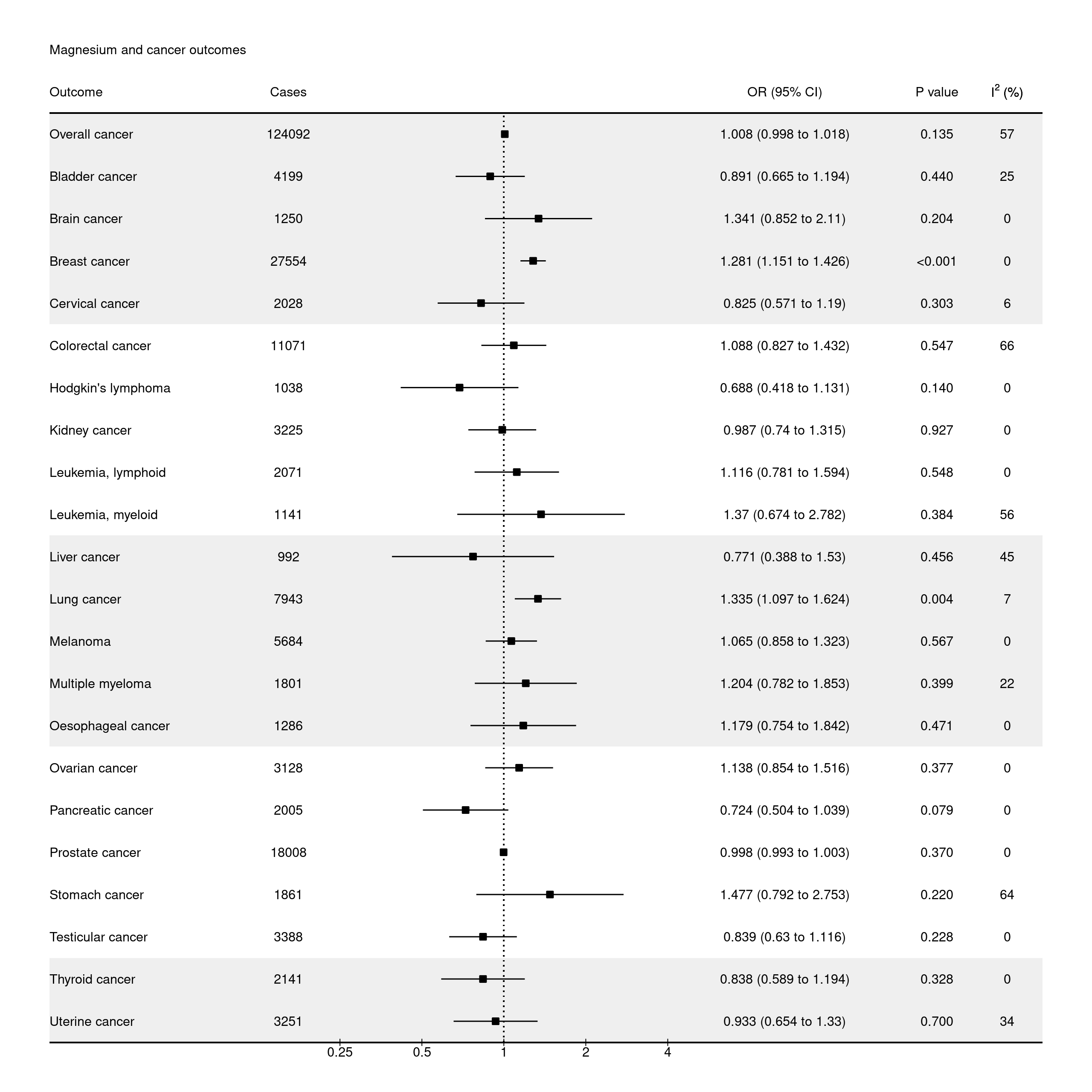


# Supplementary Figure 5. Genetic association of phosphorus with cancer outcomes


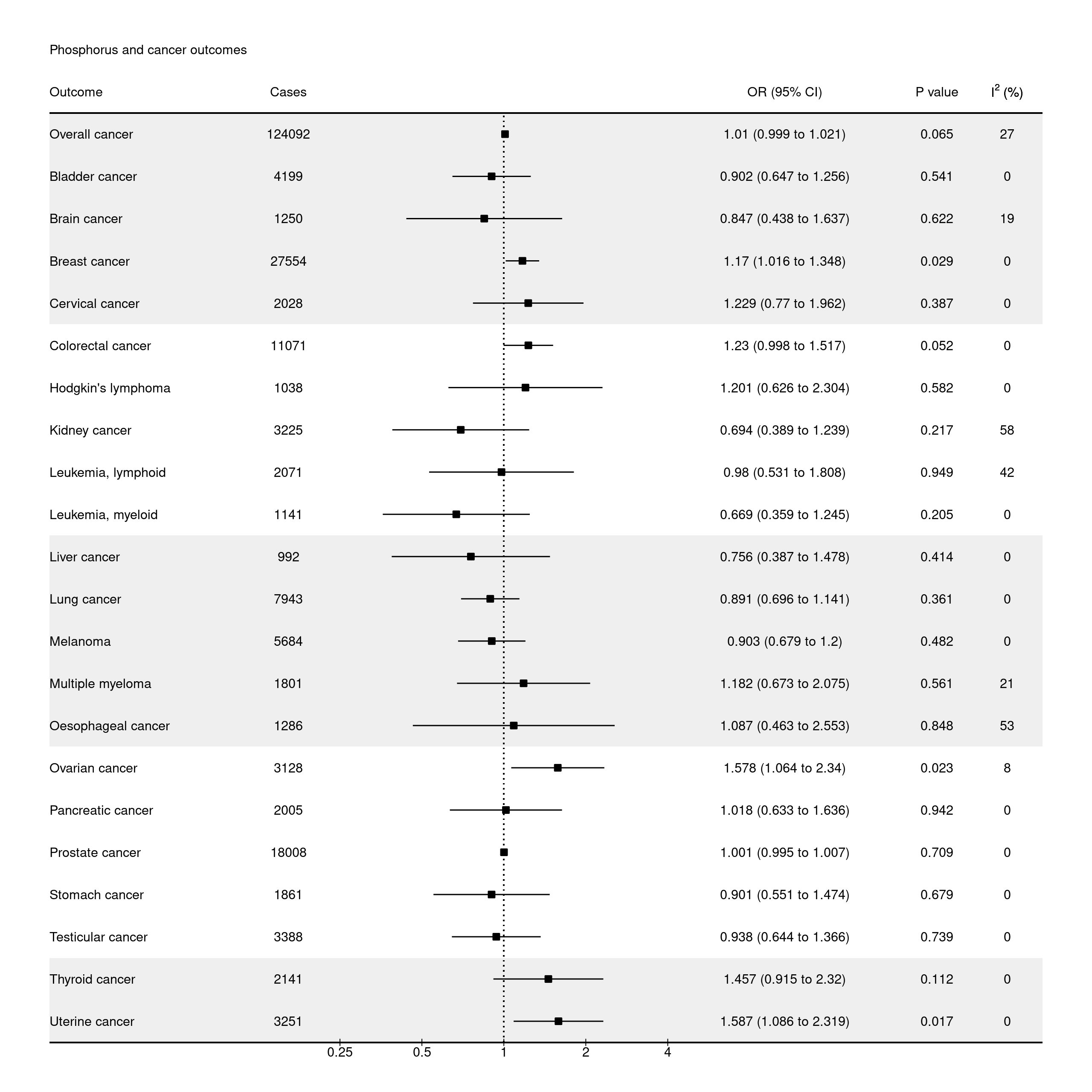


# Supplementary Figure 6. Genetic association of selenium with cancer outcomes


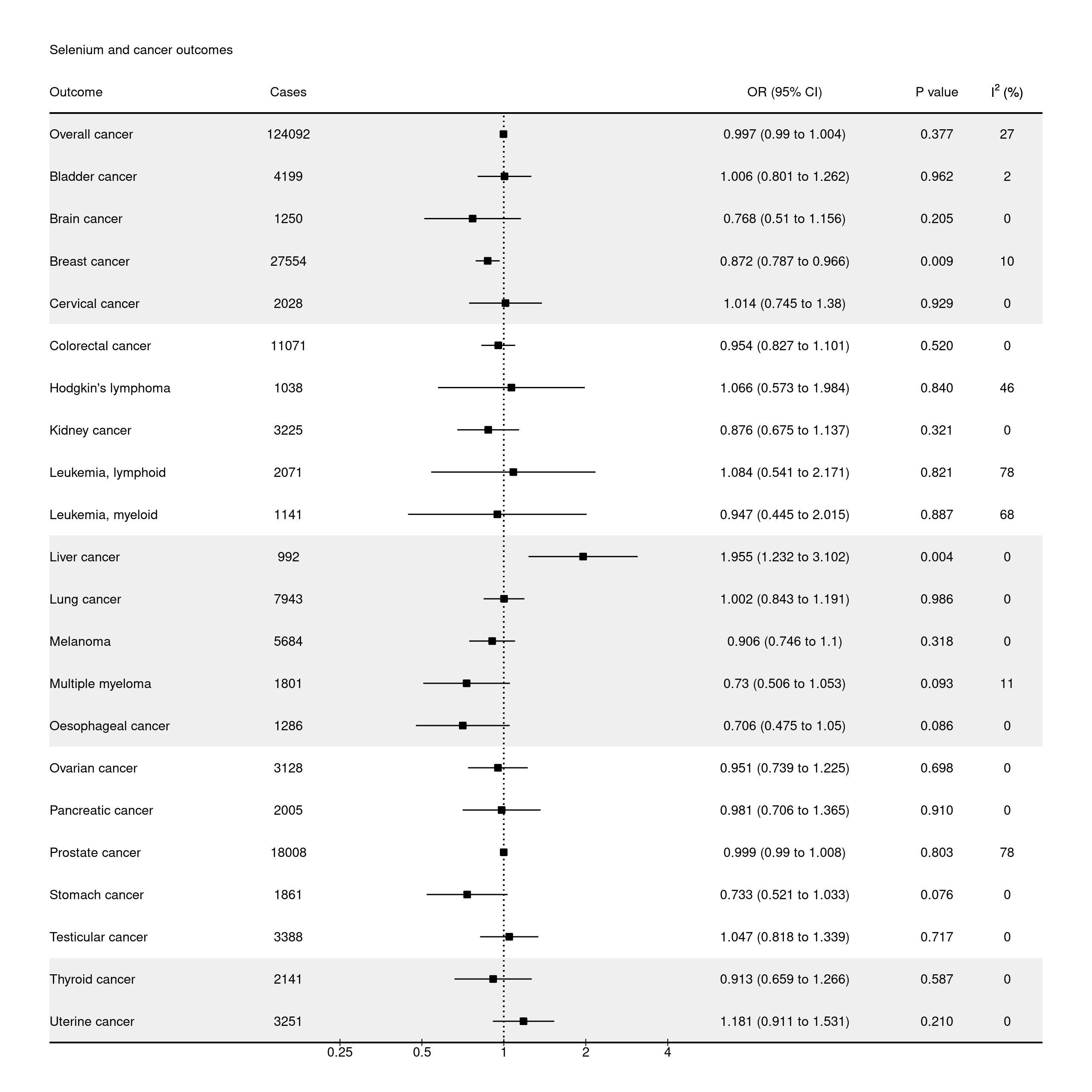


# Supplementary Figure 7. Genetic association of zinc with cancer outcomes


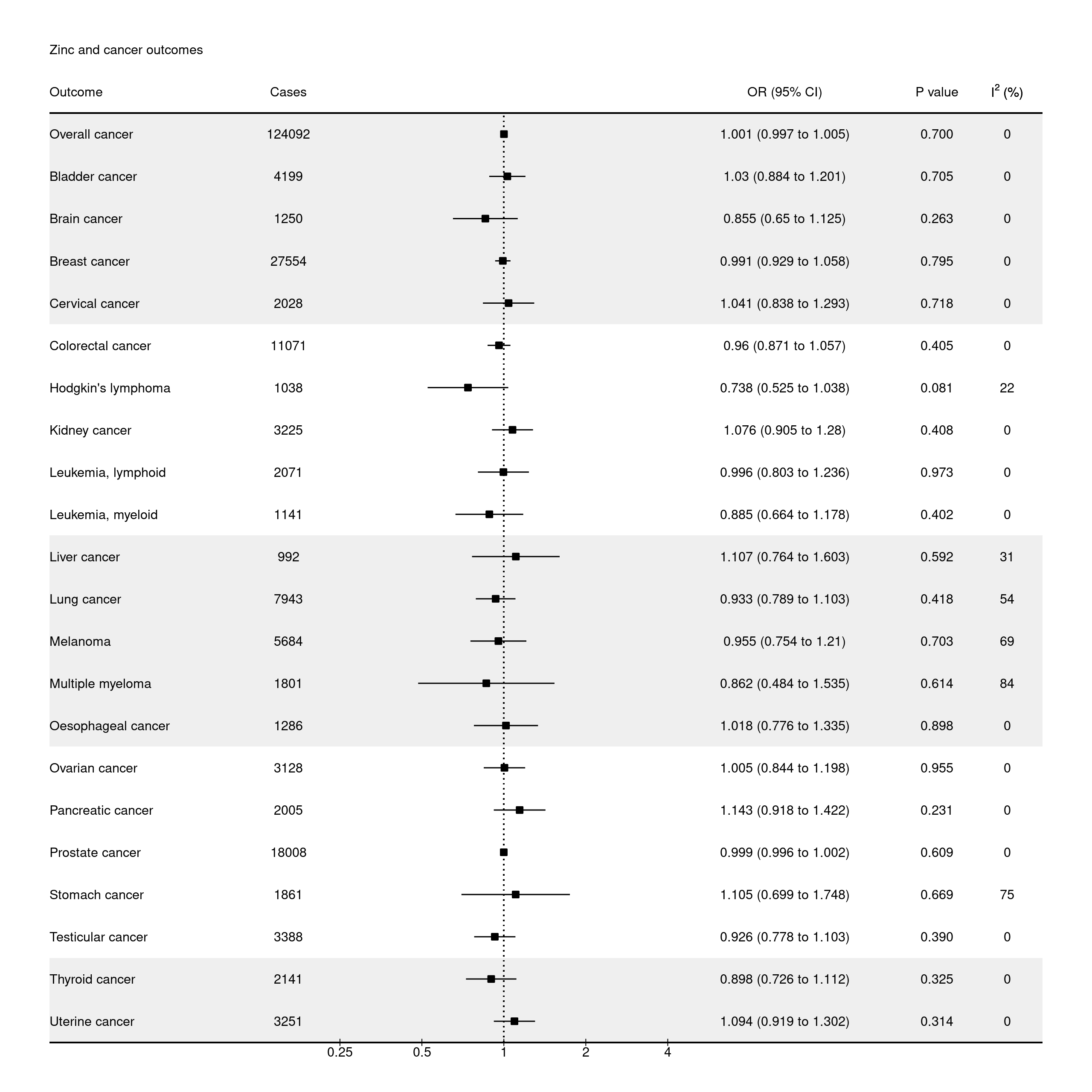


# Supplementary Figure 8. Genetic association of vitamin A1 (retinol) with cancer outcomes


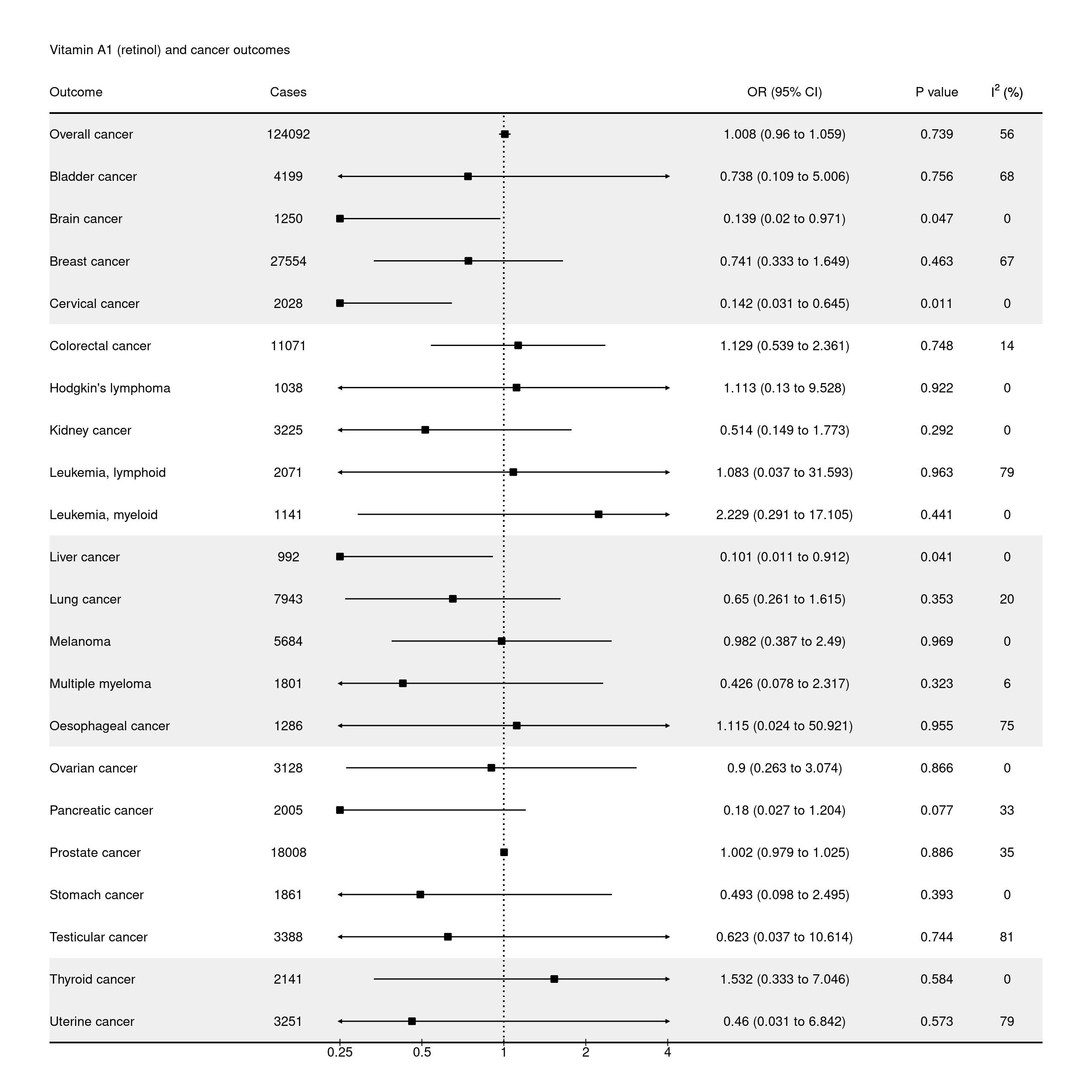


# Supplementary Figure 9. Genetic association of vitamin B6 with cancer outcomes


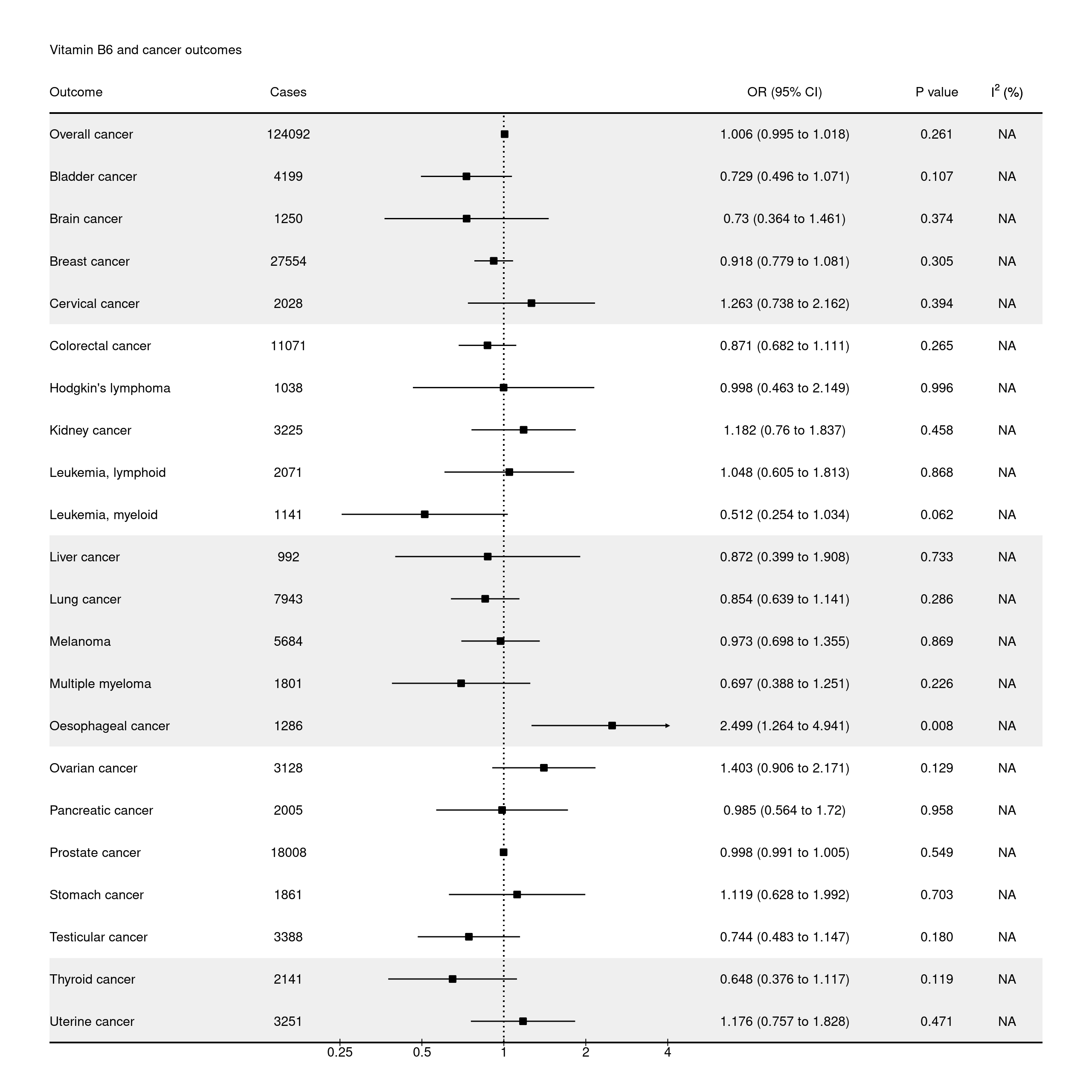


# Supplementary Figure 10. Genetic association of vitamin B9 (folate) with cancer outcomes


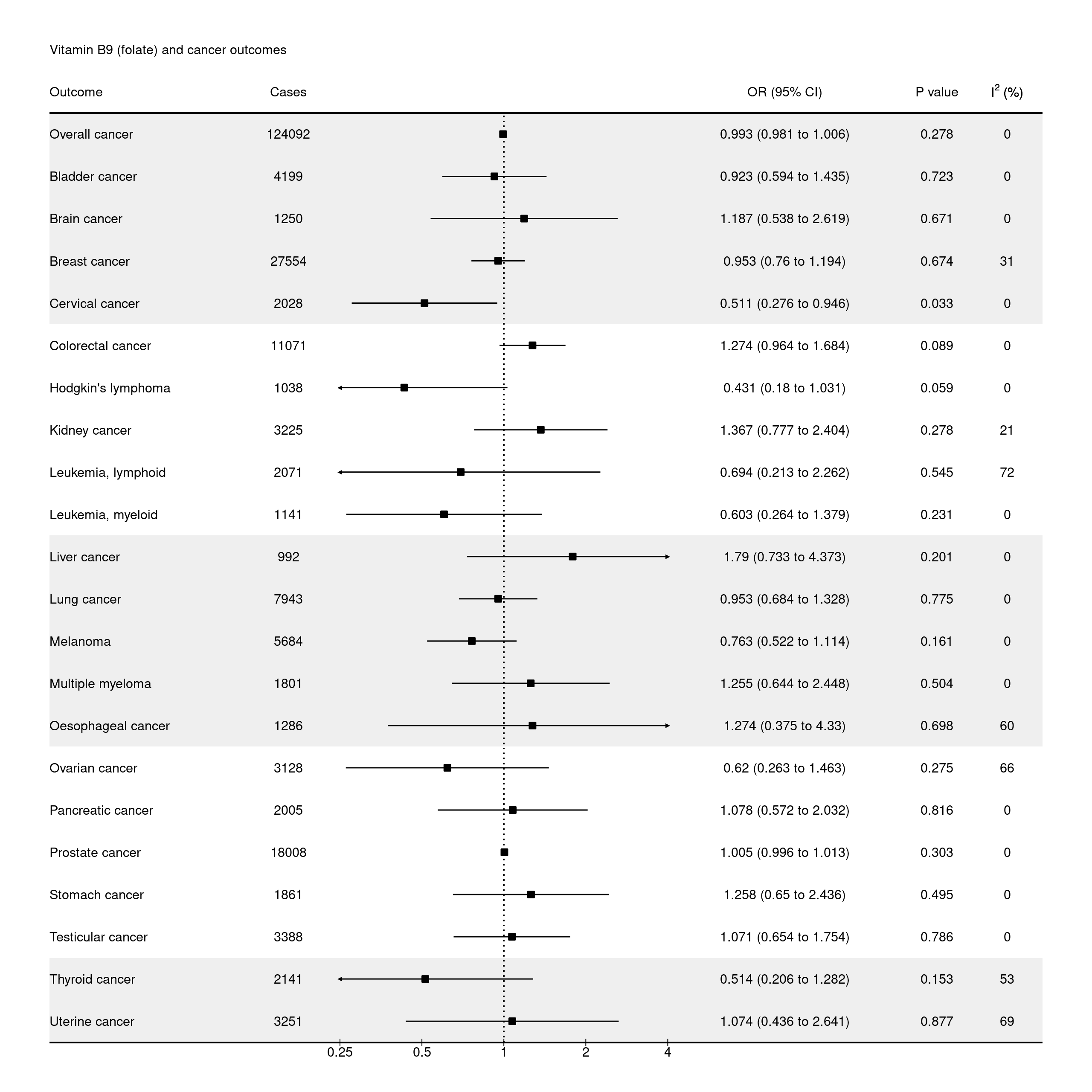


# Supplementary Figure 11. Genetic association of vitamin B12 with cancer outcomes


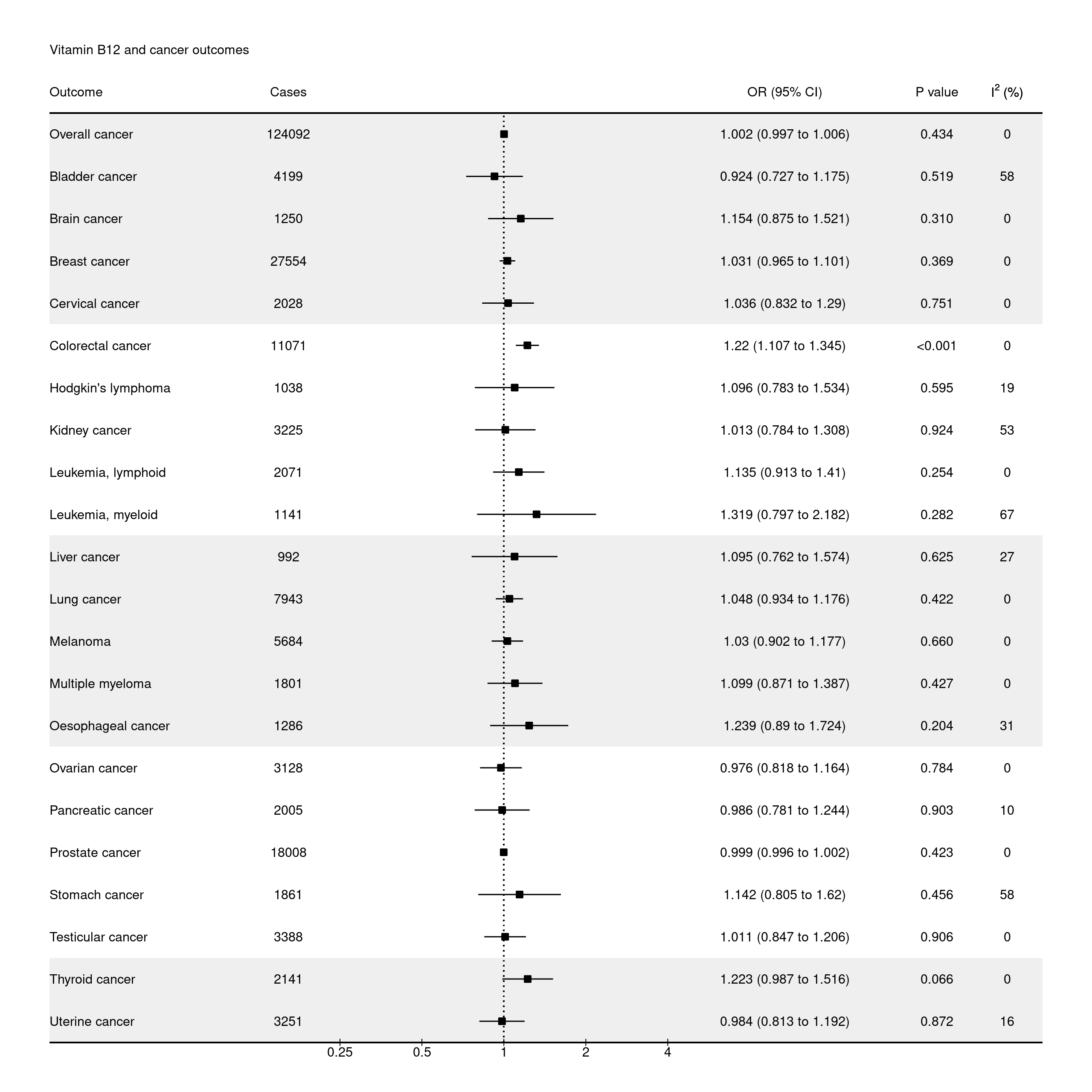


# Supplementary Figure 12. Genetic association of vitamin C with cancer outcomes


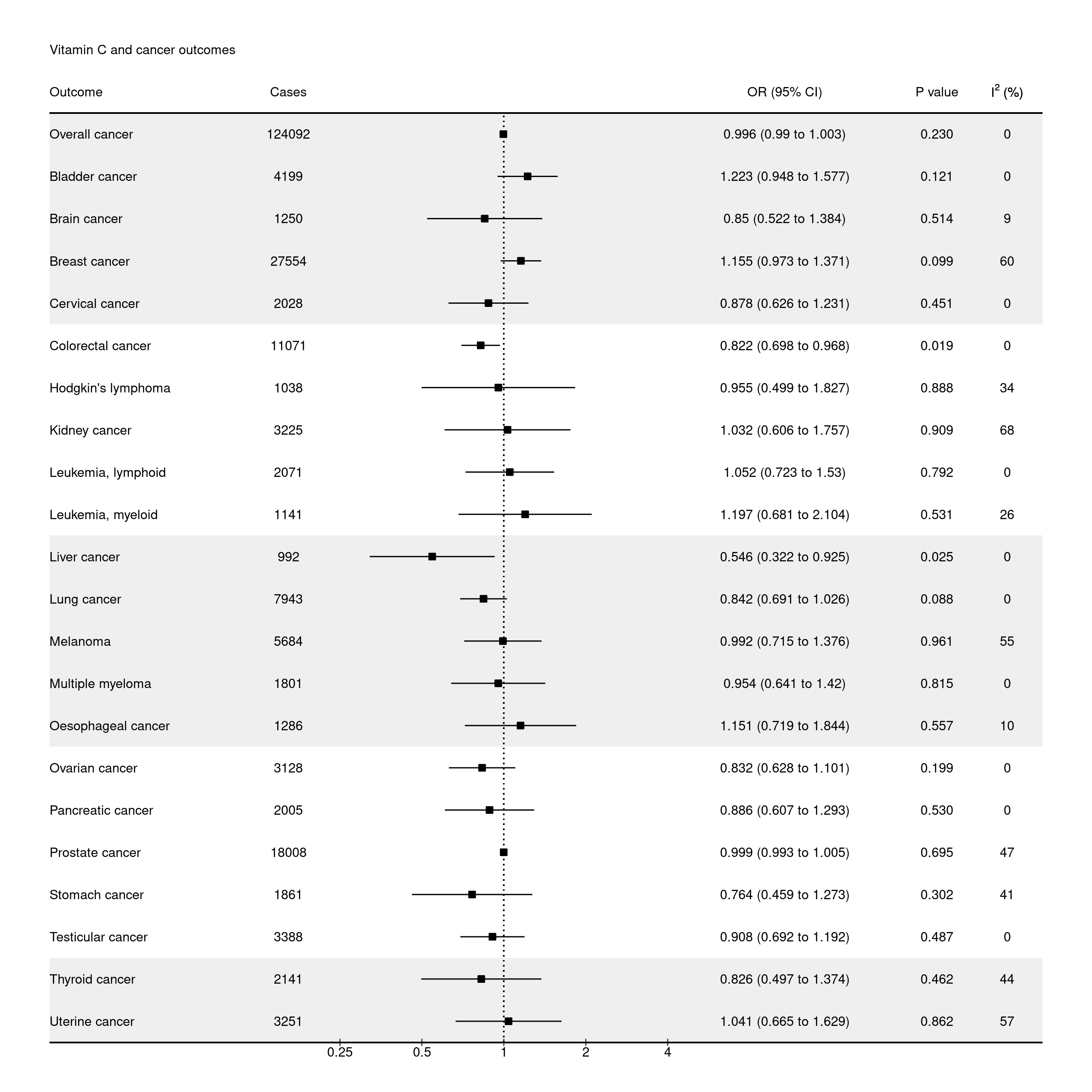


# Supplementary Figure 13. Genetic association of vitamin D (25-hydroxyvitamin D) with cancer outcomes


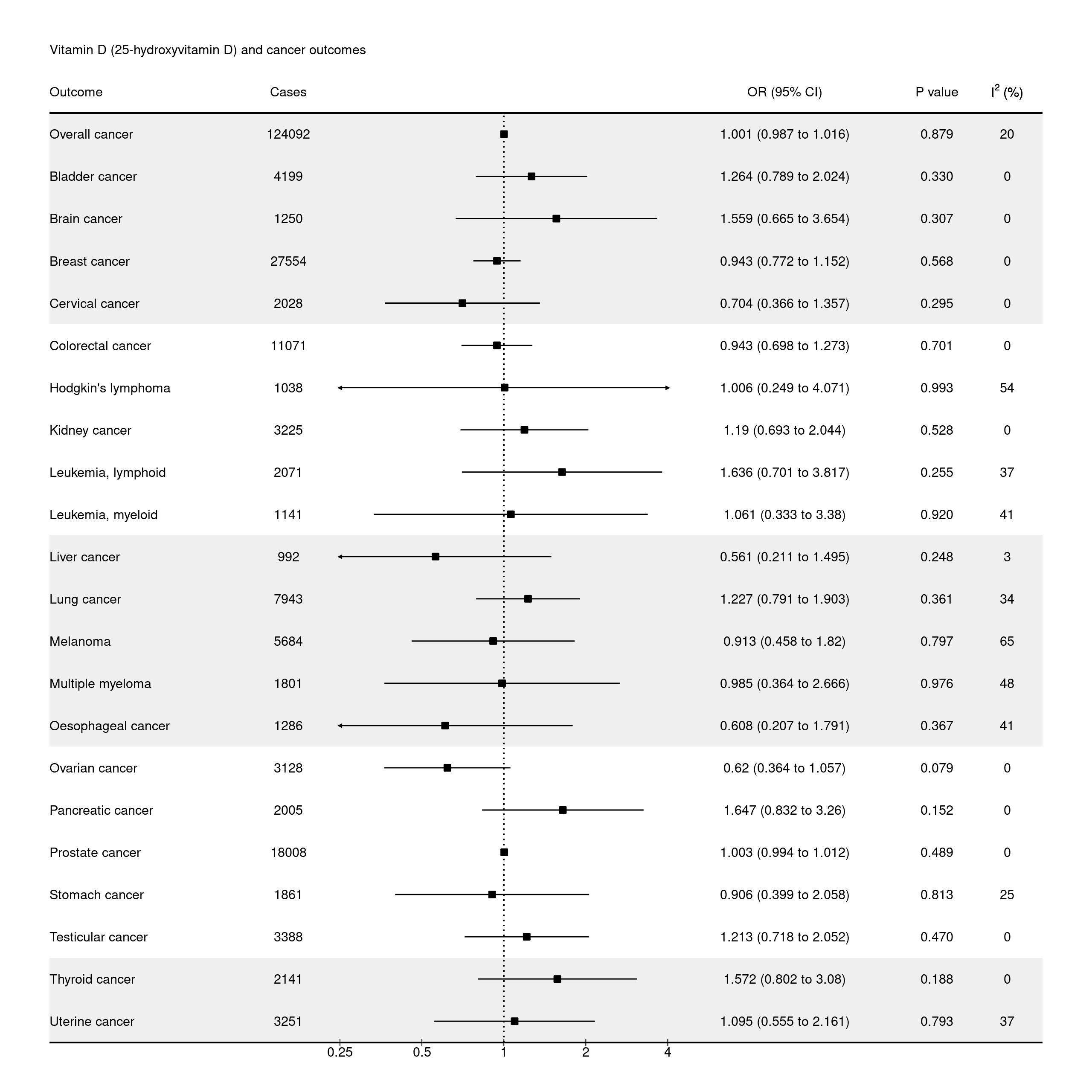


# Supplementary Figure 14. Genetic association of vitamin E with cancer outcomes


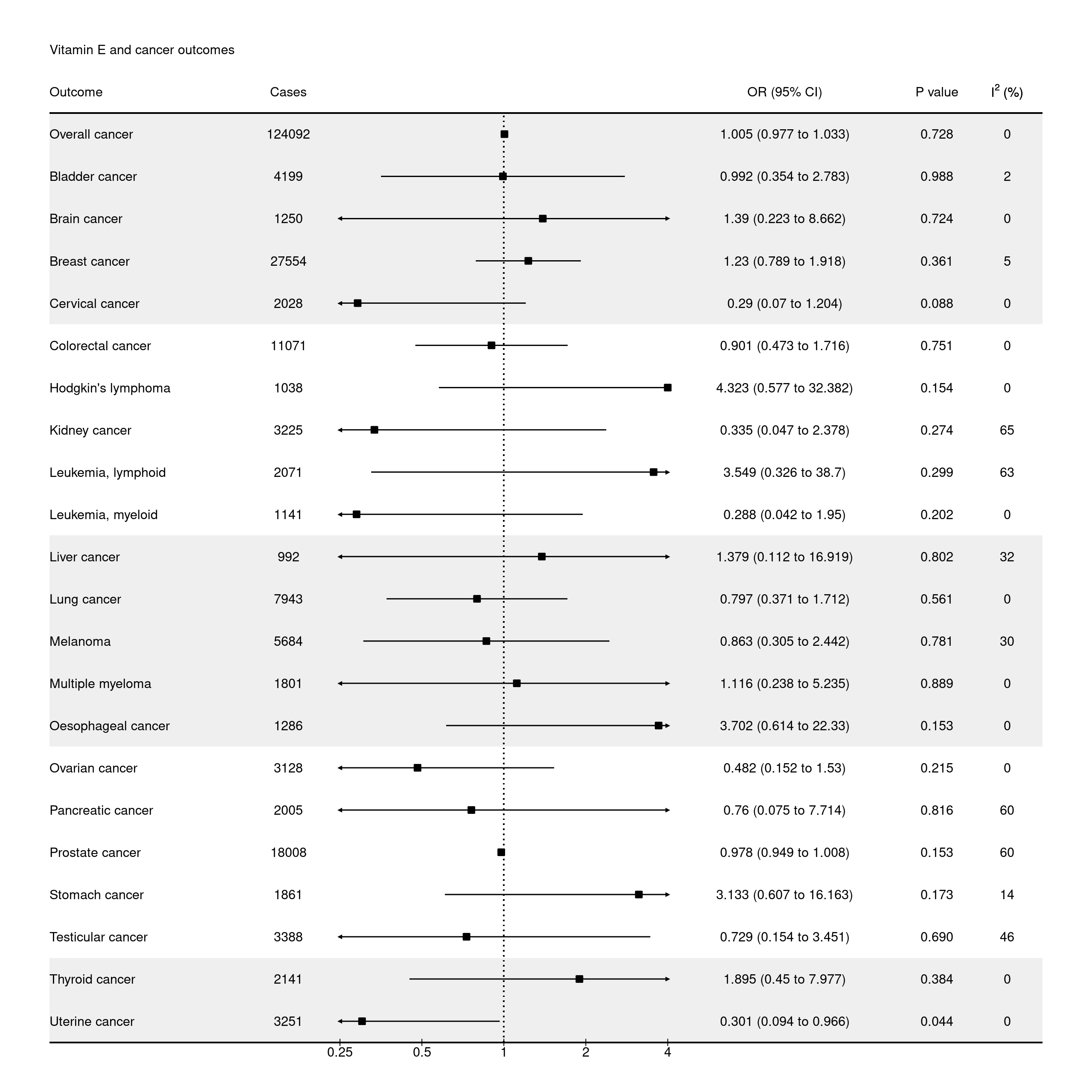


# Supplementary Figure 15. Genetic association of magnesium with breast cancer

1) Forest plot


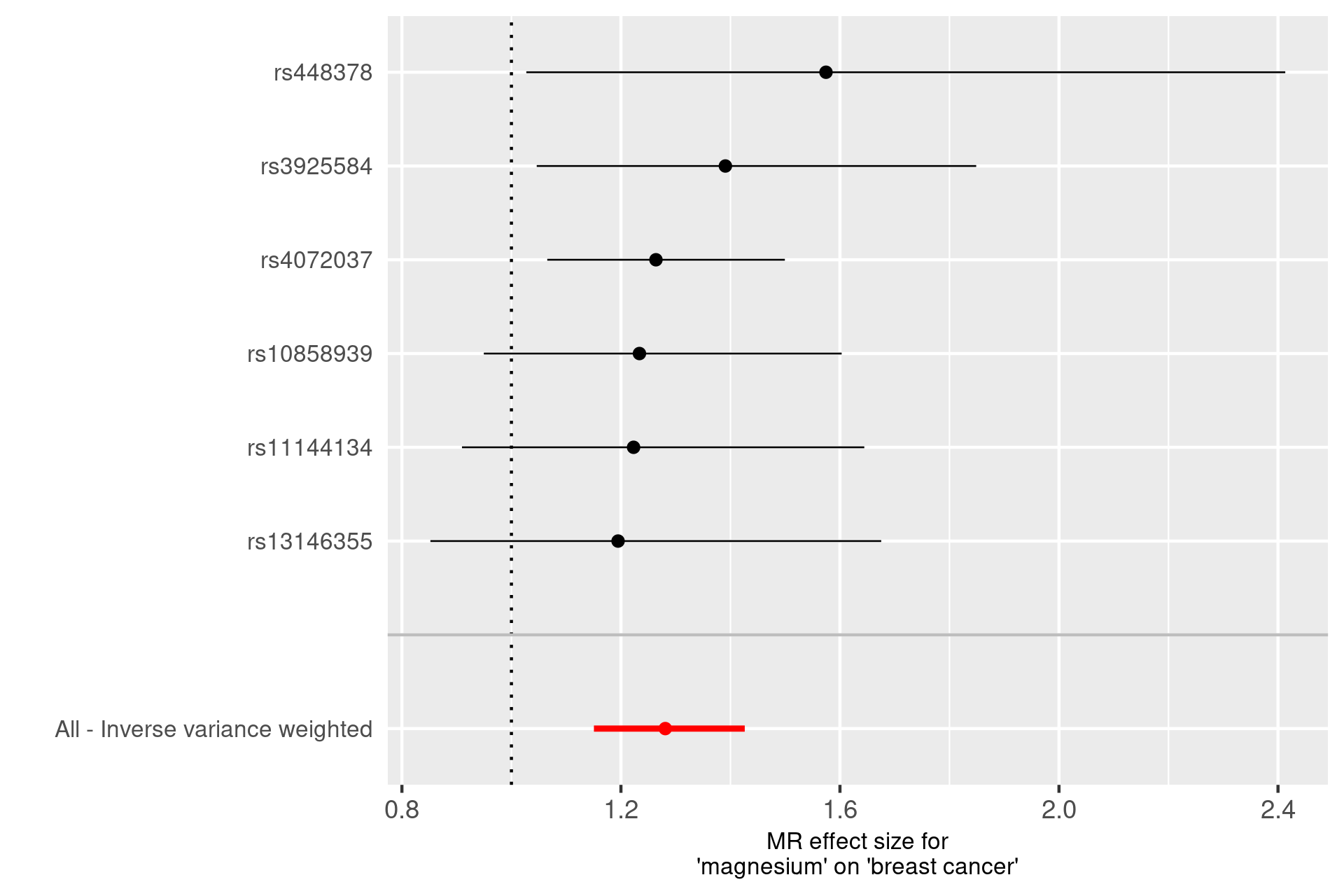


2) Leave-one-out plot


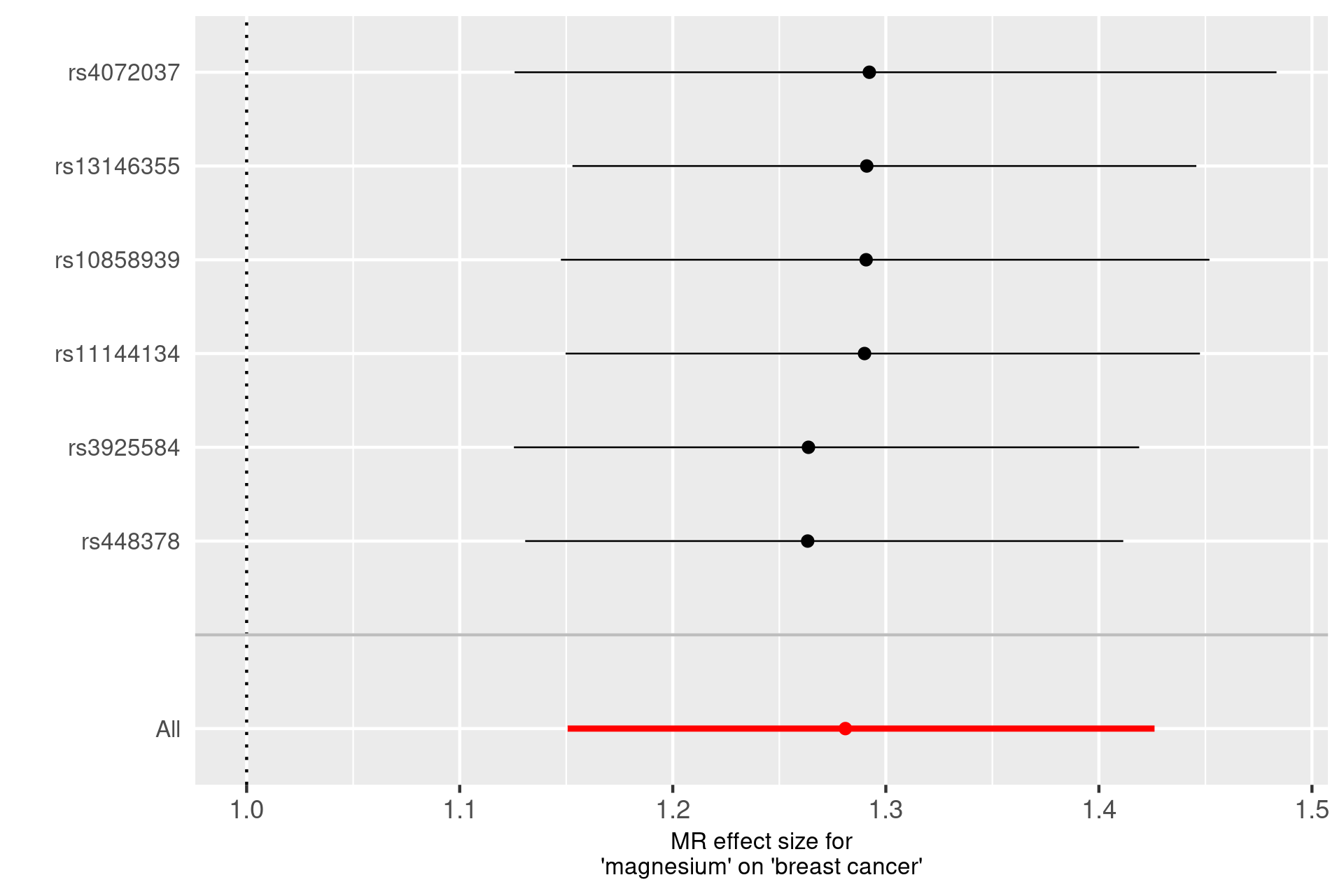


3) Scatter plot


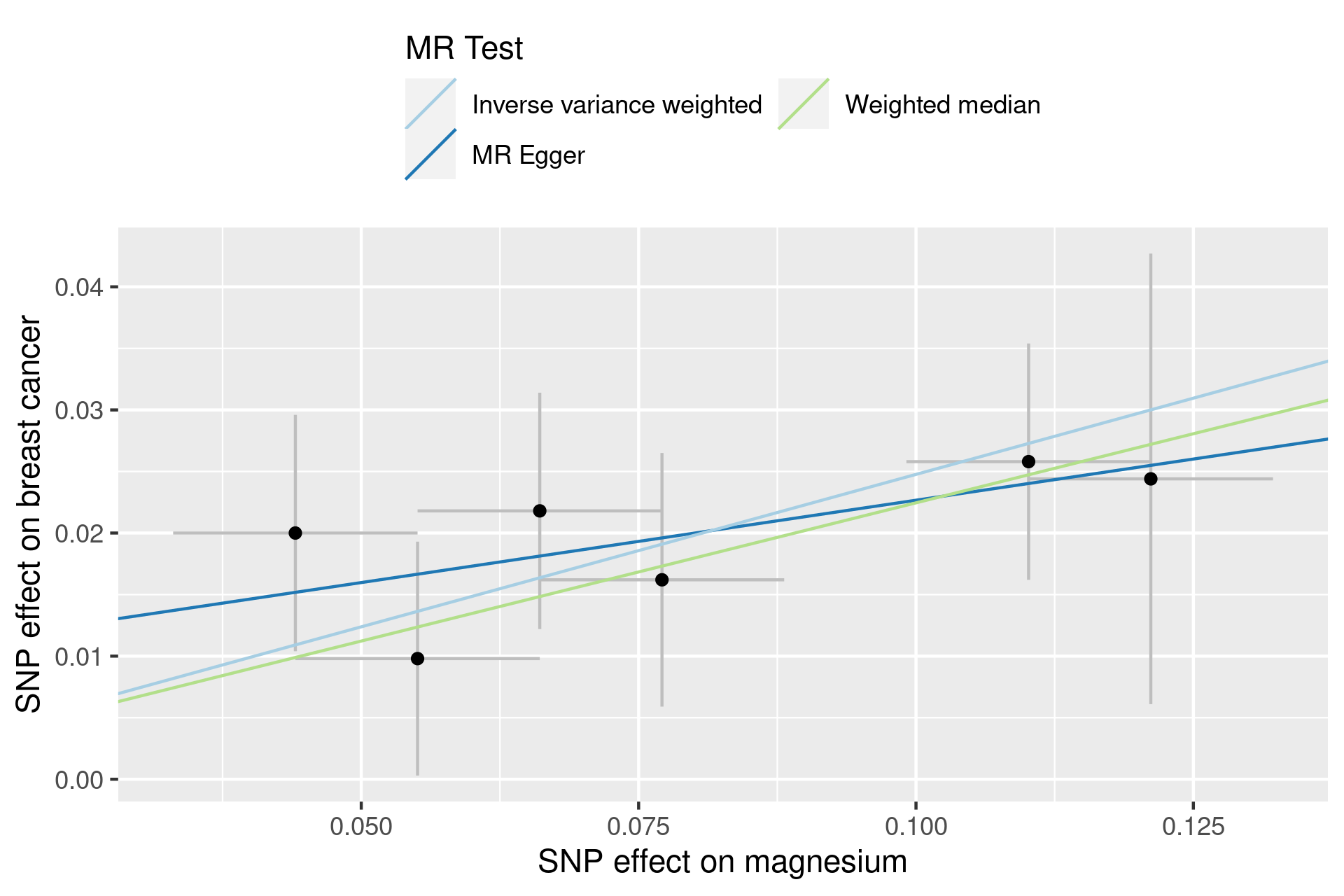


4) Funnel plot


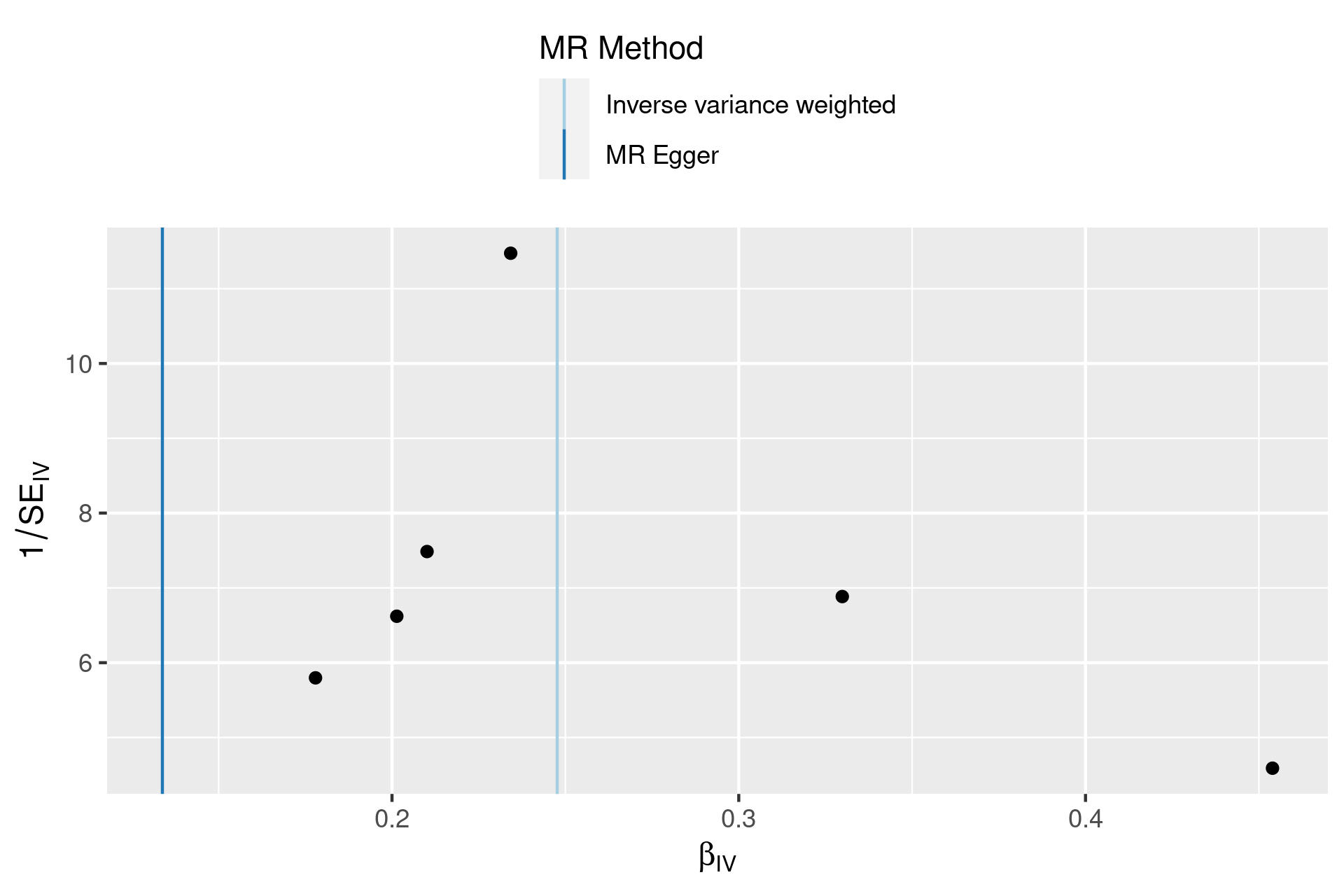


# Supplementary Figure 16. Genetic association of vitamin B12 with colorectal cancer

1) Forest plot


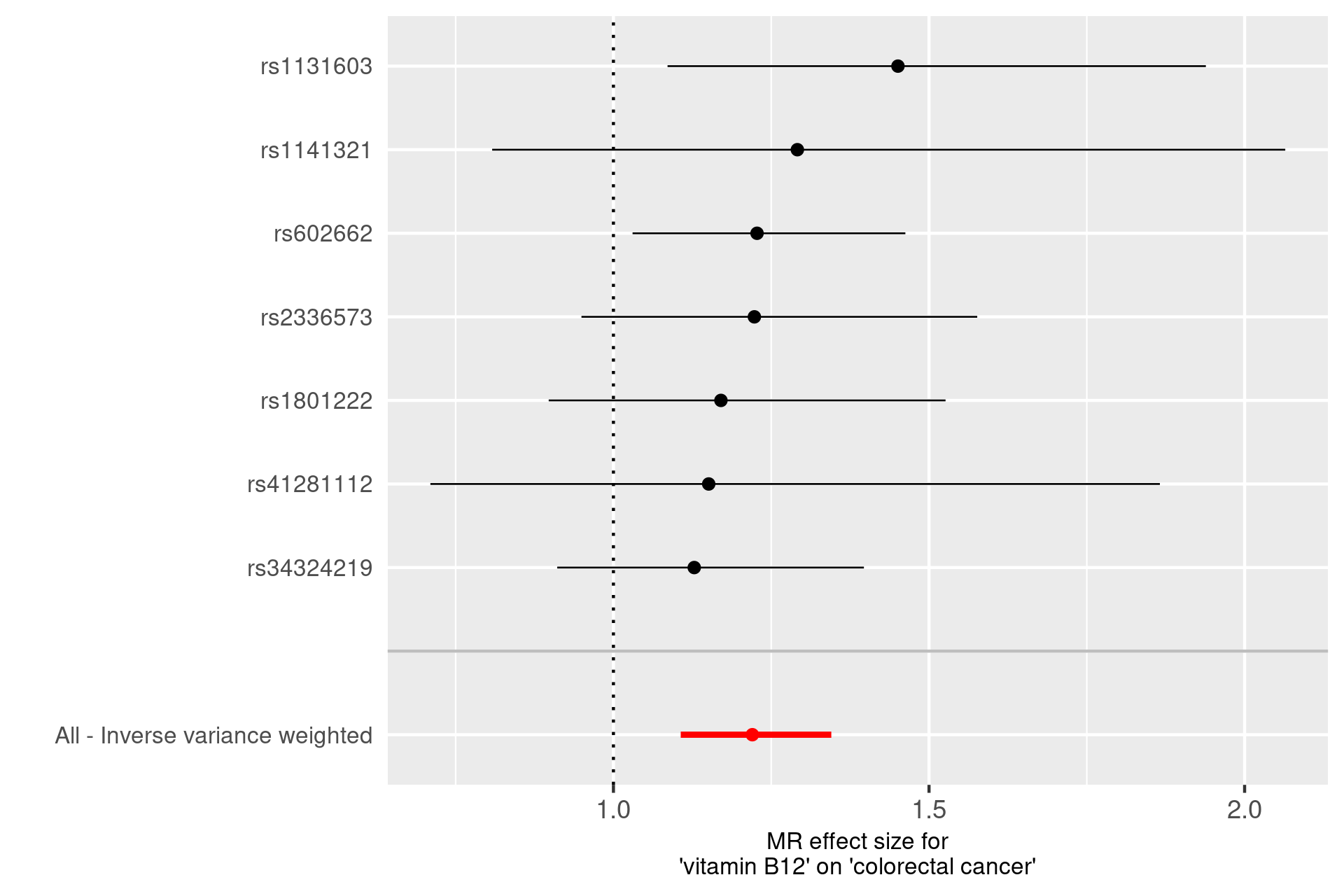


2) Leave-one-out plot


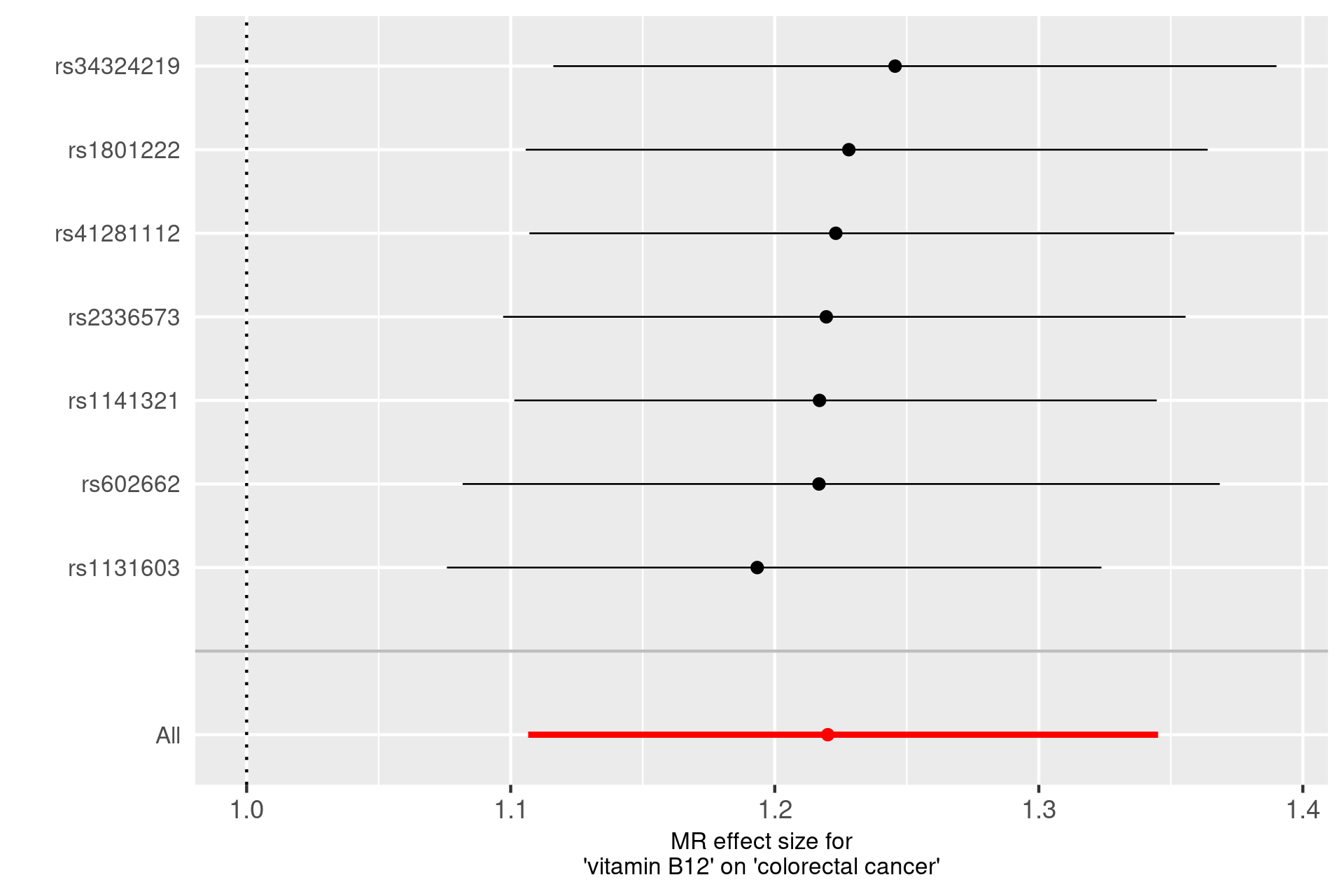


3) Scatter plot


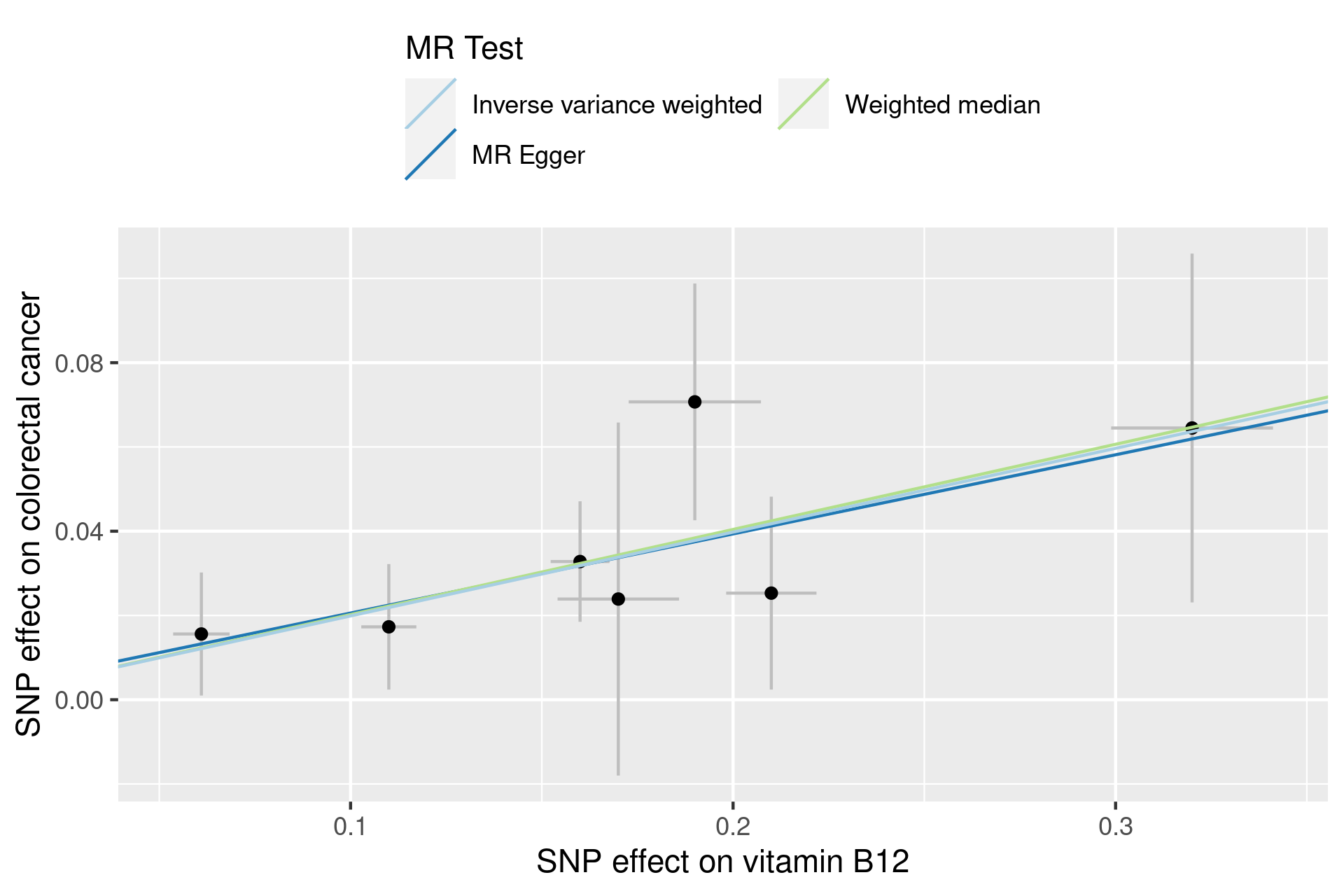


4) Funnel plot


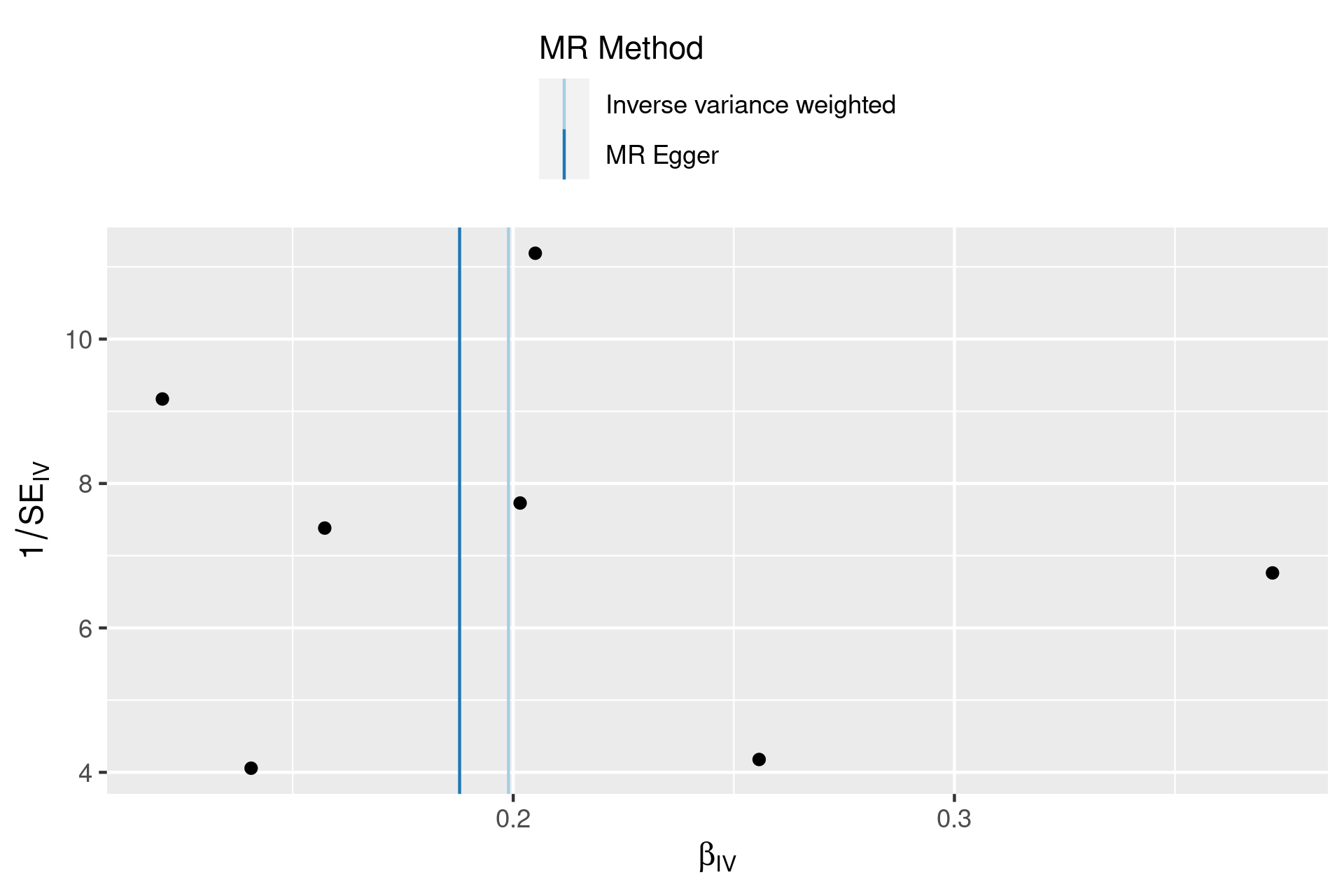


# Supplementary Figure 17. Genetic association of magnesium with lung cancer

1) Forest plot


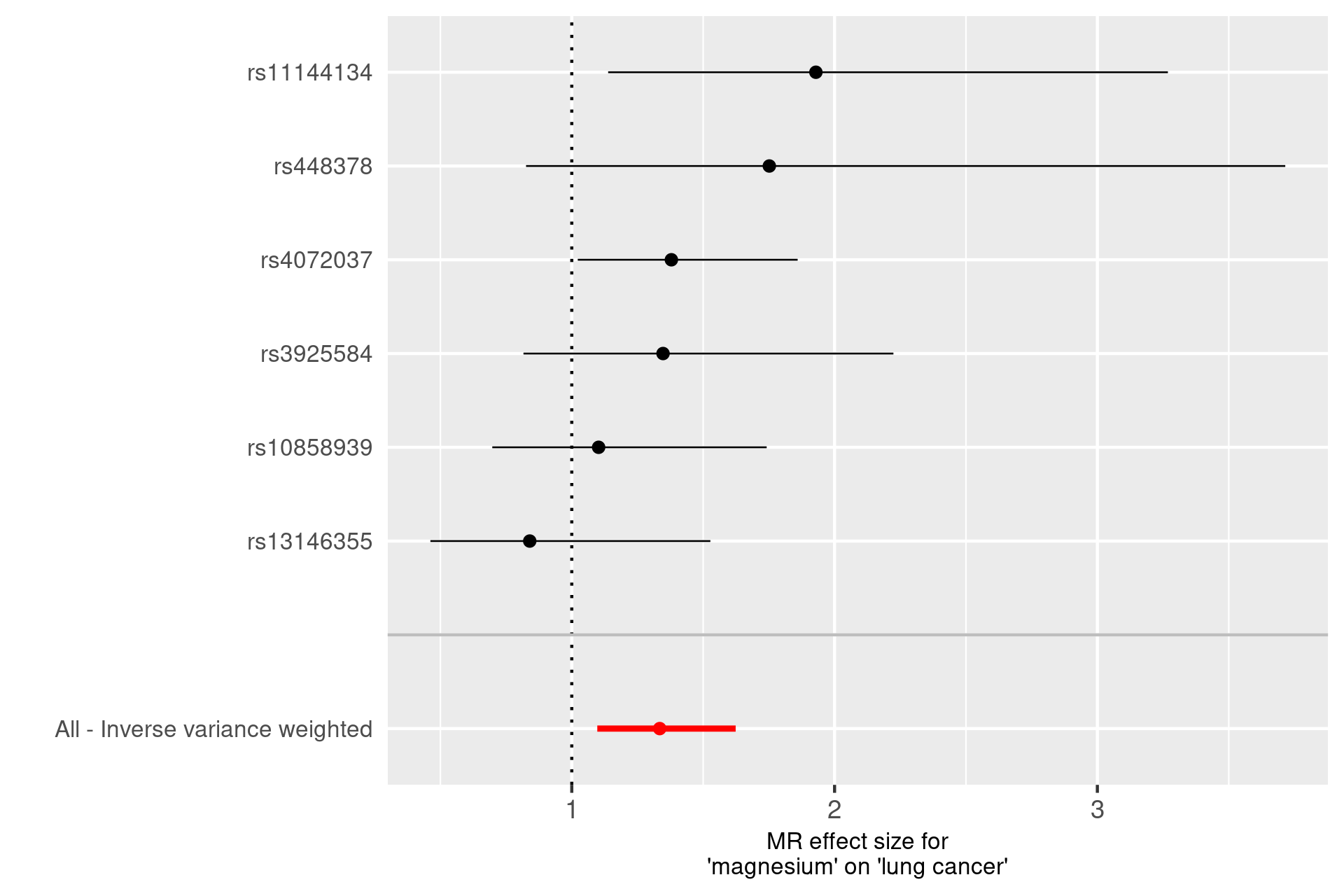


2) Leave-one-out plot


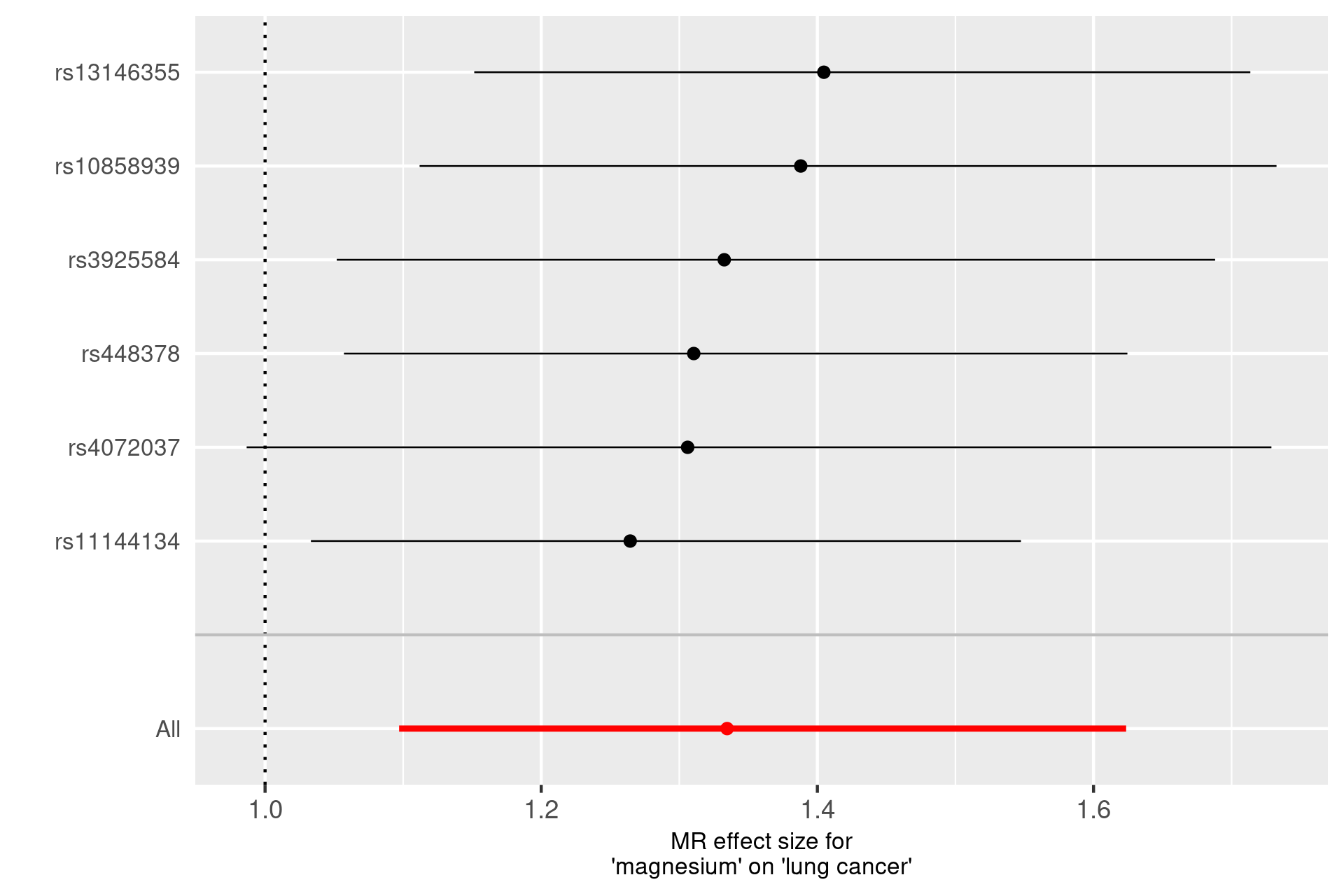


3) Scatter plot


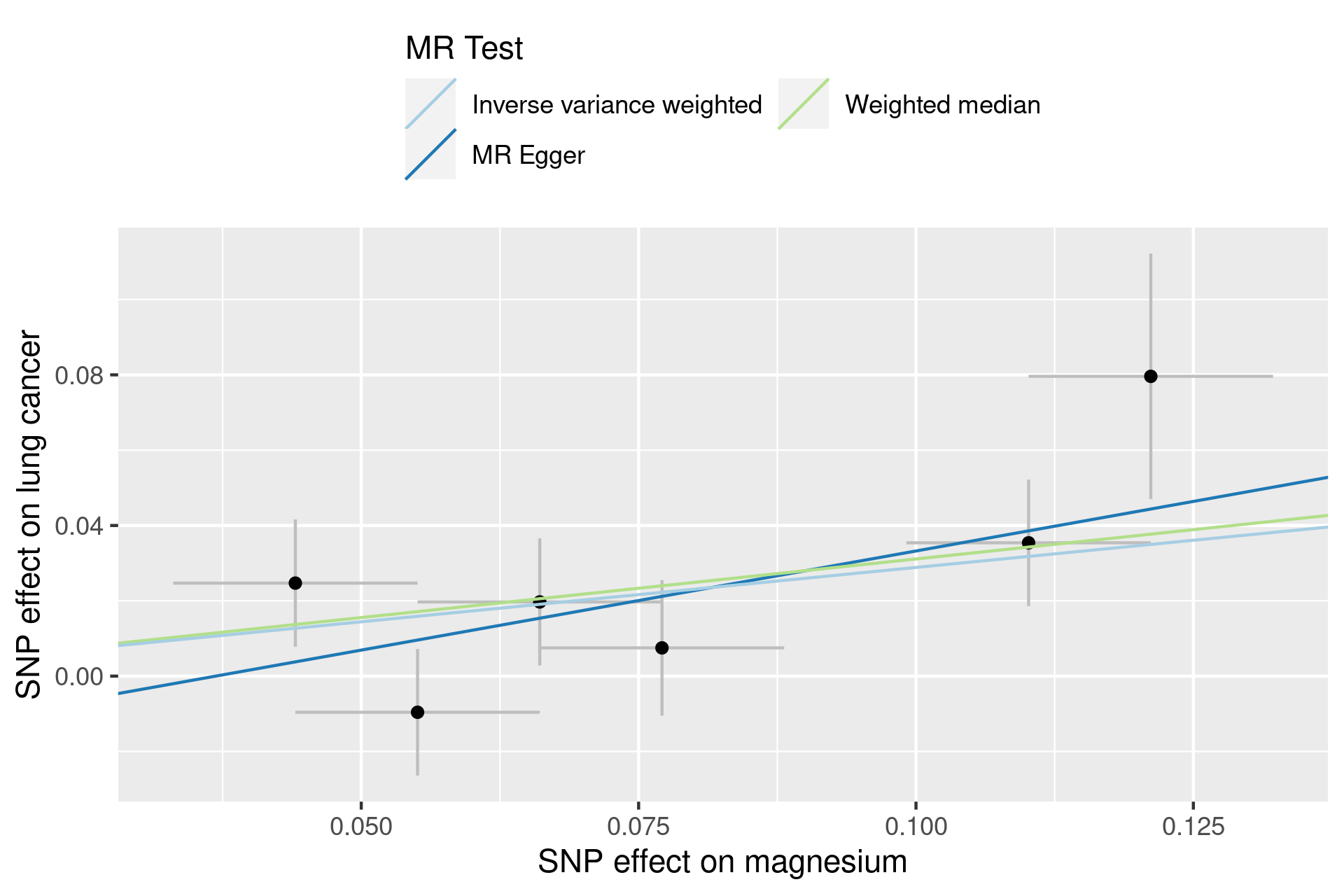


4) Funnel plot


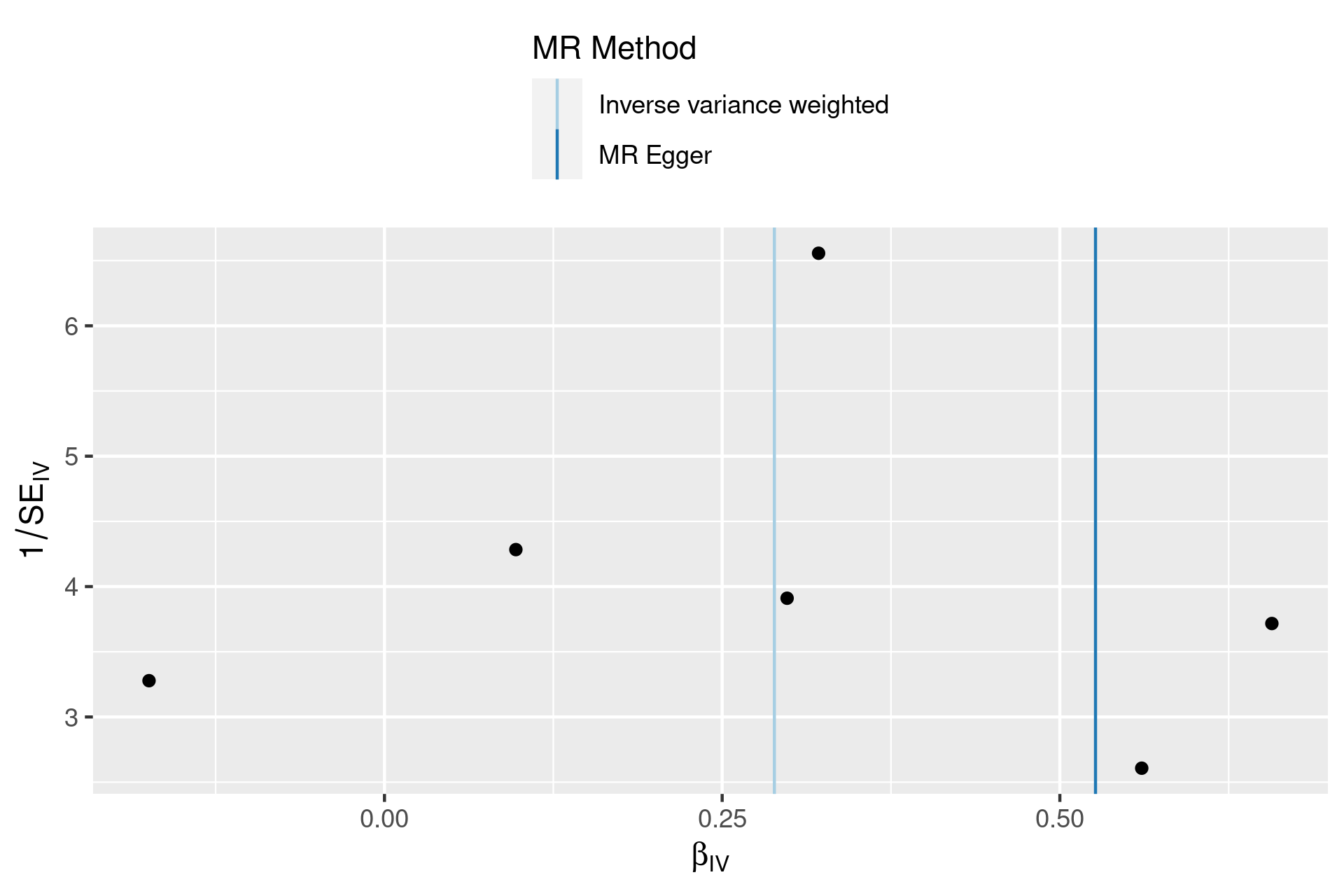


# Supplementary Figure 18. Genetic association of selenium with liver cancer

1) Forest plot


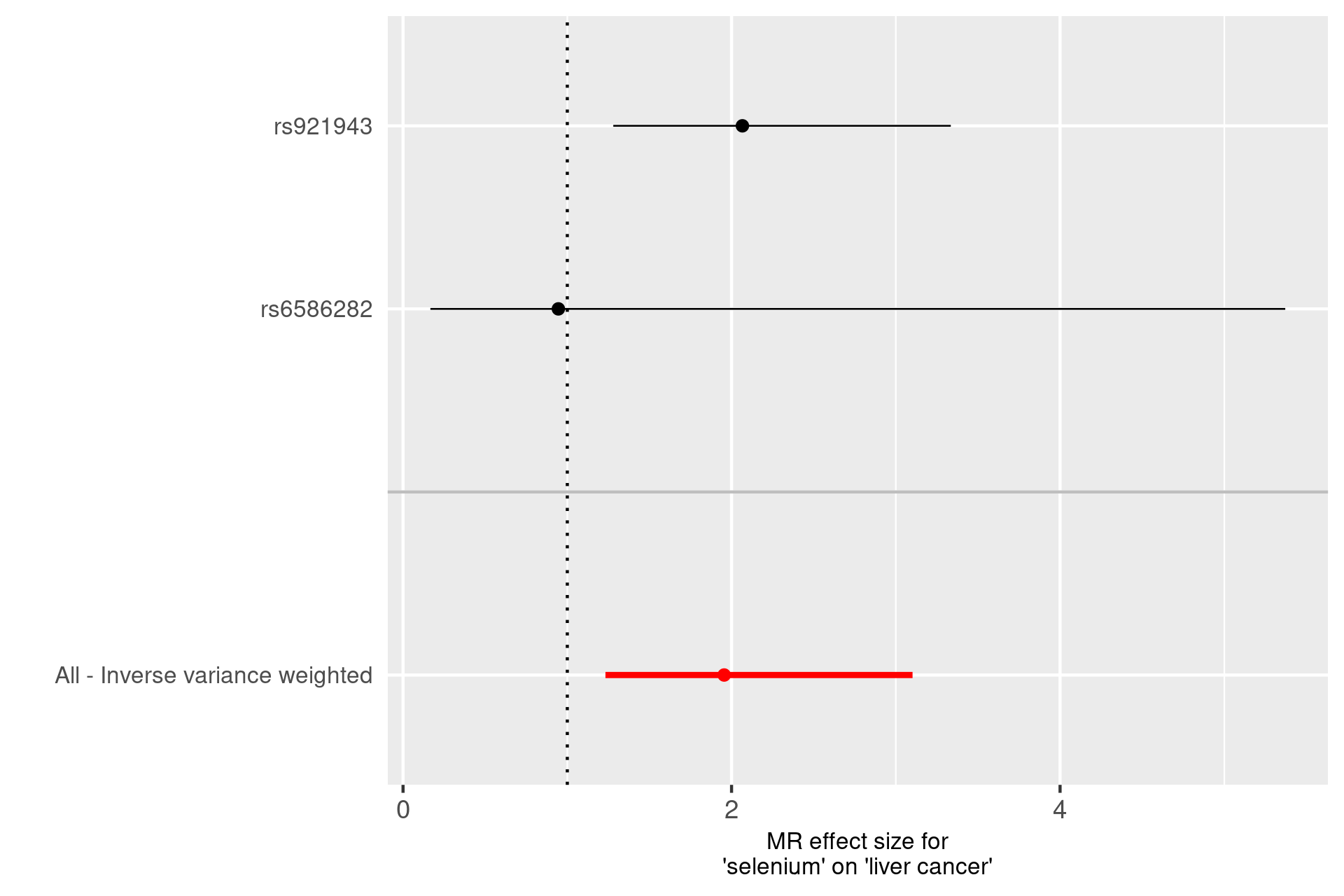


2) Leave-one-out plot

Not available because of small number of SNPs

3) Scatter plot


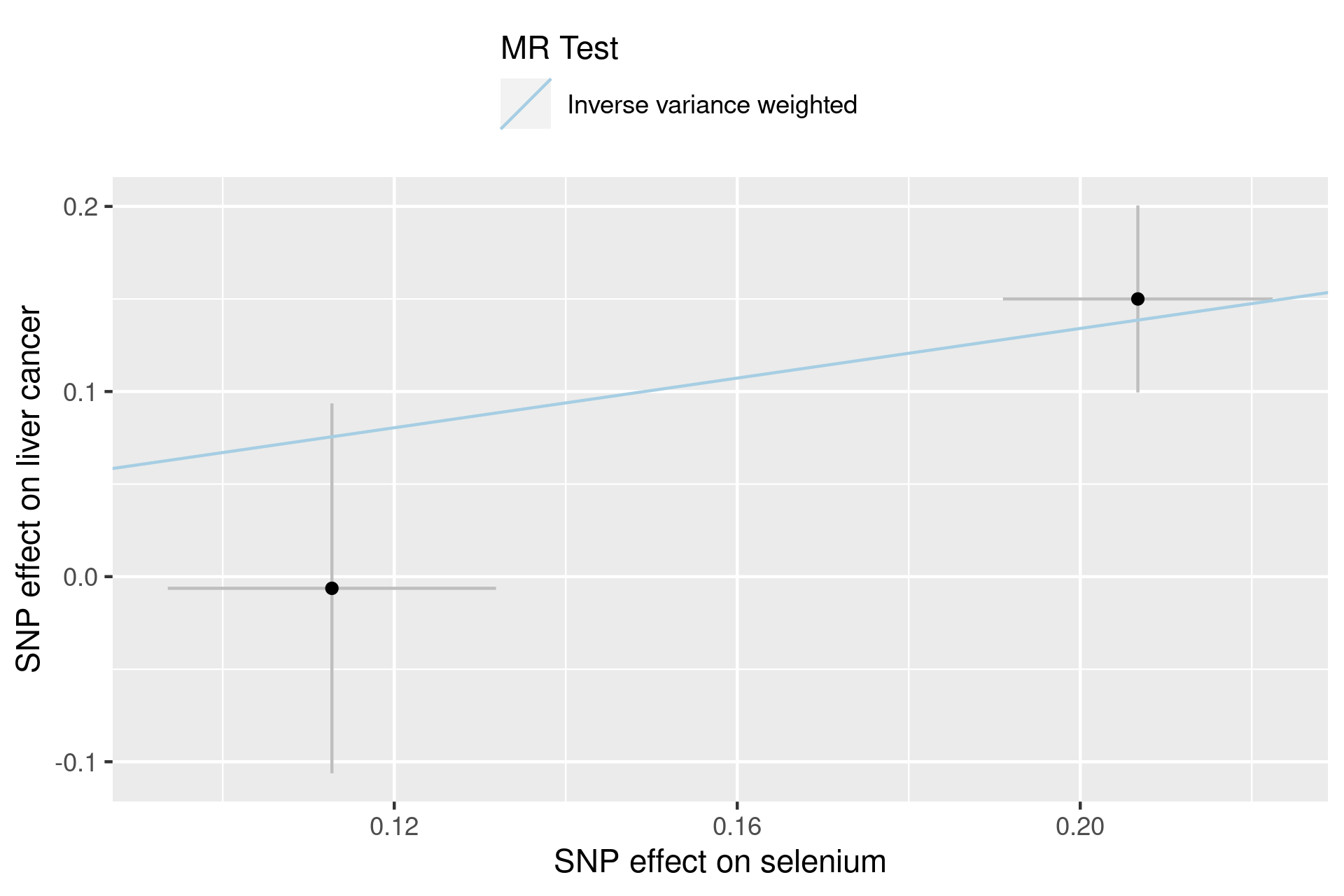


4) Funnel plot


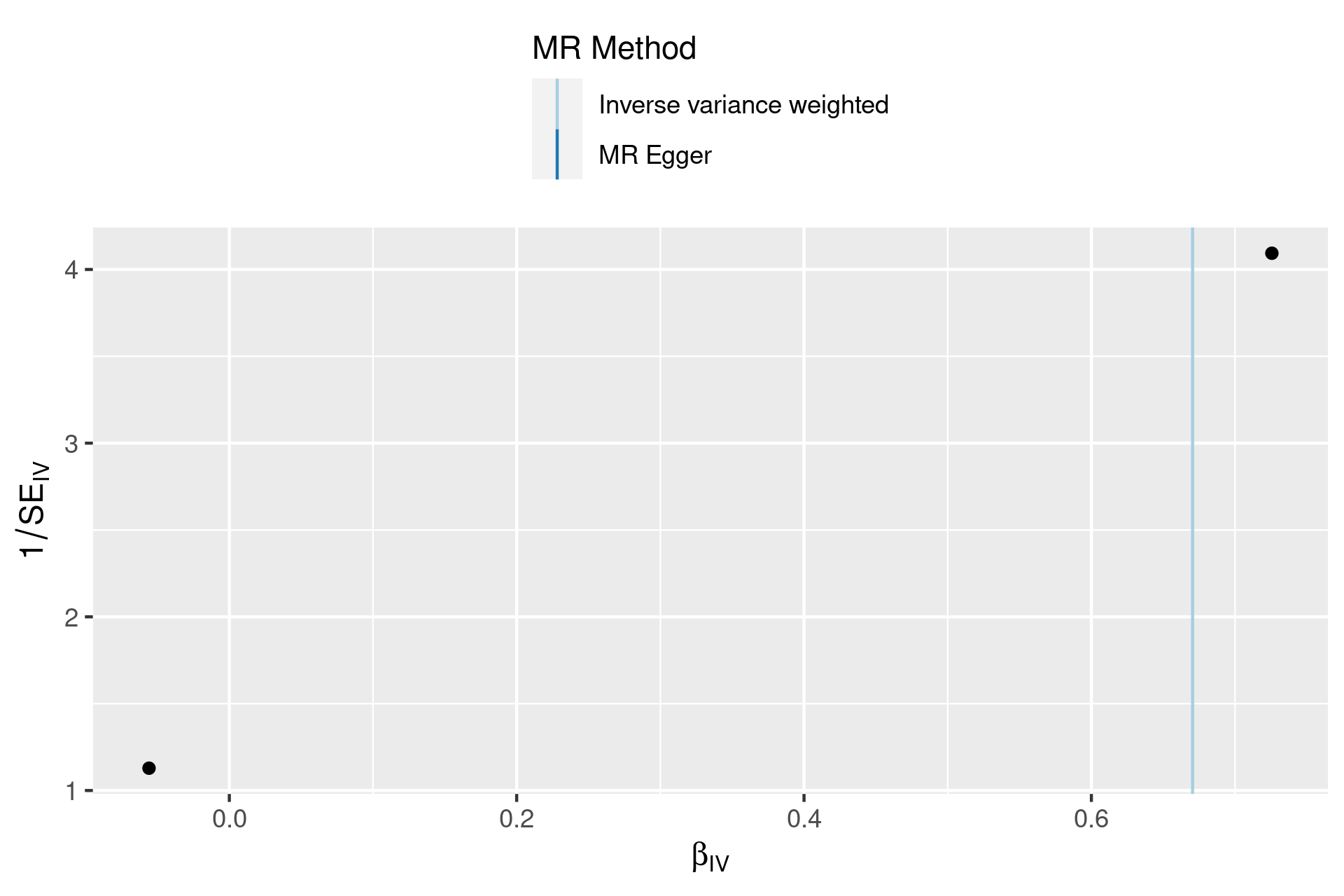


# Supplementary Figure 19. Genetic association of selenium with breast cancer

1) Forest plot


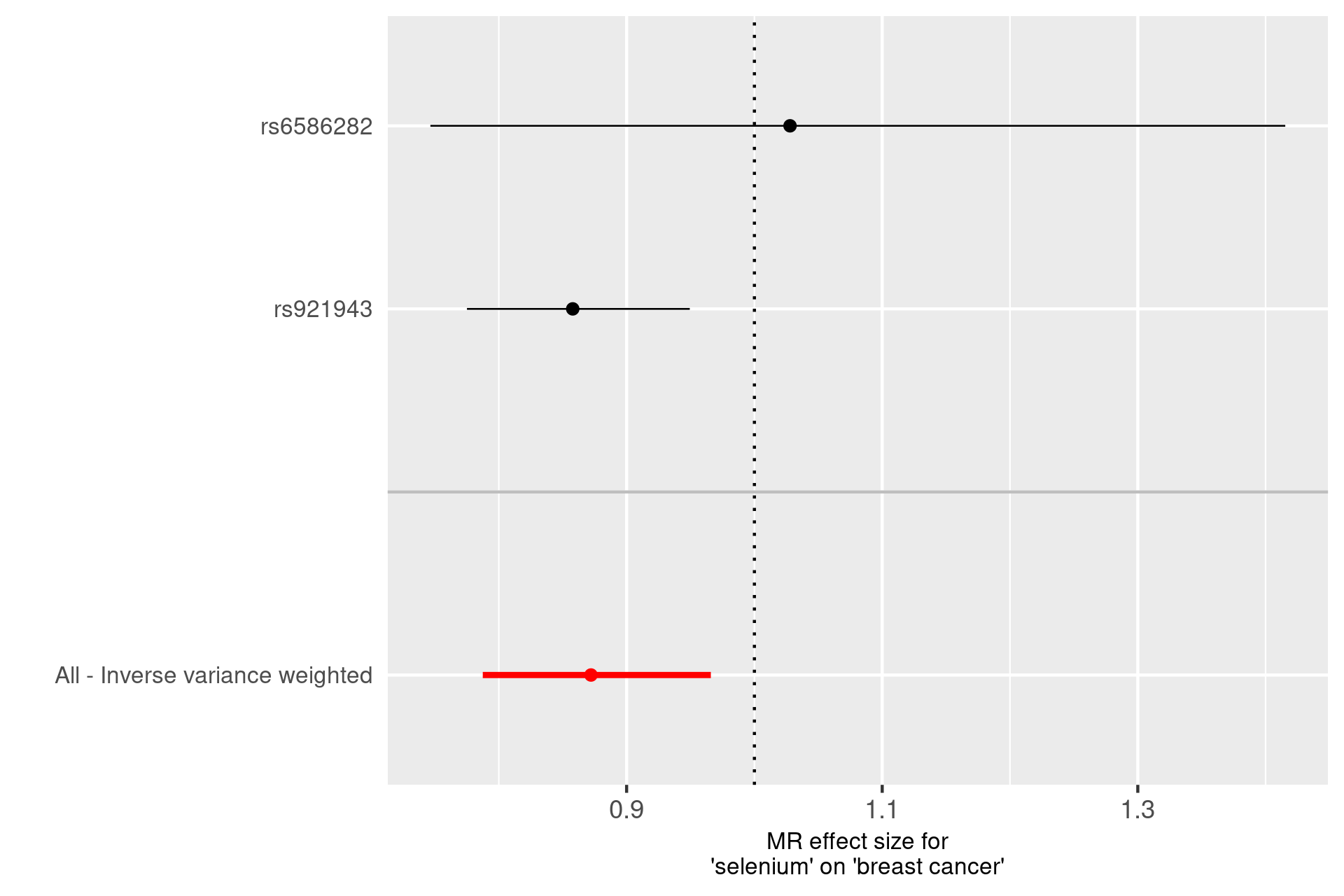


2) Leave-one-out plot

Not available because of small number of SNPs

3) Scatter plot


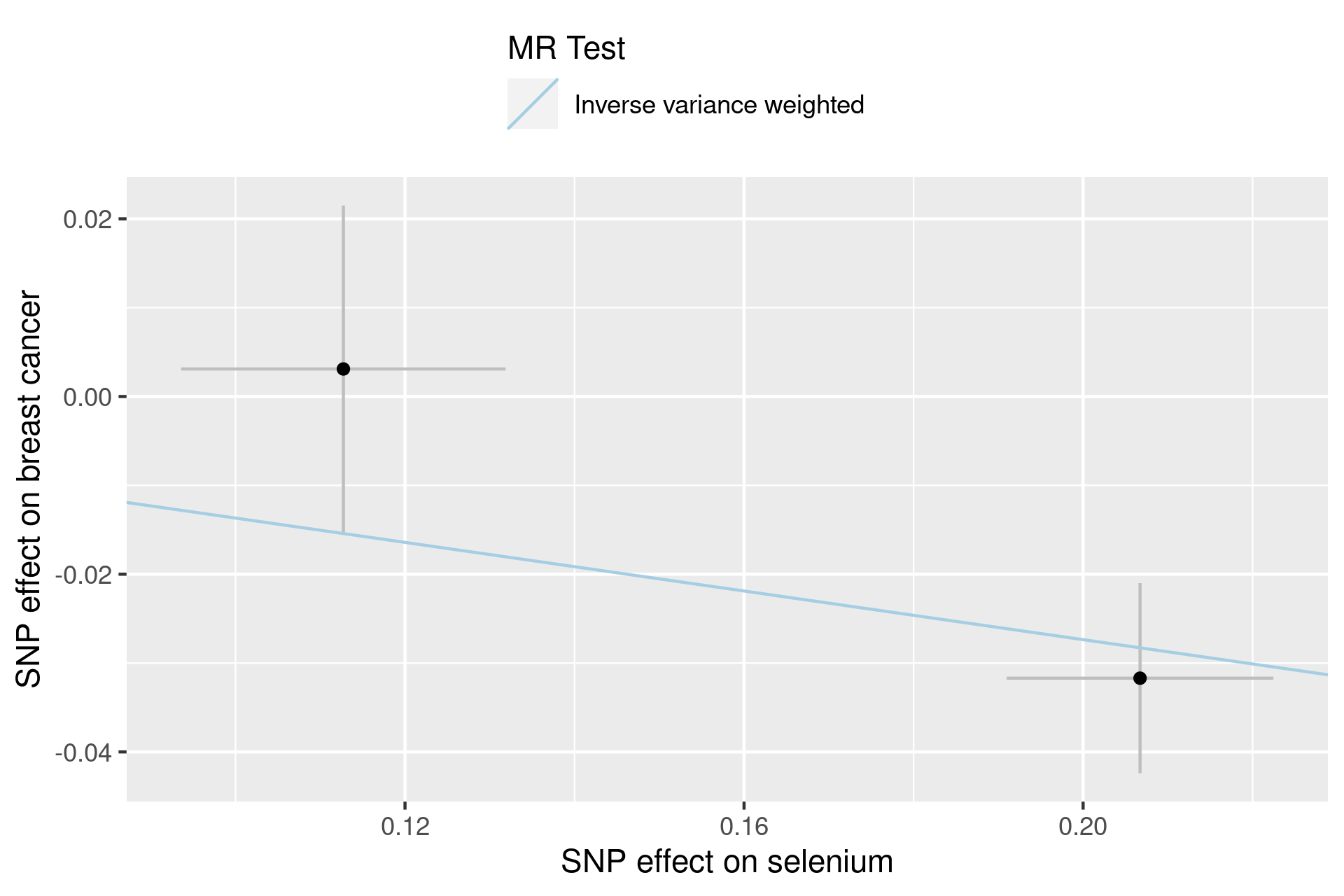


4) Funnel plot


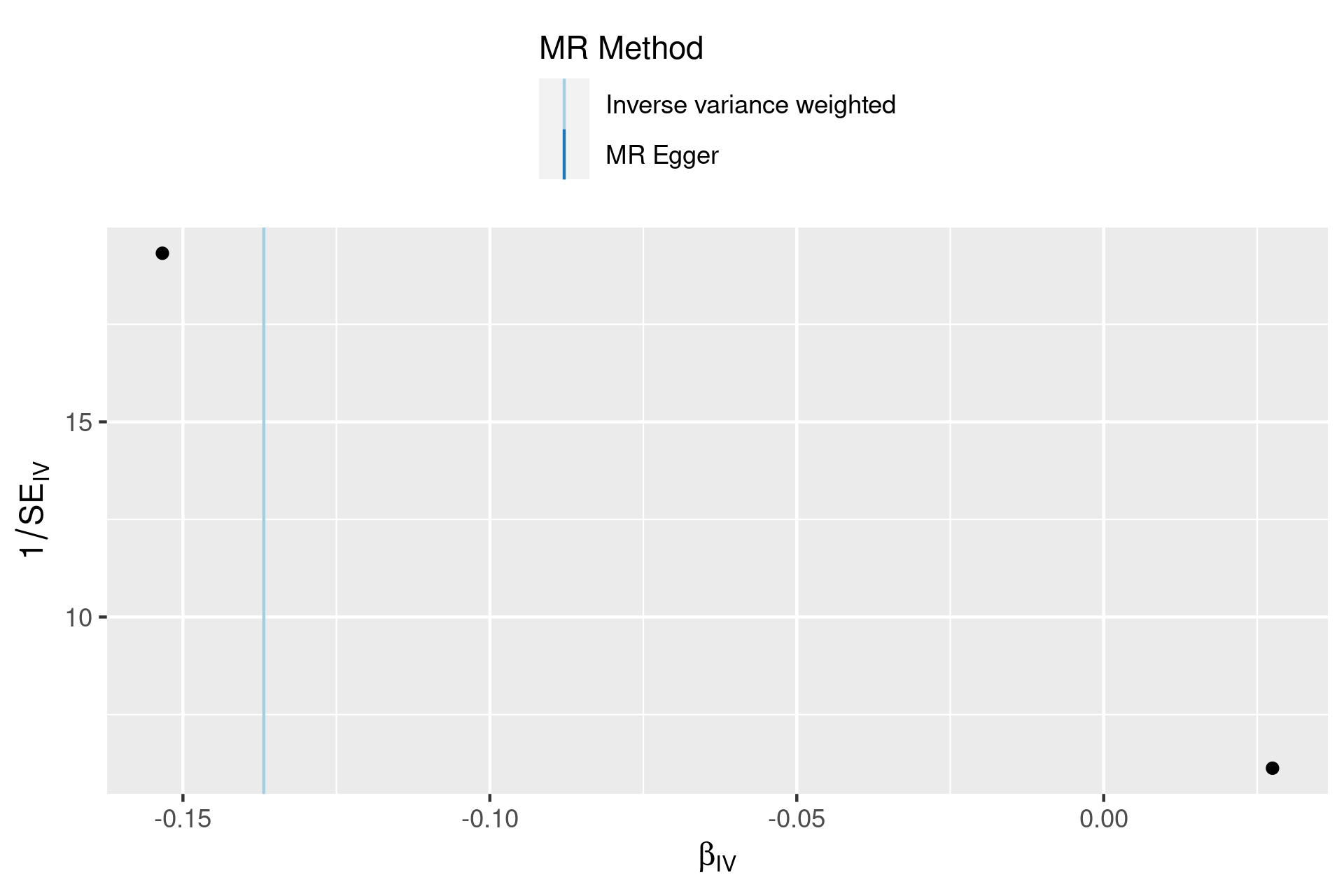


# Supplementary Figure 20. Genetic association of iron with kidney cancer

1) Forest plot


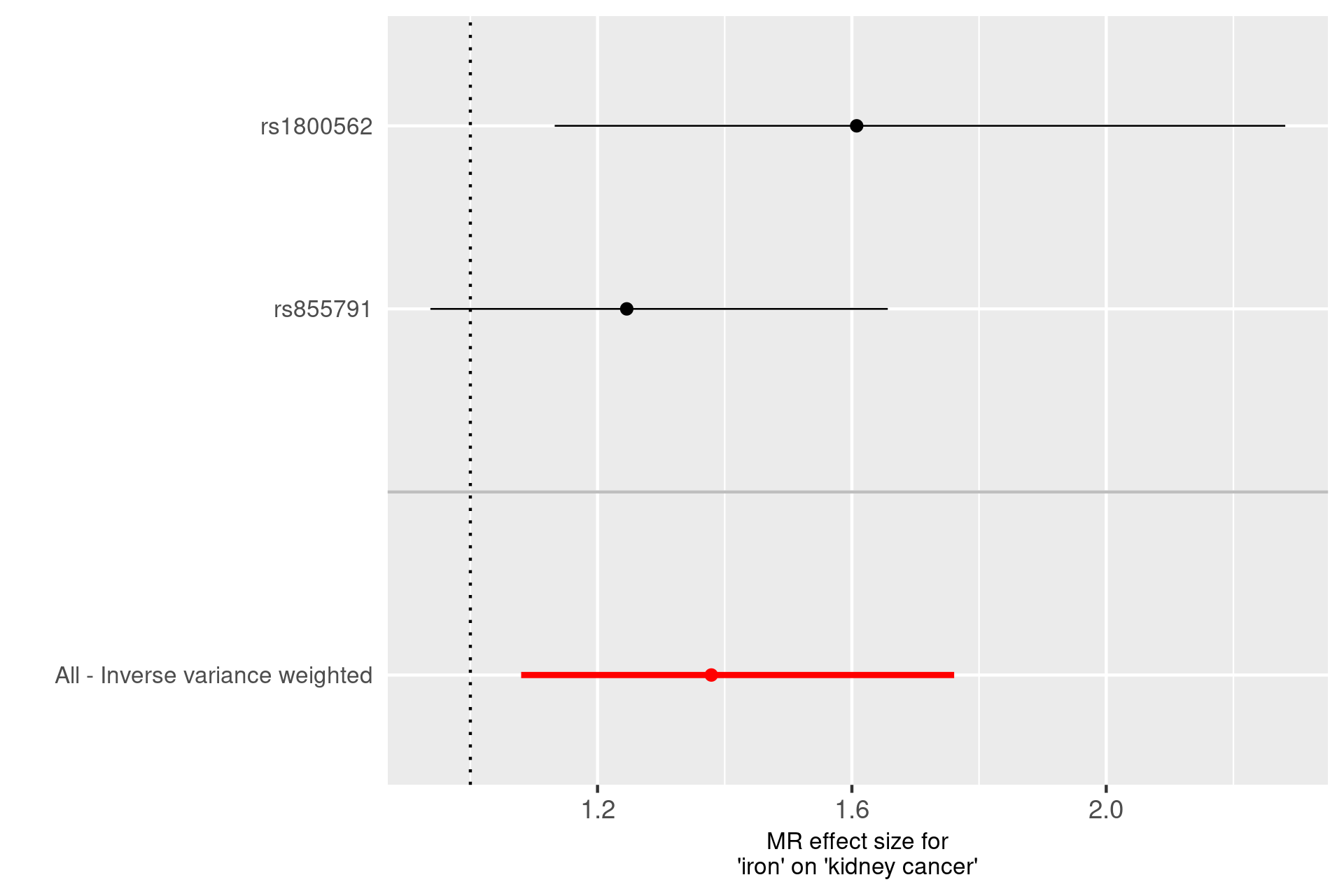


2) Leave-one-out plot

Not available because of small number of SNPs

3) Scatter plot


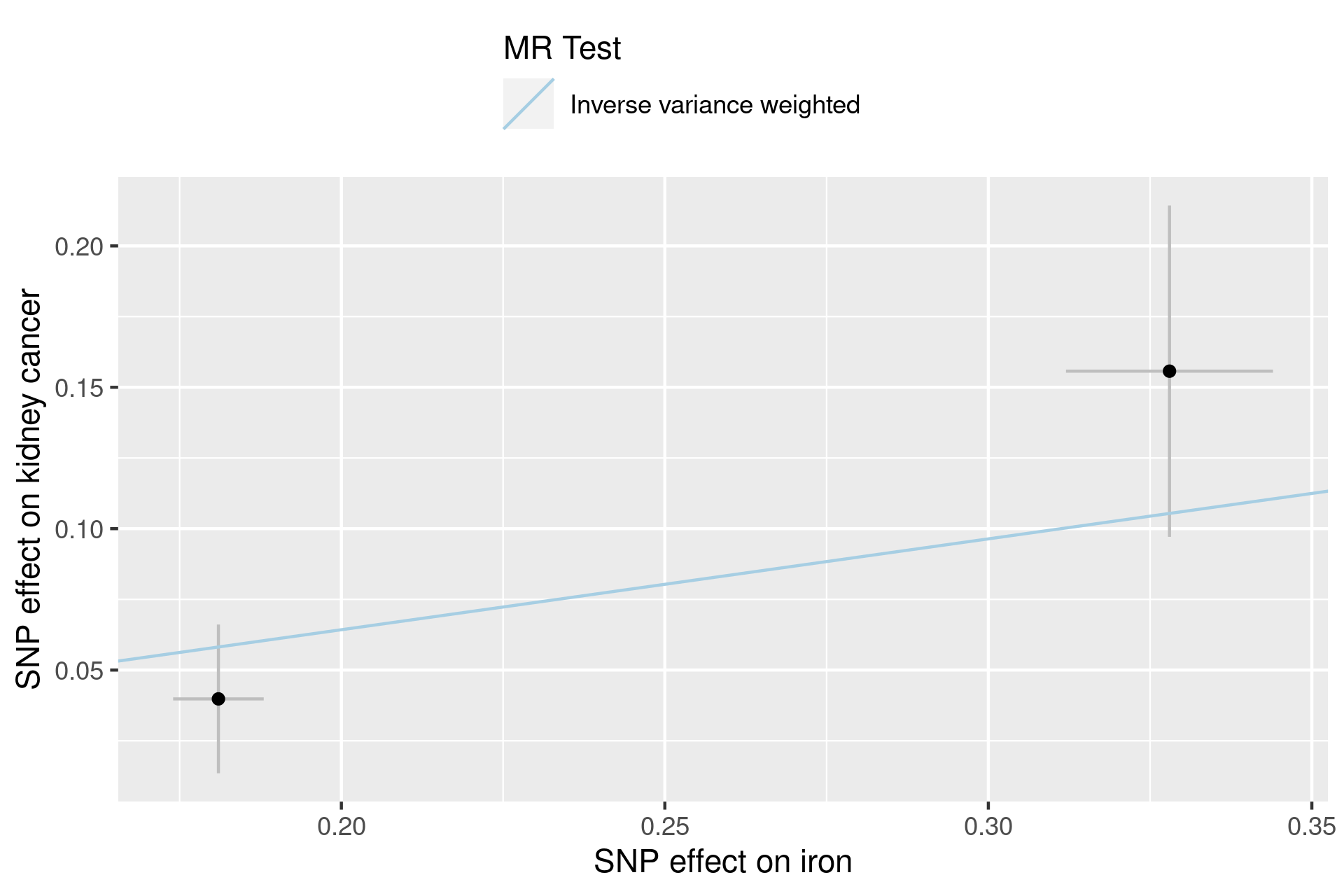


4) Funnel plot


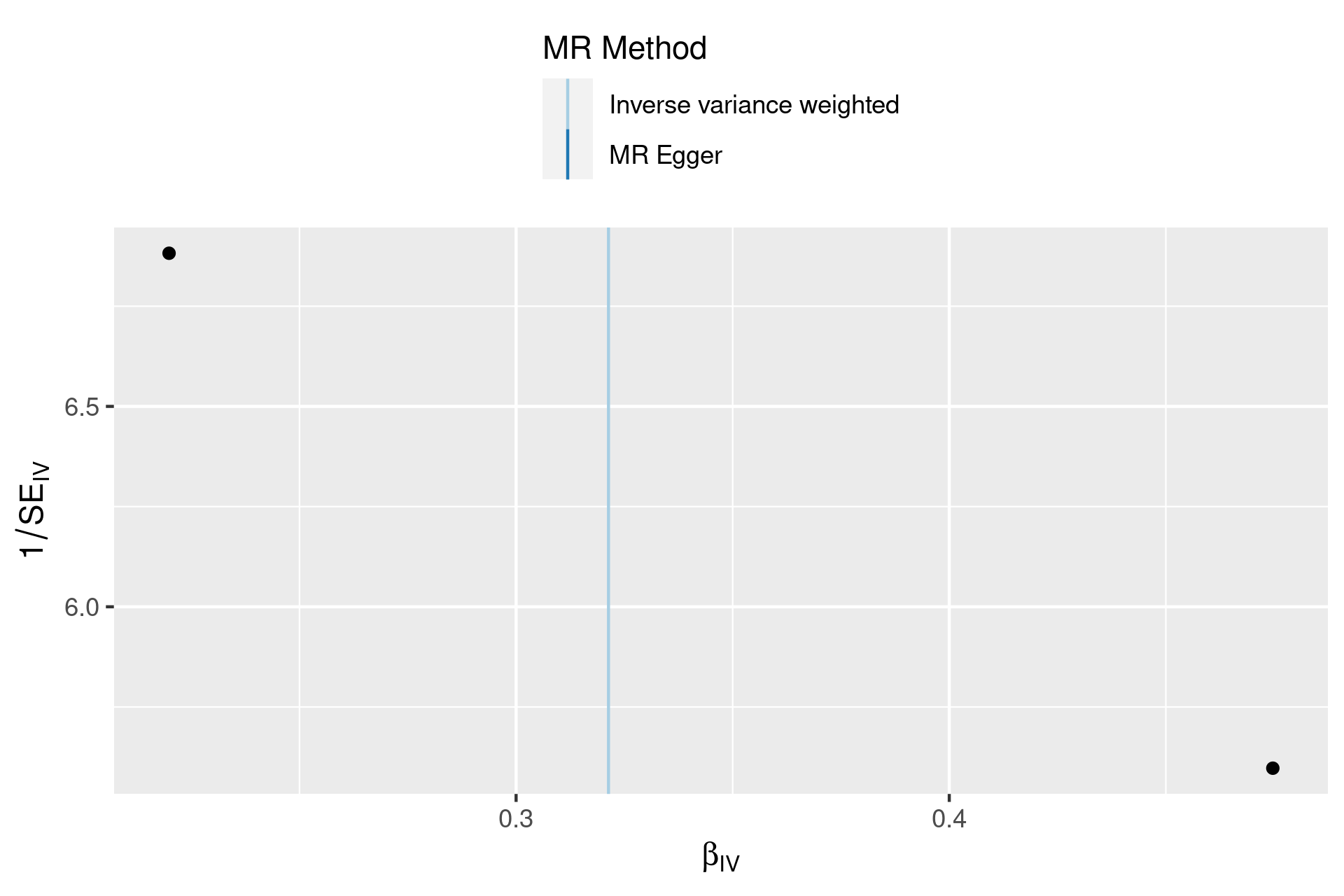


# Supplementary Figure 21. Genetic association of vitamin A1 (retinol) with cervical cancer

1) Forest plot


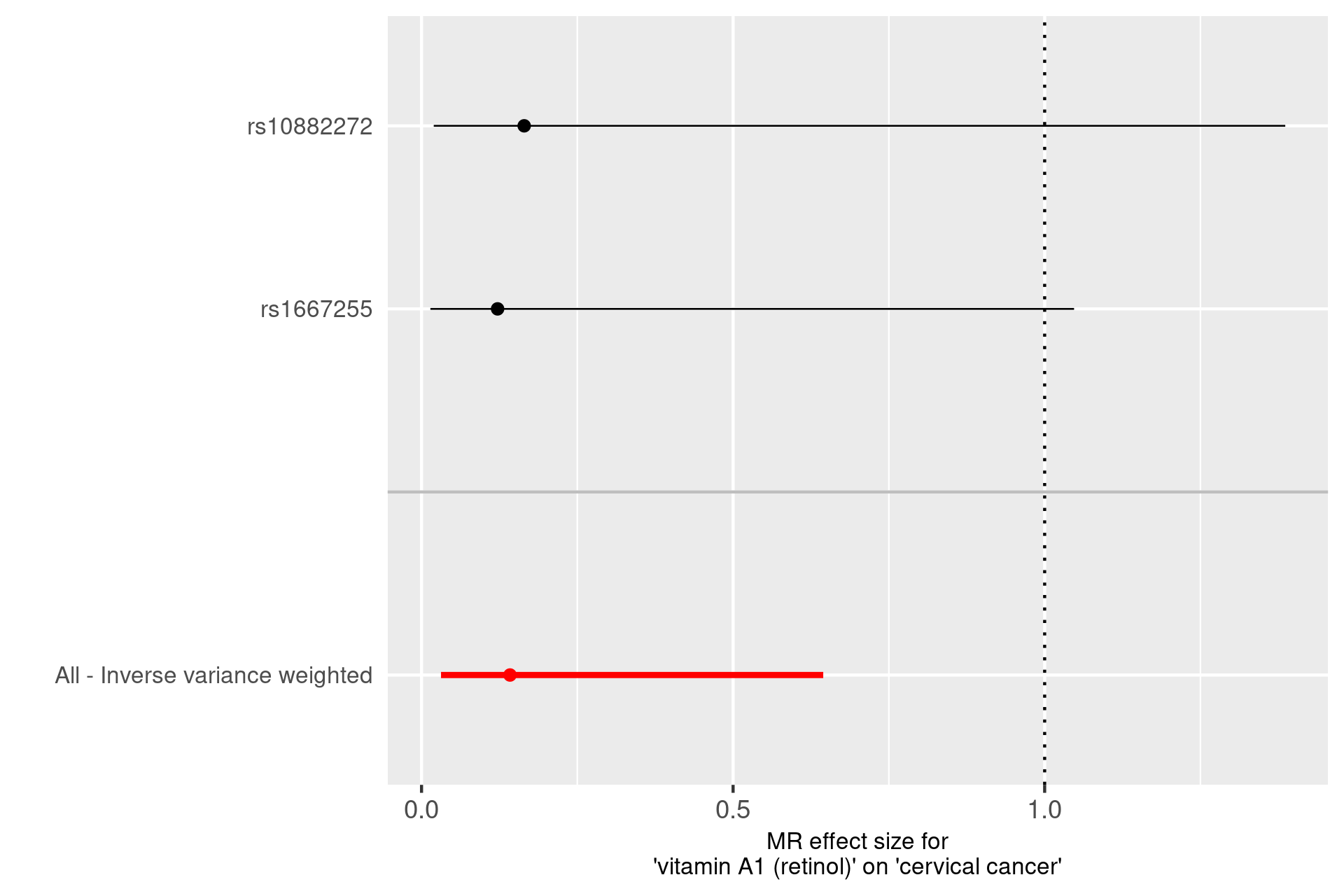


2) Leave-one-out plot

Not available because of small number of SNPs

3) Scatter plot


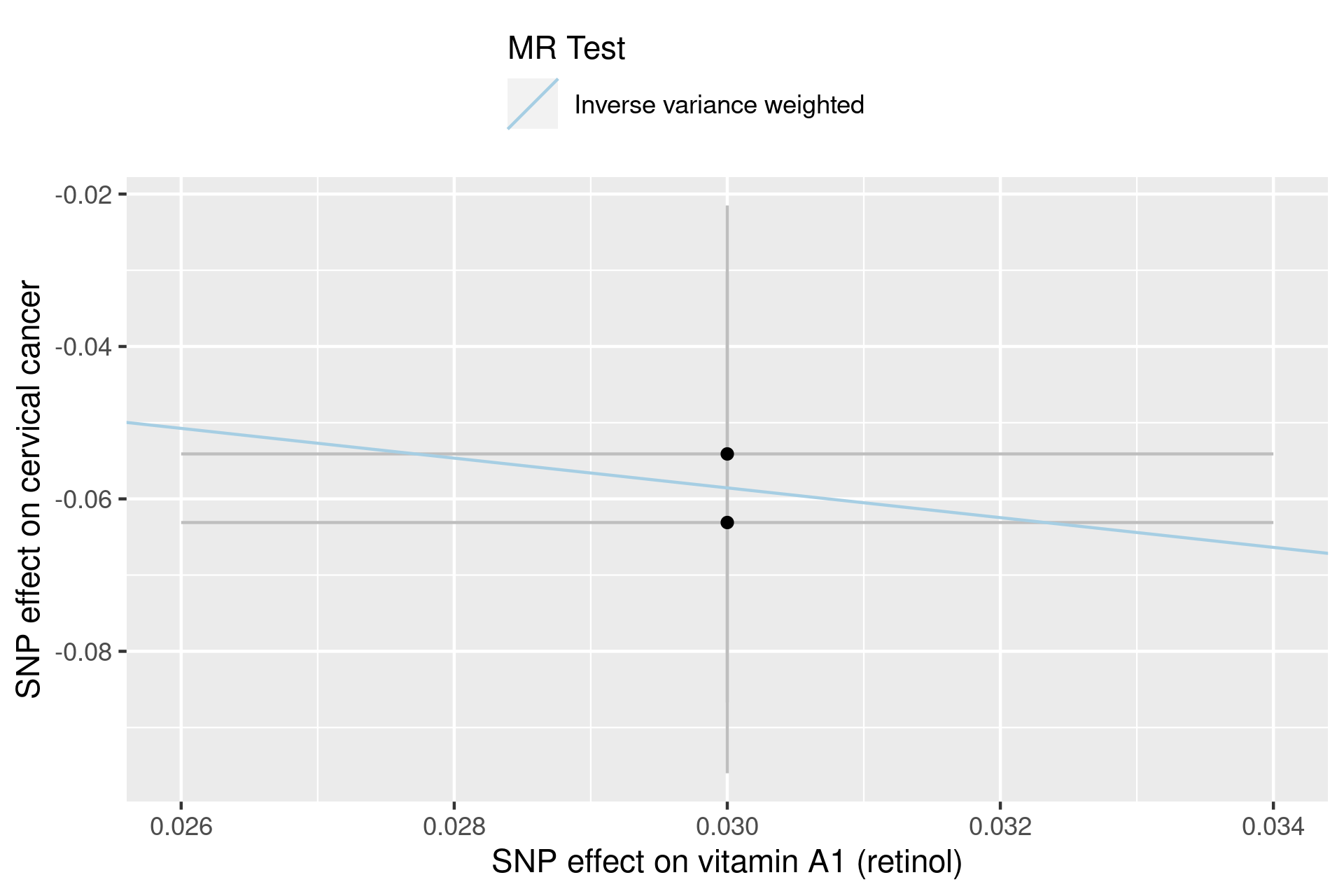


4) Funnel plot


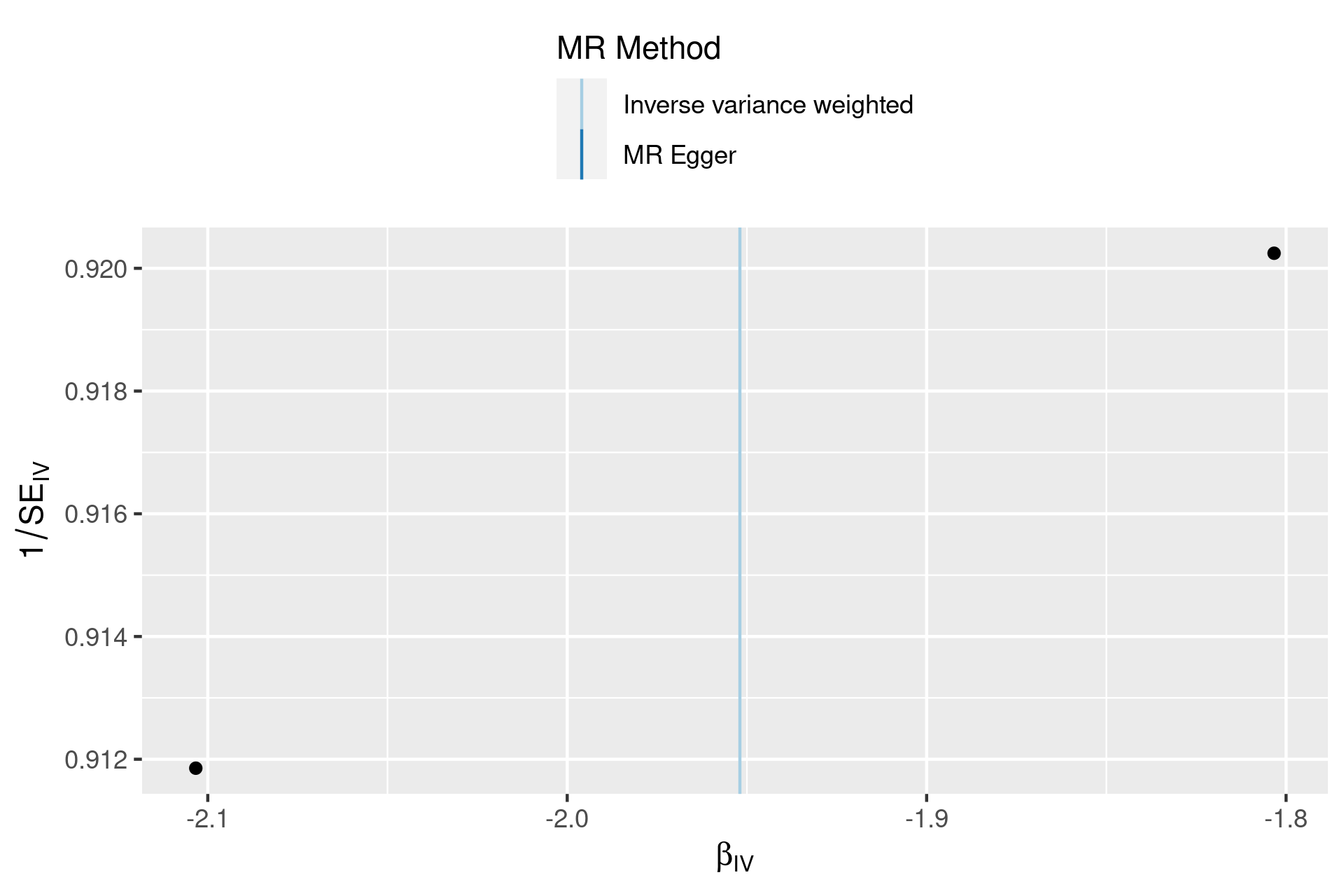


# Supplementary Figure 22. Genetic association of iron with colorectal cancer

1) Forest plot


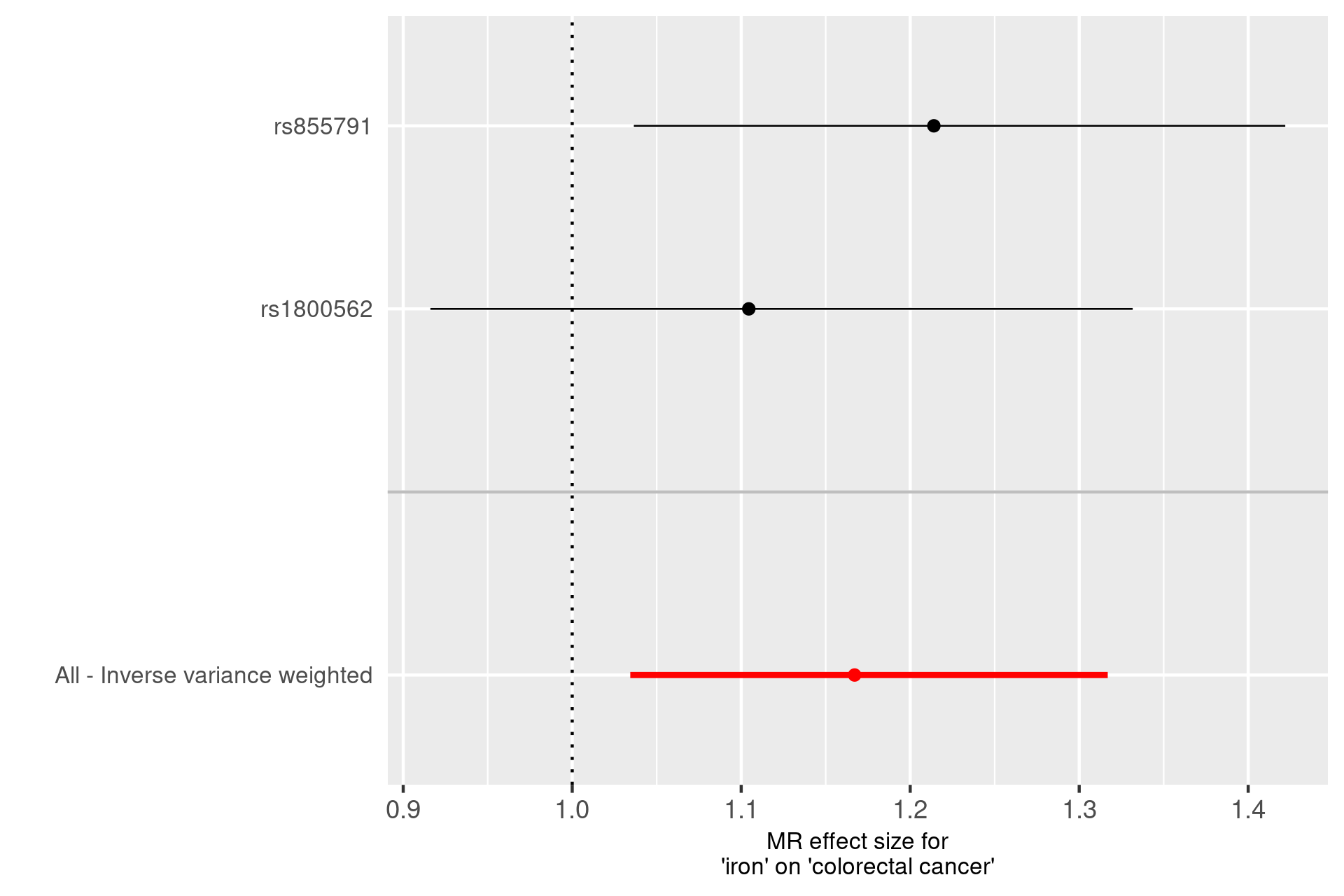


2) Leave-one-out plot

Not available because of small number of SNPs

3) Scatter plot


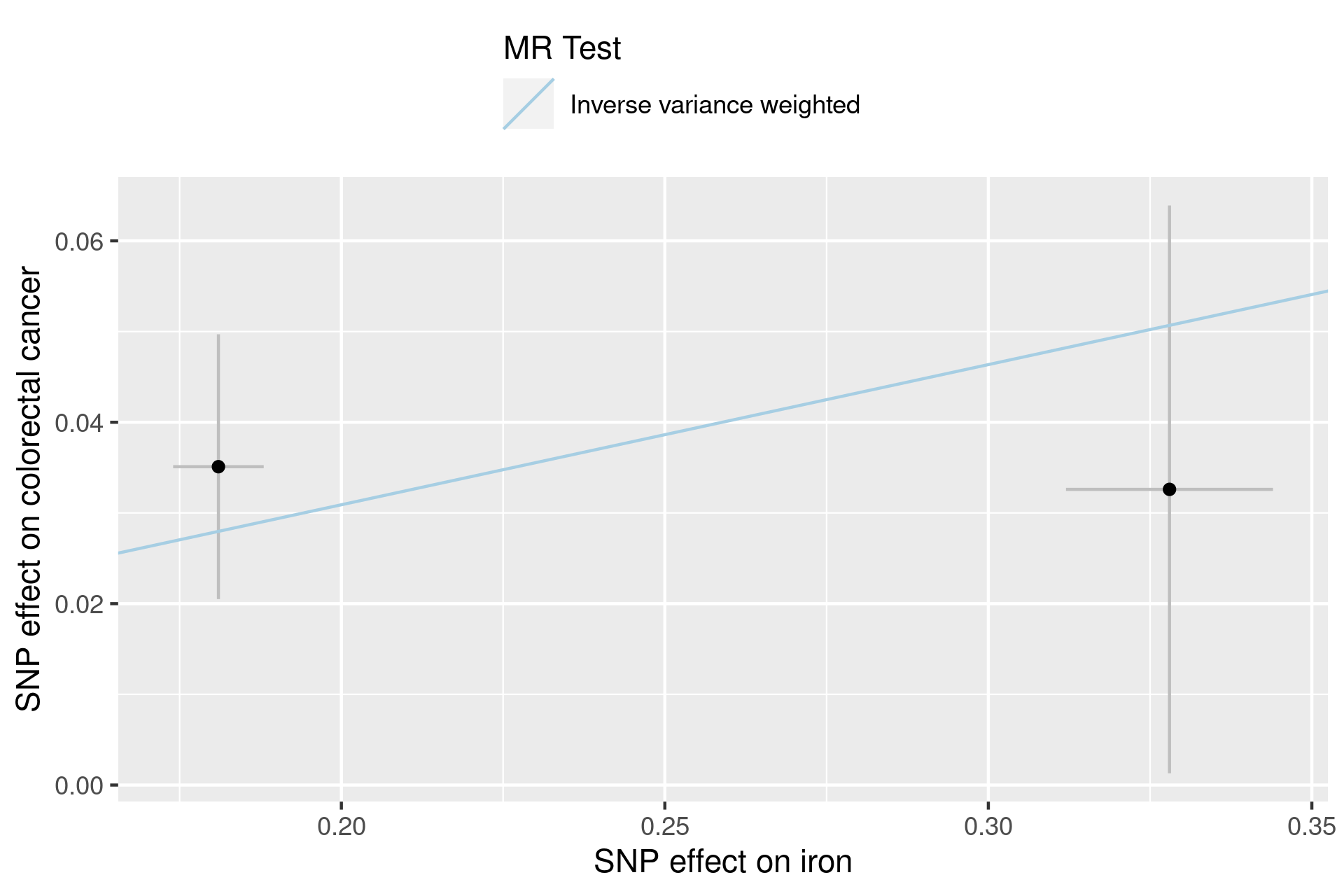


4) Funnel plot


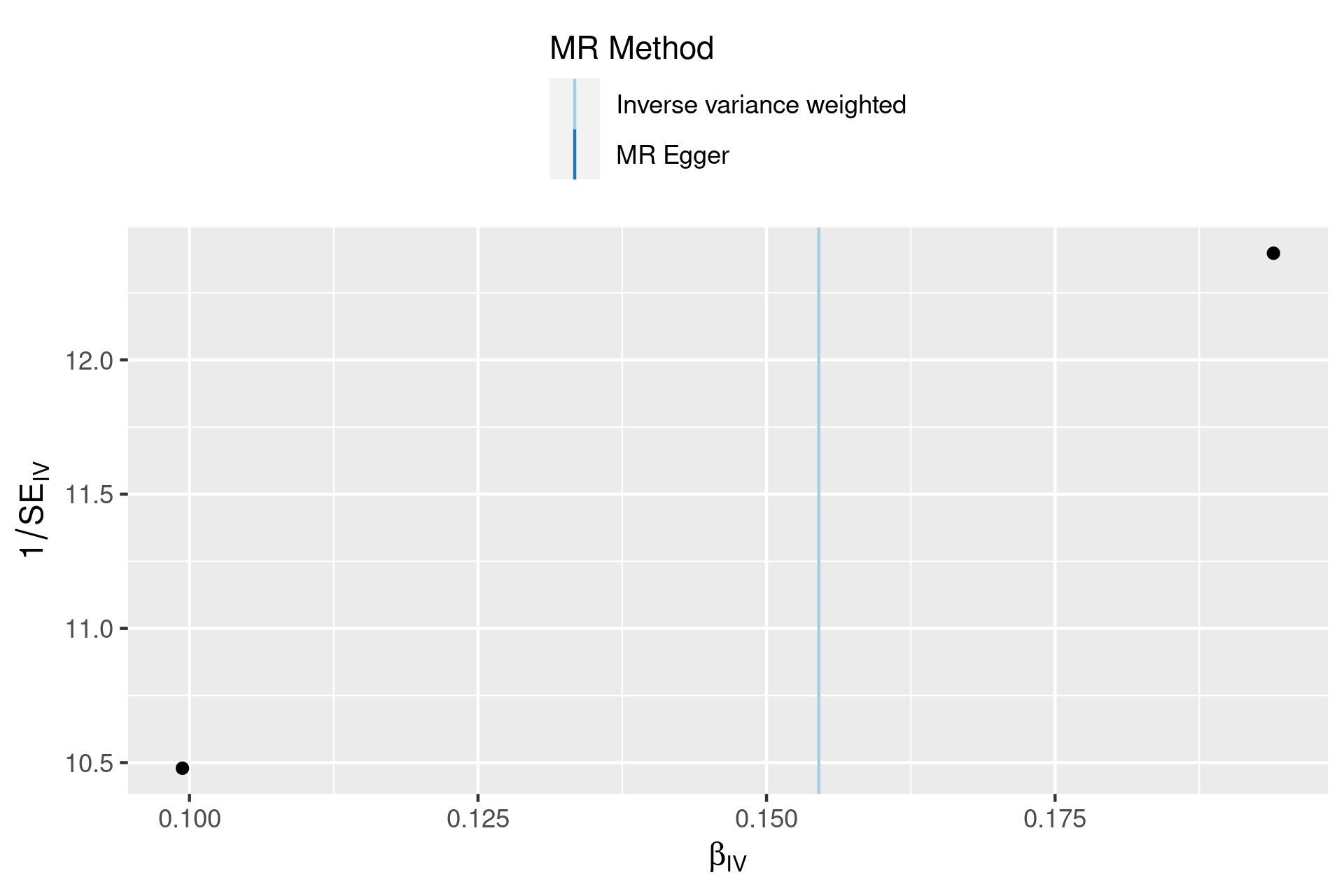


# Supplementary Figure 23. Genetic association of phosphorus with uterine cancer

1) Forest plot


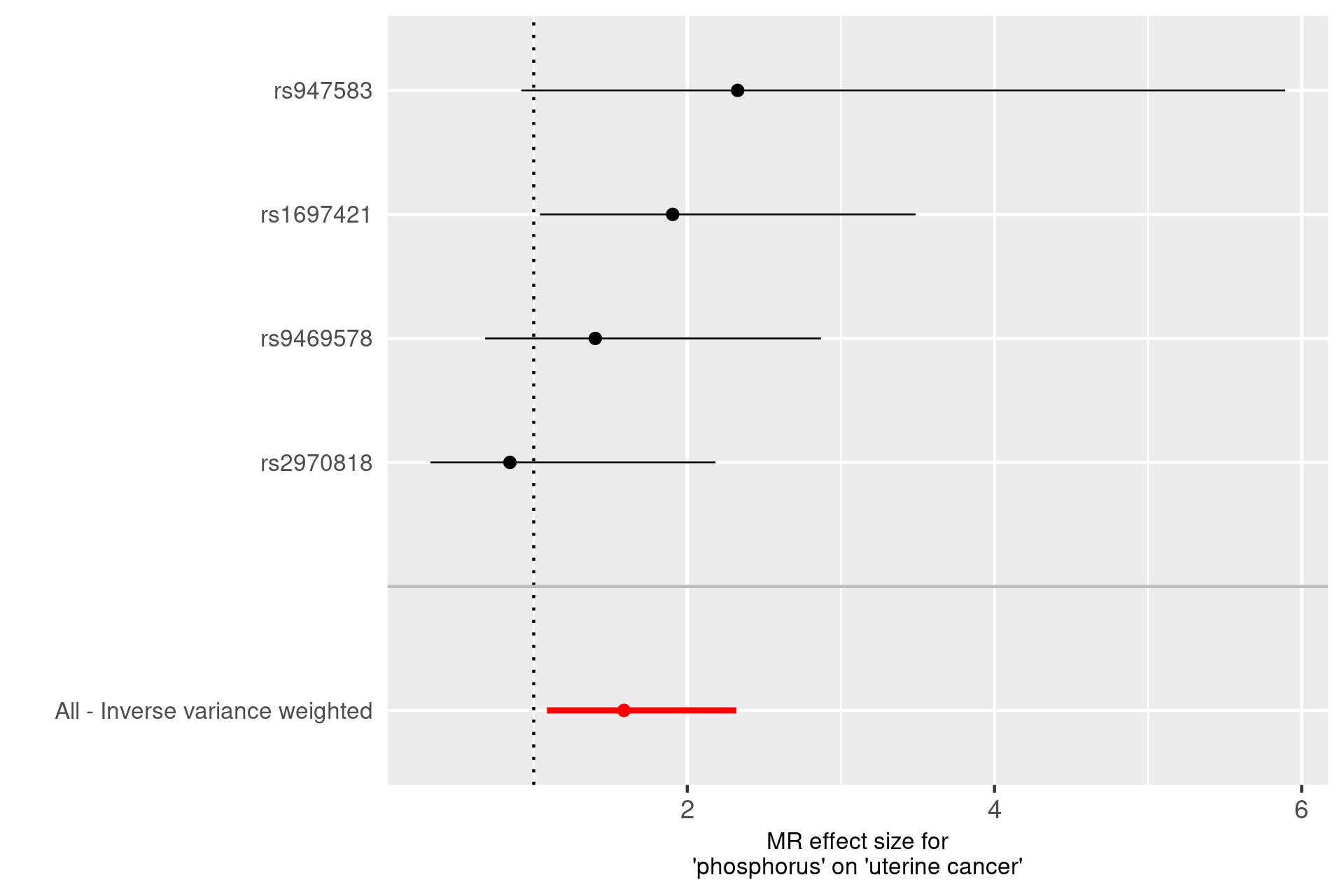


2) Leave-one-out plot


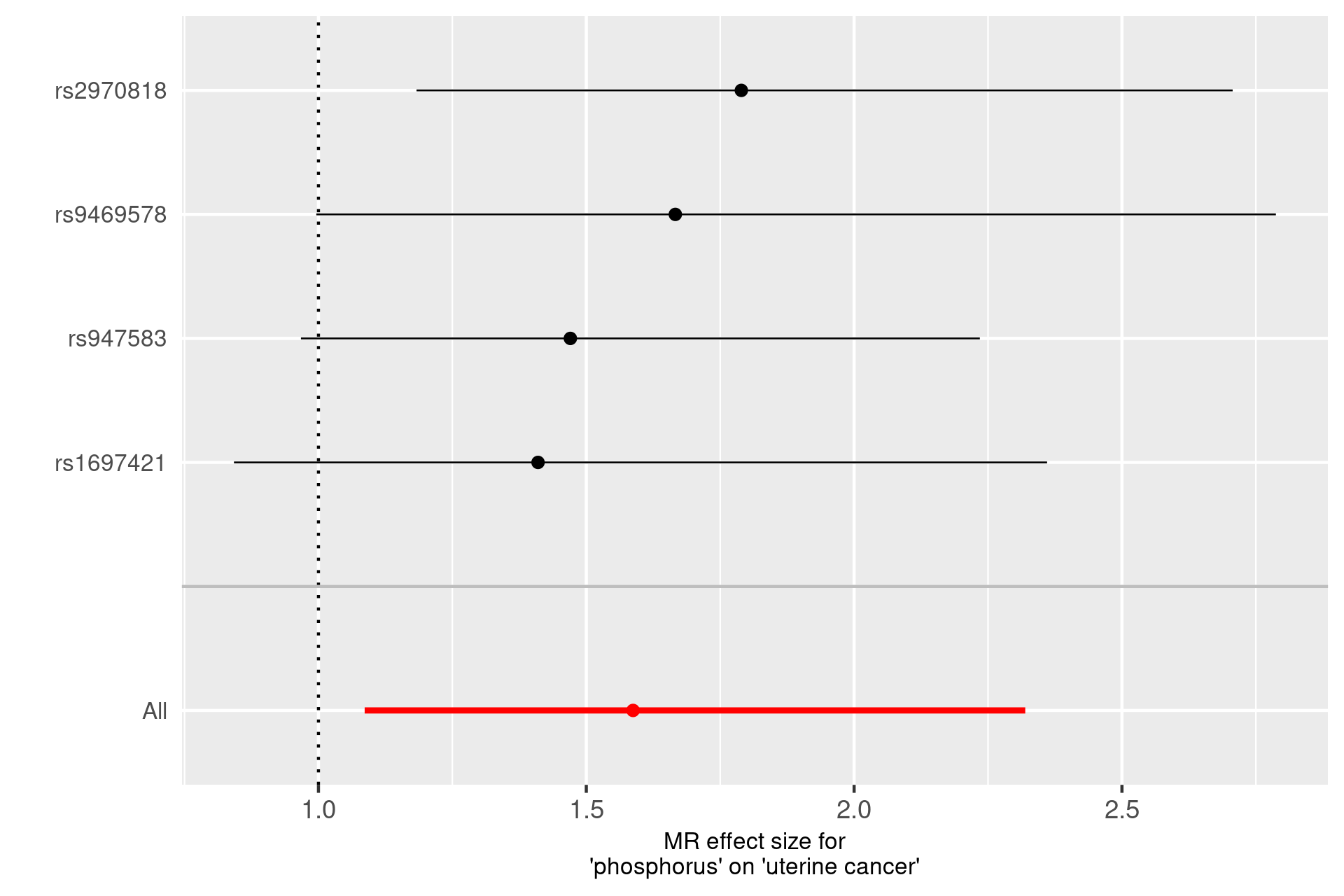


3) Scatter plot


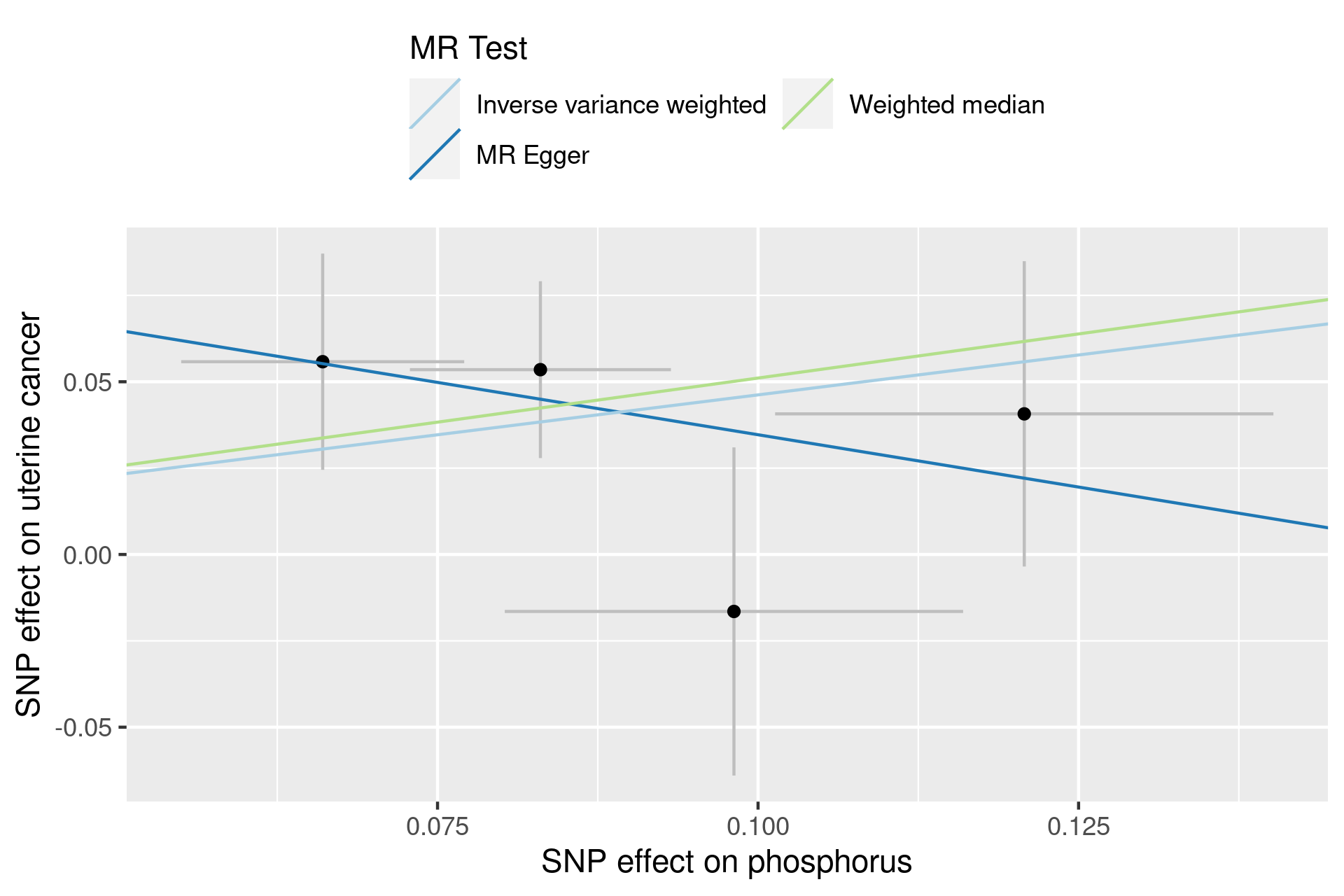


4) Funnel plot


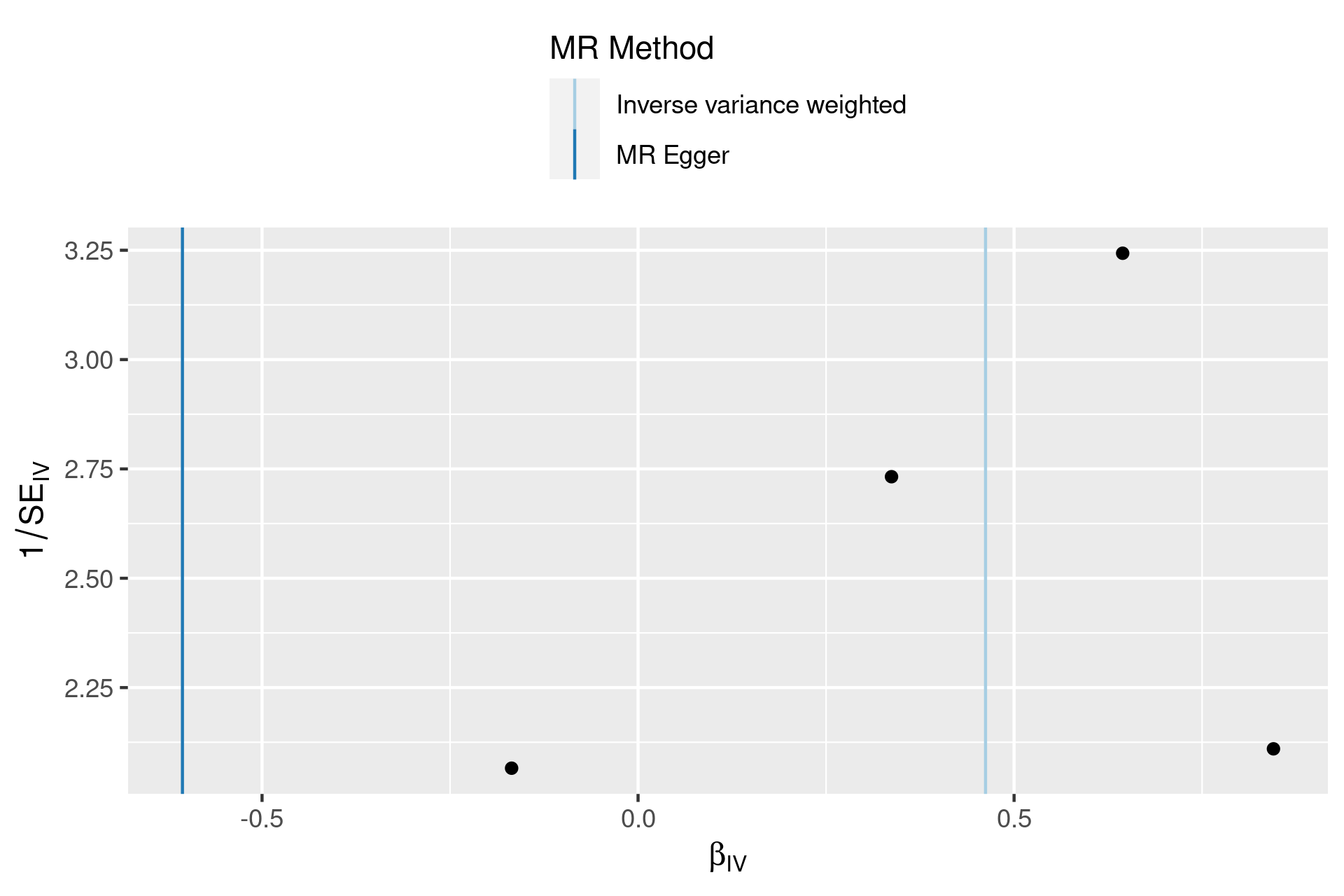


# Supplementary Figure 24. Genetic association of vitamin C with colorectal cancer

1) Forest plot


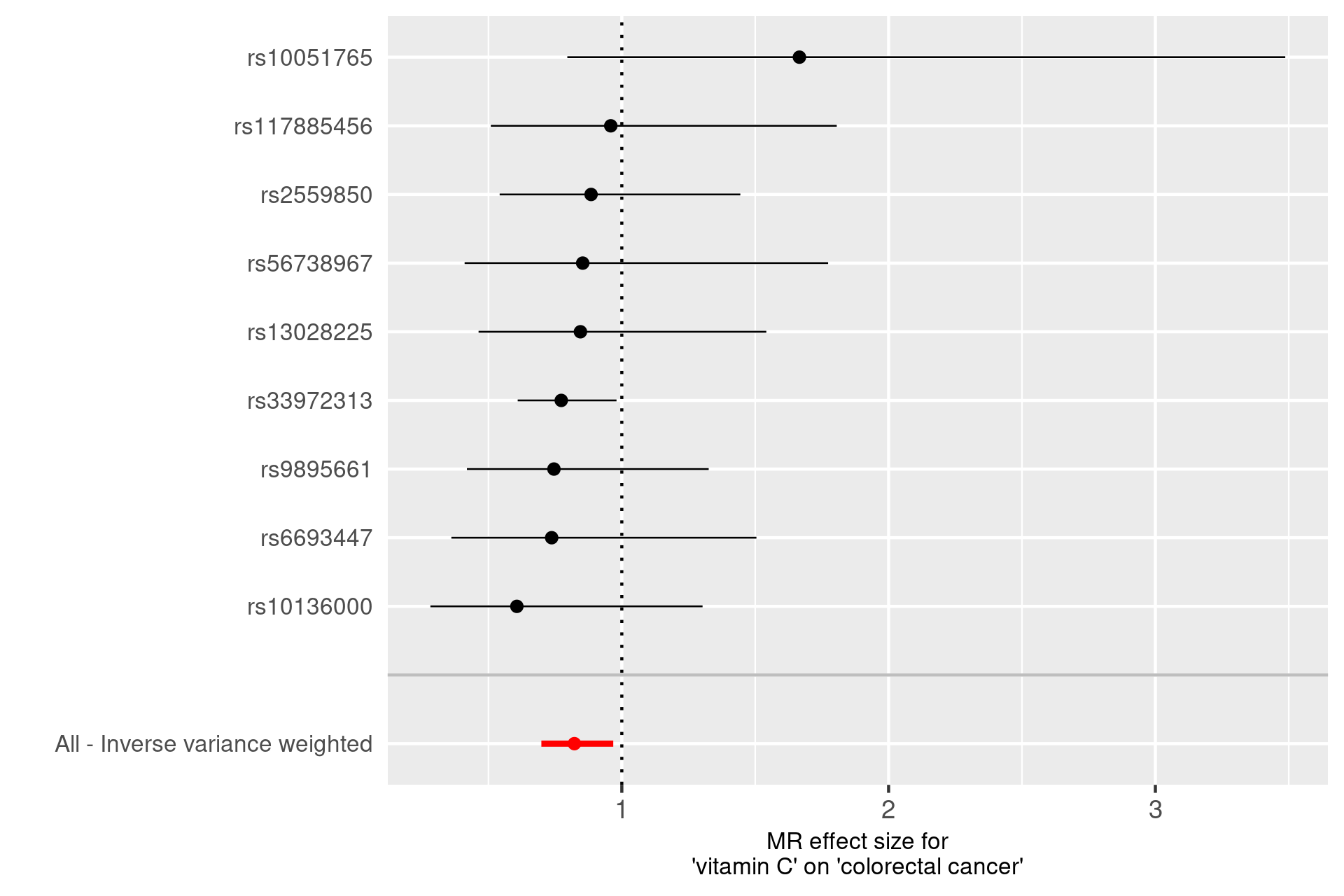


2) Leave-one-out plot


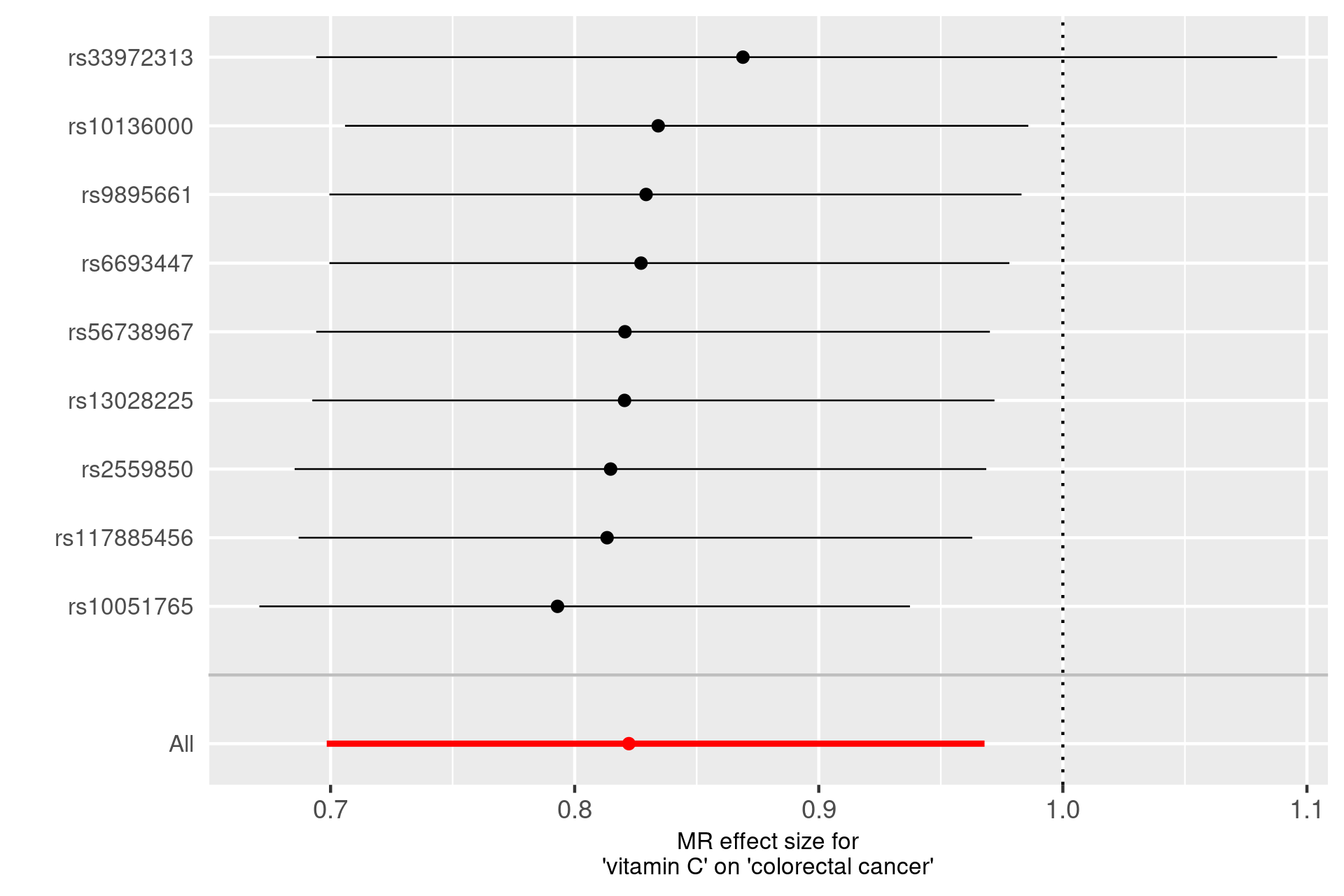


3) Scatter plot


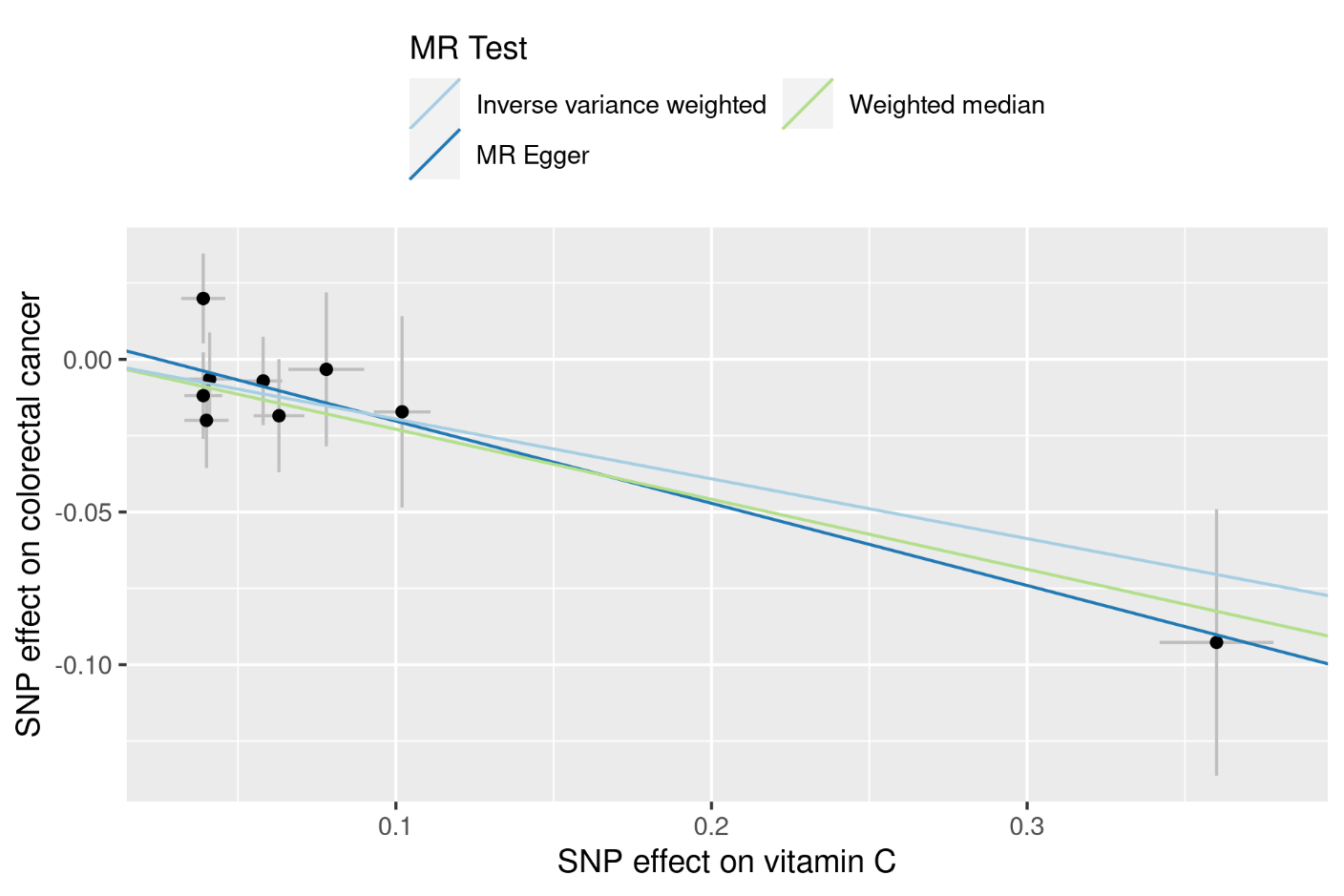


4) Funnel plot


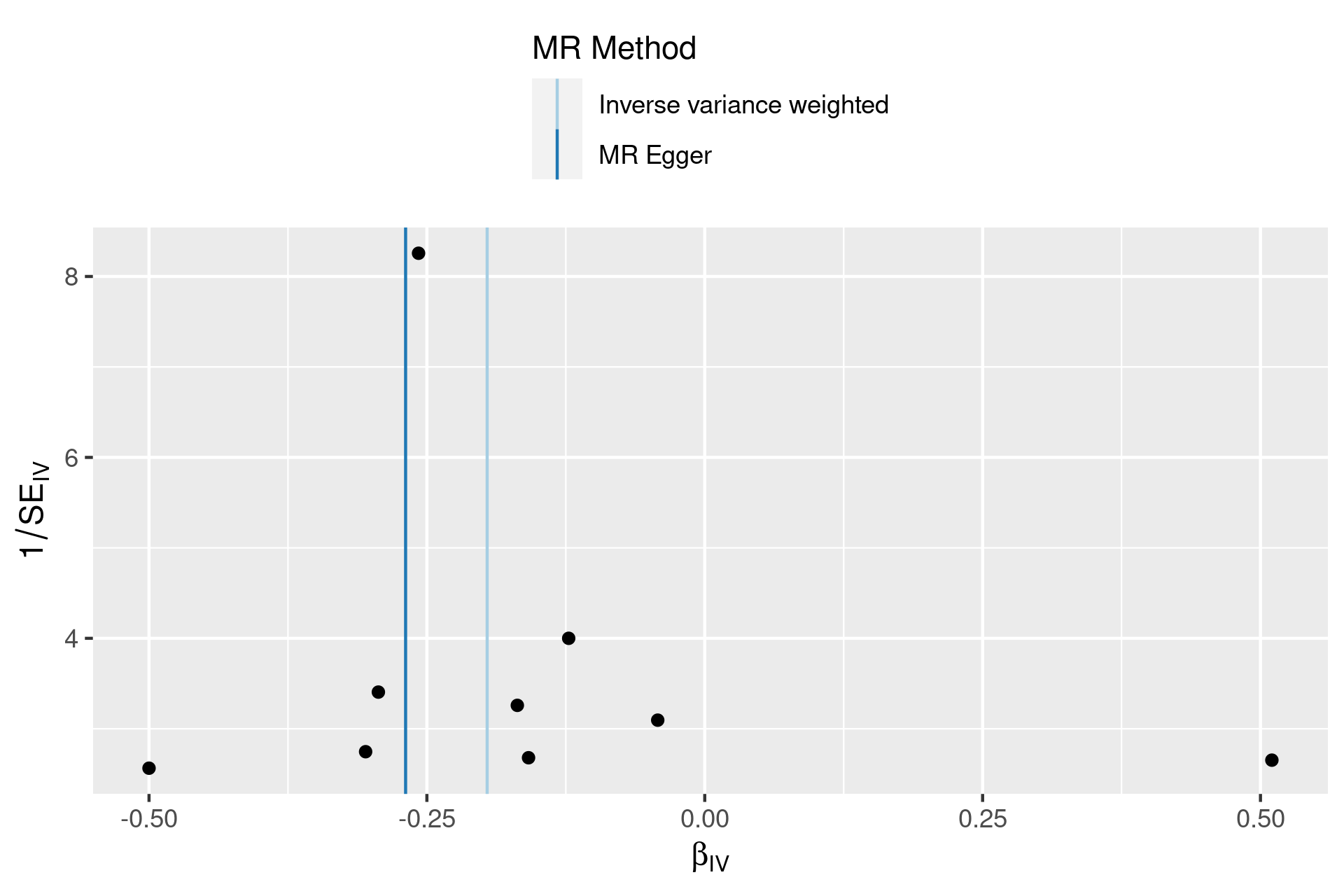


# Supplementary Figure 25. Genetic association of phosphorus with ovarian cancer

1) Forest plot


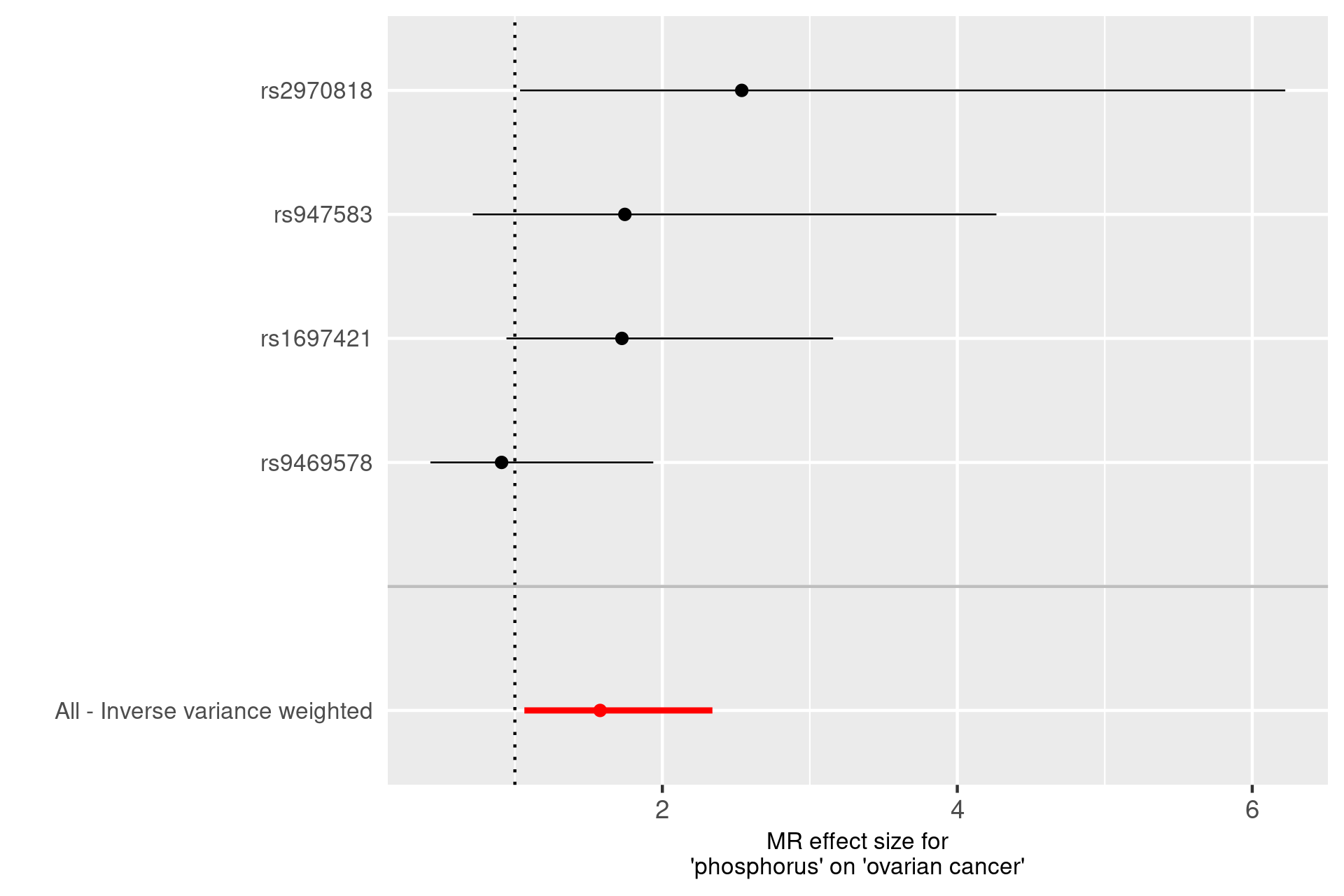


2) Leave-one-out plot


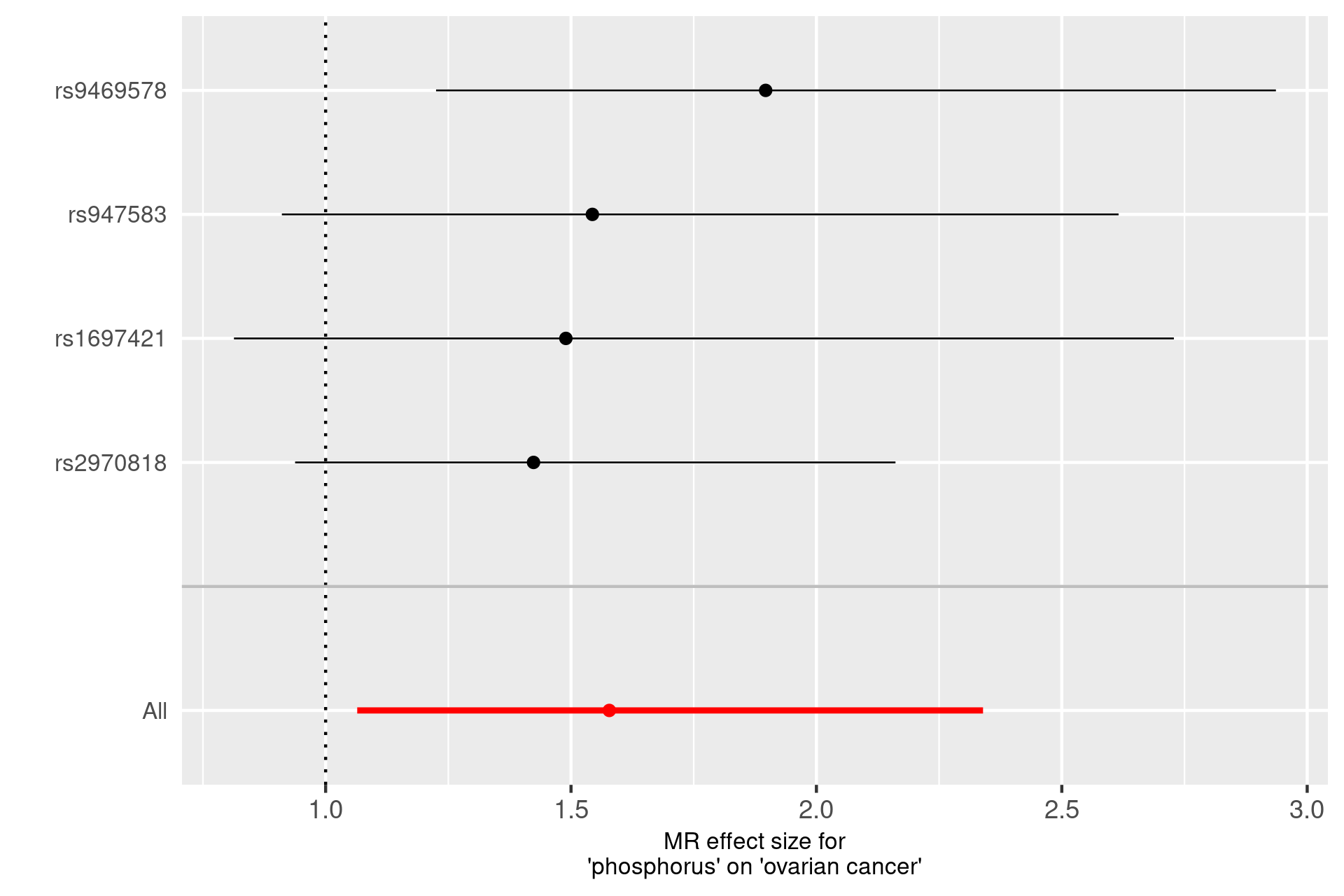


3) Scatter plot


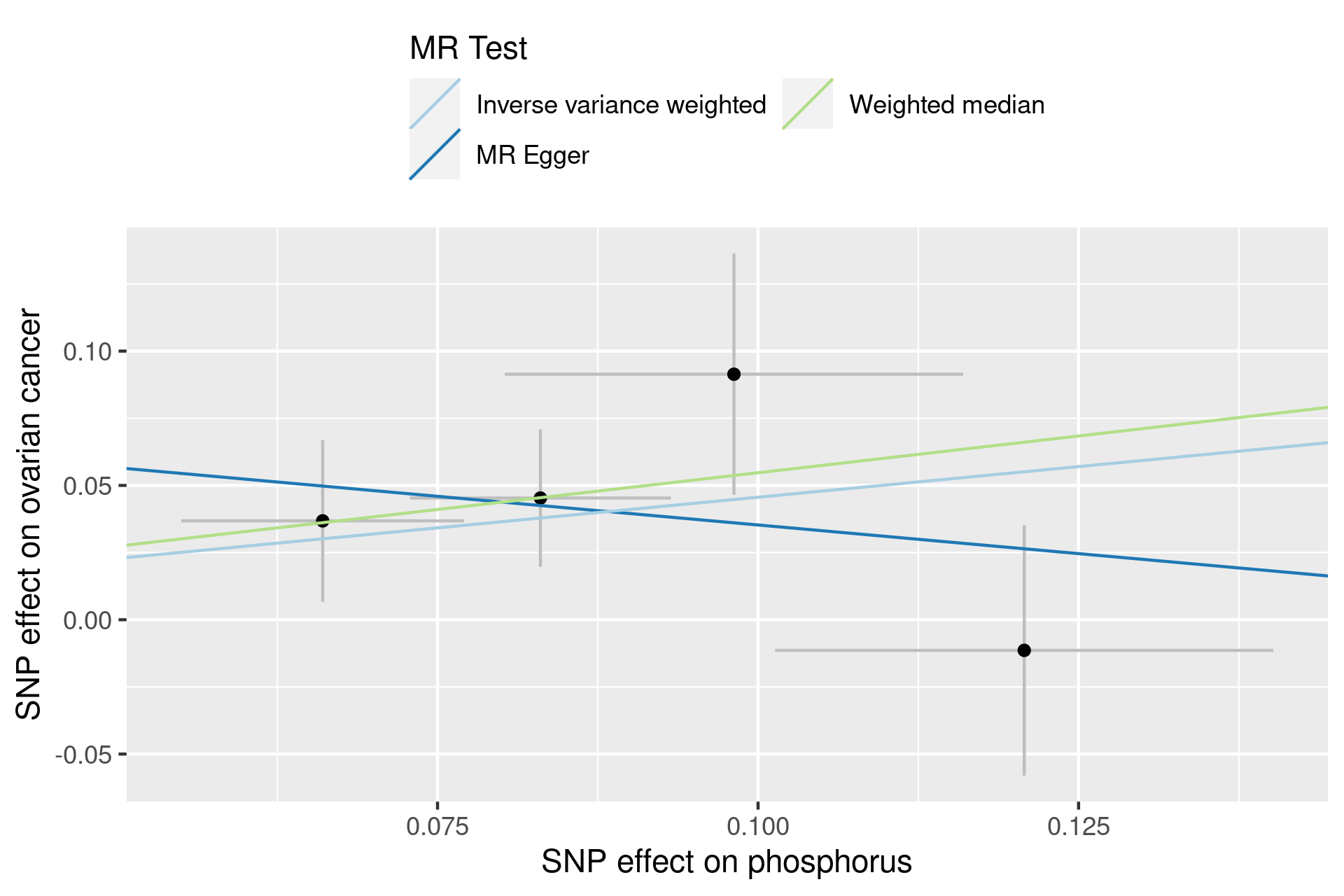


4) Funnel plot


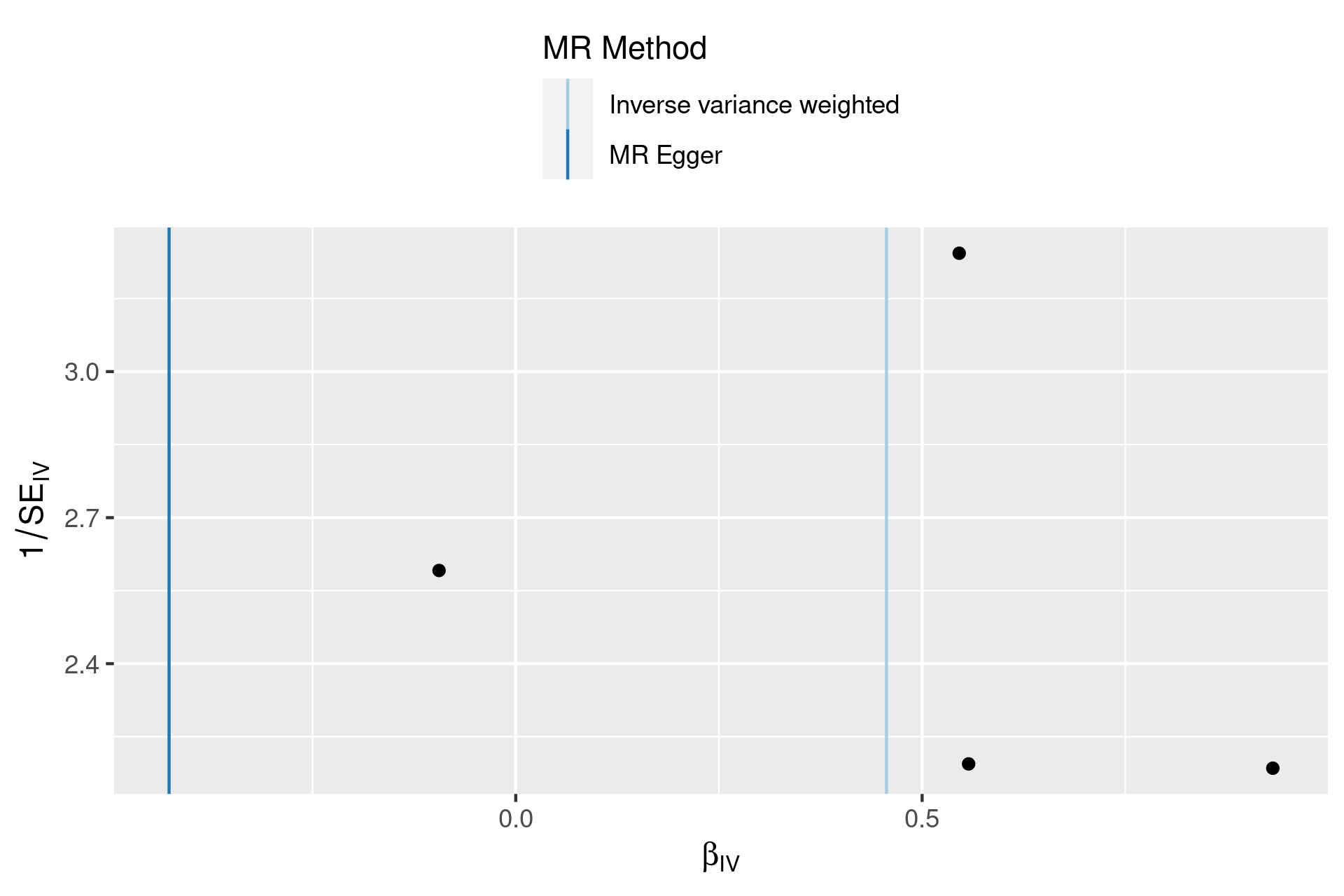


# Supplementary Figure 26. Genetic association of vitamin C with liver cancer

1) Forest plot


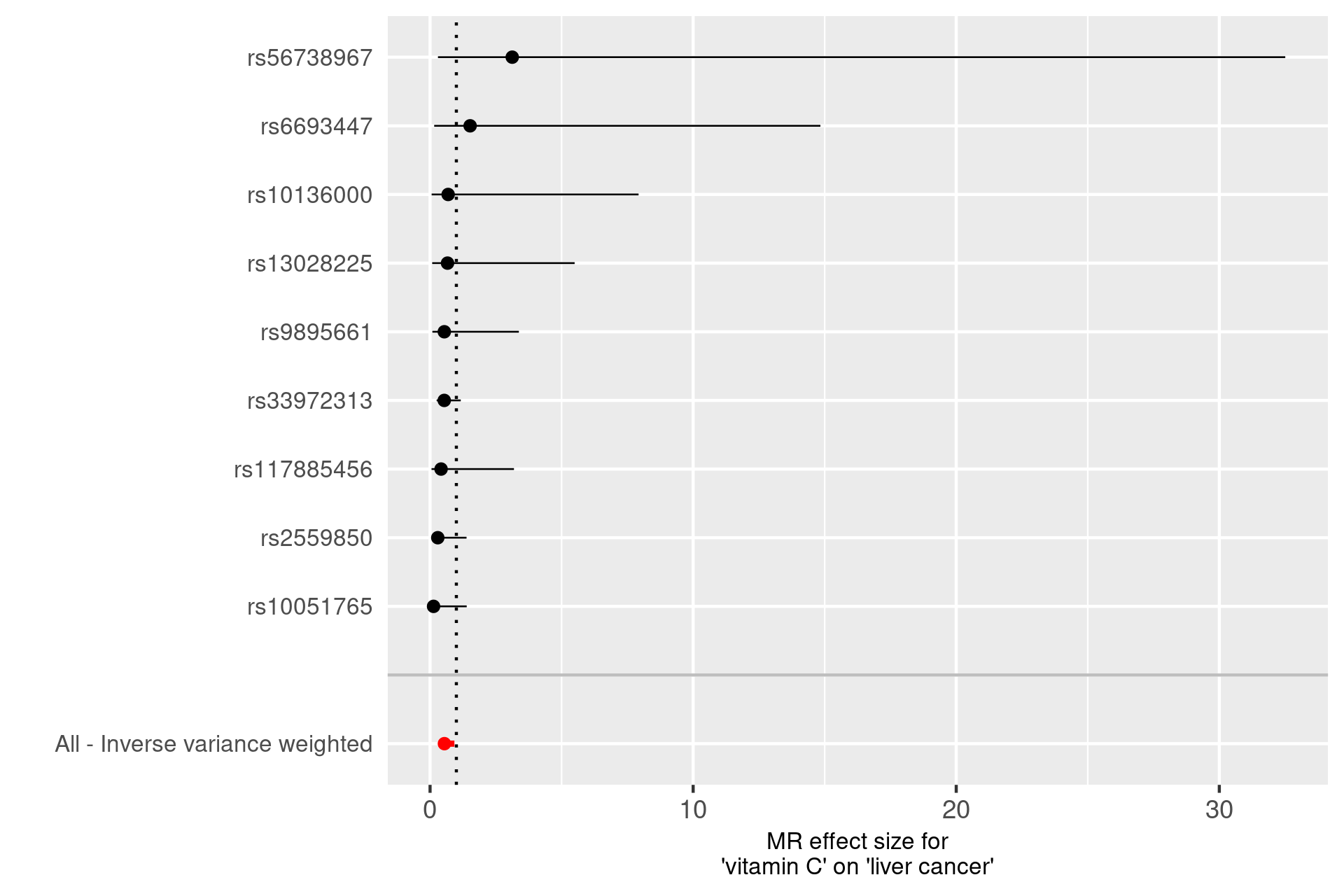


2) Leave-one-out plot


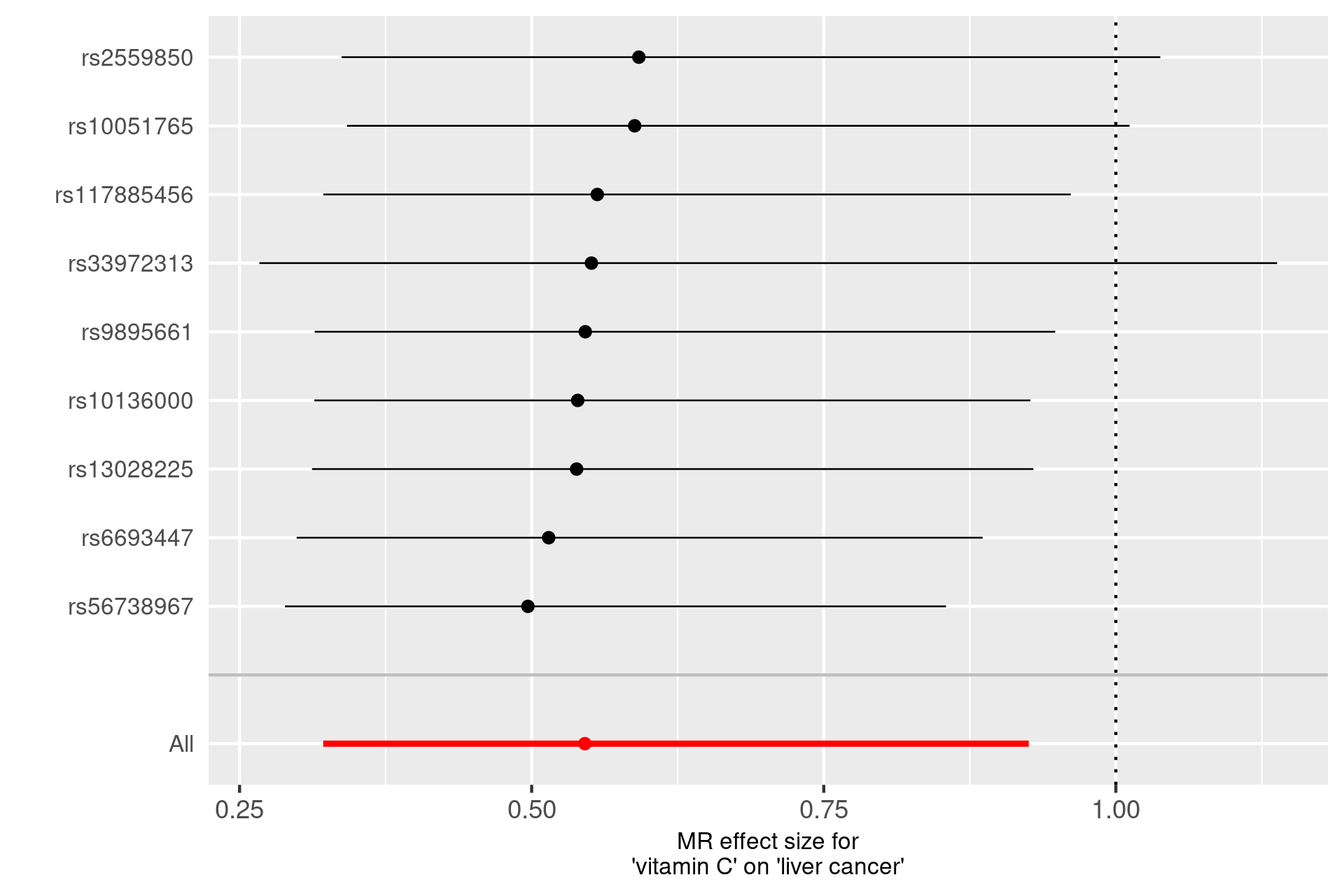


3) Scatter plot


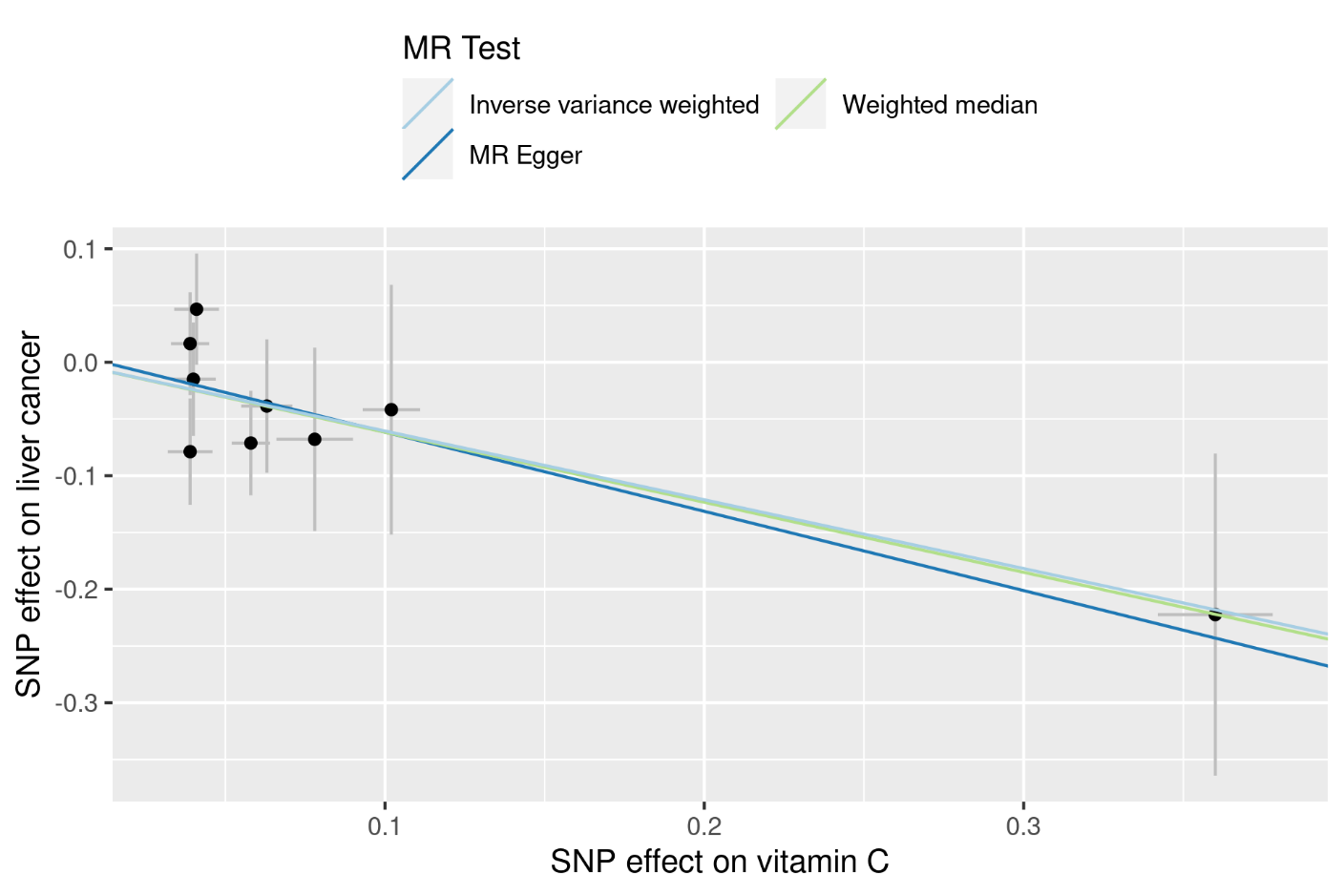


4) Funnel plot


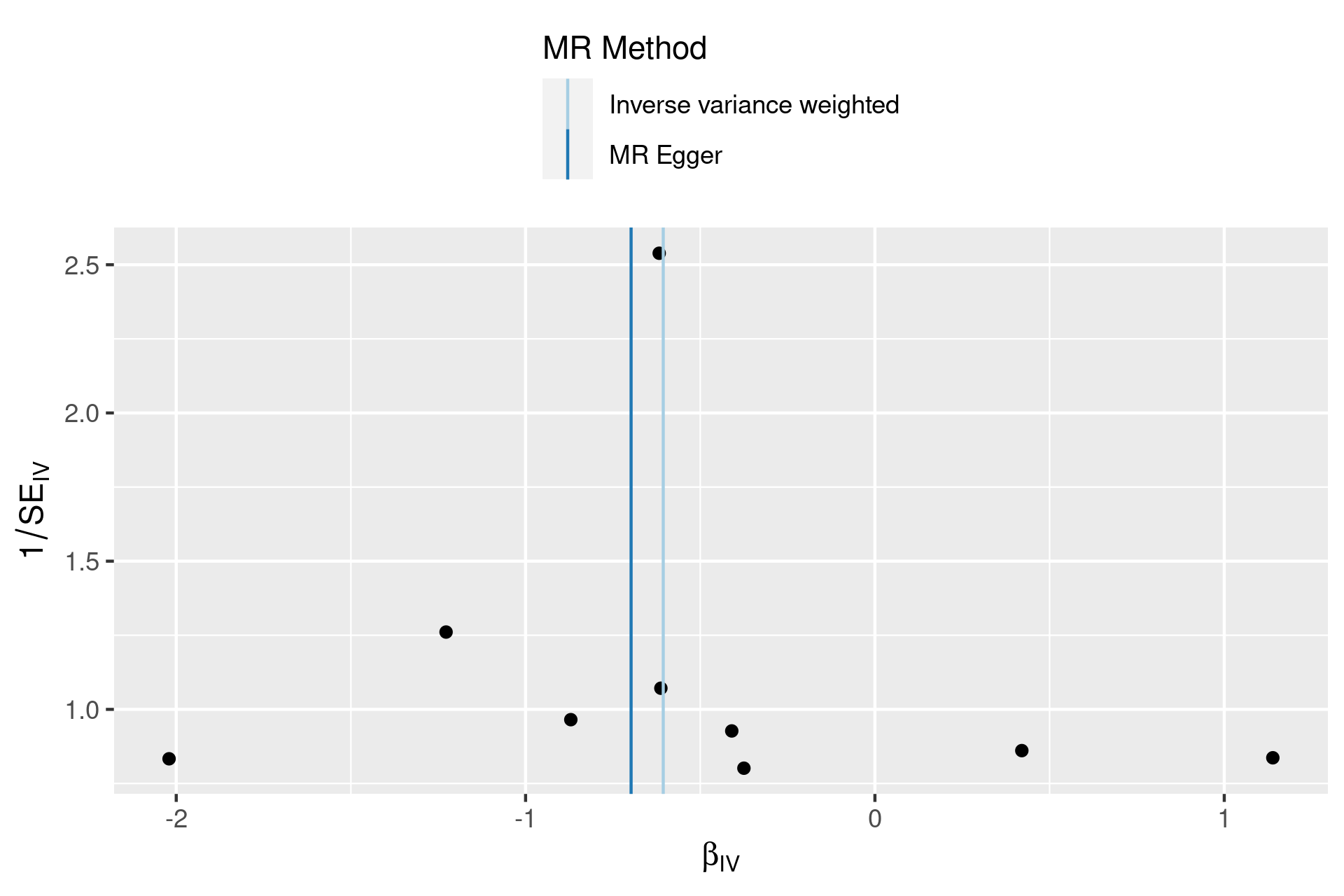


# Supplementary Figure 27. Genetic association of phosphorus with breast cancer

1) Forest plot


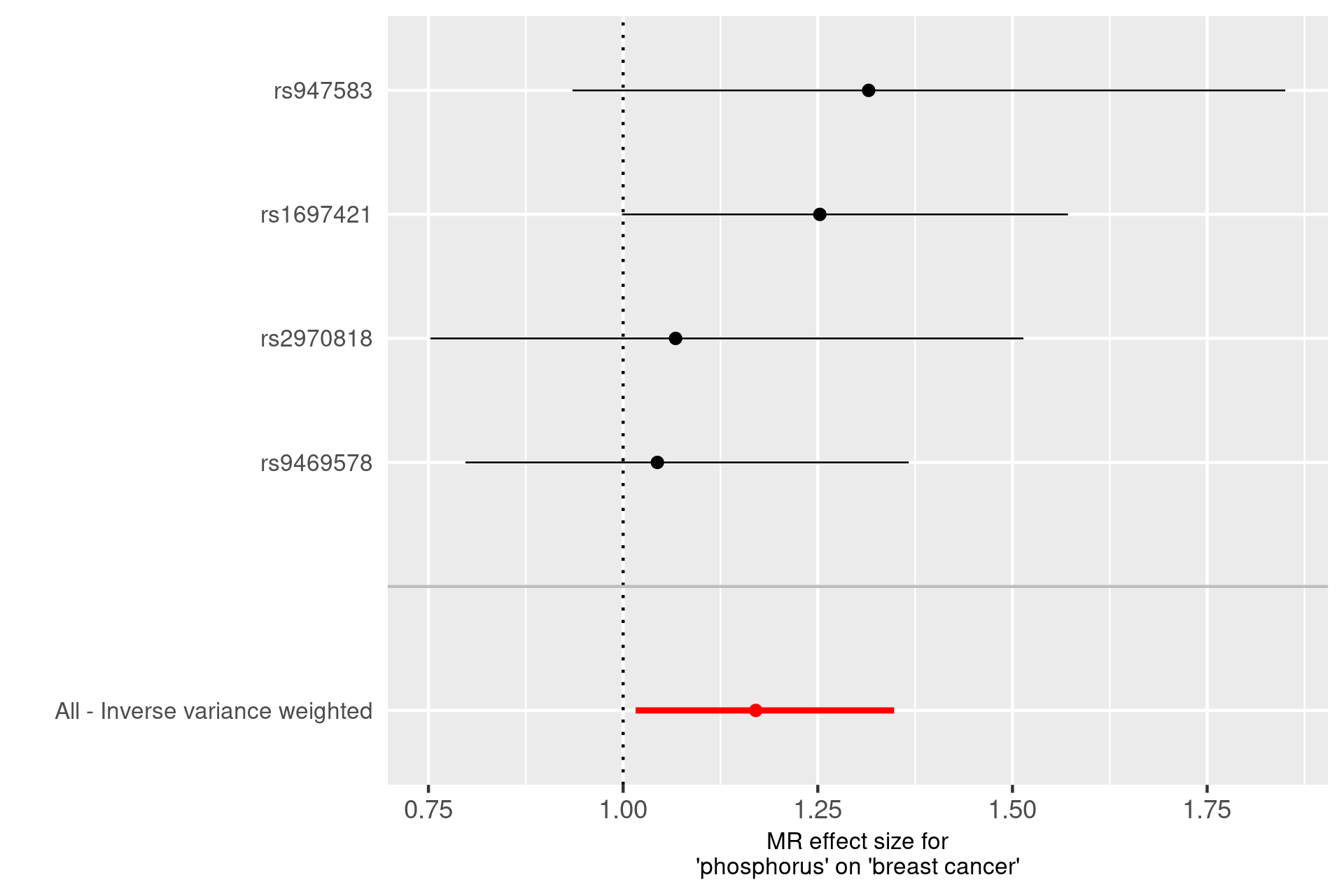


2) Leave-one-out plot


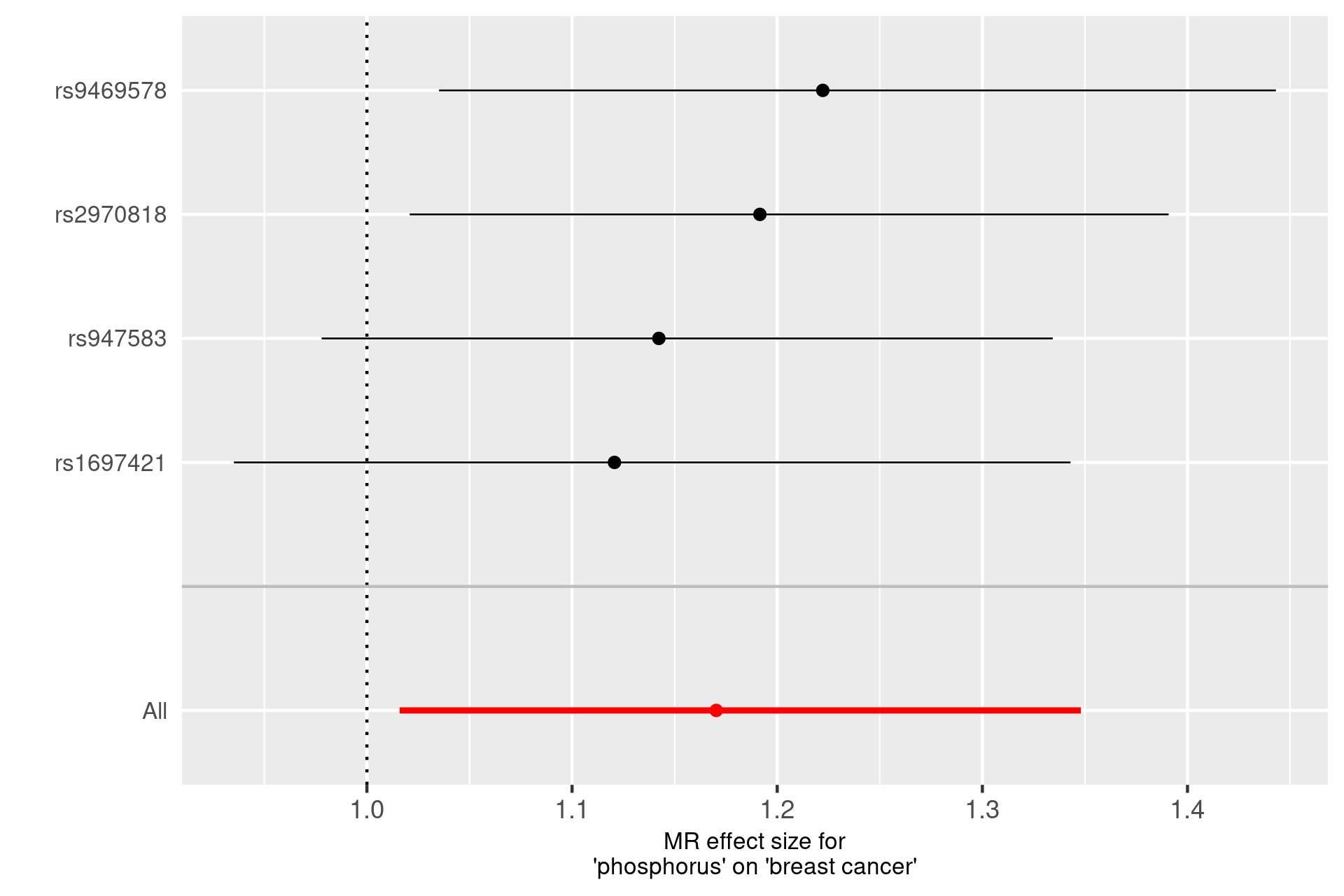


3) Scatter plot


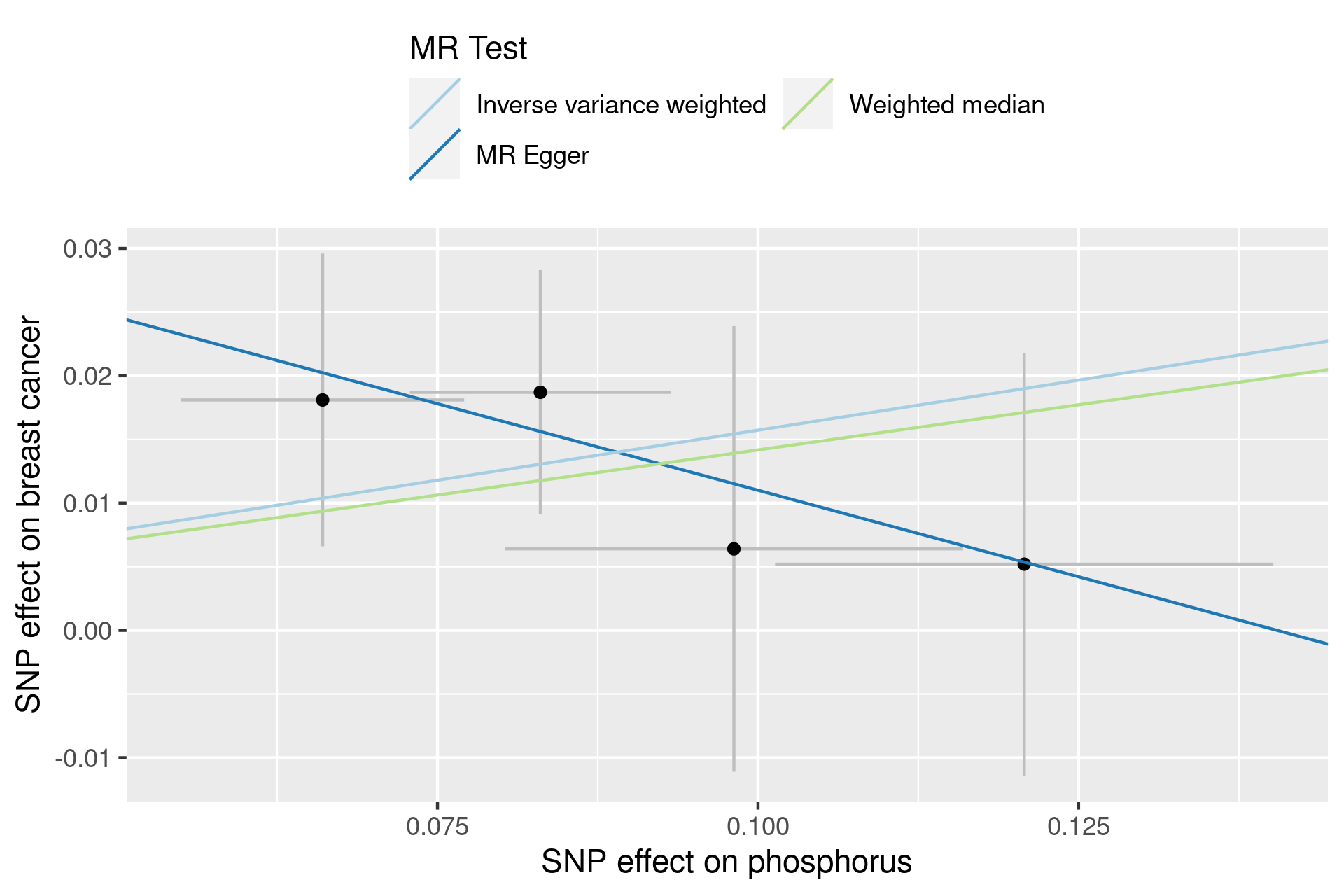


4) Funnel plot


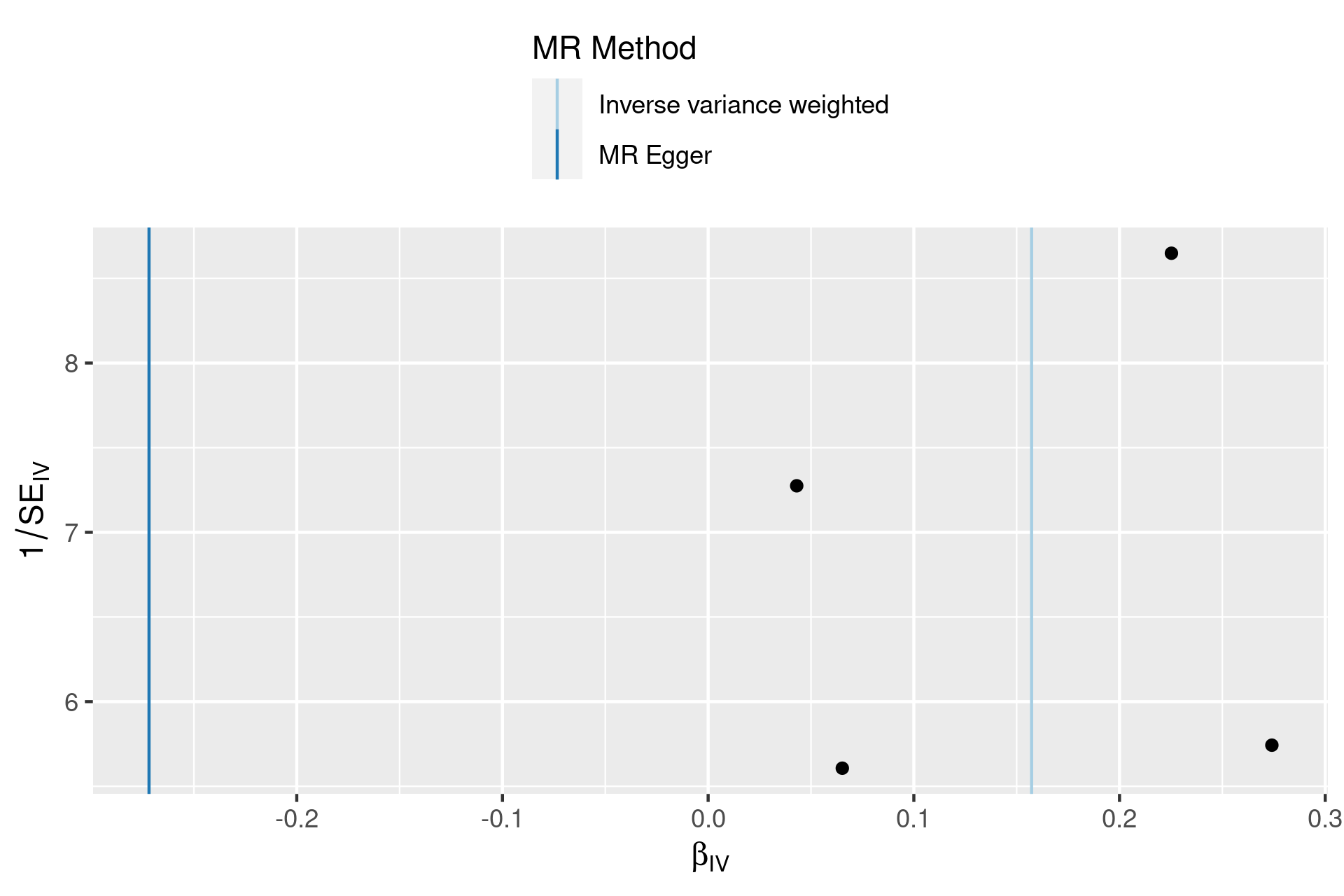


# Supplementary Figure 28. Genetic association of vitamin B9 (folate) with cervical cancer

1) Forest plot


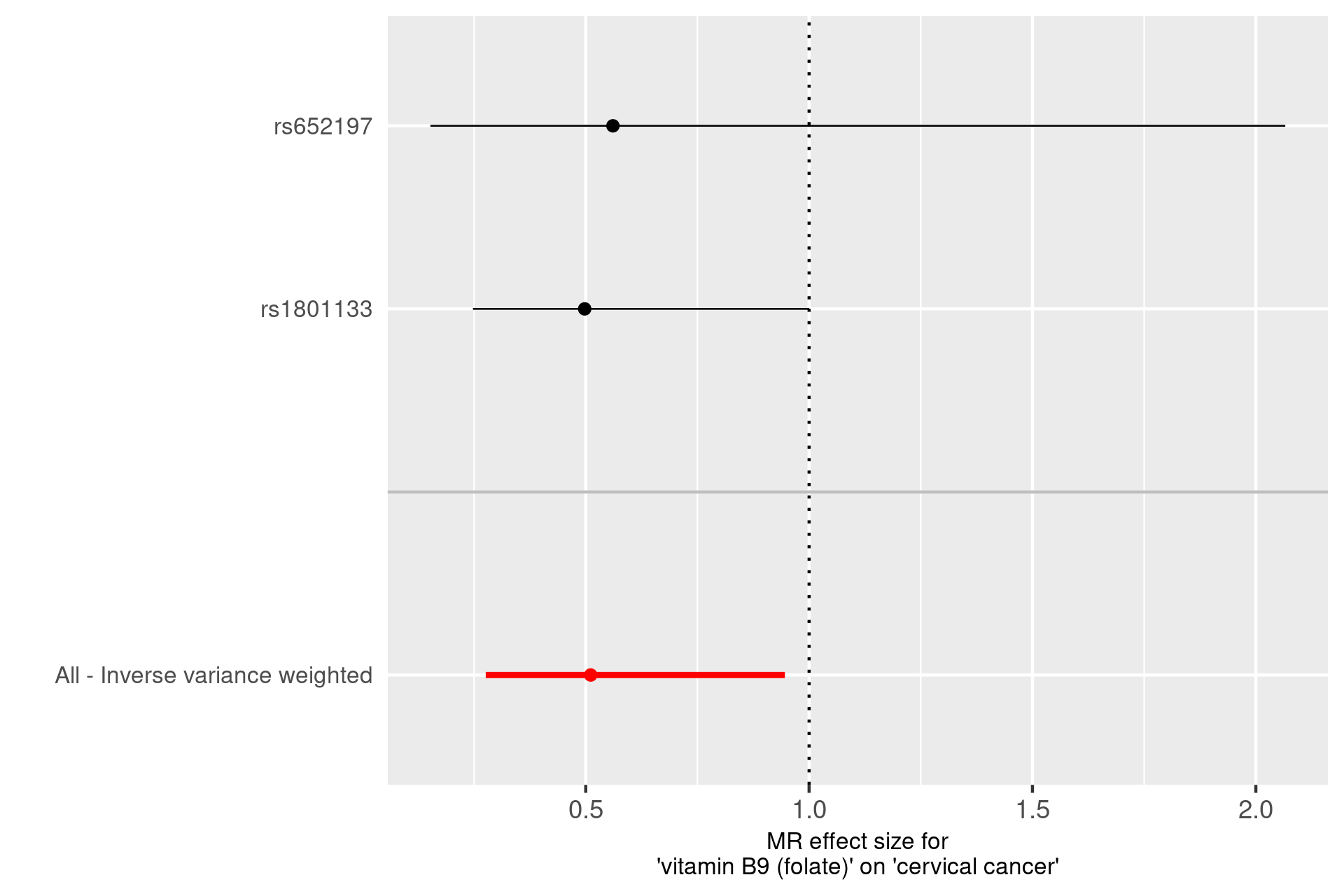


2) Leave-one-out plot

Not available because of small number of SNPs

3) Scatter plot


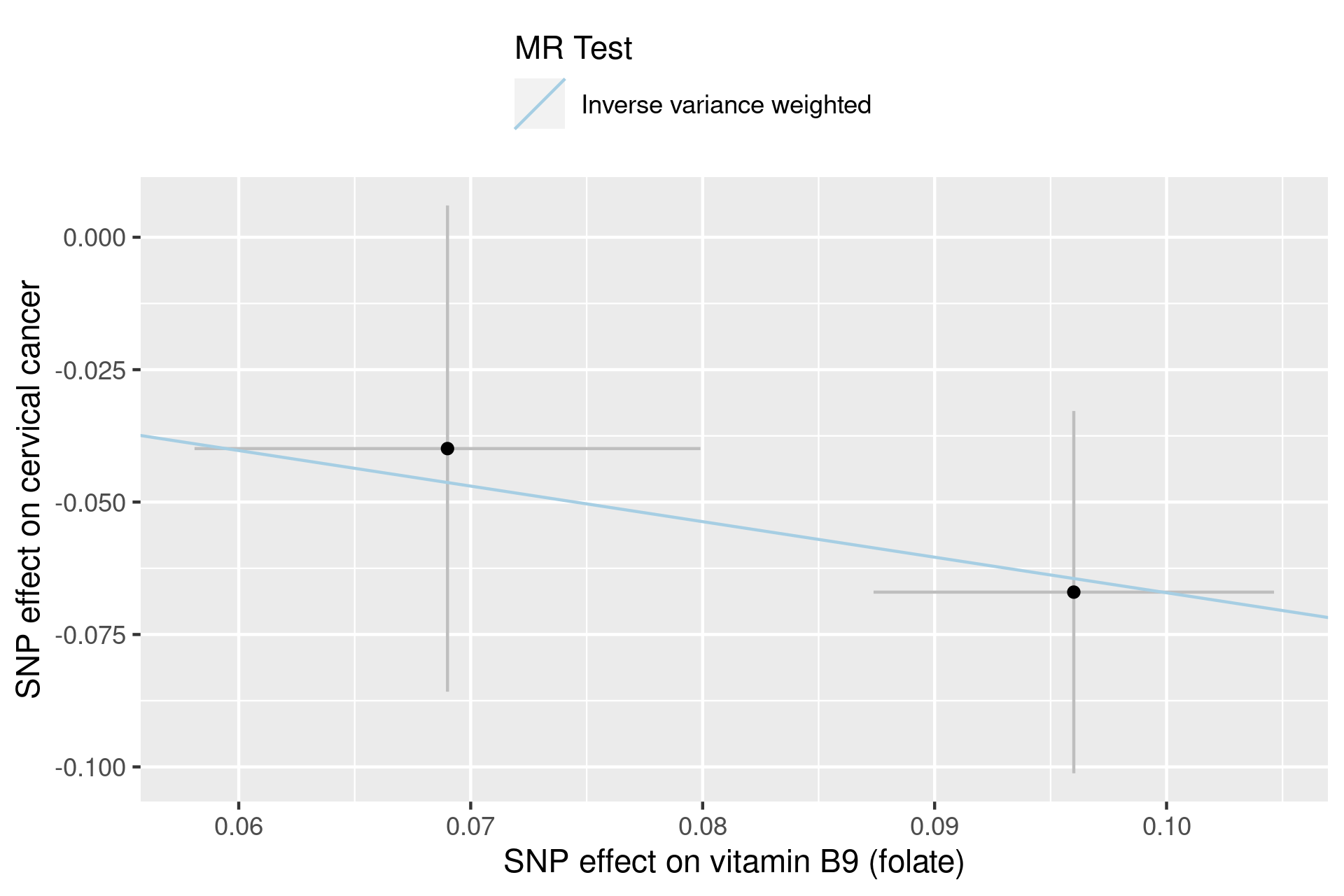


4) Funnel plot


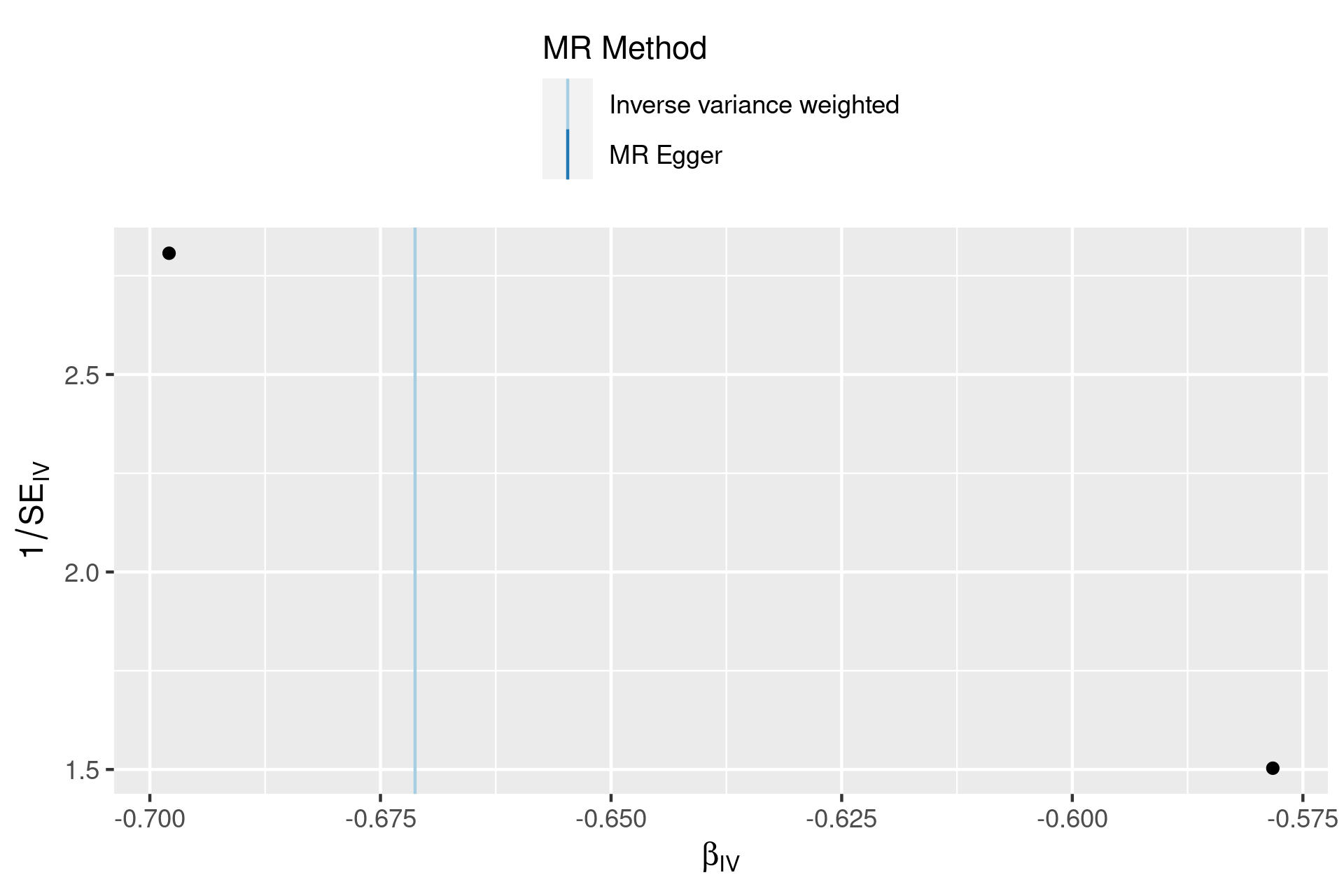


# Supplementary Figure 29. Genetic association of vitamin A1 (retinol) with liver cancer

1) Forest plot


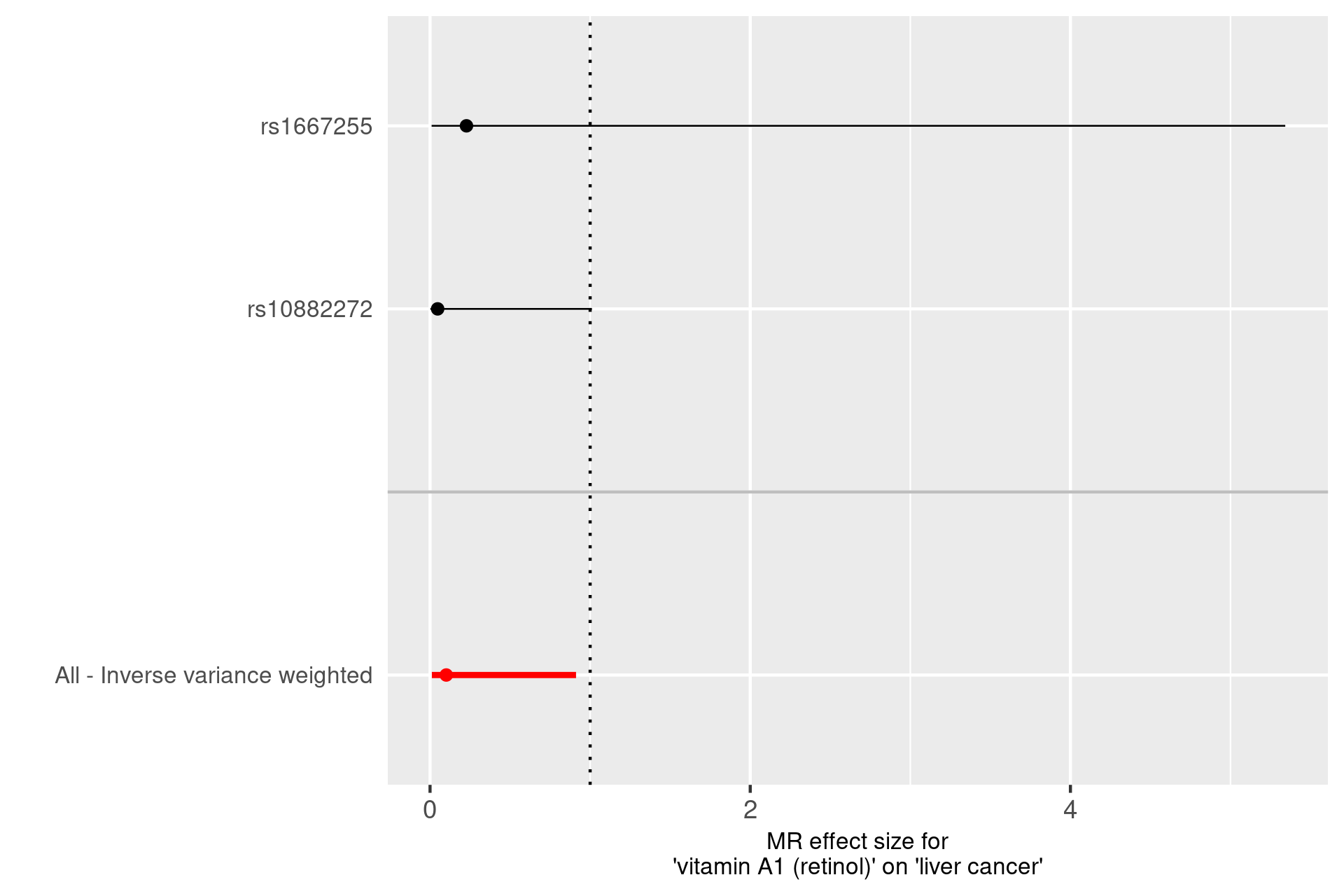


2) Leave-one-out plot

Not available because of small number of SNPs

3) Scatter plot


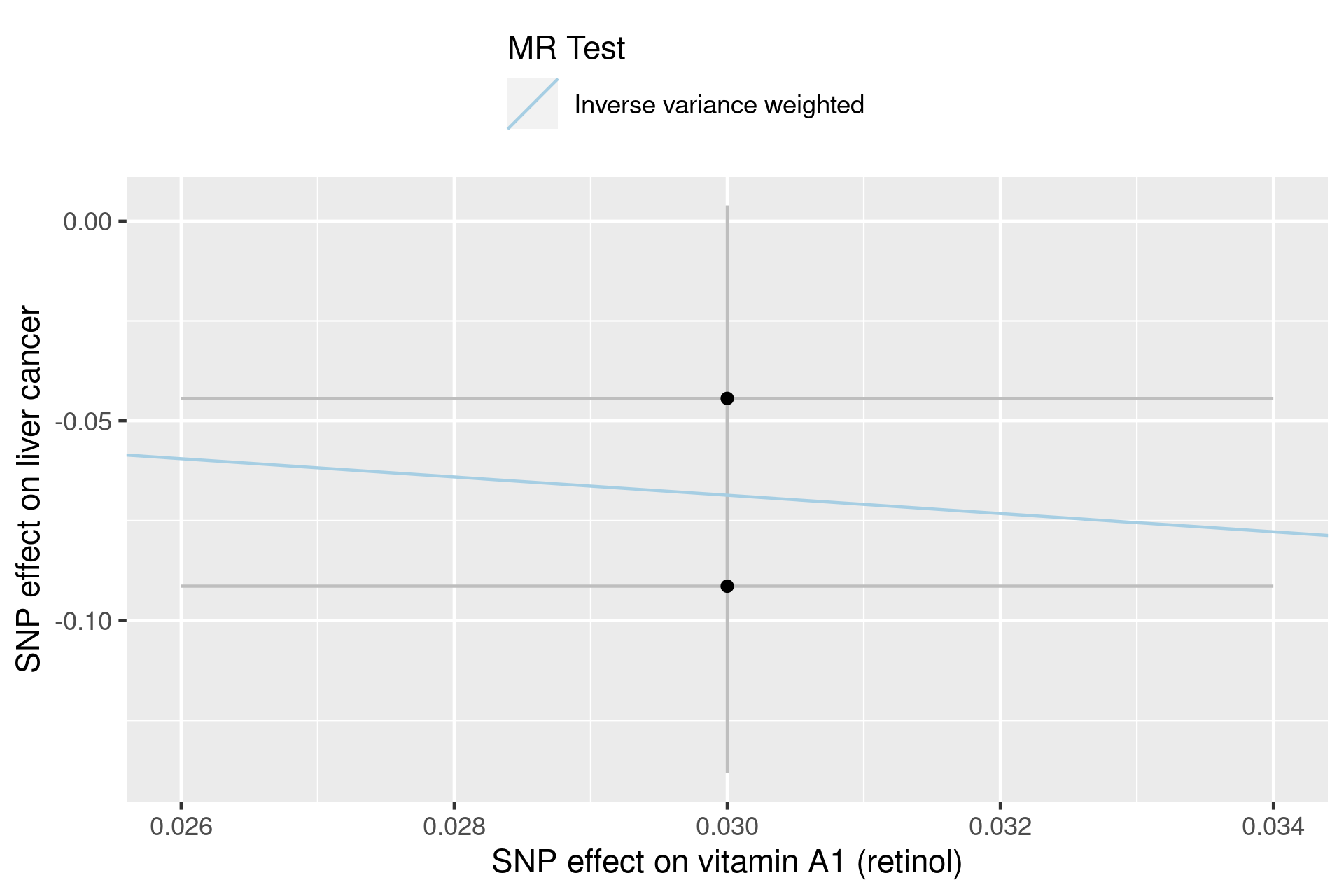


4) Funnel plot


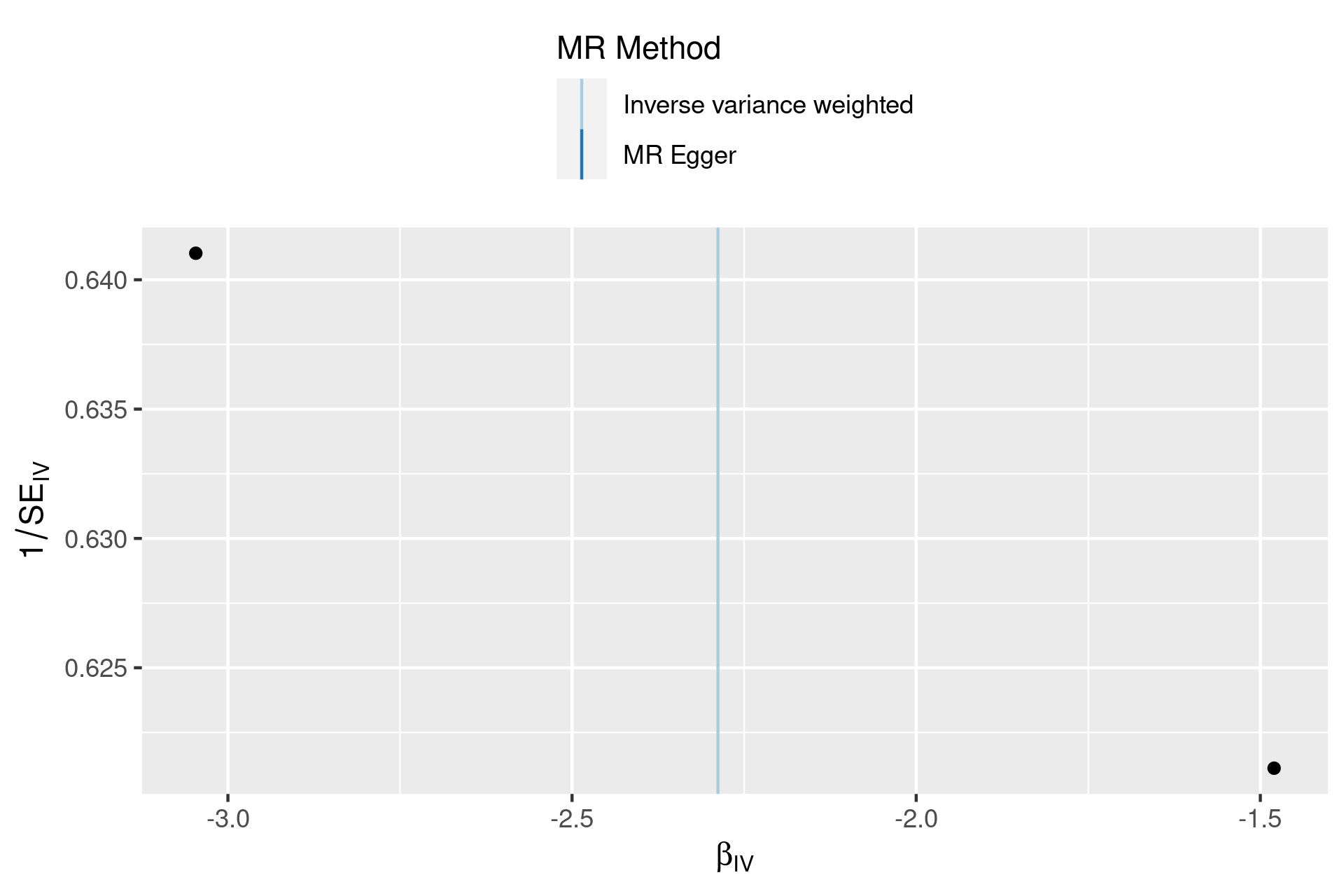


# Supplementary Figure 30. Genetic association of vitamin E with uterine cancer

1) Forest plot


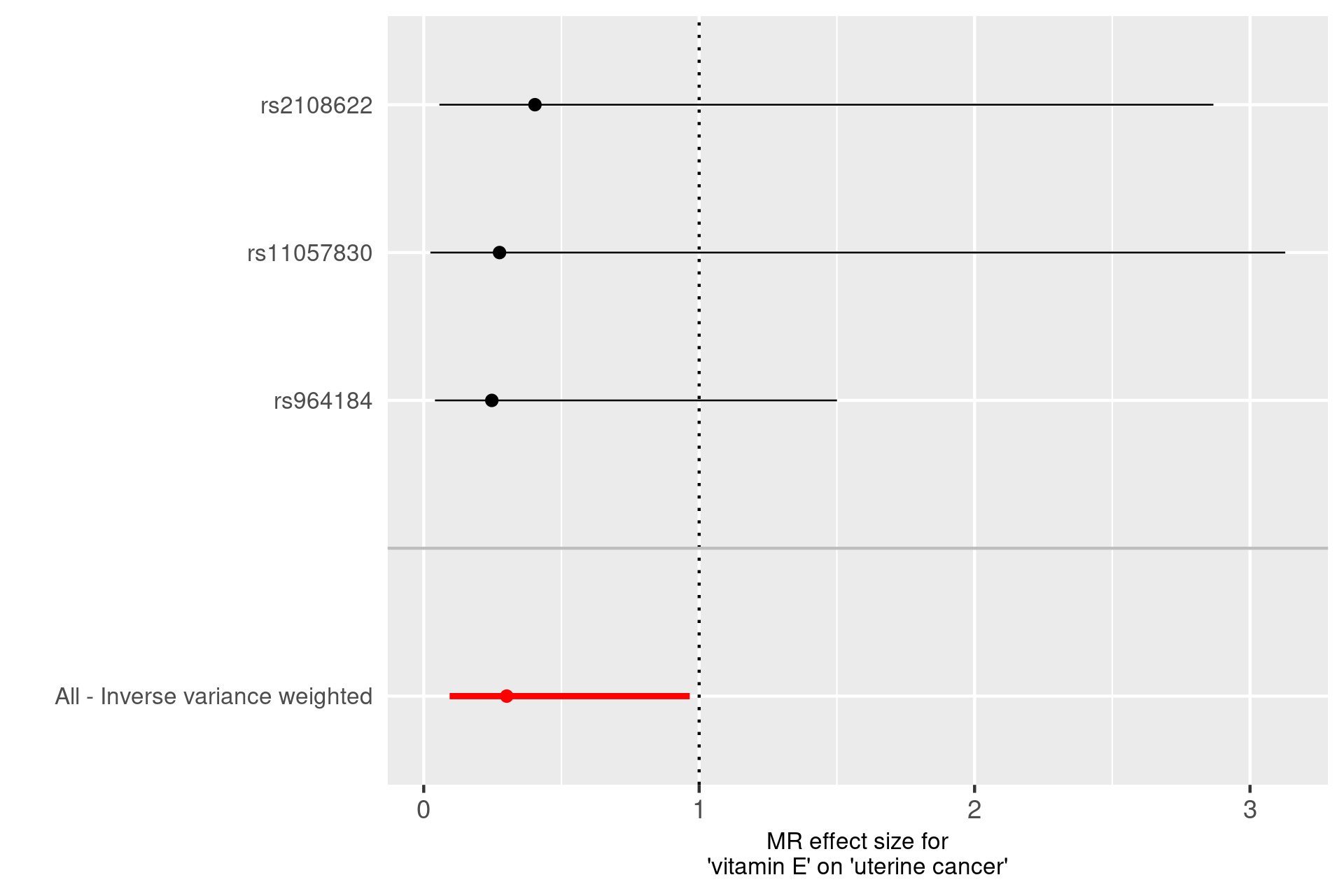


2) Leave-one-out plot


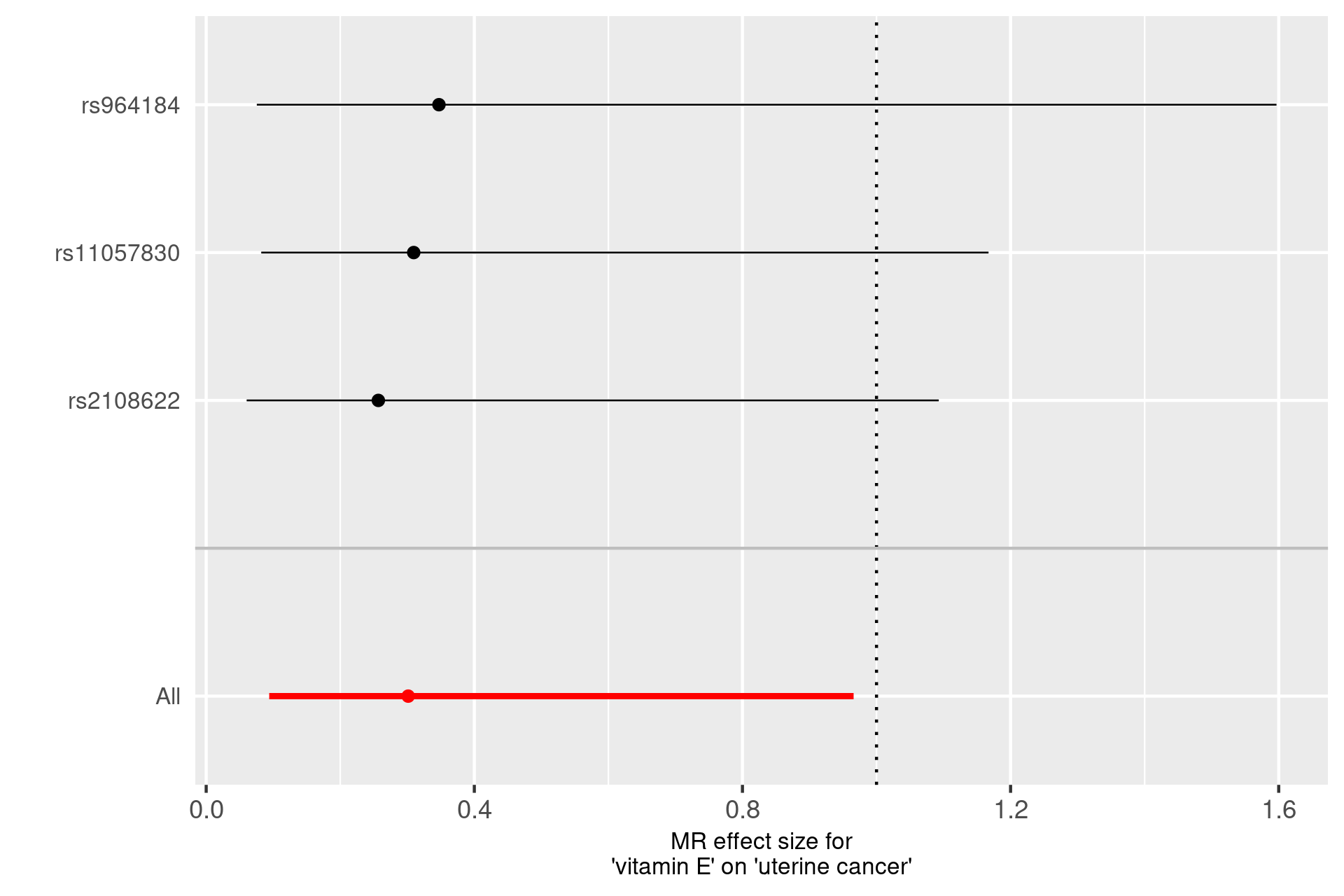


3) Scatter plot


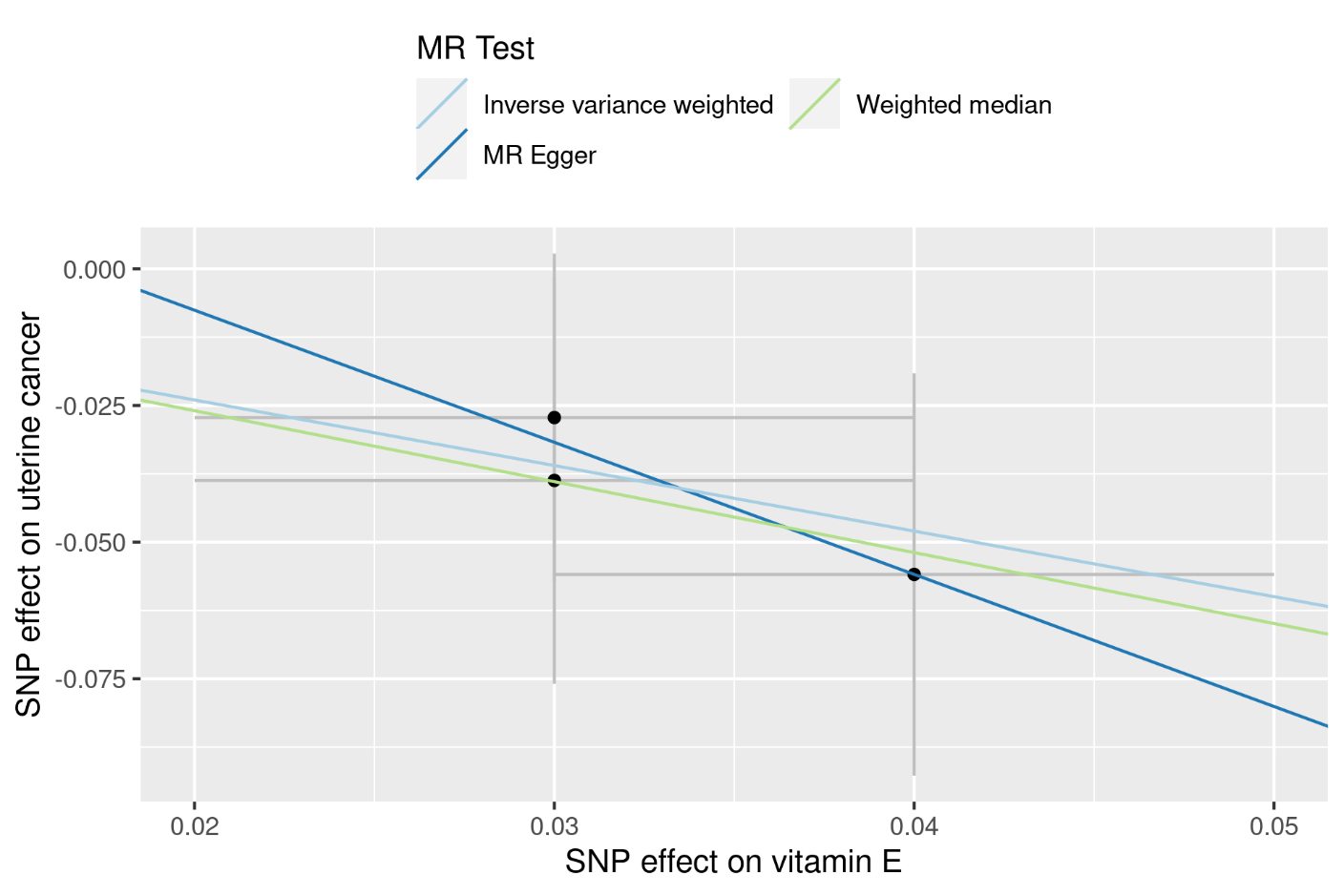


4) Funnel plot


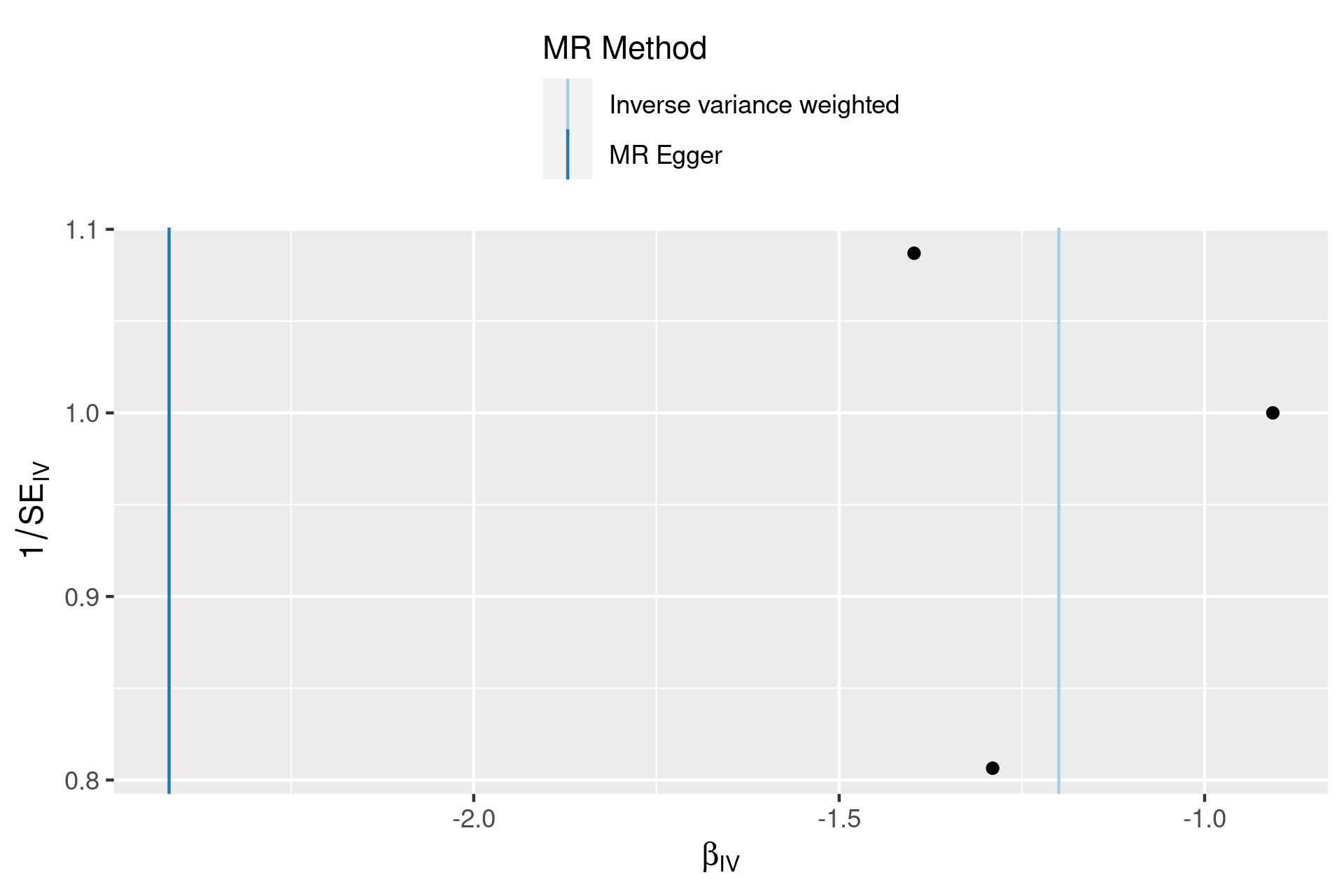


# Supplementary Figure 31. Genetic association of vitamin A1 (retinol) with brain cancer

1) Forest plot


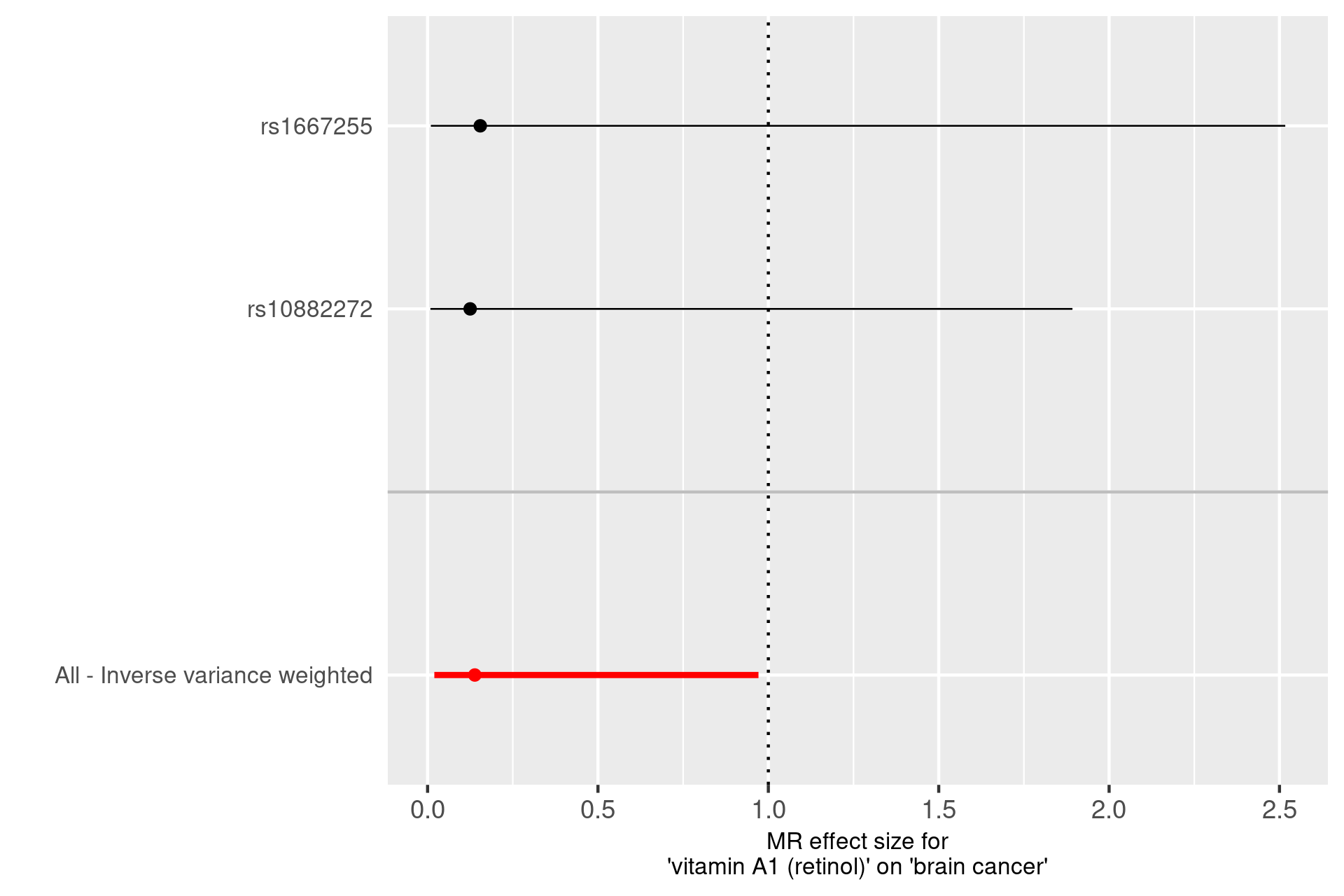


2) Leave-one-out plot

Not available because of small number of SNPs

3) Scatter plot


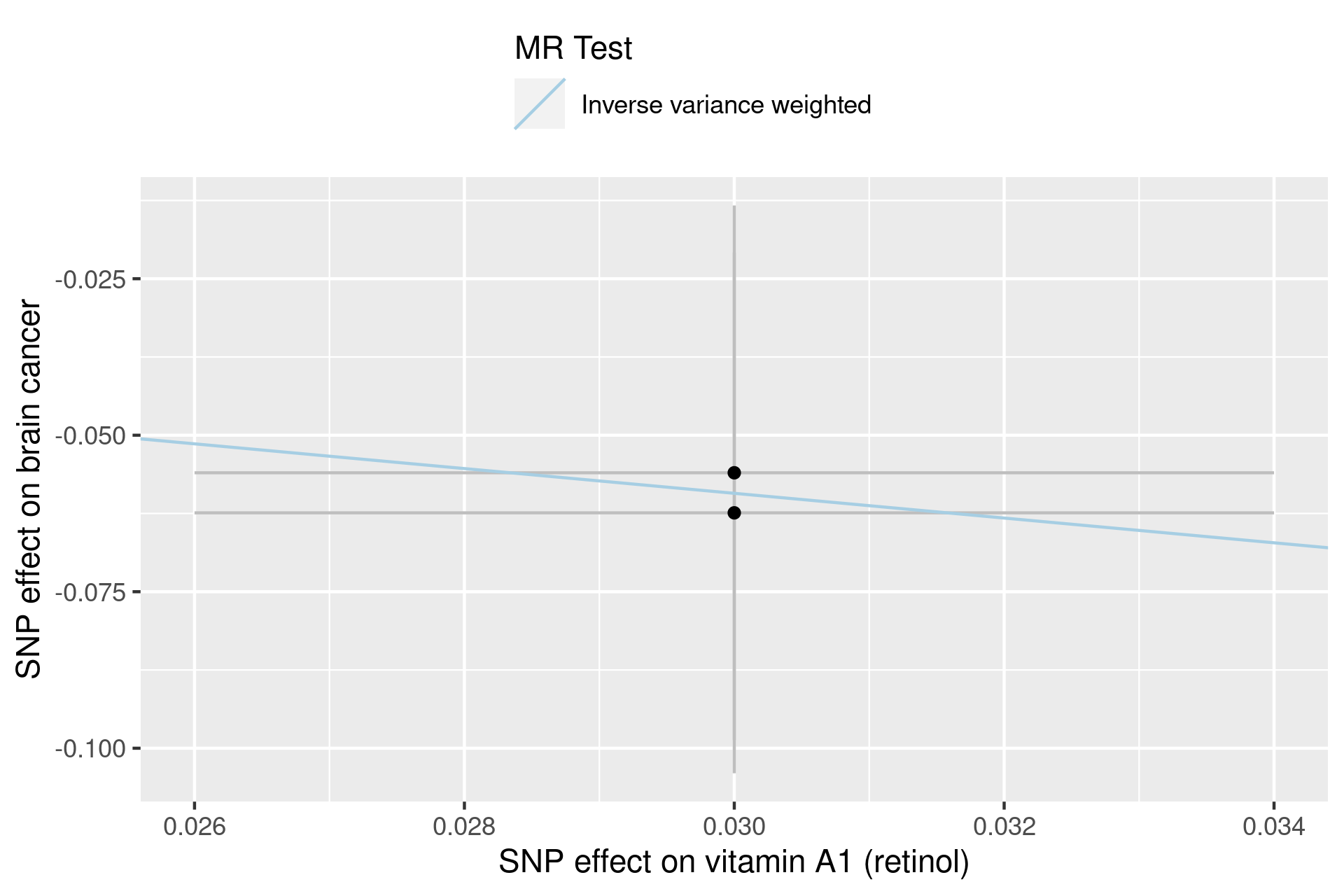


4) Funnel plot


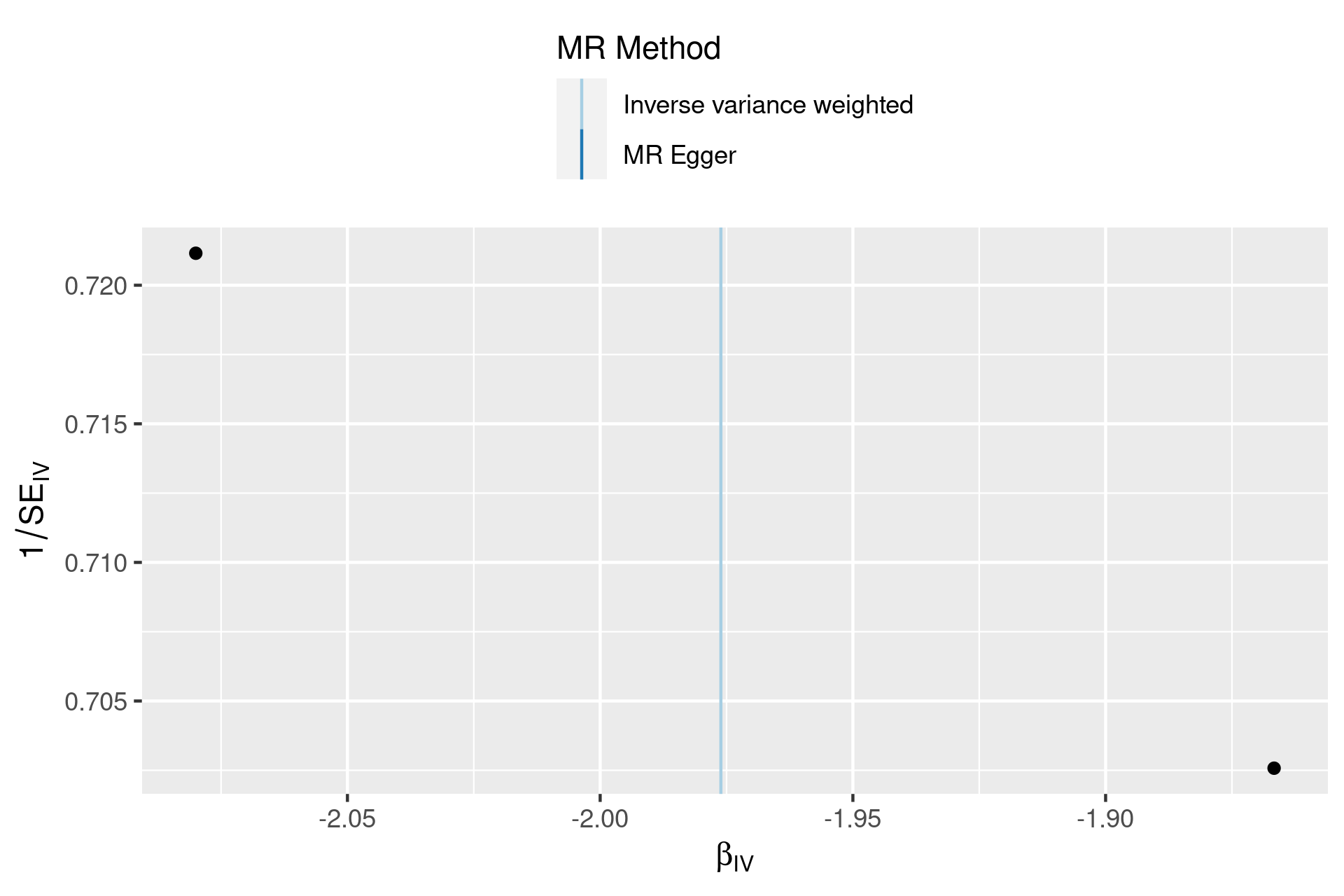


# Supplementary Figure 32. Genetic association of magnesium with breast cancer, overall

1) Forest plot


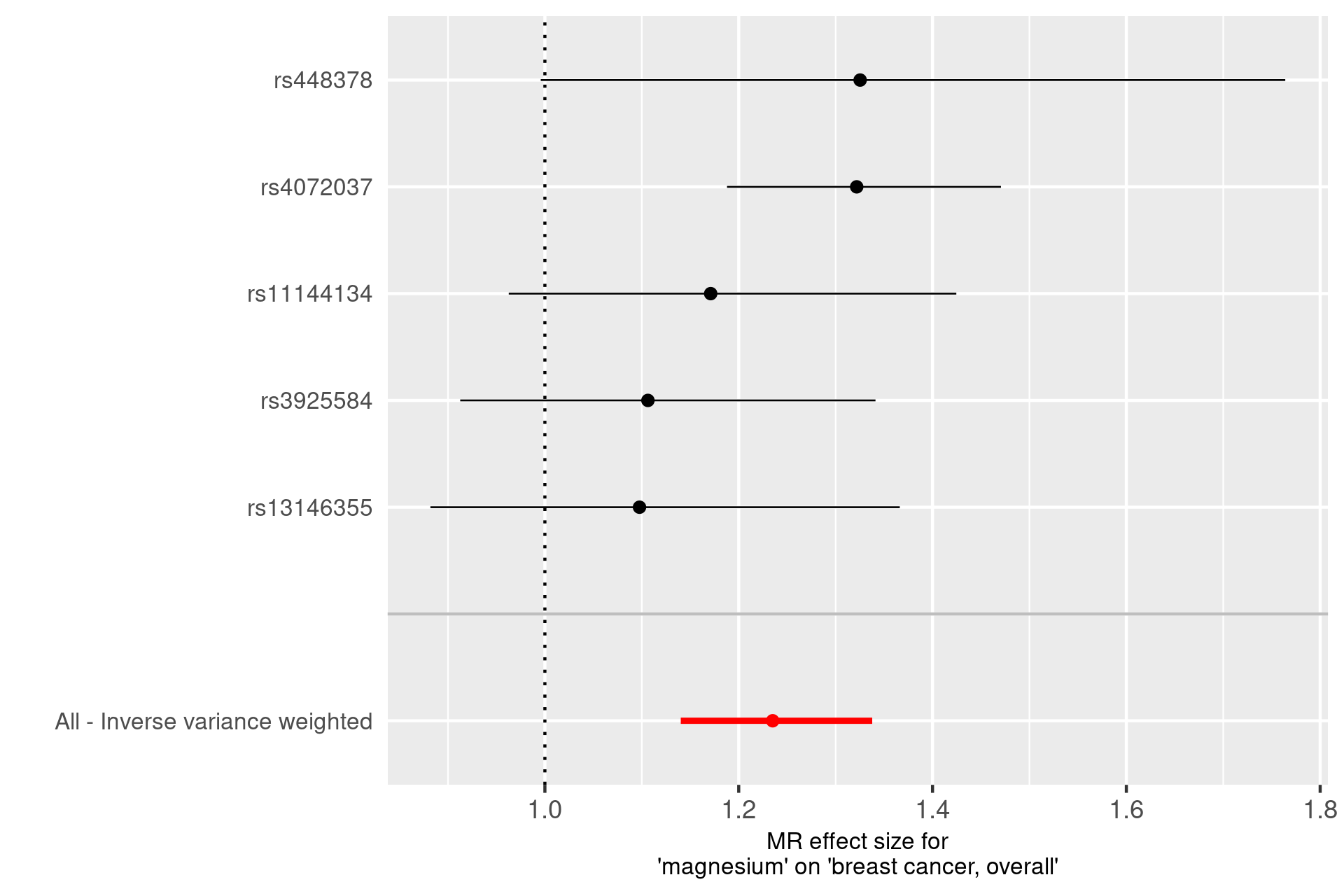


2) Leave-one-out plot


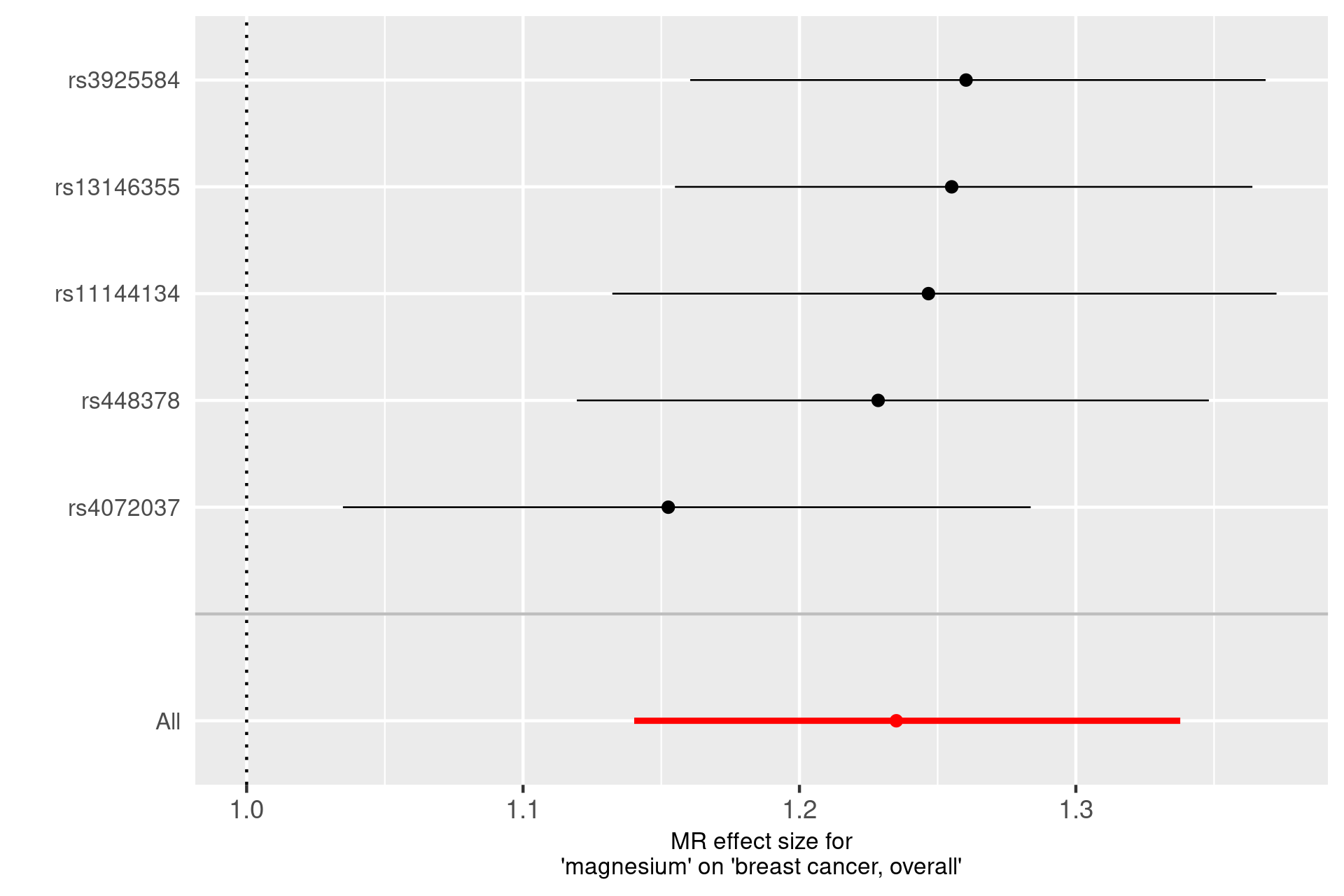


3) Scatter plot


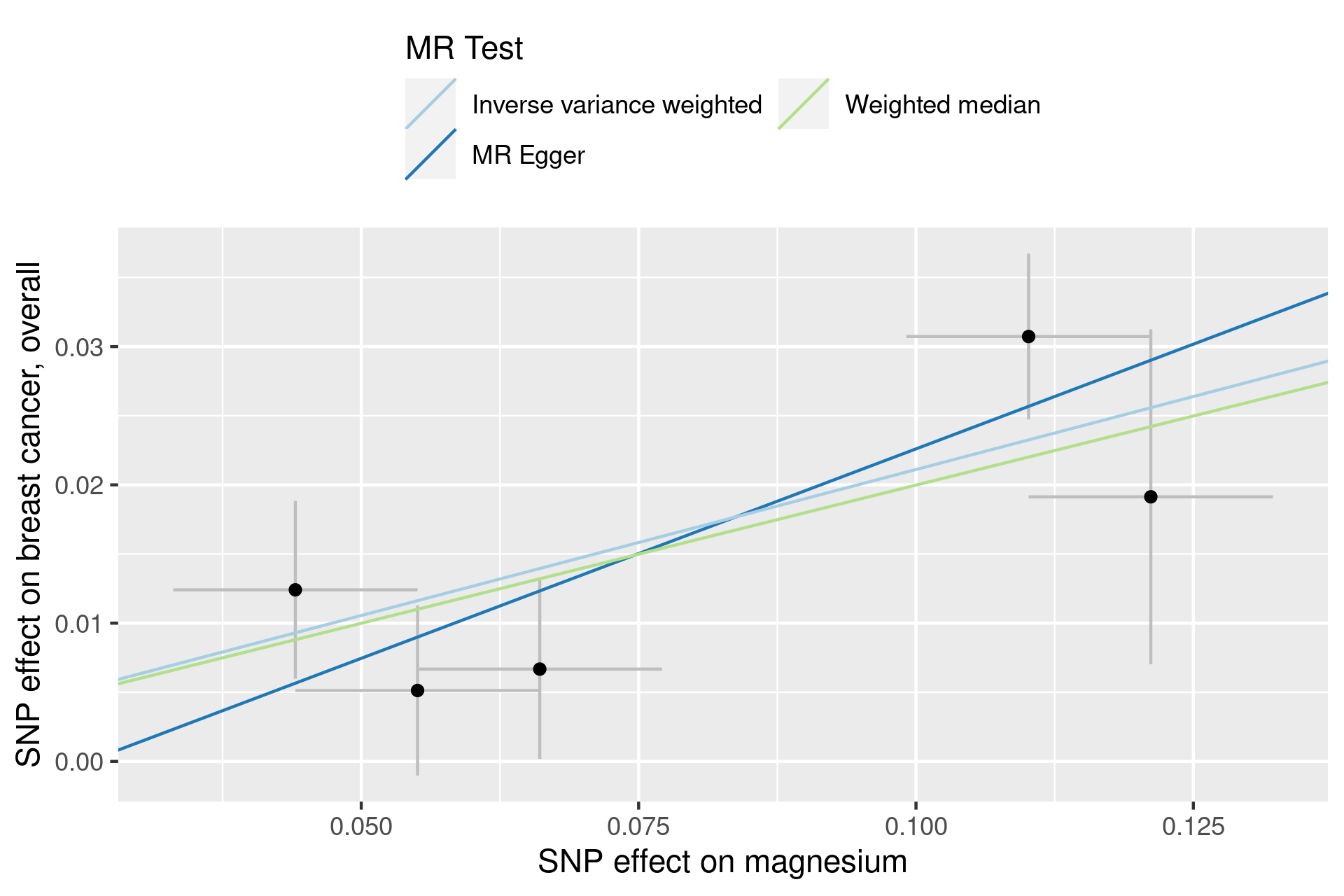


4) Funnel plot


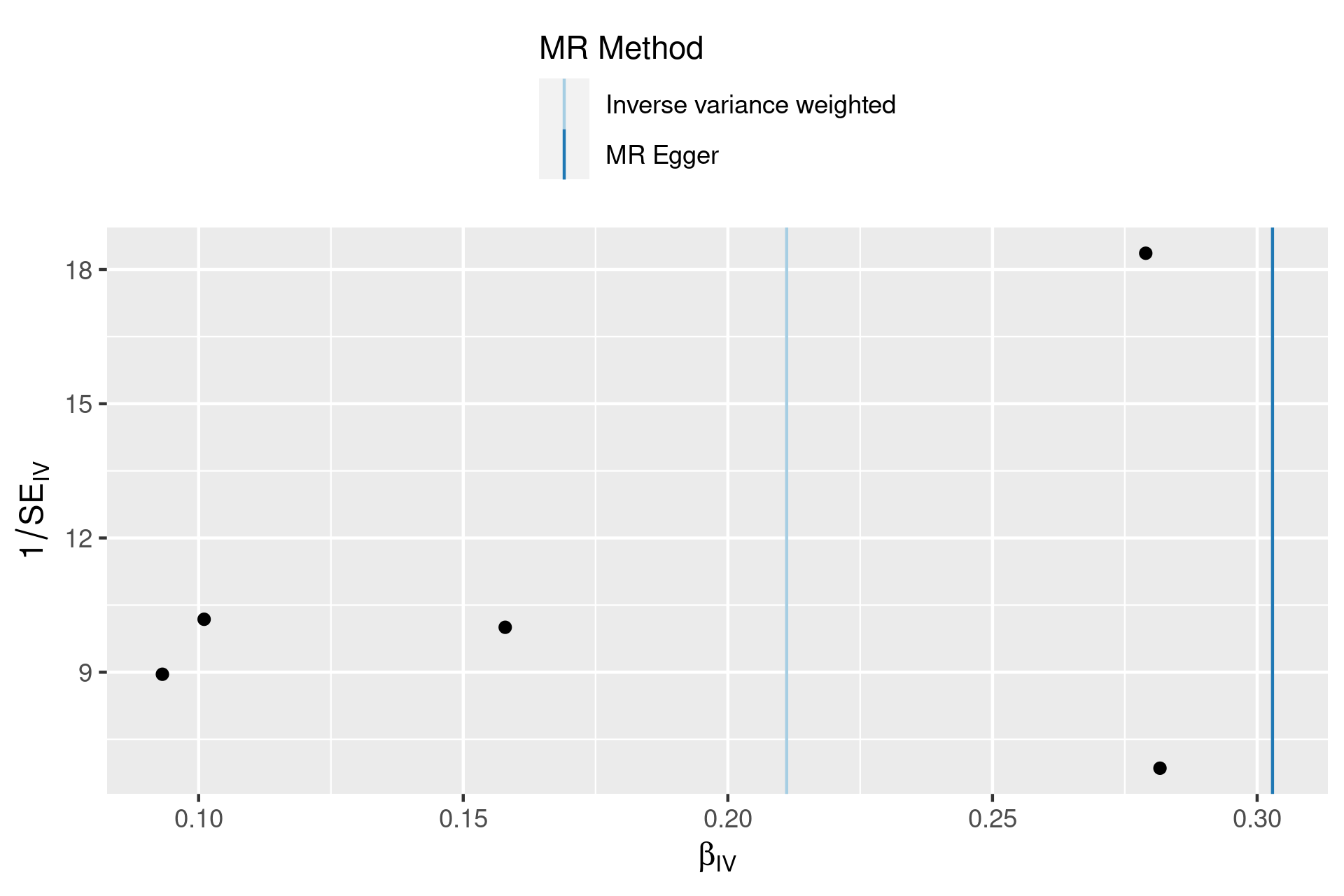


# Supplementary Figure 33. Genetic association of vitamin B12 with ovarian cancer, non-invasive

1) Forest plot


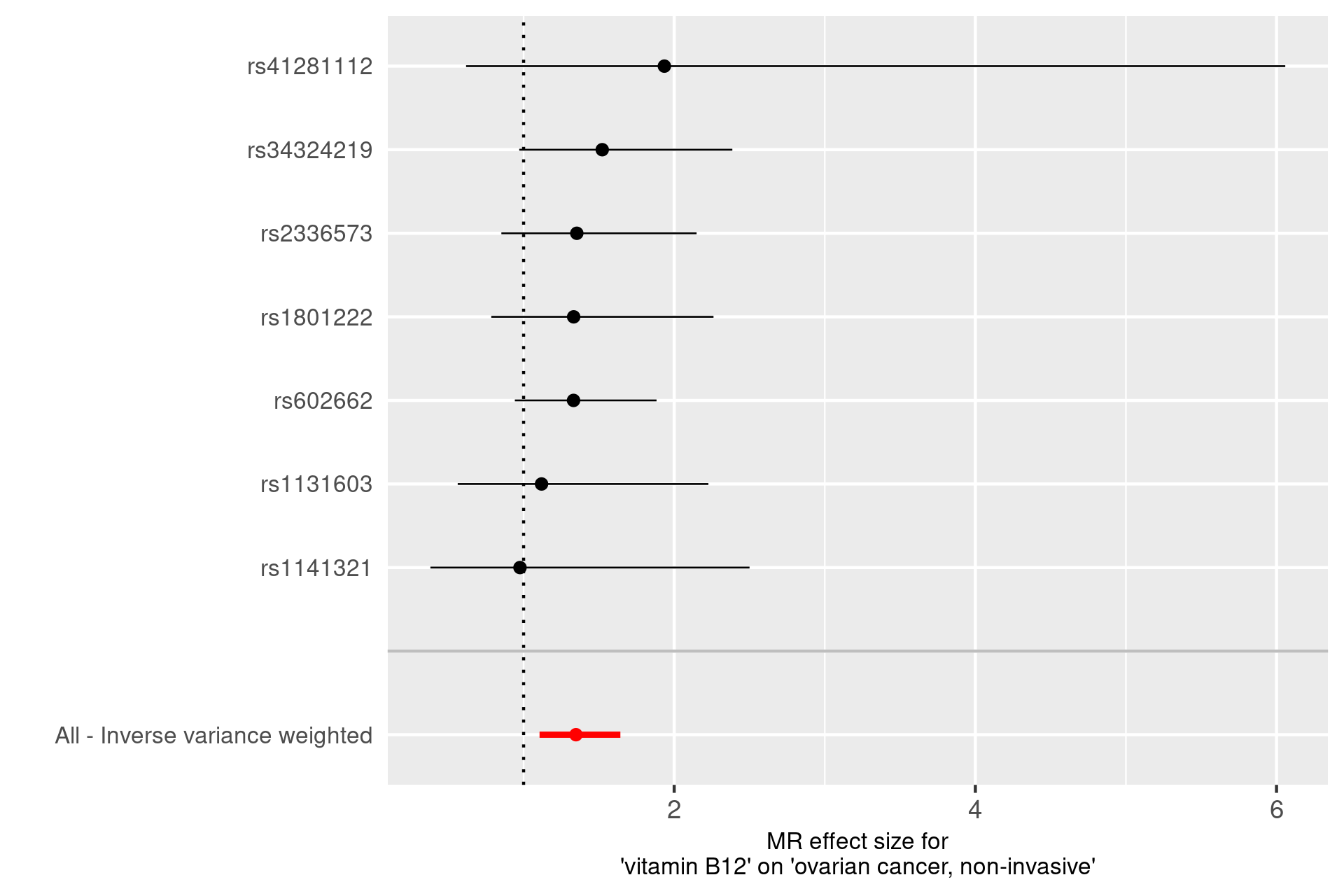


2) Leave-one-out plot


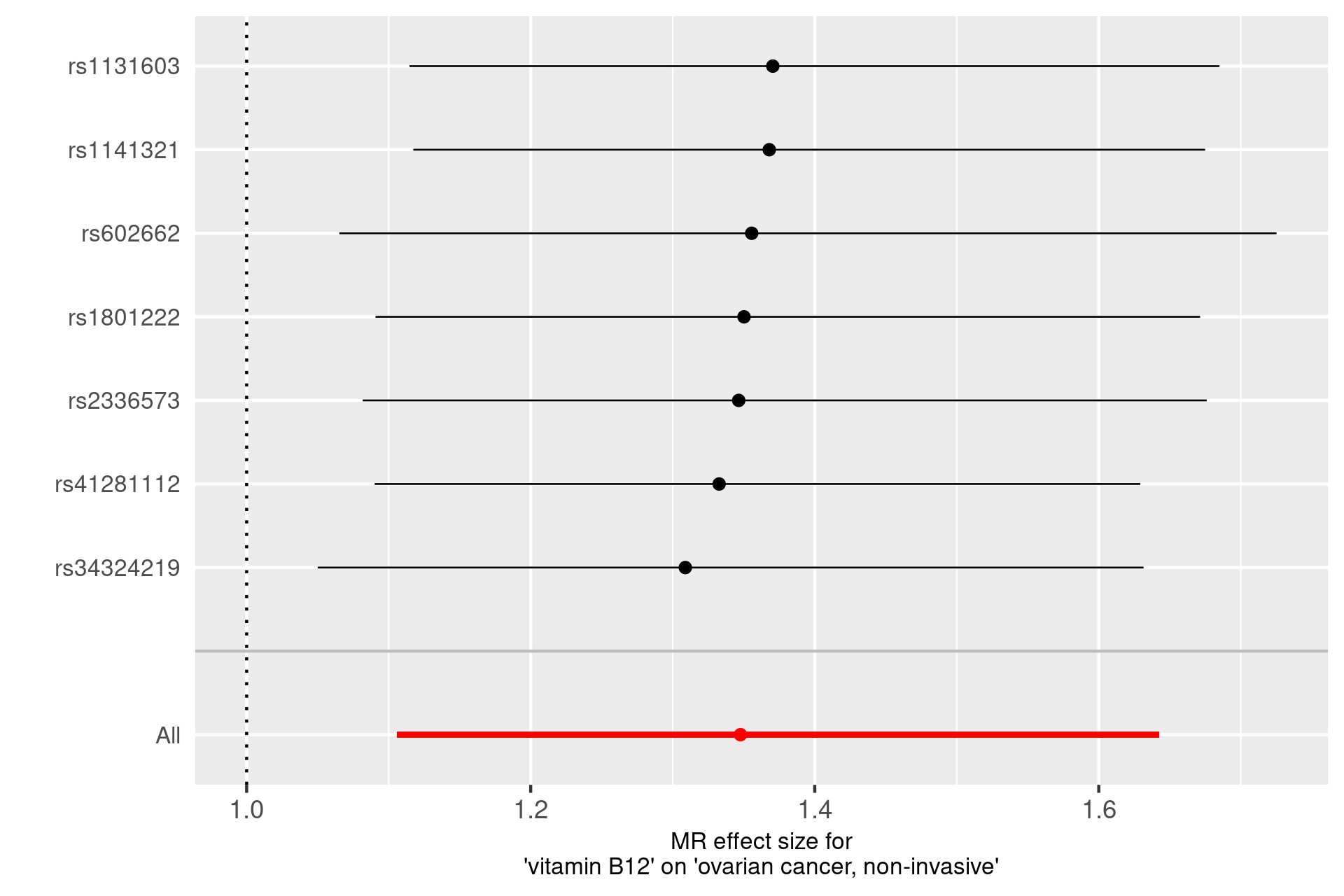


3) Scatter plot


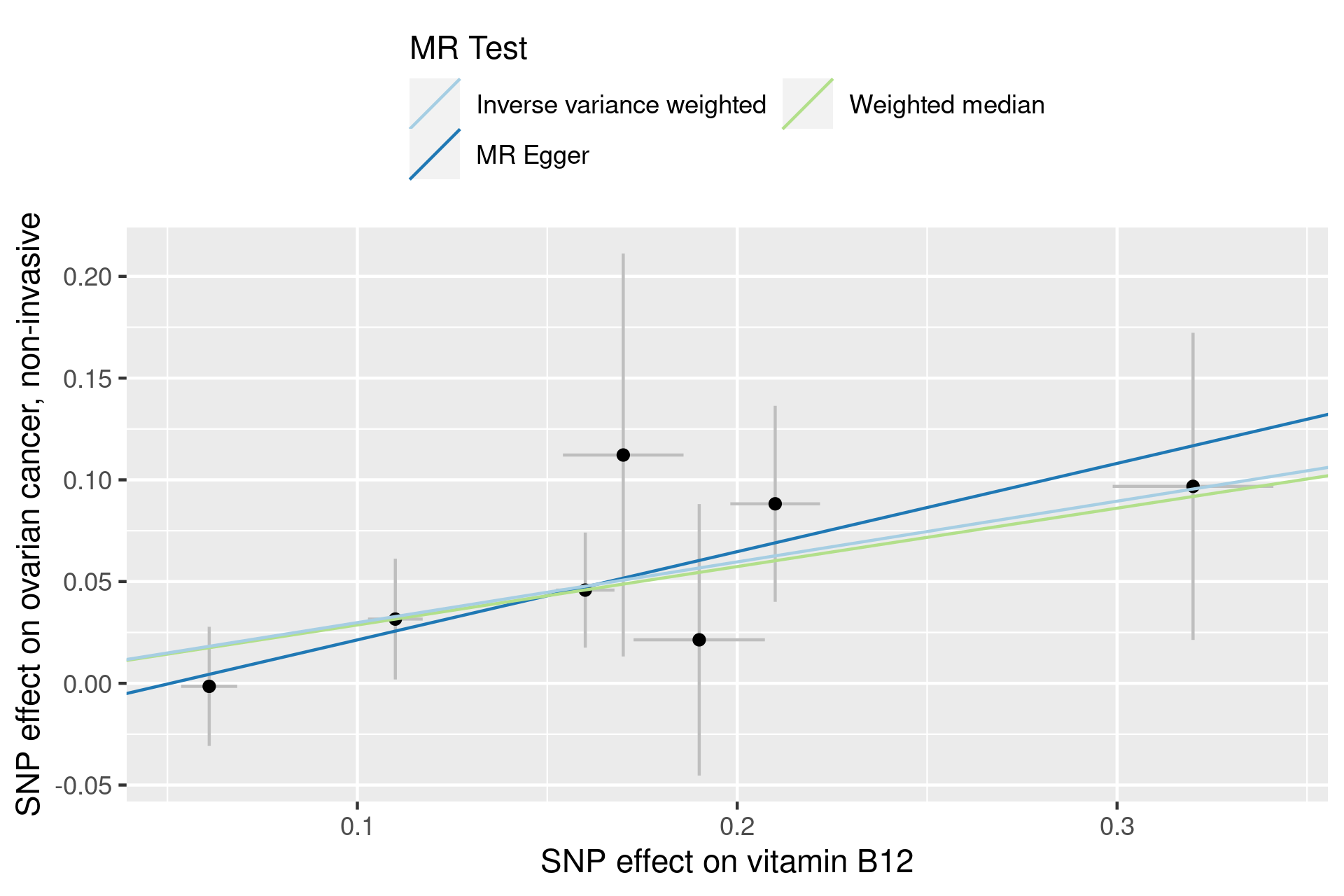


4) Funnel plot


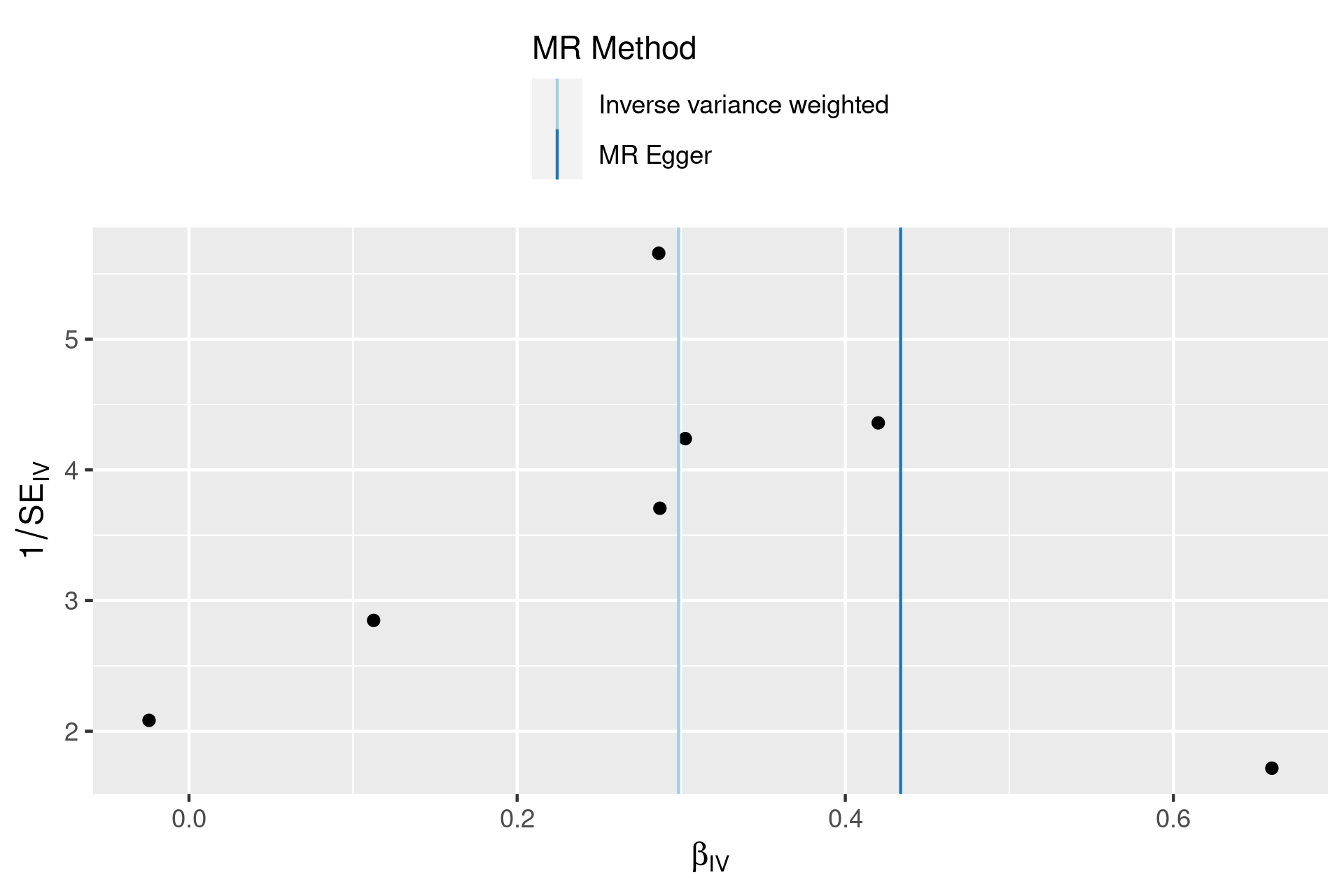


# Supplementary Figure 34. Genetic association of zinc with colorectal cancer

1) Forest plot


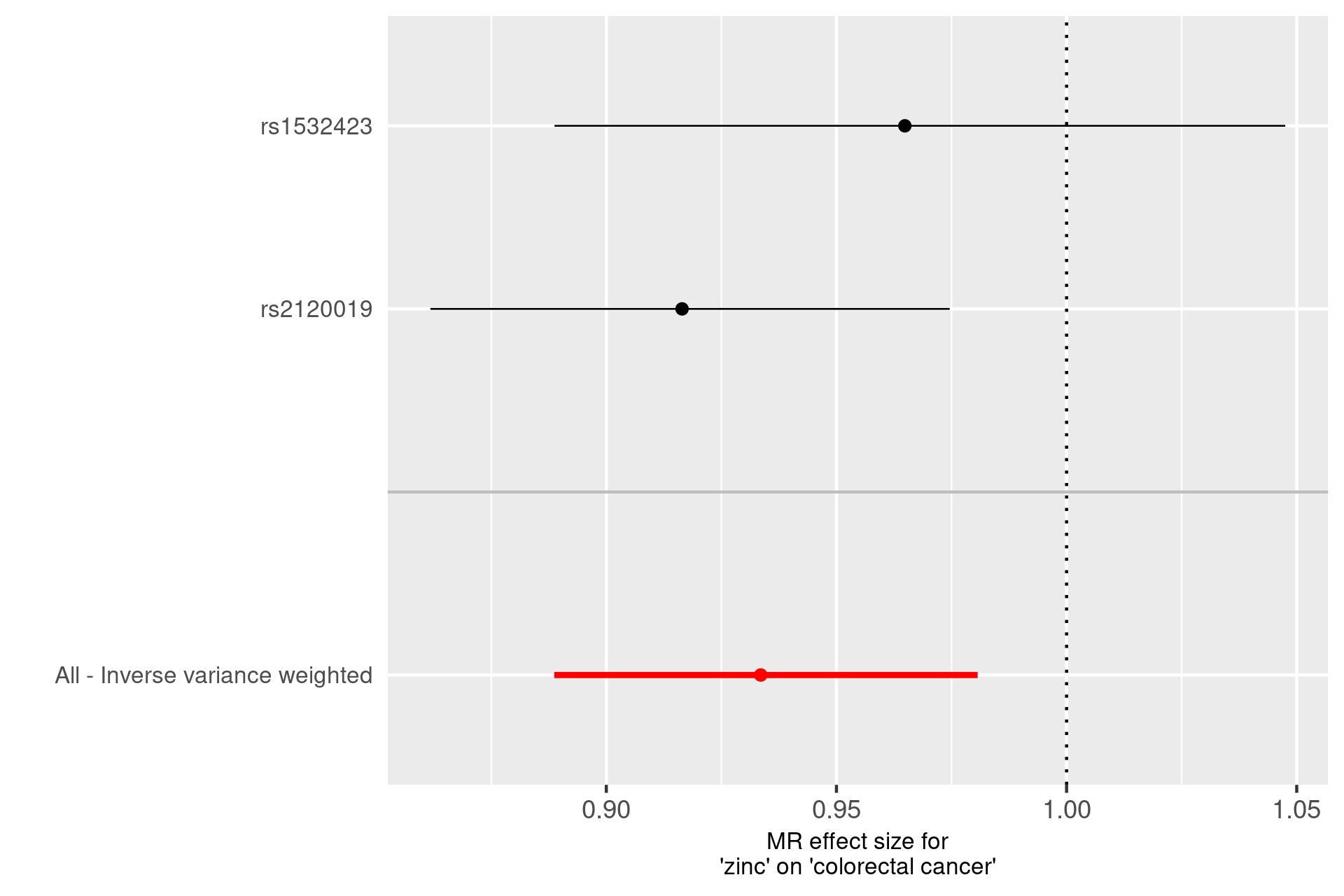


2) Leave-one-out plot

Not available because of small number of SNPs

3) Scatter plot


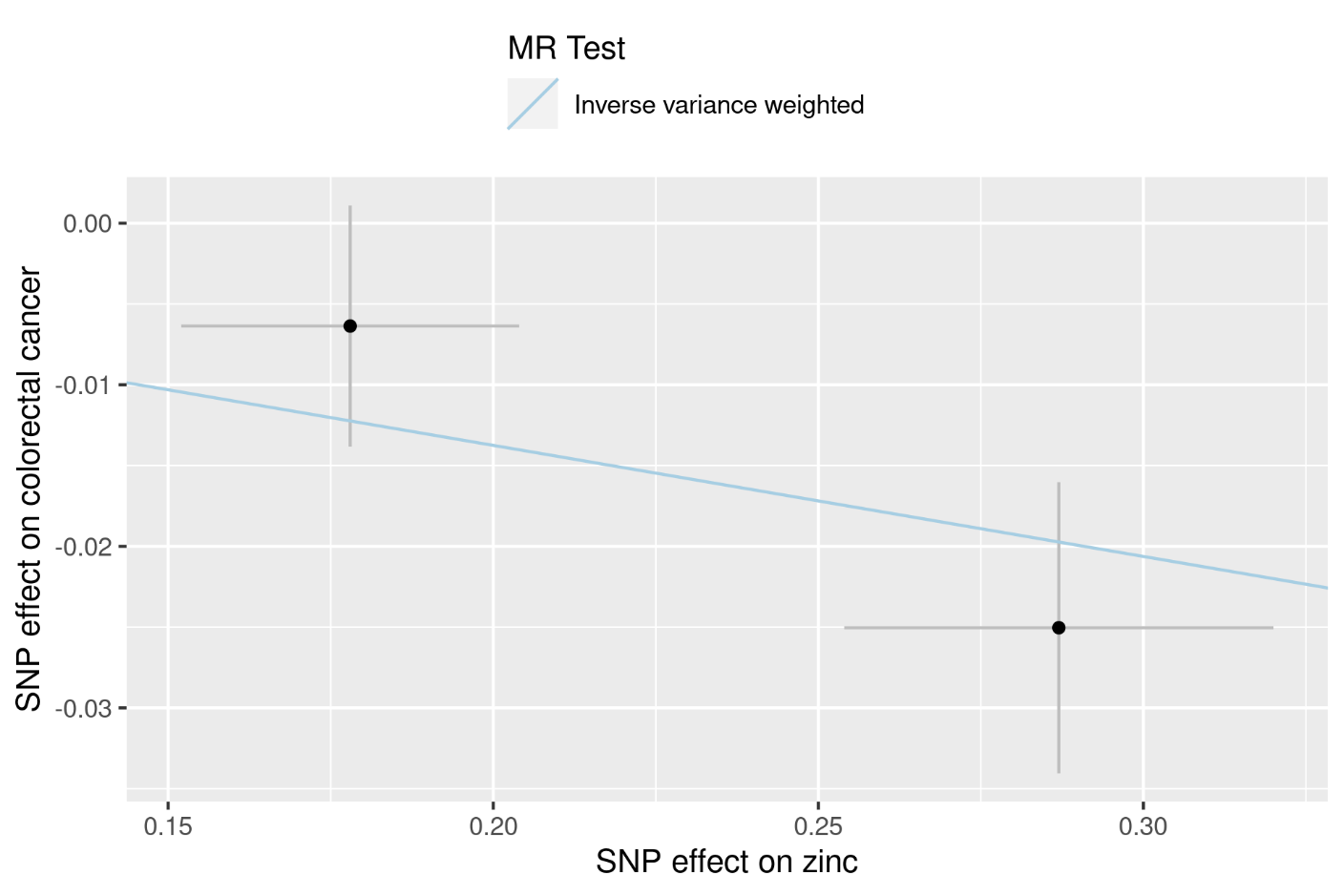


4) Funnel plot


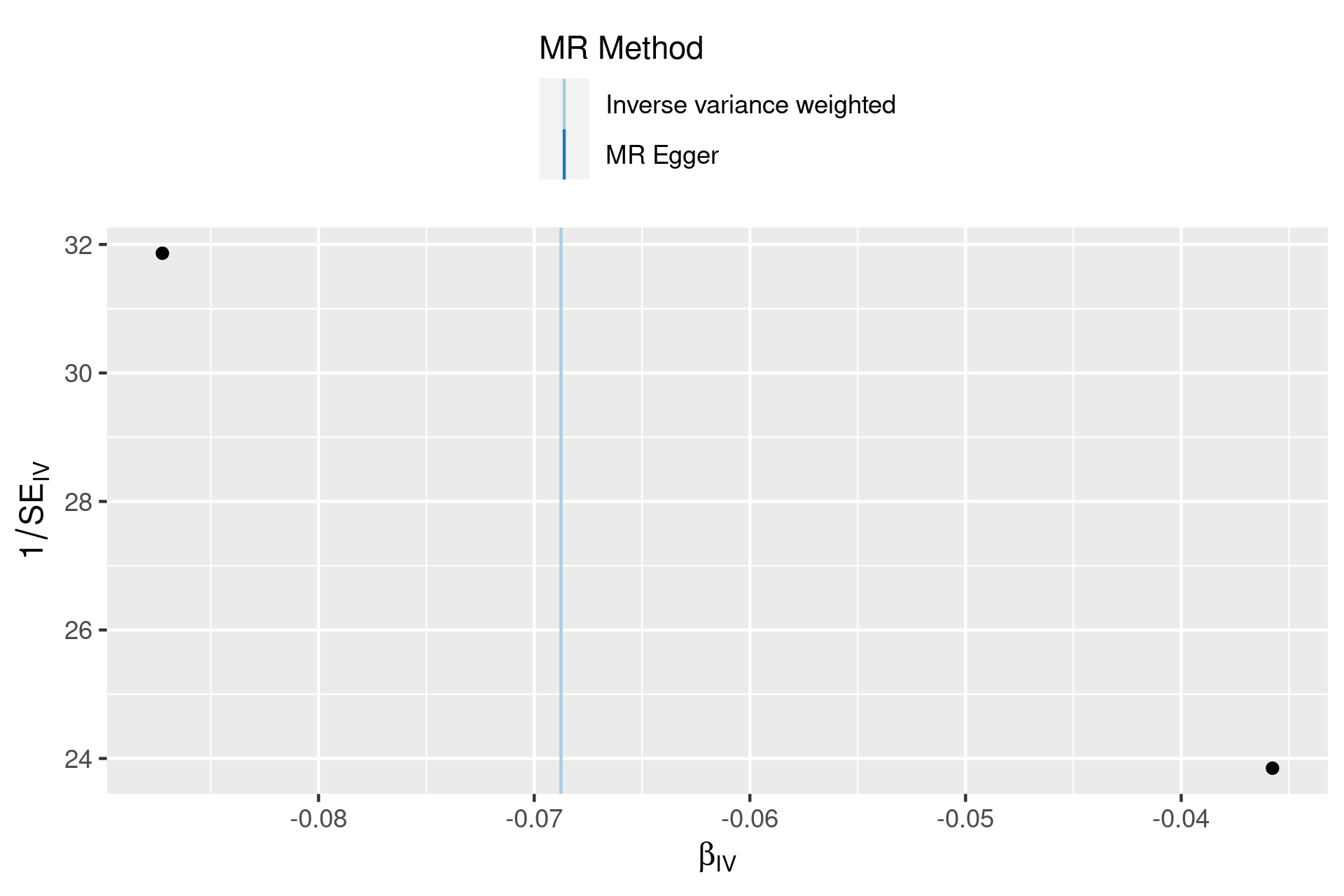


# Supplementary Figure 35. Genetic association of vitamin B12 with prostate cancer

1) Forest plot


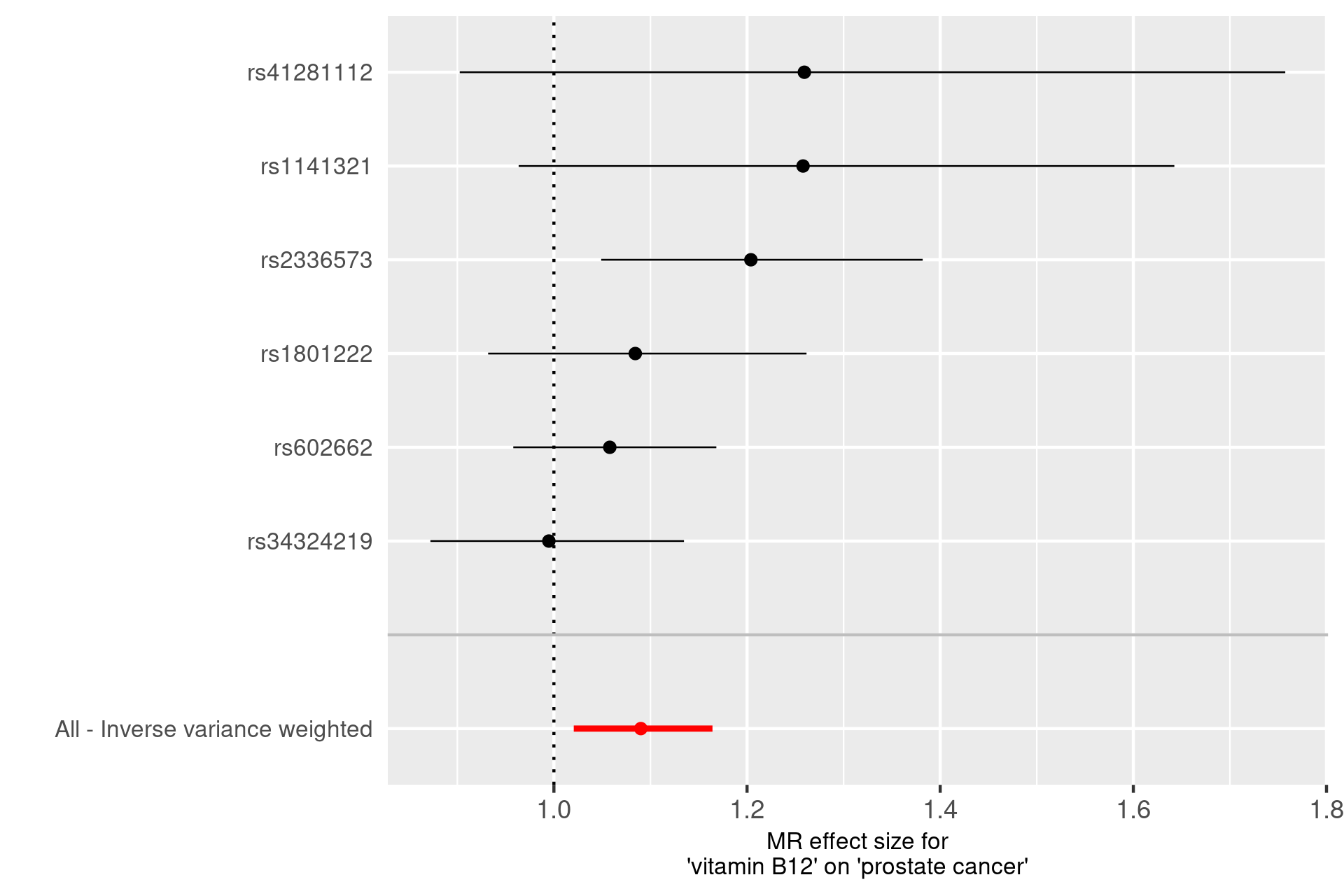


2) Leave-one-out plot


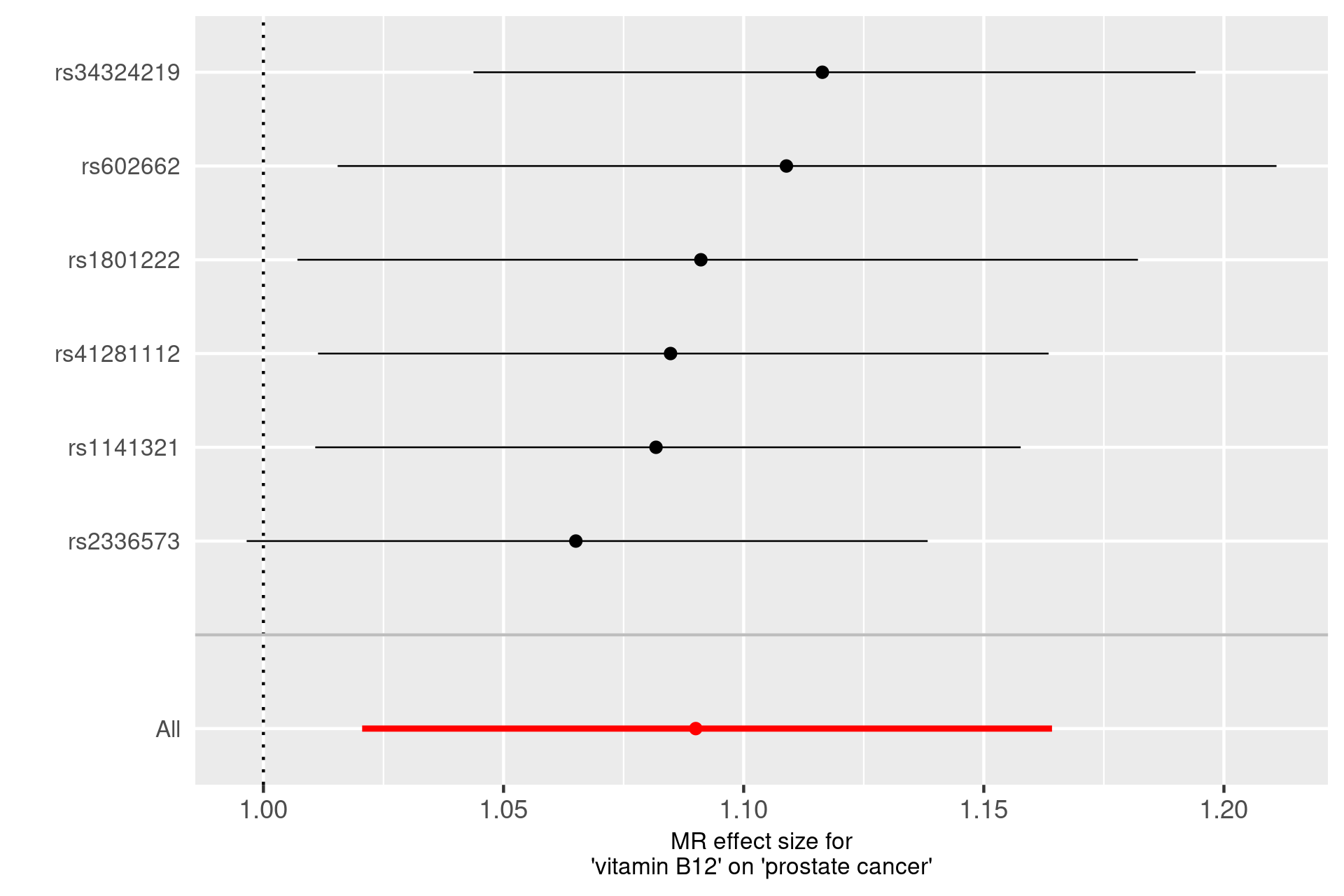


3) Scatter plot


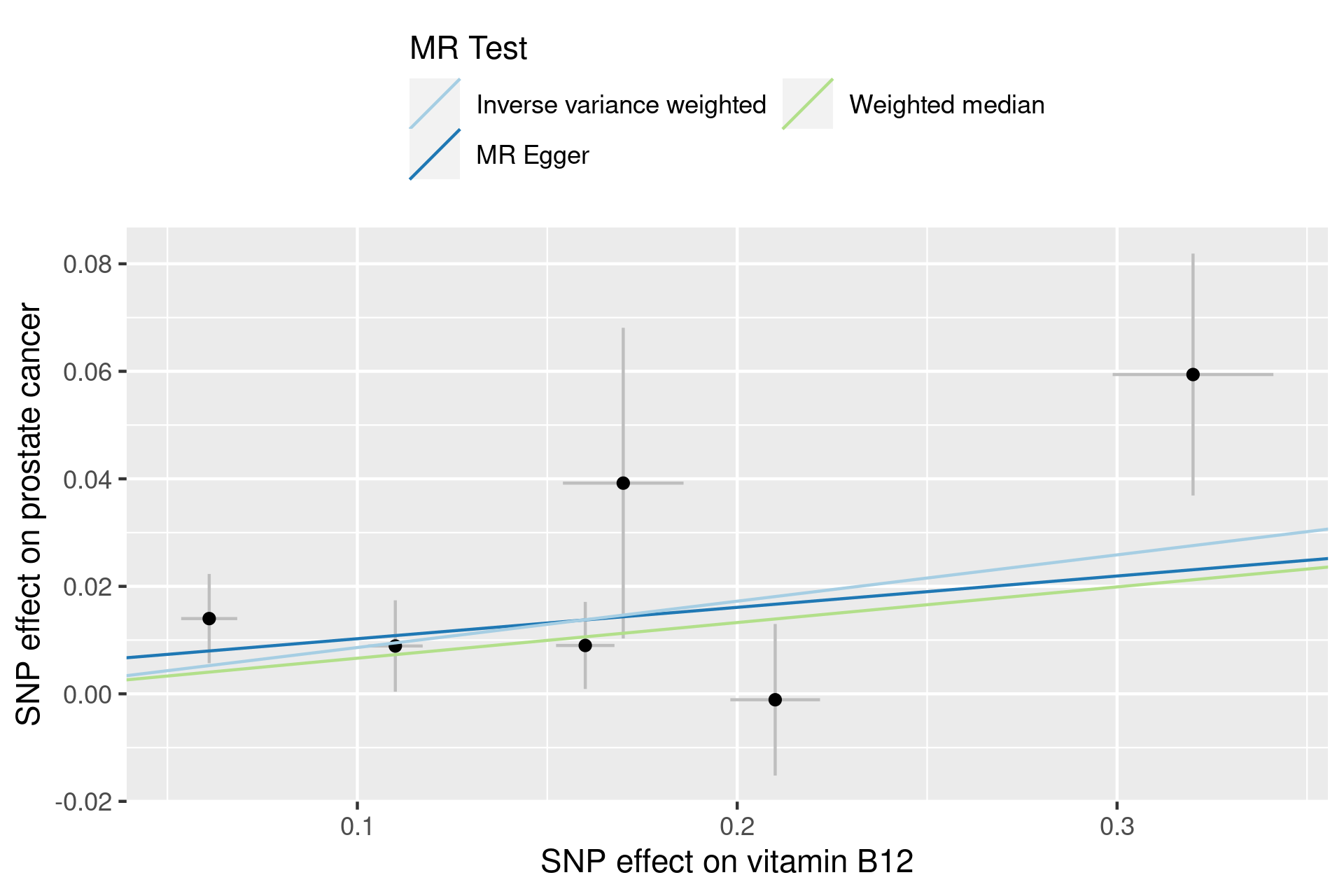


4) Funnel plot


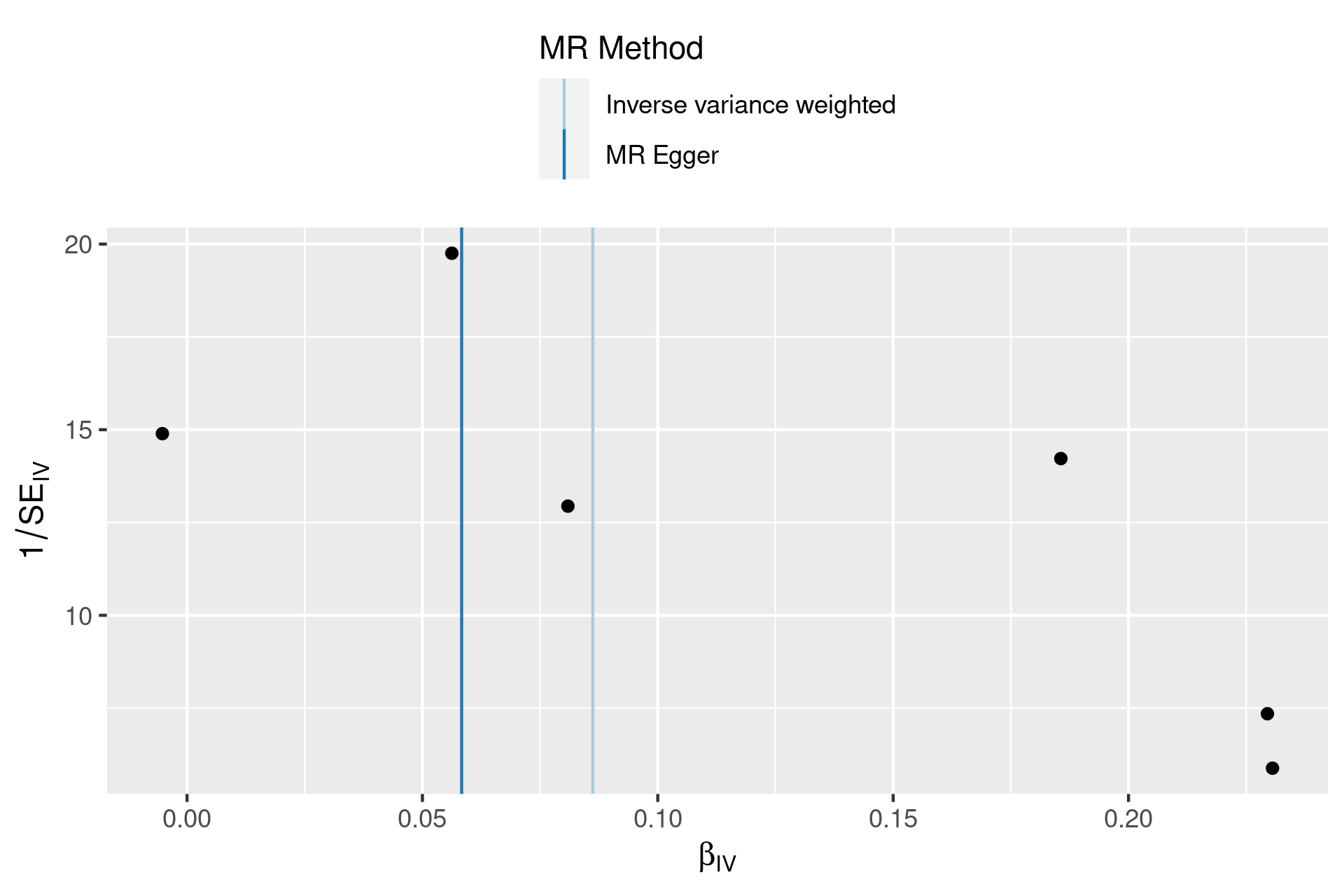


# Supplementary Figure 36. Genetic association of magnesium with ovarian cancer, invasive

1) Forest plot


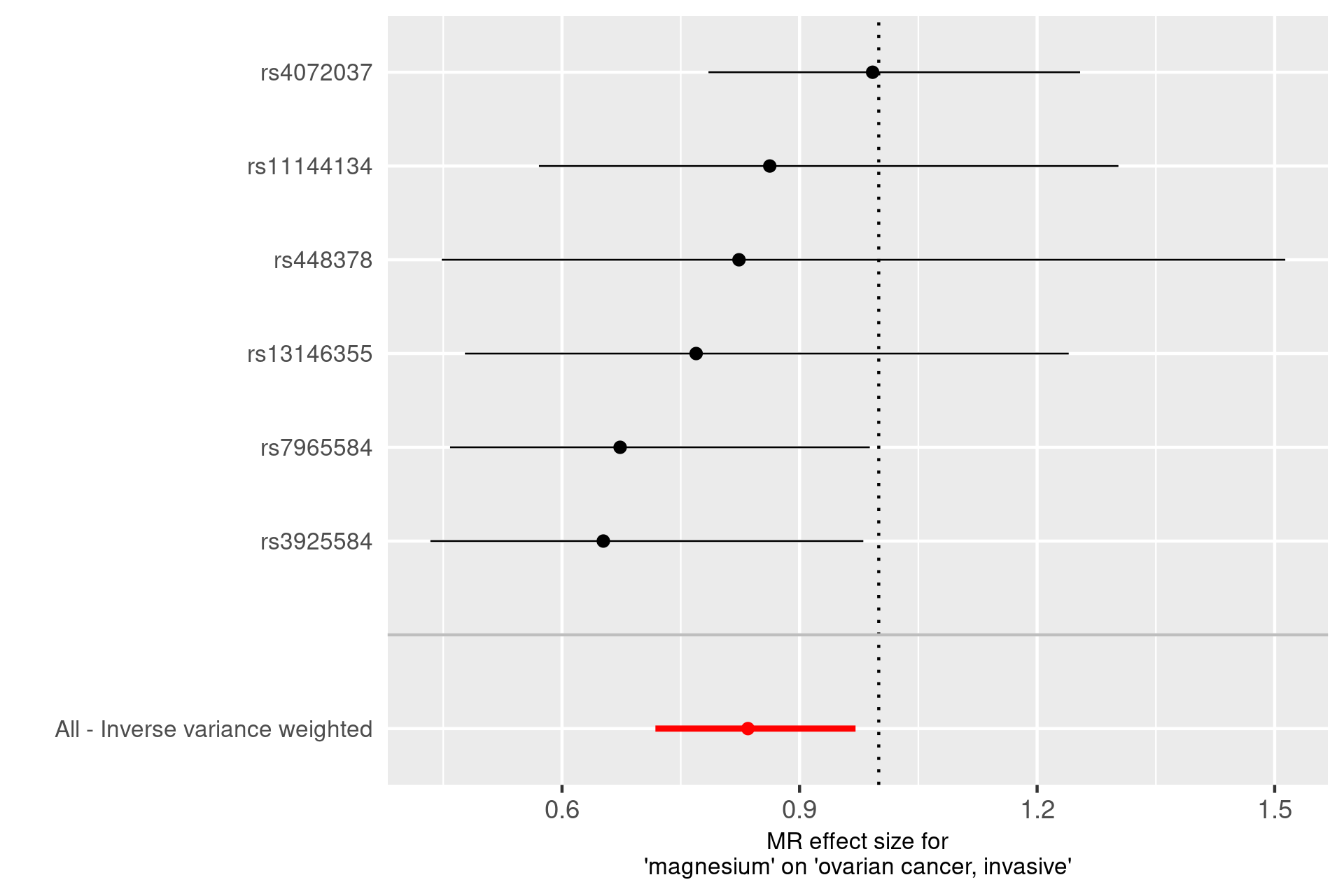


2) Leave-one-out plot


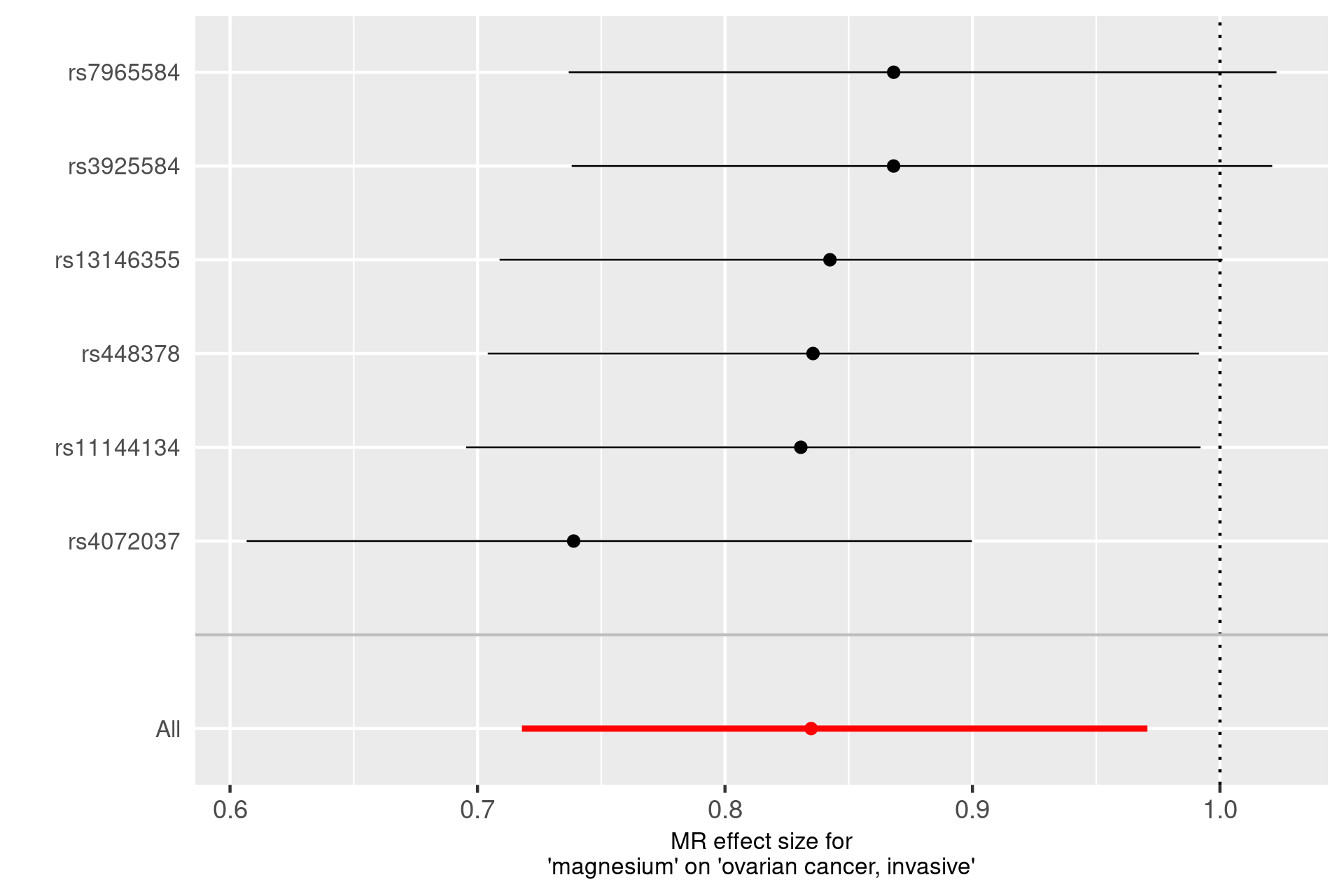


3) Scatter plot


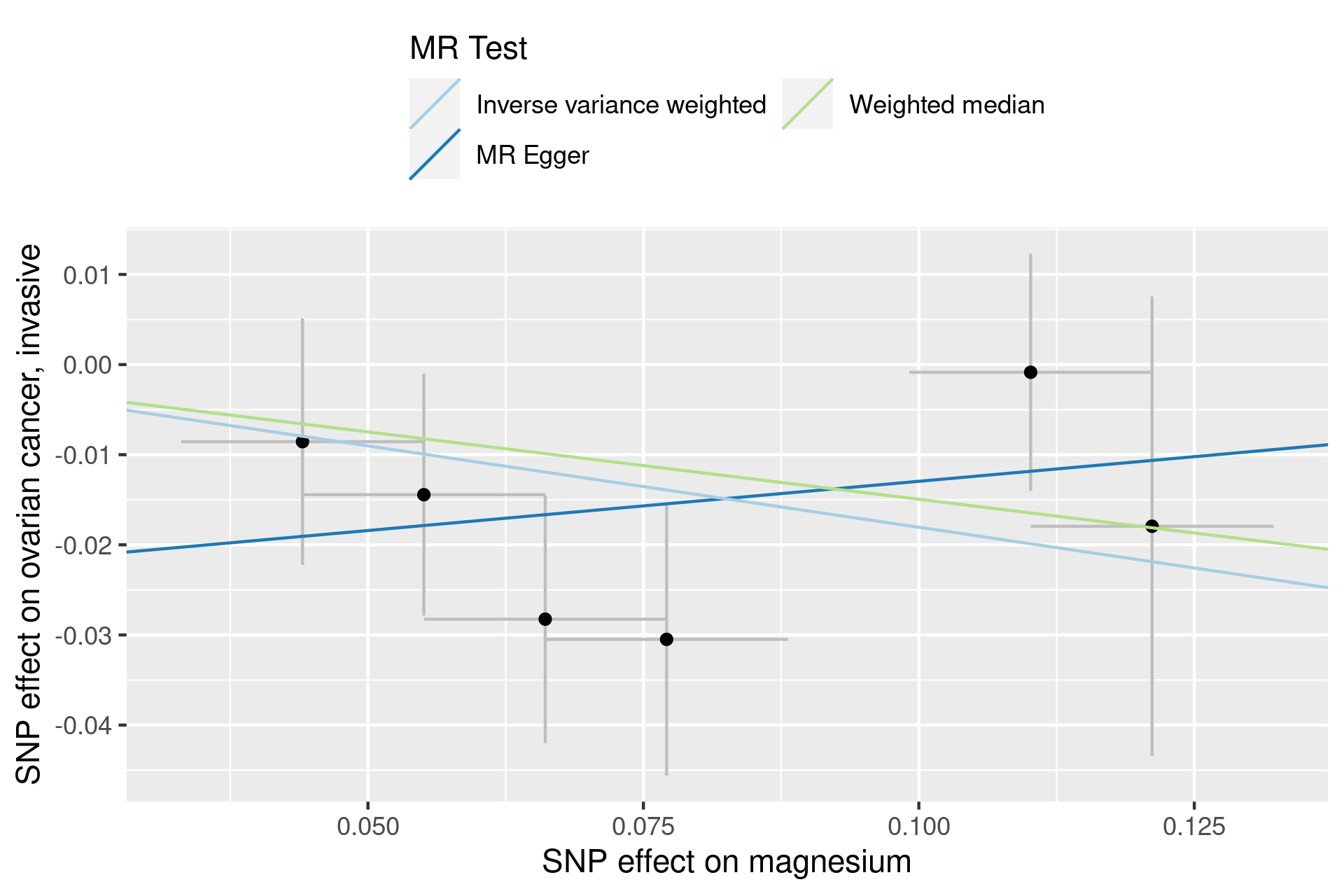


4) Funnel plot


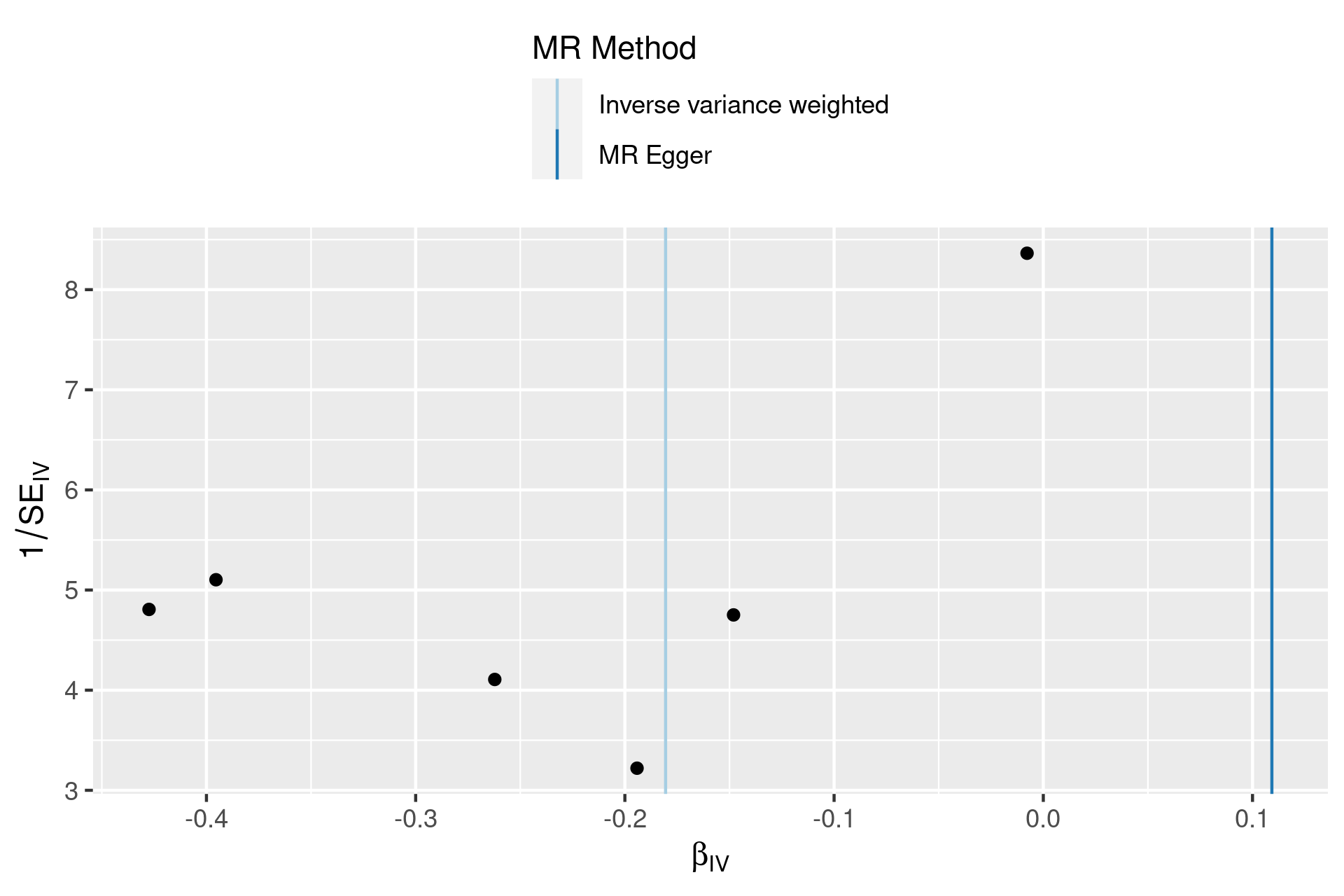


# Supplementary Figure 37. Genetic association of vitamin B12 with colorectal cancer

1) Forest plot


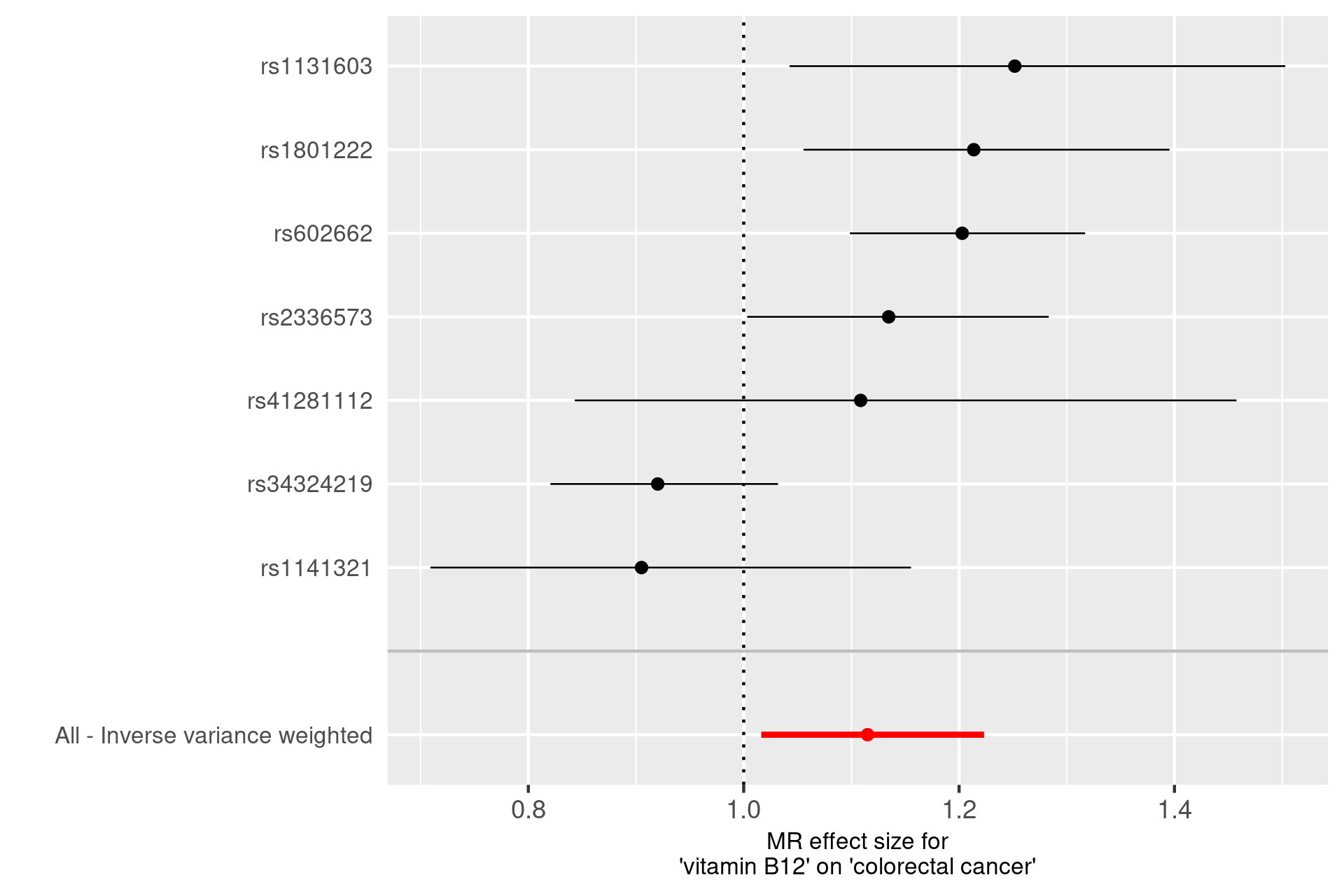


2) Leave-one-out plot


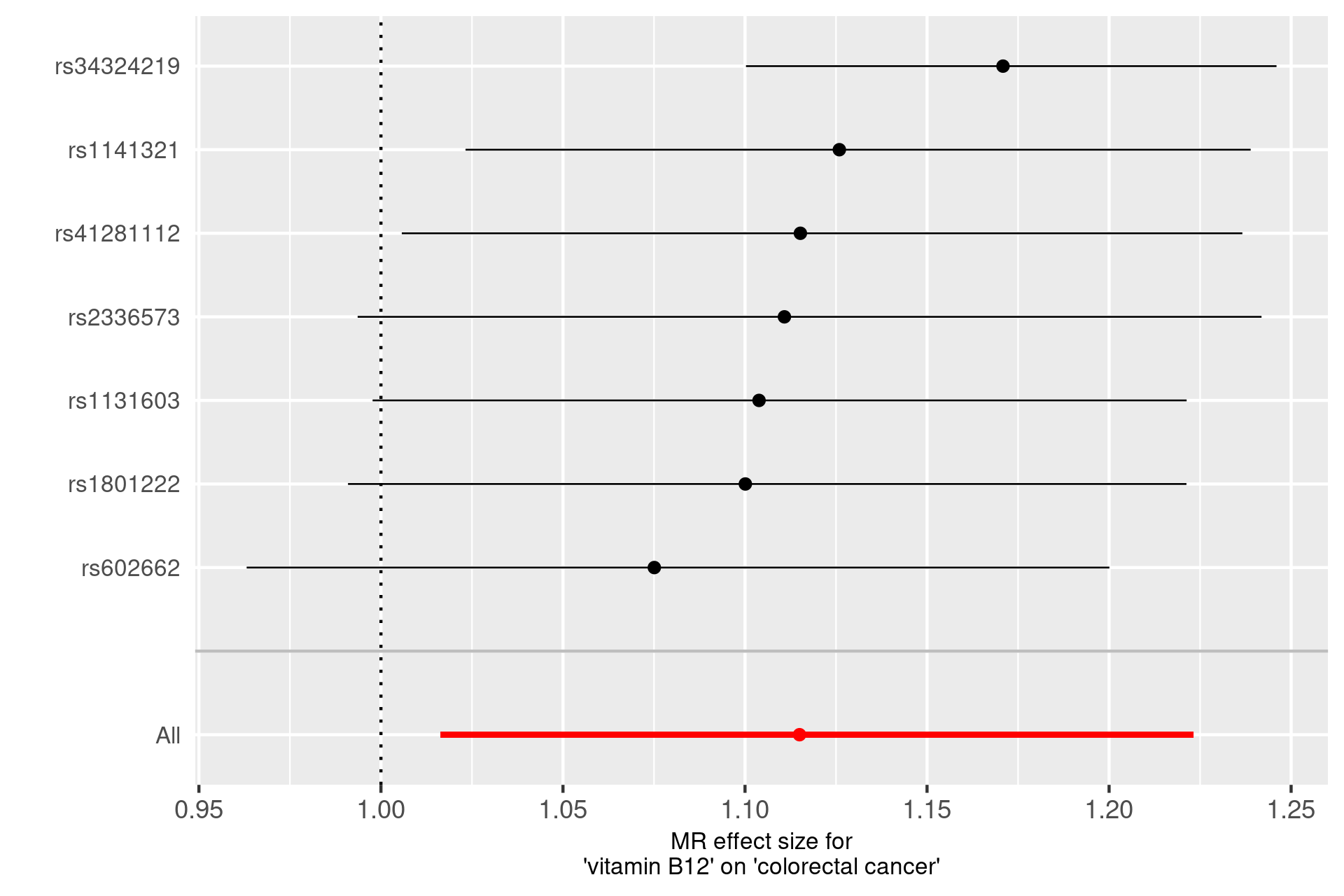


3) Scatter plot


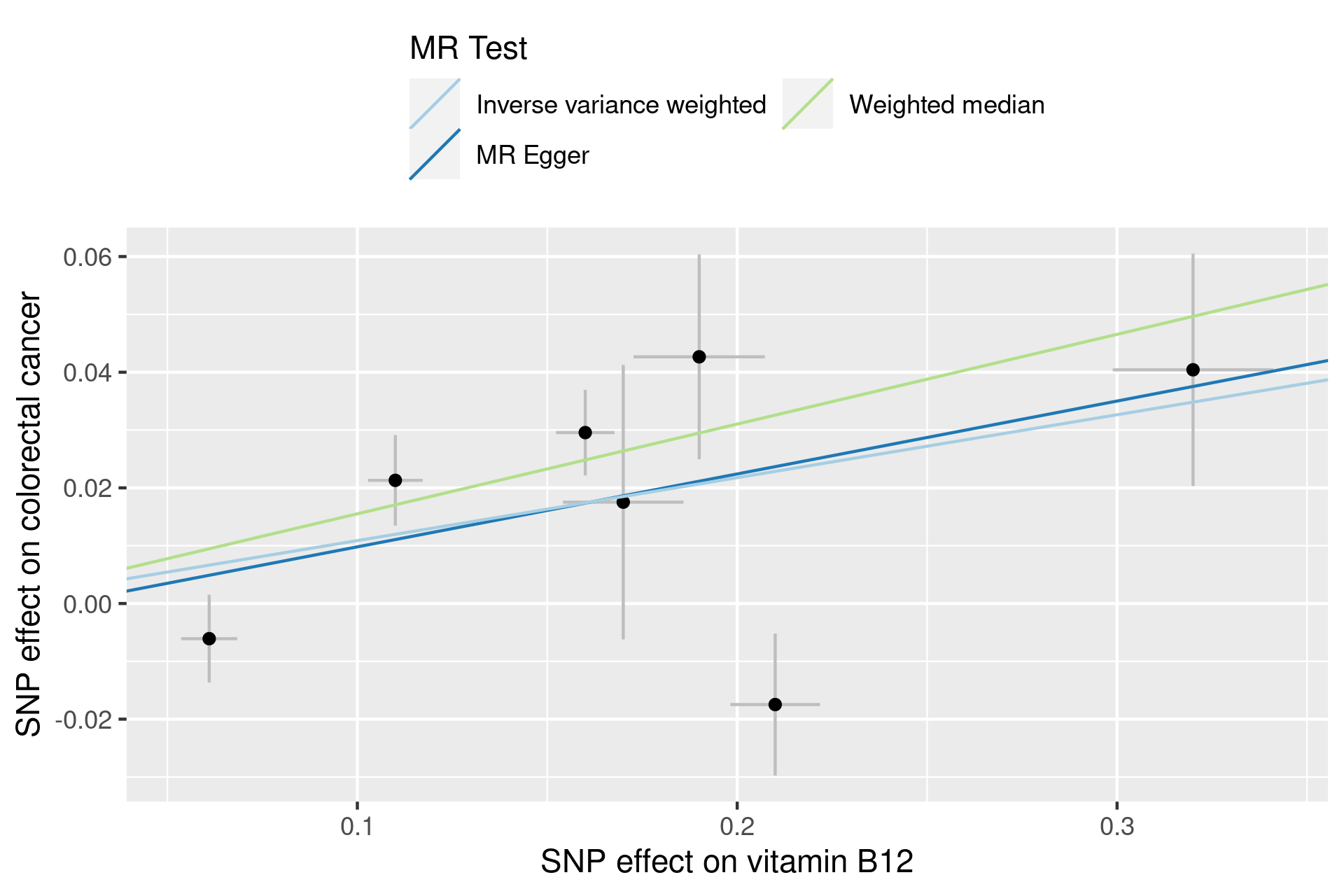


4) Funnel plot


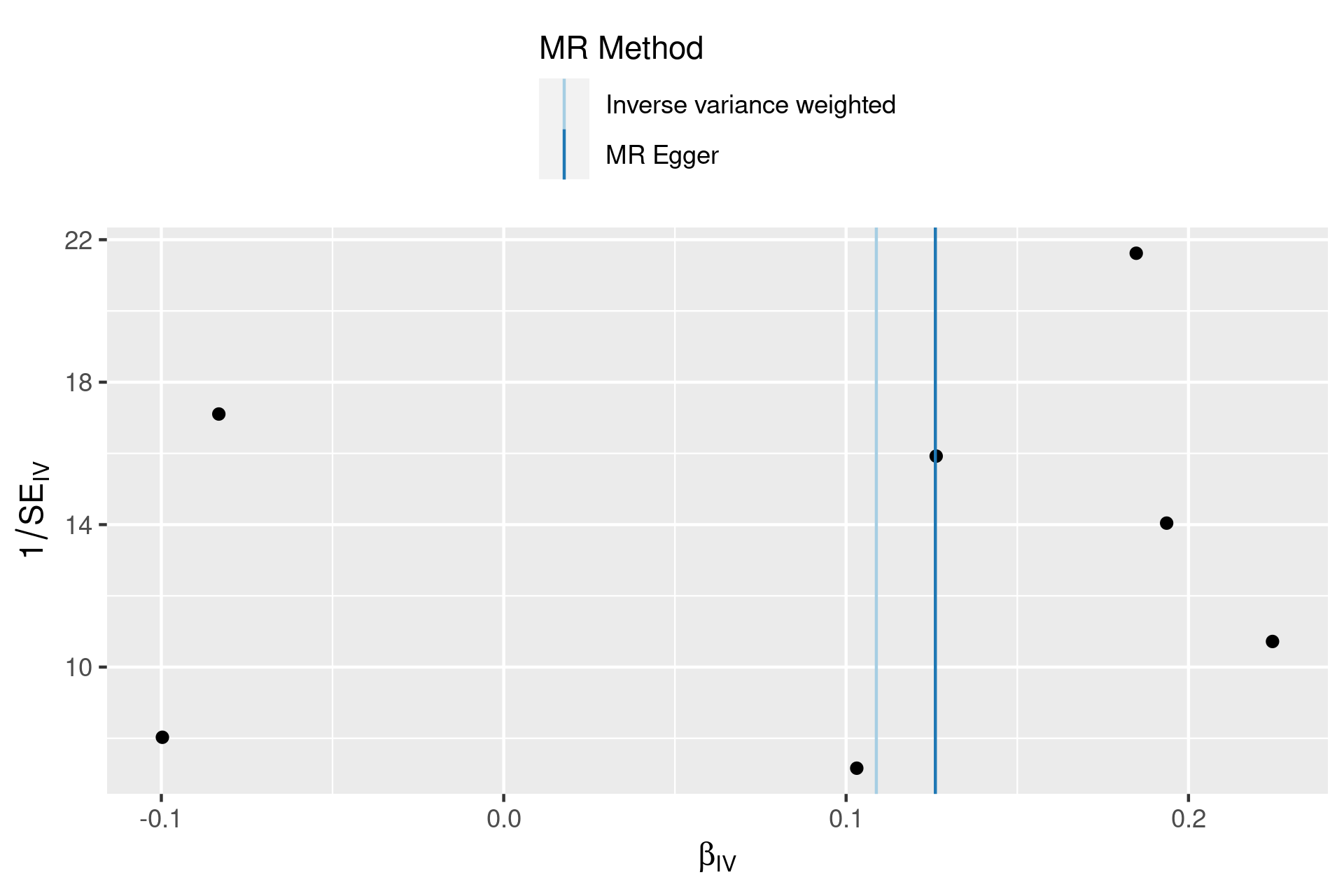


# Supplementary Figure 38. Genetic association of selenium with colorectal cancer

1) Forest plot


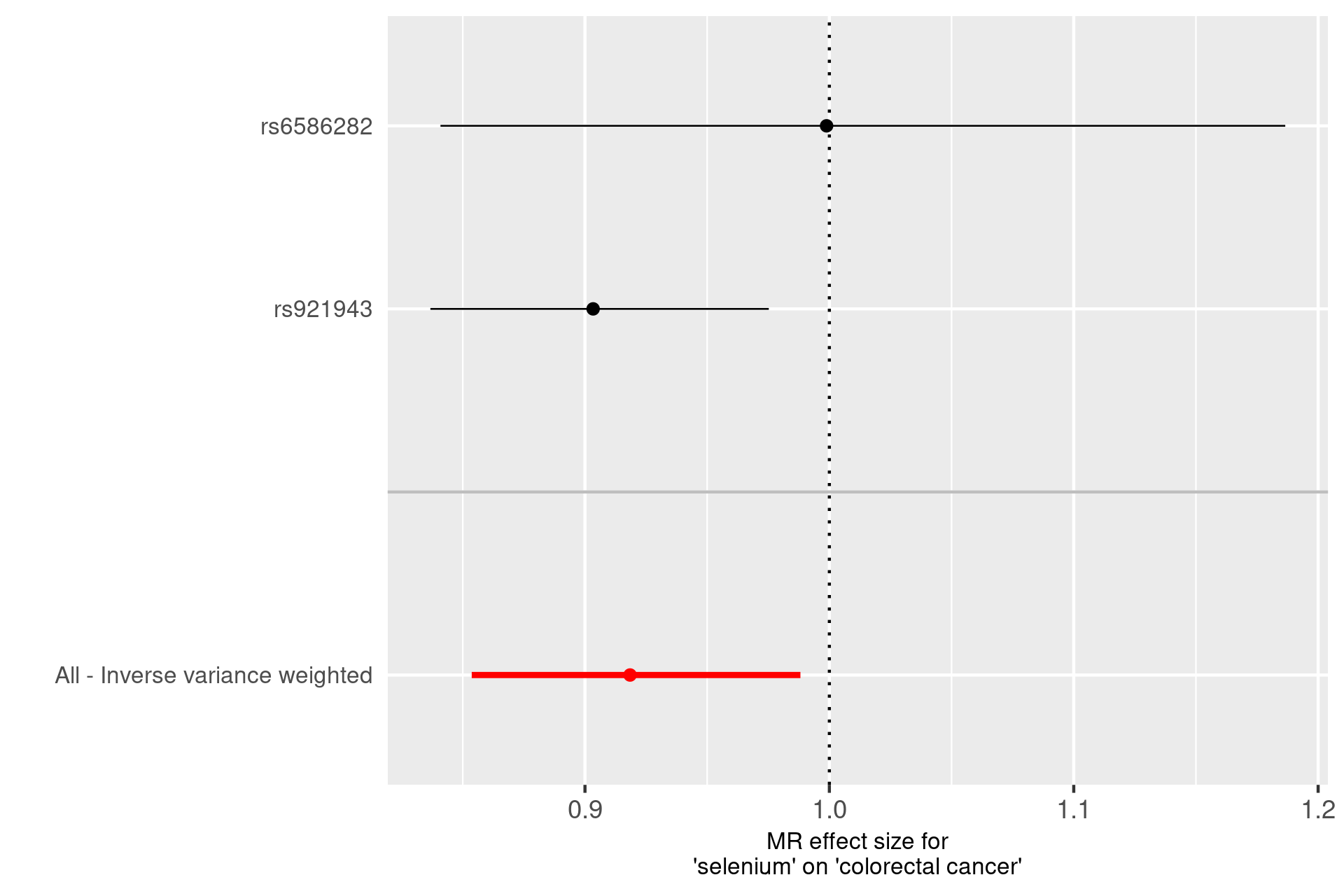


2) Leave-one-out plot

Not available because of small number of SNPs

3) Scatter plot


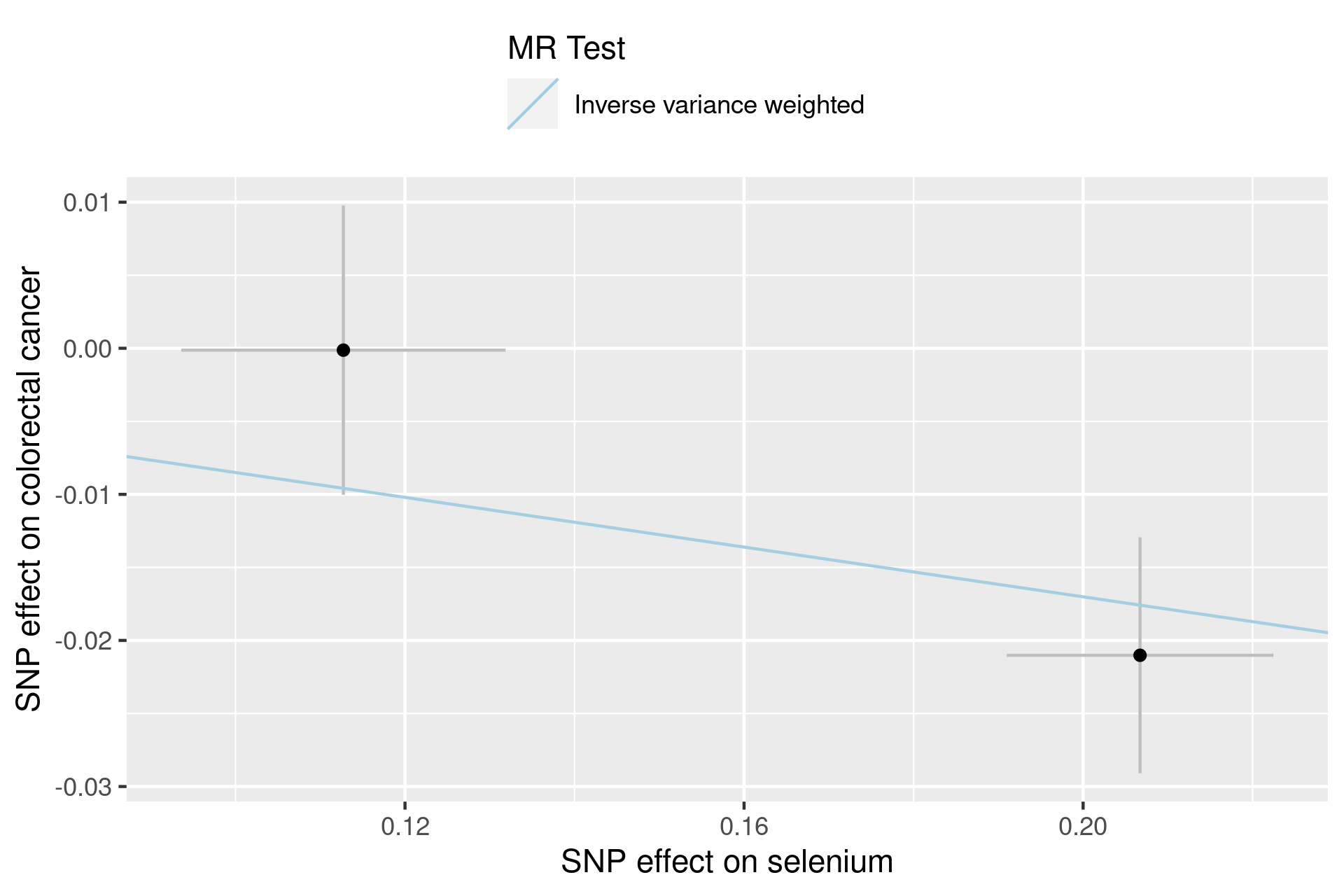


4) Funnel plot


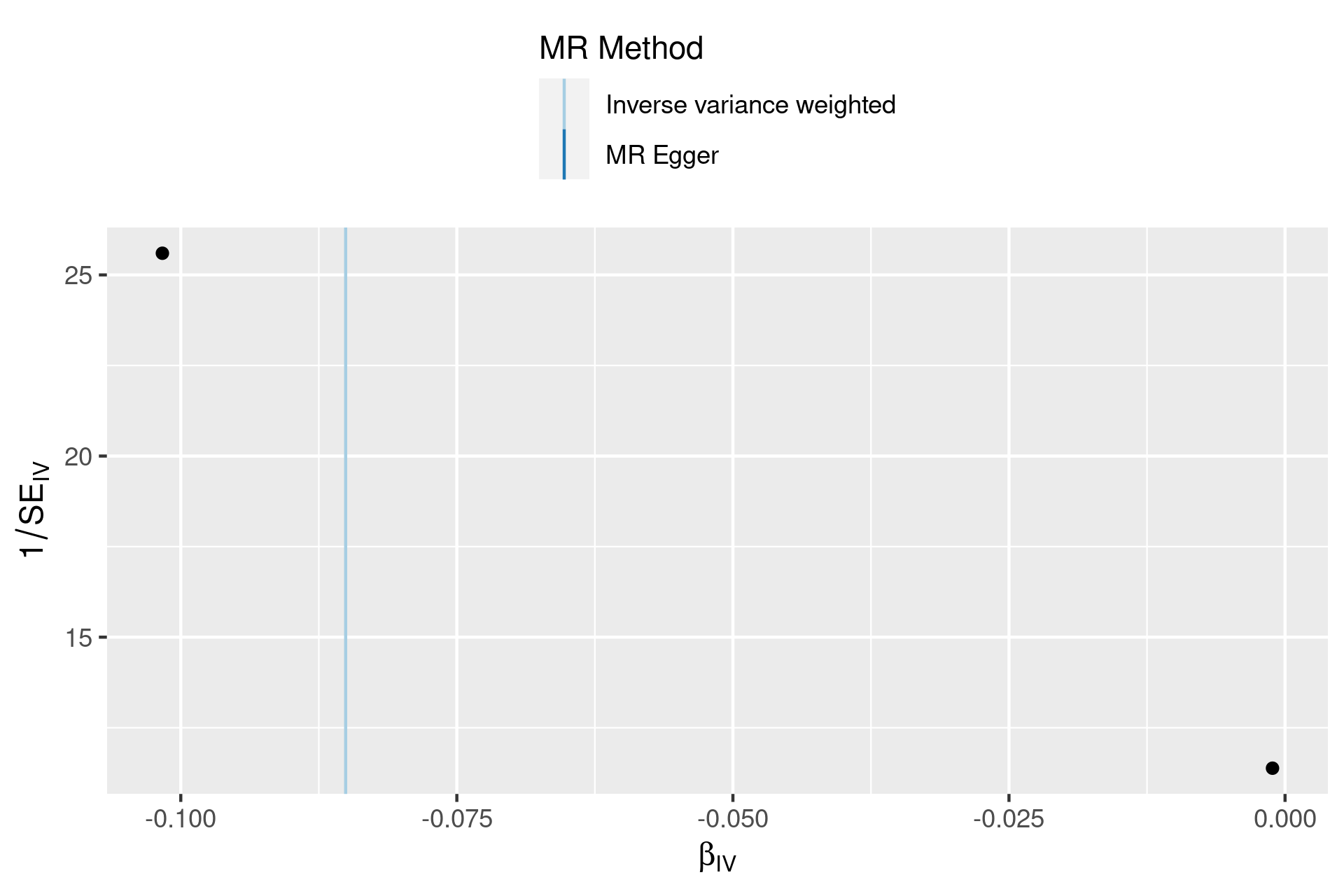


# Supplementary Figure 39. Genetic association of iron with colorectal cancer

1) Forest plot

2) Leave-one-out plot

Not available because of small number of SNPs

3) Scatter plot

4) Funnel plot

# Supplementary Figure 40. Genetic association of zinc with prostate cancer

1) Forest plot

2) Leave-one-out plot

Not available because of small number of SNPs

3) Scatter plot

4) Funnel plot

# Supplementary Figure 41. Genetic association of phosphorus with lung cancer, overall cancer type

1) Forest plot

2) Leave-one-out plot

3) Scatter plot

4) Funnel plot

# Supplementary Figure 42. Genetic association of magnesium with breast cancer, luminal A-like

1) Forest plot

2) Leave-one-out plot

3) Scatter plot

4) Funnel plot

# Supplementary Figure 43. Genetic association of magnesium with ovarian cancer, endometrioid

1) Forest plot

2) Leave-one-out plot

3) Scatter plot

4) Funnel plot

# Supplementary Figure 44. Genetic association of phosphorus with breast cancer, HER2 enriched-like

1) Forest plot

2) Leave-one-out plot

3) Scatter plot

4) Funnel plot

# Supplementary Figure 45. Genetic association of vitamin E with ovarian cancer, non-invasive serous

1) Forest plot

2) Leave-one-out plot

3) Scatter plot

4) Funnel plot

# Supplementary Figure 46. Genetic association of vitamin B12 with lung cancer, adenocarcinoma

1) Forest plot

2) Leave-one-out plot

3) Scatter plot

4) Funnel plot

# Supplementary Figure 47. Genetic association of copper with lung cancer, ever smoker

1) Forest plot

2) Leave-one-out plot

Not available because of small number of SNPs

3) Scatter plot

4) Funnel plot

# Supplementary Figure 48. Genetic association of vitamin C with breast cancer, HER2 enriched-like

1) Forest plot

2) Leave-one-out plot

3) Scatter plot

4) Funnel plot

# Supplementary Figure 49. Genetic association of vitamin B12 with ovarian cancer, non-invasive serous

1) Forest plot

2) Leave-one-out plot

3) Scatter plot

4) Funnel plot

# Supplementary Figure 50. Genetic association of copper with lung cancer, small cell carcinoma

1) Forest plot

2) Leave-one-out plot

Not available because of small number of SNPs

3) Scatter plot

4) Funnel plot

# Supplementary Figure 51. Genetic association of calcium with breast cancer, triple-negative

1) Forest plot

2) Leave-one-out plot

3) Scatter plot

4) Funnel plot

# Supplementary Figure 52. Genetic association of zinc with ovarian cancer, invasive mucinous

1) Forest plot

2) Leave-one-out plot

Not available because of small number of SNPs

3) Scatter plot

4) Funnel plot

# Supplementary Figure 53. Genetic association of vitamin B12 with ovarian cancer, clear cell

1) Forest plot

2) Leave-one-out plot

3) Scatter plot

4) Funnel plot

# Supplementary Figure 54. Genetic association of vitamin B9 (folate) with lung cancer, ever smoker

1) Forest plot

2) Leave-one-out plot

Not available because of small number of SNPs

3) Scatter plot

4) Funnel plot

# Supplementary Figure 55. Genetic association of vitamin D (25-hydroxyvitamin D) with lung cancer, small cell carcinoma

1) Forest plot

2) Leave-one-out plot

3) Scatter plot

4) Funnel plot
